# Anticancer Molecule Discovery via C2-Substituent Promoted Oxidative Coupling of Indole and Enolate

**DOI:** 10.1016/j.isci.2019.11.021

**Published:** 2019-11-16

**Authors:** Helin Lu, Guirong Zhu, Tiange Tang, Zhuang Ma, Qin Chen, Zhilong Chen

**Affiliations:** 1 School of Pharmacy, Huazhong University of Science and Technology (HUST), 13 Hangkong Road, Wuhan, Hubei 430030, China; 2 Research Center for Molecular Recognition and Synthesis, Department of Chemistry, Fudan University, 220 Handan Road, Shanghai 200433, China

**Keywords:** Organic Chemistry, Chemical Compound, Biochemistry

## Abstract

C2, C3-disubstituted indole is one of the most frequently encountered motifs in bioactive alkaloids and medicinal chemistry. Thus, developing novel, concise, and efficient access to it is highly desired in drug discovery. Herein, we present such an approach to this scaffold by direct oxidative coupling of C2-substituted indoles and enolates. Compared with indole bearing no C2-substituent, higher yields (up to 96%) were obtained for C2-substituted indoles in most cases. Mechanistic studies showed the reaction went through a Fe-chelated radical-anion oxidative coupling procedure promoted by C2-substituent on indole by two means: (1) stabilizing C2-radical intermediate during the reaction; (2) reducing indole homocoupling. This approach serves as a synthetic useful tool to quickly build up bioactive small molecule library of C2, C3-disubstituted indoles, and several products showed promising anticancer activities. Besides, indomethacin and its analogs were conveniently prepared in three-step sequence efficiently, indicating the potential application of our approach in medicinal chemistry.

## Highlights

• Fe-mediated, C2-substitutent promoted oxidative coupling of indoles and enolates
• Three-steps to prepare indomethacin and its analogues
• Quick access to complex indoles library bearing anticancer bioactivities

## Graphical Abstract

**Figure:**
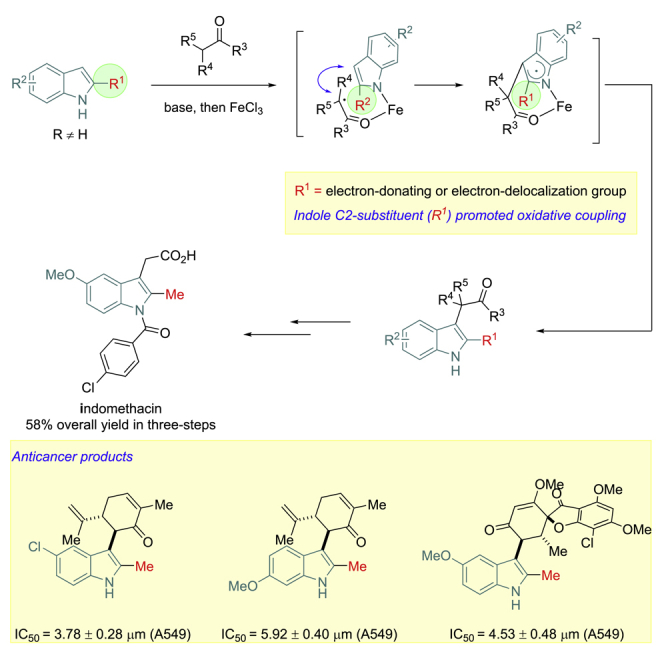

## Introduction

**Figure 1:**
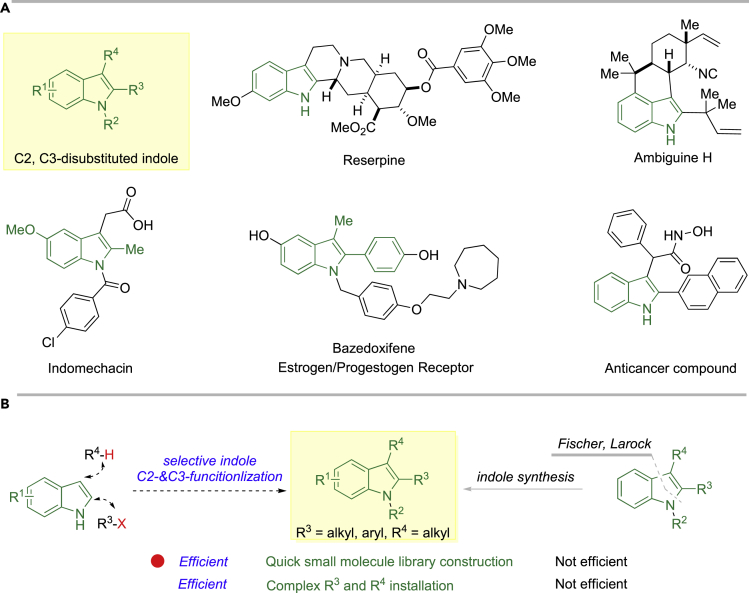
C2-C3-Substituted Indole Scaffolds (A) Representative alkaloids, drugs/drug leads bearing C2-C3-substituted indole scaffolds. (B) Strategy to prepare C2-C3-substituted indoles.

Modern drug discovery still requires tremendous resources and efforts. The chance from target validation to drug approval still remains very low (<10%). How to quickly identify small molecules with good potency and AMDET (absorption, metabolism, distribution, excretion, and toxicity) properties is one of the major challenges for current drug hunting. Developing concise, efficient, and selective synthetic methodologies, somehow, can contribute to solve such a challenge by providing precise and useful molecule-editing tools, which enable medicinal chemists to quickly assemble drug-like small molecule libraries with broader chemical space and accelerate SAR (Structure-Activities Relationship) studies (Dugger et al., 2017, Bostrom et al., 2018, Campos et al., 2019). As embodied in many bioactive natural products, drugs/drug leads, indole is one of the most commonly used drug-like motifs in small molecule drug design (Mase, 2010, Kochanowska-Karamyan and Hamann, 2010, Taber and Tirunahari, 2011, Gribble, 2016), especially the C2, C3-disubstituted ones (e.g., reserpine, ambiguine H, indomethacin, estrogen/progestogen receptor bazedoxifene, and anticancer reagent [Burn and Rand, 1958, Stratmann et al., 1994, Aksenov et al., 2015]) (Figure 1A).

Traditional preparation of C2, C3-disubstituted indole scaffolds, such as Fischer and Larock indole synthesis (Taber and Tirunahari, 2011, Gribble, 2016, Robinson, 1963, Herraiz-Cobo et al., 2015), normally requires multiple steps from commercially available starting materials, particularly for those bearing diverse functional groups. Thus, they are less efficient in access to complex indole molecules, calling for the development of novel, concise, and efficient methodologies. A late-stage, direct, and selective functionalization of indole, on the other hand, would be an ideal strategy to construct them (Wencel-Delord and Glorius, 2013, Cermak et al., 2015). In addition, this strategy could still maintain efficiency even when introducing complex C2- and C3-substitutes (Figure 1B).

**Figure 2:**
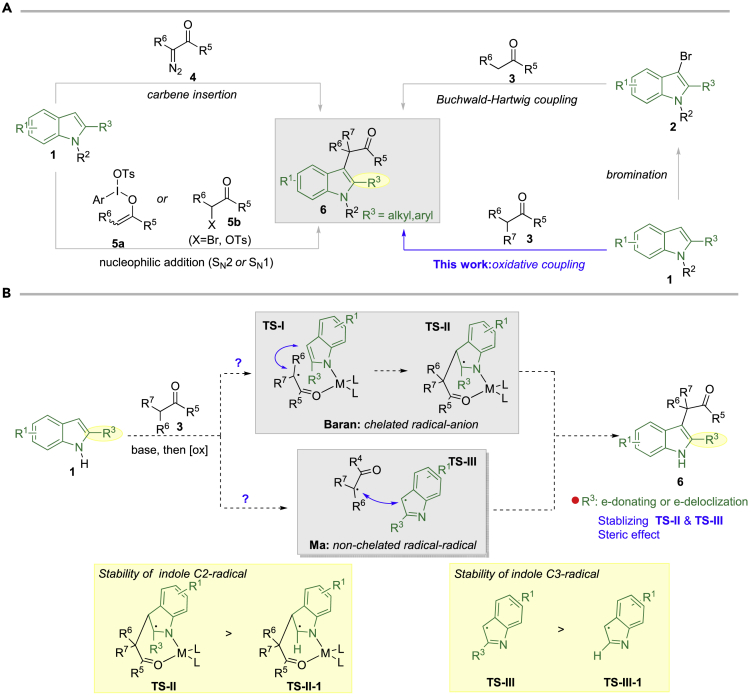
Reported Synthetic Approaches to C2, C3-disubstituted Indoles and Our Reaction Design (A) Previous synthetic methods toward C2, C3-disubstituted indole from C2-substituted indole. (B) Our reaction design via C2-substituent promoted oxidative coupling of indole and enolate.

Driven by our interest in searching for novel small molecules for cancer therapy (Ke et al., 2019), we would like to develop an approach for quick access to these scaffolds by direct and selective connecting carbonyl motifs and C2-substituted indoles, considering that carbonyl moieties are among the most synthetic useful functional groups in organic synthesis. Previous approaches in connecting carbonyl compounds and C3 position of C2-substituted indole mainly rely on carbine insertion (Keller et al., 1977, Gibe and Kerr, 2002), nucleophilic addition (Tang et al., 2012, Vander Wal et al., 2013, Maksymenko et al., 2017), and Buchwald-Hartwig coupling reactions (Esteves et al., 2017). In all these methods, either carbonyl regents (e.g., diazo, α-bromocarbonyl, enonium species) or C3-bromoindole requires additional steps for preparation from corresponding carbonyl compounds or indoles. Therefore, direct coupling of C2-substituted indoles and carbonyl compounds, from the perspective of atom- and step-economy, is more appealing. Moreover, both carbonyl compounds and C2-substituted indoles are either commercially available or can be prepared easily. Therefore, within this approach, simply from C2-substituted indoles and carbonyl compounds, small molecular library of complex C2, C3-disubstituted indoles could be easily constructed, facilitating related medicinal chemistry research (Figure 2A).

Among many excellent coupling strategies, we would like to develop oxidative coupling of C2-substituted indoles and enolates without carbon-metal bond formation, given that aryl halide could not be well tolerated in many transition-metals-catalyzed reactions (Sun et al., 2010, Wendlandt et al., 2011, Liu et al., 2011, Yeung and Dong, 2011, Zhang et al., 2012), which will restrict the derivatization of products to certain extent. Oxidative homocoupling of enolates was discovered in 1935, but limited progress was achieved in the following 50 years (Babler and Haack, 1983, Brocksom et al., 1975, Guo et al., 2012, Ito et al., 1975, Ito et al., 1977, Ivanoff and Spasoff, 1935, Kauffmann et al., 1968, Ojima et al., 1992, Rathke and Lindert, 1971). Baran first reported the intermolecular cross-coupling of indoles and enolates under similar reaction conditions via a copper-chelated radical-anion coupling procedure (Baran and Richter, 2004). By switching the oxidant from Cu(II) salts to iodine, Ma discovered that similar intramolecular reaction worked smoothly as well in a non-chelated radical-radical coupling fashion (Zuo et al., 2010). Inspired by their seminal research, it is reasonable to hypothesize that the intermolecular oxidative cross-coupling of C2-substituted indoles and enolates might also work, thus affording the desired products. According to the reaction mechanism proposed by either Baran (Richter et al., 2007) or Ma (Zuo et al., 2010), certain C2-substitute (Figure 2, *R*^*3*^) on indole (e.g., electron-donating group, electron-delocalization group), we believe, could (Figure 2B, **TS-II, TS-II-1**, **TS**-**III**, and **Ts-III-1**) promote the reaction by stabilizing the electrophilic indole C2-/C3-radicals intermediates albeit its harmful steric repulsion.

## Results and Discussion

### Condition Optimization for Oxidative Coupling of C2-Substituted Indole and Enolate

2-Methyl indole (**1-1**) and (*R*)-carvone (**3-1**) were selected as model substrates to investigate this reaction. Initially, we utilized Baran's condition (Richter et al., 2007) to test our hypothesis. However, the reaction failed to complete, producing low yield of the desired product **6-1** (Table 1, entry 2). Then we turned our attention to Ma's condition (Zuo et al., 2010), but disappointing result was observed (Table 1, entry 3). Other oxidants, like Cu(OAc)_2_ and Fe(acac)_3_, also failed to afford the product in good efficiency (Table 1, entry 7). To our delight, the yield was greatly improved in the presence of 4.0 equivalents of FeCl_3_ (Artz and Cram, 1984) (Table 1, entry 4). Further improvement was achieved by maintaining the overall reaction at −78°C after adding FeCl_3_ (57% versus 80%, Table 1, entry 6 and 7). Ultimately, the yield could be increased to 89%–93% by adding FeCl_3_ in THF solution rather than as solid in open air (Table 1, entry 1). Other oxidants, like AgO, AgOAc, CAN, and CeCl_3_, failed to give better yield. Changing the reaction solvents from THF to others, like DMF and toluene, did not enhance the efficiency either (Table 1, also see Table S2).

**Table 1:** Condition Optimization


Entry^a^	Deviation from "Standard Condition"	Yield (%)/dr^a^	
1	None	89–93/>20:1	
2	Cu(2-ethylhexanoate)_2_ (1.5 eq.) instead of FeCl_3_ (Baran's condition)	26–30^b^/>20:1	
3	I_2_ instead of FeCl_3_ (Ma's condition)	ND	
4	FeCl_3_ was added in one portion as solid, then −78°C	80/>20:1	
5	FeCl_3_ was added in one portion as solid, then −78°C to rt, 30 min	57/>20:1	
6^c^	Fe(acac)_3_ (3.0 eq.) instead of FeCl_3_	20/>20:1	
7^c^	Cu(OAc)_2_ (3.0 eq.) instead of FeCl_3_	46/>20:1	
8	LDA instead of LiHMDS	41/>20:1	
9	Toluene instead of THF	41/>20:1	
10^c^	With 1.0 equivalent of 2-methyl indole	28/>20:1	

a All the reactions were conducted with 1-1 (2.0 mmol, 2.0 equiv.), (*R*)-carvone (3-1, 1.0 mmol, 1.0 equivalent), LiHMDS (1.3 M in THF, 4.0 mmol, 4.0 equivalent), and FeCl_3_ (99%, 4.0 mmol, 4.0 equivalent in THF), isolated yield, diastereomer ration (dr) was determined by ^1^H NMR of isolated product 6-1.

b Cu(2-ethylhexanoate)_2_ (1.5 mmol, 1.5 eq.) was added at −78°C and then warmed to room temperature.

c The oxidants were added at −78°C and then warmed to room temperature and the reaction mixture was stirred for 2 h; ND = no desired product.

#### Mechanistic Investigations

**Figure 3:**
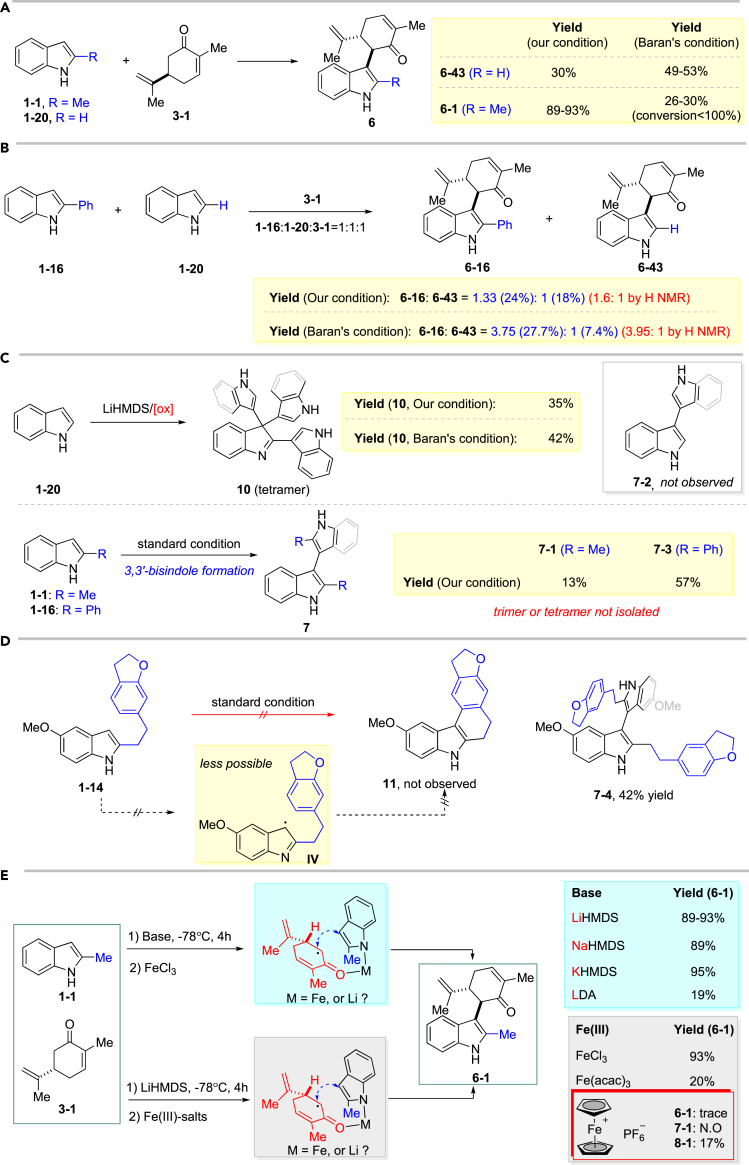
Mechanistic Investigation (A) Control experiments between 2-methylindole (**1-1**) and indole (**1-10**). (B) Competing experiments between 2-phenylindole (**1-16**) and indole (**1-20**), also see Figure S6. (C) Background reaction from homocoupling of indole (**1-20**), 2-methylindole (**1-1**), and 2-phenylindole (**1-16**), also see Figure S7 and S8. (D) Indole C3-radical trapping experiments, also see Figure S9. (E) Experiments to identify the chelating effects, also see Table S3.

This highly efficient transformation has intrigued our great interest, since it somehow proved our hypothesis that C2-substituent could promote the reaction. To further understand this reaction, several control and competing experiments were conducted. First, we compared Baran's and our condition for indole (**1–20**)/2-methylindole (**1-1**) in coupling with (*R*)-carvone (**3-1**), respectively, as shown in Figure 3A. Higher yield was obtained from 2-methylindole (**1-1**) under our reaction; in contrast, Baran's condition favored indole (**1–20**), suggesting that these two conditions can be complementary for each other depending on the indole substrates. Such evident difference between Cu(II) and Fe(III) in this reaction probably originated from their coordination ability and oxidation potential. And Fe(III) can better tolerate the steric effects from C2-substituent on indole, giving higher yield. To our surprise, in a competing experiment with (*R*)-carvone (**3-1**), 2-phenylindole (**1-16**) afforded more coupling product **6-16** than indole (**1-20**) under both conditions, although 2-phenylindole (**1-16**) is more steric hindered and less electron rich, which further supported our hypothesis about C2-substituent-promoted coupling (Figure 3B, see also Figure S6). Meanwhile, less tetramer of indole (**10**) was isolated in the background reaction under our condition, and no dimer product **7-2** was observed. Interestingly, only dimmer of C2-methyl/phenylindole (**7-1**, **7-3**) was isolated when indole **1-1** and **1-16** were subjected to the same background reactions, which can serve as a complementary approach for synthesis of 3,3′- bisindoles. Apparently, all these experiments illustrated that both C2-substitutent and our reaction condition contributed to reducing the homocoupling of indole (Figure 3C, also see Figures S7 and S8).

Both products **6** and **7**, as reported by Scott (Scott et al., 1964) and Xia (Deng et al., 2014), could be generated from the dimerization of indole C3-radicals (**III**). Therefore, compound **1-14** was utilized to probe such possibility. However, no anticipated cyclized product **13** was observed; instead, only product **7-4** was isolated, indicating that the reaction might not involve indole C3-radical in our reaction (Figure 3D, also see Figure S9).

Furthermore, to figure out whether this oxidative coupling went through Fe- or Li-chelated or non-chelated procedure (Richter et al., 2007, Casey and Flowers, 2011), different base (MHMDS, M = Li, Na, K, and LDA) and Fe(III)-oxidants were probed. All MHMDS afforded nearly the same results except evident lower yield from LDA (Jahn and Hartmann, 2001). Much inferior efficiency was observed with Fe(acac)_3_ bearing strong ligand, and only low yield of dimmer of (*R*)-carvone (**3-1**) was isolated in the presence of non-cheating [FeCp_2_]PF_6_ along with trace amount of **6-1**. These results clearly suggested that the reaction went through a Fe-chelated radical-anion coupling procedure (Figure 3E, also see Table S3).

#### Proposed Reaction Mechanism

**Figure 4:**
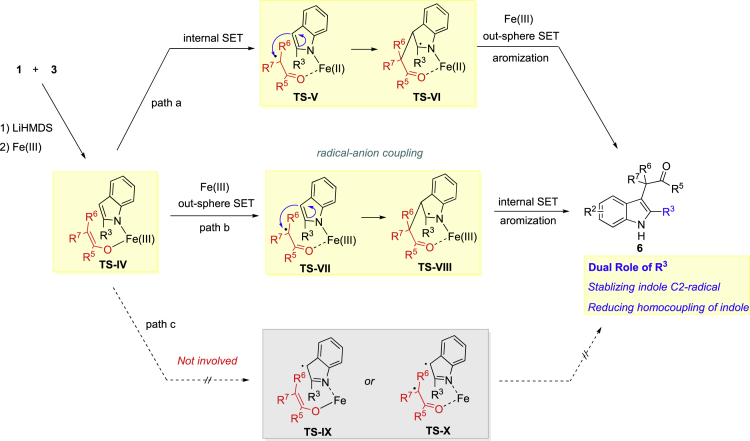
Proposed Reaction Mechanism

Based on information obtained from the mechanistic studies, we proposed reaction mechanism as following. Overall, the reaction went through Fe(III)-Fe(II)-mediated radical-anion coupling procedure (Neumann and Kochi, 1975, Al-Afyouni et al., 2014, Cassani et al., 2016, Mako and Byers, 2016). After deprotonation, FeCl_3_ chelated with indole anion and enolate (**TS-IV**), followed by an internal or out-sphere single-electron-transfer (SET) process affording enolate radical intermediate **TS-V** or **TS-VII**, which was then trapped by indole to generate indole C2-radical **TS-VI** or **TS-VIII** via radical-anion coupling (path a, b). Such indole C-2 radials were then oxidized into indole, affording the desired product **6**. The C2-substituent, as shown in **TS-VI** or **TS-VIII**, can stabilize such indole C2-radical intermediates, thus enhancing the reaction efficiency. On the other hand, the indole C3-radical intermediate, either **TS-IX** or **TS-X**, was less possibly involved. Taking into account that the dimerization of (*R*)-carvone (**8-1**) still occurred even in the presence of [FeCp_2_]PF_6_ (Figure 3E), evidently the enolates can transfer into corresponding radicals via out-sphere SET process. Thus, path b is more likely. Based on all the mechanistic studies, C2-substituent of indole plays dual roles in promoting the reaction albeit the steric repulsion: (1) stabilizing the indole C2-radical intermediates; (2) reducing homocoupling of indole (Figure 4).

#### Evaluation of C2-Substituted Indole as Coupling Partner

Within the optimized condition at hand, the C2-substituted indole scope for this reaction was probed first as shown in Table 2. Generally, indoles bearing electron-donating groups afford the desired products in relatively higher yield compared with those bearing electron-withdrawing ones (**6-2**, **6-6** versus **6-3**, **6-4**; **6-6** versus **6-10**). More steric effects hurt the reaction. For example, dropped yield was observed from 2-ethyl-indole (**6-11**), and no desired product could be isolated using 2-*tert-*butylindole (**6-12**). C2, C4-disubstituted indole can also be tolerated in this reaction, given that its huge steric hindrance might push the carbonyl substrate away (**6-9**). Indoles bearing complex moieties on C2-position, like dihydrobenzofuran and phthalimide, also worked smoothly in this reaction (**6-14** and **6-15**). C2-(hereto)aryl indoles also worked under the standard condition (**6-16**–**6-18**). Pyridine motif can be tolerated too, given that it is a good ligand to iron leading to inhibition of the reaction (**6-19**). Notably, the difference of the yields between product **6-16** and **6-18** (electron-rich arene > electron-deficient arene) also supports our hypothesis about the electrophilic nature of indole C2-radical.

**Table 2:** Scope of C2-substituted Indole


R^1^	R^2^	Product, Yield	R^1^	R^2^	Product, Yield
	H	**6-1**, 93%(78%^b^)		H	**6-11**,77%
	5-Me	**6-2**, 96%		H	**6-12**, ND
	5-Cl	**6-3**, 78%			**6-13**, 63%
	5-F	**6-4**, 67%			**6-14**, 95%
	5-Br	**6-5**, 77%		H	**6-15**, 63%
	5-OMe	**6-6**, 89%			**6-16**, 74%
	6-Me	**6-7**, 91%			**6-17**, 69%
	6-OMe	**6-8**, 70%			**6-18**, 23%
	4-OMe	**6-9**, 53%			**6-19**, 39%
	4-F, 5-OMe	**6-10**, 69%			


^a^All the reactions were conducted under standard condition with 1.0 mmol of (*R*)-carvone (3-1) and 2.0 mmol indole unless noted, isolated yield.

b Gram scale; ND = no desired product.

#### Evaluation of Carbonyl Compounds as Coupling Partner

The carbonyl substrate scope was explored as shown in Table 3. Various carbonyl compounds embodying C_α_-H could be tolerated in the reaction. Diverse cyclohexanones can afford the desired products in moderate to good yields (**6-20**–**6-23**, **6-32**, **6-34**–**6-36**). Quite interestingly, simple cyclopentanone failed to afford any product under standard reaction condition (**6-31**), whereas its derivatives, *cis*-jasmon and isojasmone, gave products in moderate yield (**6-26**, **6-27**). Utilizing stronger base, LDA, instead of LiHMDS, did afford the product **6-31**. Cycloheptanone and cyclooctenone with ring-strain can also react smoothly under standard condition (**6-24**, **6-25**). The desired products were isolated in moderate to good yield from linear enone, ester, lactone, and amide (**6-28**, **6-29**, **6-38**–**6-40**). It is worth pointing out that the thioester could also afford the desired product in moderate yield, which was not reported in previous research about oxidative coupling of enolates (**6-39**). The methyl ketones, prone to dimerization as documented in the literature (Richter et al., 2007), also yielded the cross-coupling products (**6-41**, **6-42**).

**Table 3:** Enolate Scope


					
	**6-20**, 46%	6-21, 63%	**6-22** 84%, >20:1 dr	**6-23**, 79%	**6-24**, 64%
				
	**6-25**, 72%	**6-26**, 54%	**6-27**, 48%	**6-28**, 22%	**6-29**,42%
					
	**6-30** 66%, (1.9:1 dr)	**6-31**, 39%^b^	**6-32**, 49%	**6-33**, 33%	**6-34**, 41%
				
	**6-35**, 64%	**6-36**, 36%	**6-37** 89%, (2.4:1 dr)	**6-38** 56%, (2.2:1 dr)	**6-39**, 66%
			
	**6-40**, 58%	**6-41**, 16%	**6-42**,16%		

^a^All the reactions were conducted under standard condition with 1.0 mmol of carbonyl compound and 2.0 mmol indole unless noted, isolated yield.

b LDA was used instead of LHMDS.

#### Bisindole Products Formation

**Figure 5:**
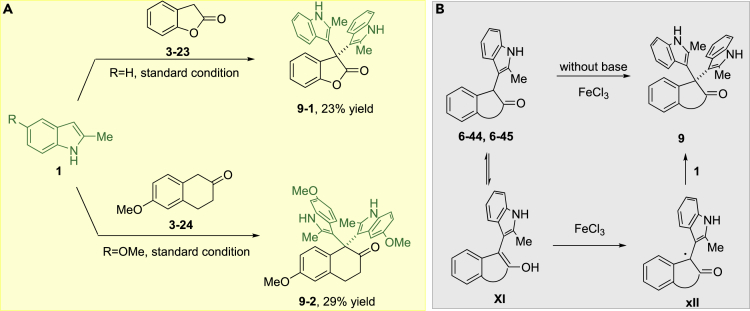
Bisindole Products Formation (A) Bisindole products observed in the reaction. (B) Proposed reaction mechanism for bisindole product formation.

Quite interestingly, instead of the normal coupling product **6-43** and **6-44**, the highly steric hindered bisindole **9-1** and **9-2** were obtained from cyclized benzylenolate **3-23** and **3-24**, respectively. We suspected that the normal product **6-44/6-45** could easily be oxidized into enolate-radical intermediate **XII**, which was stabilized by two arenes followed by coupling with another indole in the presence of FeCl_3_ (Wu, et al., 2015) (Figure 5).

#### Limitation of Substrate Scope

**Figure 6:**
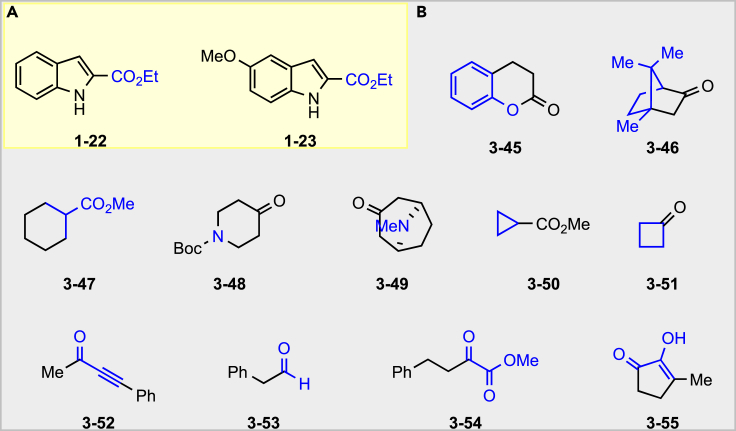
Failed Substrates (A) Failed indole substrates. (B) Failed carbonyl substrates.

Although our reaction expands the landscape for oxidative coupling of indole and enolate, providing a concise access to construct diverse C2, C3-disubstituted indole scaffolds, however, limitation of substrate scope still exists as shown in Figure 6. Extremely low-yield oxidative coupling product (2% isolated yield) was isolated from C2-carboxylate indoles (**1-22** and **1-23**). For the carbonyl compounds, no desired product was obtained when chroman-2-one (**3-45**) was investigated; instead, an amide product from indole opening the lactone was isolated. Other carbonyl compounds, like those bearing highly steric hindrance (**3-46** and **3-47**), heterocycles (**3-48** and **3-49**), small rings (**3-50** and **3-51**), ynone (**3-52**), aldehyde (**3-53**), or ɑ-ketone-ester (**3-54**), all could not afford the desired coupling products. About 8% yield of the desired oxidative coupling product was isolated when cyclopentane-1,2-dione **3-55** was utilized in this reaction, although 1,2-diketone has never been studied in oxidative coupling of enolates. The intramolecular reaction was also conducted; however, the desired ring-closing products were failed to be isolated (Figure S6, also see Figure S5).

### Synthetic Application

#### Synthesis of Indomethacin and Its Analogue

**Figure 7:**
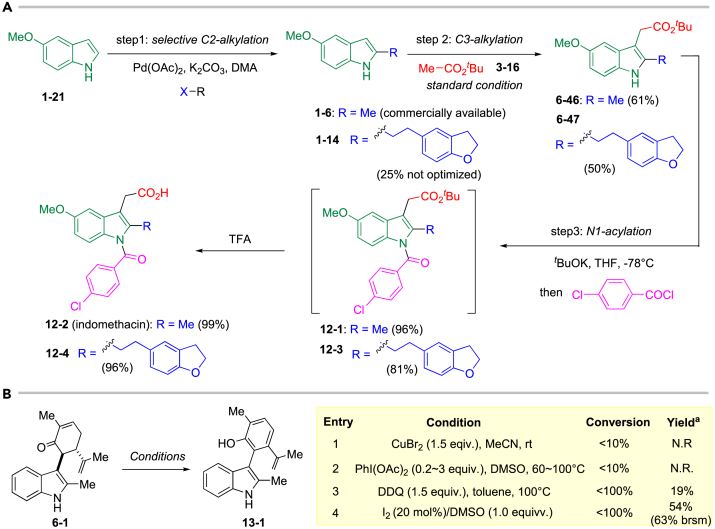
Synthetic Application (A) Three steps to prepare indomethacin and its analogue, see also Figure S4. (B) C3-aryl indole synthesis via aromatization, see also Table S1.

Indomethacin is a selective COX-1 (cyclooxygenases-1) inhibitor, which has been broadly used as an anti-inflammatory drug for decades. Recent research showed that inhibition of COX is beneficial for cancer therapy by inhibiting drug resistance and immune evasion of certain cancer cells (Yu et al., 2009, Vinogradova et al., 2011, Zelenay et al., 2015, Hashemi et al., 2019). Therefore, design and synthesis of novel selective COX inhibitors based on indomethacin can potentially benefit cancer therapy. Previous research about indomethacin analogues synthesis required multiple steps (Rosenbaum et al., 2015, Arisawa et al., 2012); thus, developing concise and efficient synthetic routes for synthesis of indomethacin analogues is important. Based on this reaction, both indomethacin and its analogues could be prepared in a three-step sequence. Under the standard reaction condition, 2-methyl-5-methoxy indole (**1-6**) and *tert-*butyl acetate smoothly afford the precursor of indomethacin **6-46** in moderate yield (61%). After installing the 4-chlorobenzoyl moiety on N1-position and deprotection of *tert-*butyl with trifluoroacetic acid (TFA) sequentially, indomethacin **12-2** was obtained in excellent yield (58% total yield). The indomethacin analogue was prepared conveniently and similarly from indole: selective C2-functionation, C3-alkylation via oxidative coupling, and N1-acylation. For example, the selective C2-alkylation of indole **1-21** could be conveniently achieved to afford **1-14** under Bath's condition (Jiao and Bach, 2011). The desired product **12-4** can be obtained in good yield followed by the same procedure as synthesis of indomethacin smoothly (Figure 7A, also see Figure S4).

#### C3-Aryl Indole Construction

C3-aryl indole scaffolds can also be found in many bioactive small molecules (Liu et al., 2000, Güzel et al., 2009, Luz et al., 2015). We hypothesized that the oxidative aromatization of carvone moiety in product could afford such scaffold. Therefore, several transformations were utilized to transfer compound **6-1** into C3-aryl indoles. Many commonly used oxidants failed to afford the desired aromatization product, such as CuBr_2_ and Iodobenzene diacetate (Hisahiro et al., 2002, Dethe et al., 2015). Low yield of compound **13-1** was obtained when DDQ (Sadak et al., 2010) was utilized in high reaction temperature. Such inert reactivity in aromatization may probably be due to highly steric hindrance of carvone motif as shown in X-ray of product **6-5**. Interestingly, when iodine was utilized as stoichiometric in DMSO, only trace amount of the desired product was obtained; however, good conversion was observed when catalytic amount of iodide in 2.0 equivalent of DMSO was used (Wang et al., 2016, Liang et al., 2016). Finally, the desired product **13-1** could be obtained in moderate yield (Figure 7B, also see Table S2).

### Biological Evaluation

#### Anticancer Bioactivities Evaluation against A549 Cells

**Figure 8:**
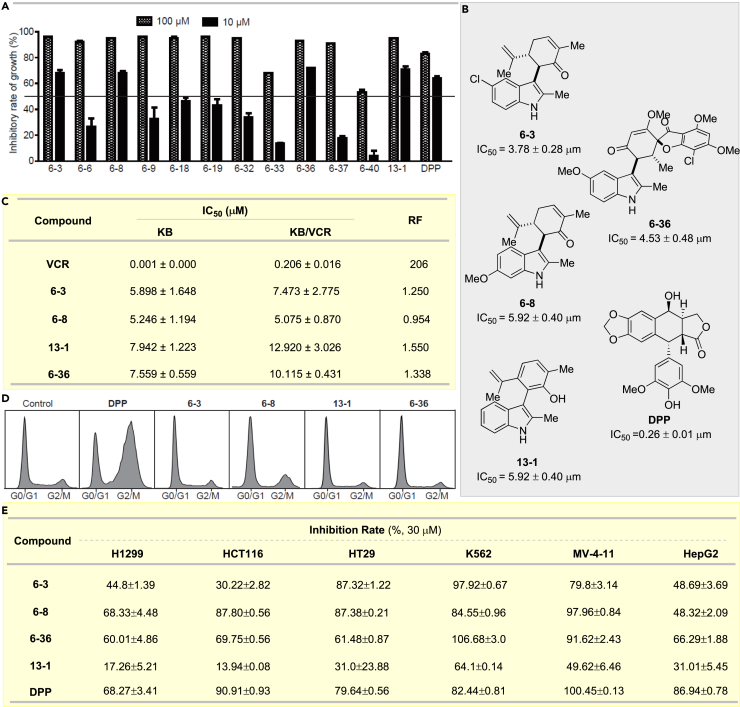
Bioactivity Evaluation (A) Cytotoxicity evaluation of selected products against A549 cells. (B) Structure and IC_50_ Value of selected molecules against A549 cells. (C) Resistance factors (RFs) of the compounds in KB-3-1 and KB/VCR cells; cells were treated with different concentrations of indicated compounds for 72 h and IC_50_ values were measured by sulforhodamine B assays. (D) The effects of the compounds on cell cycle progression; A549 cells were treated with indicated compounds (10 μM) for 24 h and cell cycle distribution was measured by flow cytometry. (E) Cytotoxicity evaluation of selected products against H1299, HCT116, HT29, K562, MV-4-11, and HepG2 cells for selected compounds. Also see in Supplemental Information.

Given that indoles have promising anticancer potential, we selected a dozen of our reaction products for the evaluation of cytotoxicity in human lung cancer cell line A549. To our delight, all the tested products exhibited significant anticancer bioactivities at 100 μM (Figure 8A). Then the compounds with an inhibitory rate higher than 50% at 10 μM were further investigated. As shown in Figure 8B, compounds **6-3**, **6-8**, **6-36**, and **13-1** showed marked cytotoxicities with IC_50_ (concentration to inhibit cell growth by 50%) in the low micromolar range, serving as a promising starting point for novel anticancer drug discovery. Notably, compound **13-1** showed comparable IC_50_ value as those of compound **6-3** and **6-8**, indicating that such observed cytotoxicity of compound **6-3** and **6-8** might not be from being Michael acceptor in carvone.

#### Multidrug Resistance Evaluation

Multidrug resistance (MDR) (Szakács et al., 2006, Housman et al., 2014) is a major impediment to effective chemotherapy of cancer. The cytotoxicities of compounds **6-3**, **6-8**, **13-1**, and **6-36** were evaluated in an MDR cell line. Resistance factor (RF), the ratio of the IC_50_ against MDR cells (KB/VCR) to that against the parental cells (KB-3-1), was calculated. The RF value for vincristine (VCR) was 206, which validated the MDR phenotype of KB/VCR cells. However, compounds **6-3**, **6-8**, **13-1**, and **6-36** maintained their potency against KB/VCR cells, suggesting that they could overcome multidrug resistance (Figure 8C).

#### Cell Cycle Progression Investigation

To further explore the mechanism underlying the anti-proliferative activities of compounds **6-3**, **6-8**, **13-1**, and **6-36**, their effects on cell cycle progression were examined. 4′-Demethylepipodophyllotoxin (DPP), a microtubule inhibitor, induced G2/M arrest in exponentially growing A549 cells. Although compounds **6-2**, **6-8**, **13-1**, and **6-36** share some structural similarities with DPP, they did not have significant impact on cell cycle progression, suggesting they exerted their anticancer activity through a mechanism different from that of DPP. Further studies on the exact mechanism are currently ongoing in our laboratory (Figure 8D).

#### Anticancer Bioactivities Evaluation against H1299, HCT116, HT29, K562, MV-4-11, and HepG2 Cells

Some of the products showed promising anticancer activity against A549 cells; however, whether they were effective in other cancer cell lines remains unknown. Thus, compound **6-3**, **6-8**, **6-36**, and **13-1** were investigated in several other cancer lines, including human lung cancer (H1299), colon cancer (HCT116, HT29), leukemia (K562, MV-4-11), and liver cancer (HepG2) cells. To our delight, the primary results showed promising results. For example, compound **6-8** and **6-36** showed good to excellent inhibition rate against all these cell lines except HepG2 for compound **6-8**. And all these compounds except **13-1** exhibited good to excellent inhibition effects in leukemia cell lines K562 and M-V-411. In contrast, compound **13-1** showed only a moderate inhibition rate in K562 cell. These results showed that our products could serve a good starting point for anticancer drug discovery. Further biological study of these compounds is under way in our laboratory (Figure 8E).

### Conclusion

In summary, we have developed an efficient approach to construct C2, C3-disubstituted indole by C2-substituent-promoted oxidative coupling of indole and enolate. Broad substrate scope and moderate to excellent yield were observed. The mechanism studies illustrated that the C2-substituent has dual effects to promote the reaction: stabilizing of the indole C2-radicals and decreasing the homocoupling of indole. Moreover, several products showed promising anticancer activities in several cell lines and can overcome multidrug resistance in KB/VCR cells in primary biological evaluation, which potentially serves as starting points for novel anticancer drug discovery.

## Methods

All methods can be found in the accompanying Transparent Methods supplemental file.

## Data and Code Availability

The data for the X-ray crystallographic structures of **6-5**, **6-5**, and **6-35** are available free of charge from the Cambridge Crystallographic Data Center under accession numbers CCDC: 1913053, 1914654, and 1913054, respectively.

## Supplemental Information

Document S1. Transparent Methods, Figures S1–S9, and Tables S1–S3
