# Supplementary material for: Anticancer Molecule Discovery via C2-Substituent Promoted Oxidative Coupling of Indole and Enolate
Source: iScience. 2019 Nov 16;22:214–28. doi: 10.1016/j.isci.2019.11.021 (PMC6909134; doi:10.1016/j.isci.2019.11.021)

ISCI, Volume *22*

Supplemental Information

Anticancer Molecule Discovery via C2-Substituent Promoted Oxidative Coupling of Indole and Enolate

Helin Lu, Guirong Zhu, Tiange Tang, Zhuang Ma, Qin Chen, and Zhilong Chen

I. Transparent Methods

I-1. General information

Glassware and stir bars were dried in an oven at 95 °C for at least 12h and then cooled in a desiccator cabinet over Drierite prior to use. Optimization and substrate screen were performed in 25 mL Schlenk flask. All other reactions were performed in round-bottom flasks sealed with rubber septa. Plastic syringes or pipets were used to transfer liquid reagents. Reactions were stirred magnetically using Teflon-coated, magnetic stir bars. Analytical thin-layer chromatography (TLC) was performed using glass plates pre-coated with 0.25 mm of 230–400 mesh silica gel impregnated with a fluorescent indicator (254 nm and 320 nm). TLC plates were visualized by exposure to ultraviolet light and/or exposure to $KMnO_4$ stain as well as phosphomolydic acid (PMA) and cerium molybdate stain. Organic solutions were concentrated under reduced pressure using a rotary evaporator. Flash-column chromatography was performed on silica gel (60 Å, standard grade).

Materials and Instrumentation. Nuclear magnetic resonance spectra were recorded at ambient temperature (unless otherwise stated) on Bruke 400 MHz spectrometers. All values for proton chemical shifts are reported in parts per million (δ) and are referenced to the residual protium in $CDCl_3$ (δ 7.26), CD_3OD (δ 3.31) and DMSO-d_6 (δ 2.50). All values for carbon chemical shifts are reported in parts per million (δ) and are referenced to the carbon resonances in $CDCl_3$ (δ 77.16), CD_3OD (49.00) and DMSO-d_6 (39.52). NMR data are represented as follows: chemical shift, multiplicity (s = singlet, d = doublet, t = triplet, q = quartet, quin = quintet, m = multiplet, br = broad), coupling constant (Hz), and integration. Infrared spectroscopic data was recorded by Bruker VERTEX 70 and reported in wavenumbers ($cm^{-}1$). High-resolution mass spectra were obtained using a liquid chromatography-electrospray ionization and Time-of-flight mass spectrometer by Bruker SolariX 7.0T.

I-2. Starting material synthesis

I-2-1. Preparation of C2-substituted indole

General procedure A Step 1: To a 0 °C solution of 6-methyl-1*H*-indole (15.2 mmol, 2.0 g, 1.0 equivalent) in THF (40 mL) was added NaH (60%, 916 mg, 22.8 mmol, 1.5 equivalent) under N_2 protection. The mixture was stirred for 1 h before benzenesulfonyl chloride (2.4 mL, 18.8 mmol, 1.25 equivalent) was added dropwise at the same temperature. The reaction mixture was then allowed to warm to room temperature and stirred for an additional 1 h. After completion of the reaction monitored by TLC, the reaction mixture was poured into a saturated NH_4Cl solution (100 mL). The aqueous phase was separated and extracted with ethyl acetate (50 mL × 2), the combined organic layers were washed with water (50 mL × 2) and brine (50 mL), dried over Na_2SO_4, and evaporated in *vacuo*. The residue was recrystallized (petroleum ether, EtOAc) to obtain the intermediate **14-1** (3.75 g, 91% yield) as a yellow crystal.

Step 2: To a -40 °C mixture of the indole intermediate **14-1** obtained from step 1(1408 mg, 5.2 mmol, 1.0 equivalent) in THF (35 mL) was added *n*-BuLi dropwise (3.9 mL, 1.6 M, 6.24 mmol, 1.2 equivalent) under N_2 protection. The mixture was stirred for an additional 1.5 h, then MeI (0.4 mL, 6.24 mmol, 1.2 equivalent) was added at the same temperature followed by warming to room temperature for an extra 3 h. After completion of the reaction confirmed by TLC, the mixture was poured into a saturated aqueous NH_4Cl solution (85 mL) and extracted with EtOAc (85 mL). The aqueous phase was separated and the organic layers were washed with water (40 mL × 2) and brine (40 mL), dried over Na_2SO_4, and evaporated in *vacuo*. The residue **15-1** was directly used for next step without purification.

Step 3: A mixture of **15-1** (1055.5 mg, 3.7 mmol, 1.0 equivalent), TBAF (1.0M in THF, 29.6 mL, 29.6 mmol, 8.0 equivalent), and THF (30 mL) was refluxed overnight. After completion of the reaction confirmed by TLC, the residual THF was evaporated in *vacuo*, and H_2O (15 mL) was added to the residue. The mixture was extracted with EtOAc (100 mL), The aqueous phase was separated and the organic layers were washed with water (15 mL × 2) and brine (15 mL), dried over Na_2SO_4, and evaporated in *vacuo*. The crude product was purified by column chromatography (silica gel, petroleum ether, ethyl acetate) to give the product **1** as a yellow solid (Figure S1).

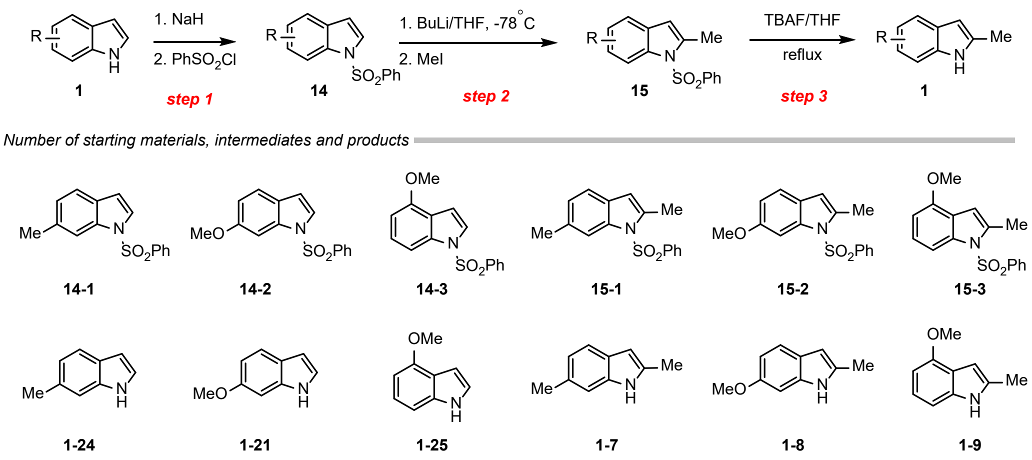

Figure S1. General procedure A for C2-substutited indole preparation, Related to Table 2

General procedure B To a 100 mL round bottom flask was successively charged with **1-21** (1471.8 mg, 10.0 mmol, 1.0 equivalent), norbornene (1881.5 mg, 20.0 mmol, 2.0 equivalent), K_2CO_3 (2.76 g, 20.0 mmol, 2.0 equivalent), $Pd(OAc)_2$ (224.9 mg, 10.0 mol %), **16-3** (2271.5 mg, 10.0 mmol, 1.0 equivalent) and DMA (50 mL, 0.5 M H_2O). The resulting solution was evacuated and backfilled with N_2 for 3 times followed by heating to 80 ℃ for 18 h. After completion of the reaction confirmed by TLC, the reaction mixture was diluted with DCM (100 mL) and filtered. The filtrate was washed with plenty of water several times to remove most of DMA. The aqueous phase was separated and the organic layers were washed with brine (30 mL x 2), dried over Na_2SO_4, and evaporated in *vacuo*. The residue was purified by flash column chromatography (silica gel, petroleum ether, EtOAc, DCM) to give the product **1-14** as a yellow solid (Figure S2).

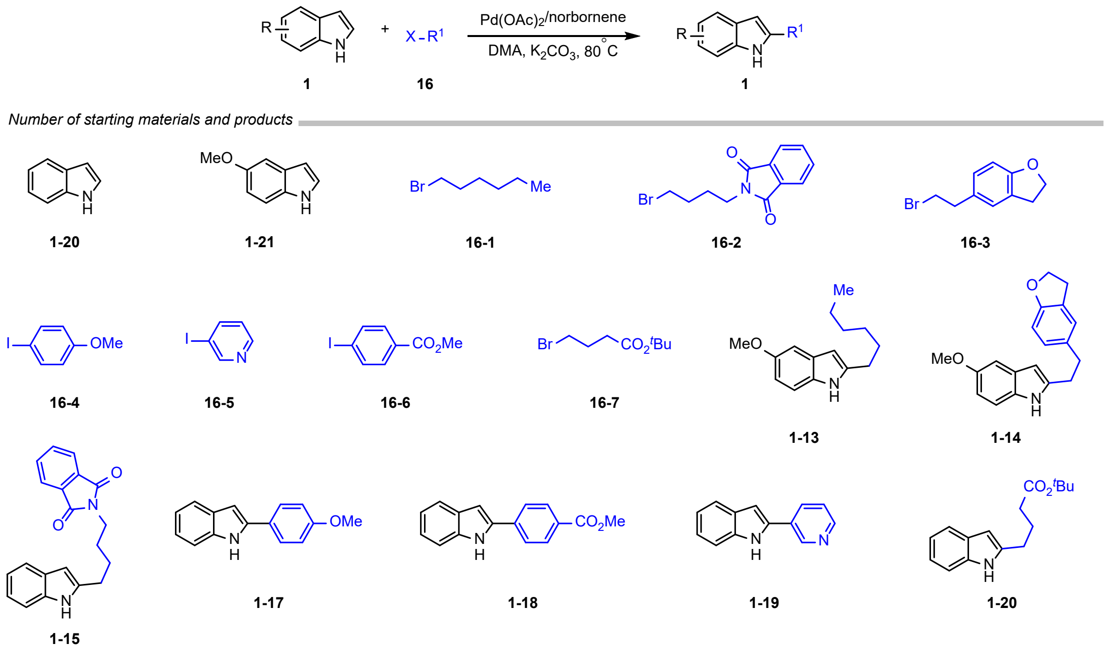

Figure S2. General procedure B for C2-substutited indole preparation, Related Table 2

I-2-2. Oxidative coupling of C2-substituted indoles and enolates

General procedure C To a 25 mL oven-dried Schlenk flask equipped with a magnetic stirring bar and a rubber stopper was charged with indole substrate **1** (2.0 mmol, 2.0 equivalent) and carbonyl compound **3** (1.0 mmol, 1.0 equivalent). The flask was evacuated and backfilled with N_2 for 3 times at least before adding THF (5 mL) by syringe. The resulting mixture was cooled to -78℃ and then LiHMDS (1.3 M in THF, 4.0 mmol, 4.0 equivalent) was added dropwise. The reaction mixture was stirred at -78 ℃ for 3h, followed by adding $FeCl_3$ (648 mg, 4.0 mmol, 4.0 equivalent, in 2 mL THF) for an additional 1 h at the same temperature, then H_2O (15 mL) and EtOAc (50 mL) was added, the aqueous phase was separated and the organic layers were washed with water (15 mL × 2) and brine (15 mL), dried over Na_2SO_4, and evaporated in *vacuo*. The crude product was purified by column chromatography (silica gel, petroleum ether, ethyl acetate) to give the desired product **6** (Figure S3).

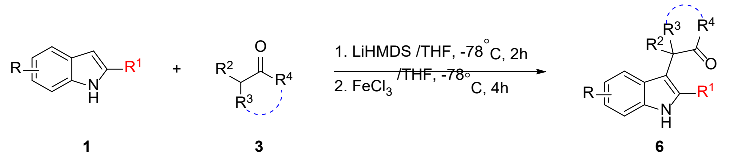

Figure S3. Oxidative coupling of C2-substituted indoles and enolates, Related to Table 1, Table 2 and Table 3

I-2-3. Center-to-axial chirality transfer reaction to synthesize C3 axially chiral indole

To the mixture of compound **6-1** (1.0 equivalent) and solvent was added oxidant. Next, the reaction was conducted according to following **Table 1** and monitored by TLC analysis, and then quenched by adding EtOAc and washed by H_2O, $Na_2S_2O_3$ (aq.), brine and dried by Na_2SO_4. The desired product **13-1** was isolated by flash chromatography (**Table S1**, entry 1: 0.3 mmol **6-1**; **13-1** was obtained as sticky oil, 16 mg, 19% yield; entry 9: **6-1** 27.9 mg, 0.1 mmol, 1.0 equivalent; **13-1** was obtained in 54% yield, 15.1 mg).

Table ST1. **Condition for aromization of compound 6-1, Related to Figure 7b**

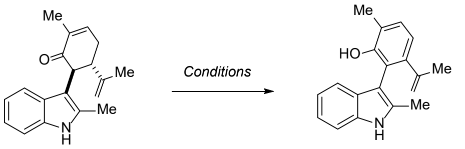

6-1 13-1

Entry	Condition	Conversion	Yield[a]	ee/es[b]
1	$CuBr_2$ (1.5 equiv.), MeCN, rt	<10%	N.R	--
2	AgOAc (1.5 equiv.), MeCN, rt.	<10%	N.R	--
3	NBS (1.5 equiv.), DCM, rt	100%	<5%	--
4	$IPh(OAc)_2$ (0.2~3 equiv.), DMSO, 60~100°C	<10%	N.R.	--
5	MnO_2 (3 equiv.), DMSO, 80°C	<10%	N.R.	--
6	$Hg(OAc)_2$ (2 equiv.), toluene, rt-50°C	<100%	messy	--
7	DDQ (1.5 equiv.), toluene, 100°C	<100%	19%	N.D
8	I_2 (2.0 equiv.)/ DMSO(sol)	<50%	<10%	--
9	I_2 (10 mol%)/DMSO (1.0 equiv)	<100%	54% (63% brsm)	rac/0%

I-2-4. Three-step synthesis of Indomethacin and its analogue

Step 1：Compound **6-45** (84.3 mg, 61% yield, 1 mmol scale from **1-6**) and **6-46** (102.2 mg, 50% yield, 0.5 mmol scale from **1-14**) were obtained according to **general procedure C**.

Step 2：To a 25 mL Schlenk vial equipped with a magnetic stirring bar was charged with indole substrate **6-45** (0.055 mmol, 1.0 equivalent) or **6-46** (0.158 mmol, 1.0 equivalent) The vial was evacuated and backfilled with N_2 for 3 times before adding THF (4 mL). The resulting mixture was cooled to -78℃ and *t*-BuOK (1.4 equivalent, in 2 mL THF) was added dropwise by syringe. After 1h, 4-Chlorobenzoyl chloride (1.3 equivalent, in 0.5 mL THF) was then added dropwise for overnight. After completion of the reaction confirmed by TLC, the mixture was poured into a saturated aqueous NH_4Cl solution (15 mL) and extracted with EtOAc (50 mL). The aqueous phase was separated and the organic layers were washed

with water (15 mL × 2) and brine (15 mL), dried over Na_2SO_4, and evaporated in *vacuo*. The residue was purified by flash column chromatography to give intermediate **12-1** (21.6 mg, 96% yield) or **12-3** (69.9 mg, 81% yield).

Step 3: intermediate **12-1**(0.052 mmol, 1.0 equivalent) or **12-3** (0.037 mmol, 1.0 equivalent) was dissolved in excess CF_3COOH (1.0 mL), and the resulting mixture was stirred for 2 h at room temperature. After completion of the reaction monitored by TLC, the residual CF_3COOH was evaporated in *vacuo*, and the crude compound was purified by flash column chromatography to give desired product **12-2** (18.4 mg, 99% yield) or **12-4** (17.5 mg, 96% yield) as a white solid (Figure S4).

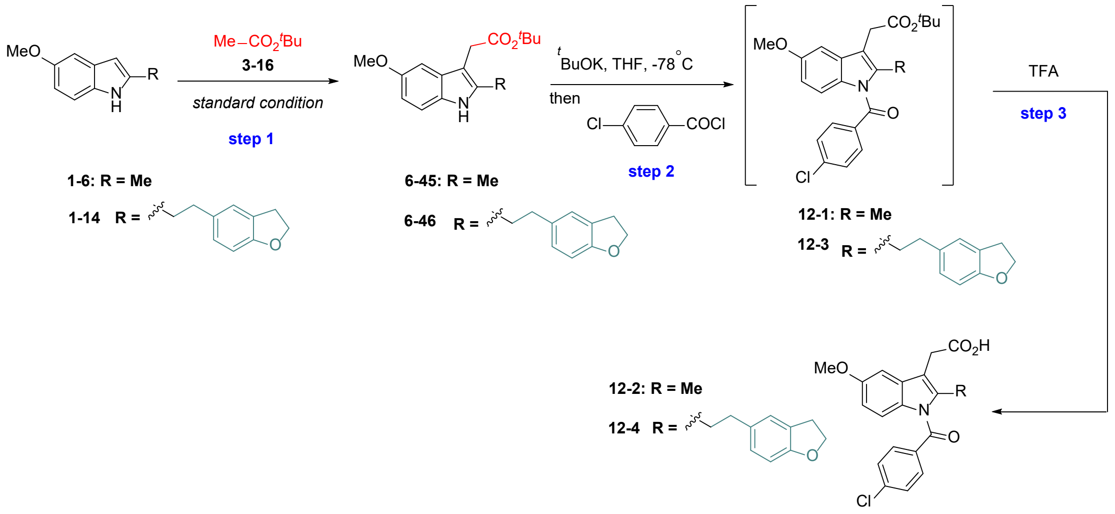

Figure S4. General procedure D for Indomethacin analogue preparation, Related to Figure 7a

I-2-5. Intramolecular Oxidative coupling of C2-substituted indoles and enolates

The intramolecular reaction was conducted similarly as General procedure C instead of utilizing 2.5 equivalent of LiHMDS and $FeCl_3$. However, the desired product from intramolecular oxidative coupling of indole and enolate, 6-49, was failed to be isolated. Instead, the homocoupling product **7-4** was isolated in 19% yield (Figure S5).

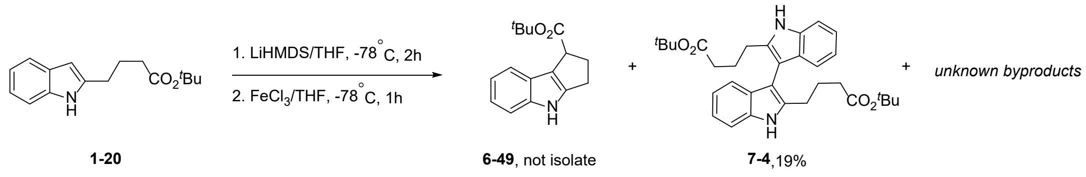

Figure S5. Fe-mediated intramolecular coupling of indole and enolate, Related Figure 6

I-3. Reaction Condition Optimization

Table ST2. Reaction Condition Optimization, Related Table 1

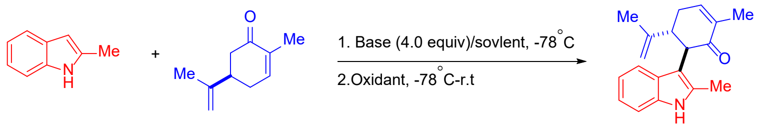

1-1 **3-1** **6-1**

Entry[a]	Base (equiv.)	Oxidant (equiv.)	Solvent	Condition	Conversion (%)	Yield (%)
1[b]	LDA (3.0)	Cu(2-ethylhexanoate)$_2$ (1.5)	THF	**A**	<100%	26-30
2[d]	LiHMDS (4.0)	$CuBr_2$ (3.0 in THF)	THF	**A**	<50%	N.R
3[e]	LiHMDS (4.0)	$CuBr_2$ (3.0)	THF	**A**	<100%	trace
4[f]	LiHMDS (4.0)	I_2 (3.0)	THF	**A**	<10%	trace
5	LiHMDS (4.0)	Fe(acac)$_3$ (3.0)	THF	**A**	<100%	20
6	LiHMDS (4.0)	$FeCl_3$ (3.0)	THF	**A**	<100%	31
7	LiHMDS (4.0)	$CuCl_2$ (3.0)	THF	**A**	<100%	30
8	LiHMDS (4.0)	$FeCl_3$ (4.0)	THF	**B**	100%	57
9	LiHMDS (2.0)	$FeCl_3$ (4.0)	THF	**B**	<100%	20
10	LiHMDS (5.0)	$FeCl_3$ (4.0)	THF	**B**	<100%	52
11	LiHMDS (4.0)	$FeCl_3$ (4.0)	DMF	**B**	<10%	trace
12	LiHMDS (4.0)	I_2 (3.0)	THF	**B**	<10%	trace
13	LDA (2.0)	$FeCl_3$ (4.0)	THF	**B**	<100%	19
14	LiHMDS (4.0)	$FeCl_3$ (4.0)	toluene	**B**	100%	41
15	*LiHMDS (4.0)*	*$FeCl_3$ (4.0)*	*THF*	**C**	*100%*	*89-93*
16	LDA (4.0)	$FeCl_3$ (4.0)	THF	**C**	100%	41

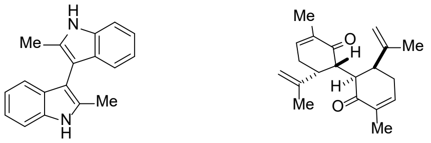

7-1 **8-1**

a. all the reactions were conducted with compound **1-1** (2.0 mmol, 2.0 equivalent) and (*R*)-carvone (**3-1**, 1.0 mmol, 1.0 equivalent), isolated yield; dr was determined by H-NMR of the isolated product or the yield of two isomers; b. Phil Barans best condition in oxidative coupling of indole and carvone (Richter et al., 2007); c. N.R = No Reaction; d. $CuBr_2$ cannot be able to be solved in THF, thus it was difficult to adding $CuBr_2$ in THF solution; e. dimmerization of compound **1-1** and **3-1** was observed, isolated compound **7-1** and **8-1**;f. Ma's reaction condition for intramolecular oxidative coupling of enolate and indole (Zuo et al., 2010);

Condition A: Baran's reaction condition, after adding LiHMDS or LDA at -78℃ for 0.5h followed by adding

oxidant in one portion as solid, the reaction was warmed to room temperature for 15 min.

Condition B: After adding LiHMDS or LDA at -78℃ for 3 h followed by adding oxidant in one portion as solid, the reaction was warmed to room temperature.

Condition C: After adding LiHMDS or LDA at -78℃ for 3 h followed by adding $FeCl_3$ in anhydrous THF, the reaction was stirred at -78℃ for 1h.

I-4. Mechanism study

I-4-1. Competing experiments

To the mixture of indole **1-16** (193 mg, 1.0 mmol, 1.0 equivalent), **1-20** (117 mg, 1.0 mmol, 1.0 equivalent), and (*R*)-carvone **3-1** (150 mg, 1.0 mmol, 1.0 equivalent) in THF (5 mL) at -78℃ under N_2 balloon was slowly added LiHMDS (1.3 M in THF, 4.0 mmol, 4.0 equivalent). Next, the reaction was stirred at -78℃ for 2h, followed by adding $FeCl_3$ (648 mg, 4.0 mmol, 4.0 equivalent, in 2 mL THF). After stirring for additional 5h at -78℃, the reaction was quenched by adding around 0.5 mL H_2O and diluted with around 100 mL EtOAc. The organic lawyer was then washed by H_2O, brine and dried by Na_2SO_4. The desired product **6** was isolated via flash chromatography. The distribution of product **6-16** and **6-42** was determined by both H-NMR of crude product and isolated yields (Figure S6).

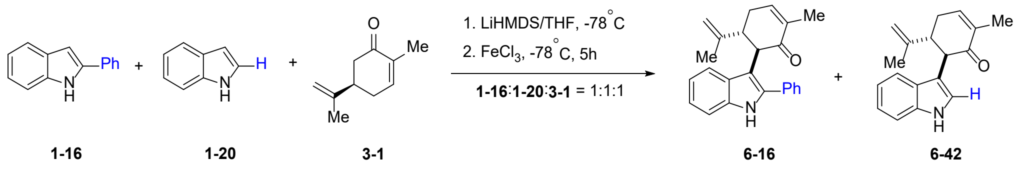

Figure S6. Competing reactions between 1-16 and 1-20 in coupling with (*R*)-carvone (3-1), Related to Figure 3b

I-4-2. Background reactions: homocoupling of C2-substituted indole

To the mixture of indole **1** (2.0 mmol, 1.0 equivalent) in THF (5 mL) at -78℃ under N_2 balloon was slowly added LiHMDS (1.3 M in THF, 4.0 mmol, 4.0 equivalent). Next, the reaction was stirred at -78℃ for 3h, followed by adding $FeCl_3$ (648 mg, 4.0 mmol, 4.0 equivalent, in 2 mL THF). After stirring for additional 5h at -78℃, the reaction was quenched by adding around 0.5 mL H_2O and diluted with around 100 mL EtOAc. The organic lawyer was then washed by H_2O, brine and dried by Na_2SO_4. The 3,3'-bisindole product **7** was isolated via flash chromatography (Figure S7).

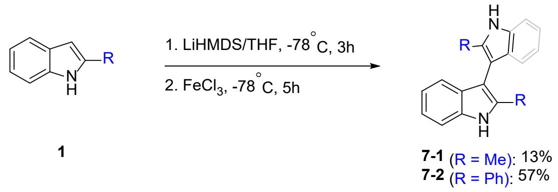

Figure S7. Homocoupling of C2-substituted indole, Related to Figure 3c

I-4-3. Comparison our and Baran's approach in background reaction of indole tetramerization

To the mixture of indole **1-20** (2.0 mmol, 1.0 equivalent) in THF (5 mL) at -78℃ under N_2 balloon was slowly added LiHMDS (1.3 M in THF, 4.0 mmol, 4.0 equivalent). Next, the reaction was stirred at -78℃ for 3h, followed by adding $FeCl_3$ (648 mg, 4.0 mmol, 4.0 equivalent, in 2 mL THF). After stirring for additional 1h at -78℃, the reaction was quenched by adding around 0.5 mL H_2O and diluted with around 100 mL EtOAc. The organic lawyer was then washed by H_2O, brine and dried by Na_2SO_4. The tetramer of indole **10** (81.2 mg, 35% yield) was isolated as yellow solid *via* flash chromatography. The control experiment of Baran's condition (3.0 equivalent of LDA, 1.5 equivalent of Cu(2-ethylhexanoate)$_2$, -78℃ to rt) was conducted according to literature, and compound **10** was obtained in 42% yield (Figure S8).

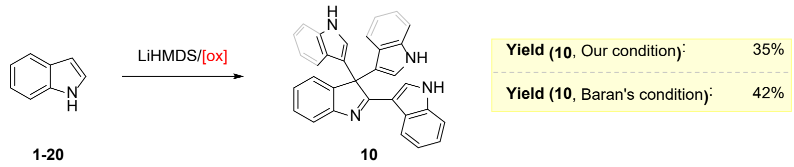

Figure S8. Background reaction of indole tetramerization, Related to Figure 3c

I-4-4. Indole C3-radical intermediate exploring reaction

To the solution of compound **1-14** (52.5 mg, 0.18 mmol, 1.0 equivalent) in THF (4 mL) was added LiHMDS (1.3 M, 0.28 mL, 0.36 mmol, 2 equivalent) and $FeCl_3$ (60 mg, 0.36 mmol, 2.0 equivalent) as **general procedure C**. After completion of the reaction, the products were isolated. And only compound **7-3** (22.2 mg, 42% yield) was isolated as brown solid (Figure S9).

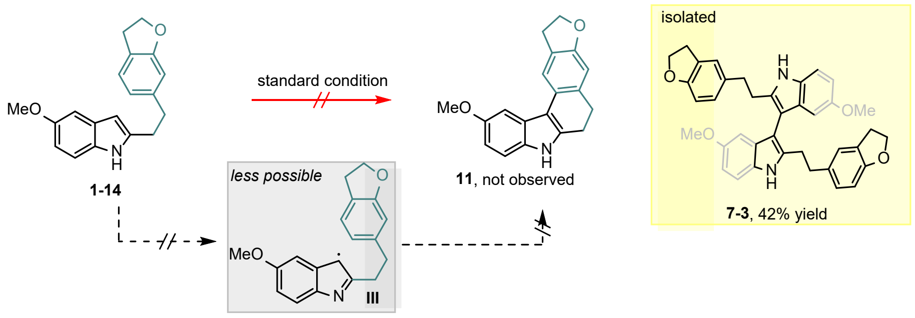

Figure S9. **Indole C3-radical trapping experiments, Related to Figure 3d**

I-4-5. Transition state investigation experiments

These experiments were conducted according to **general procedure C** by switching either the base or Fe-oxidant based as summarized in following **Table ST3** to figure the chelating metal (Fe or Li) in transition state**.** The yields of compound **6-1** in each reaction was isolated after the reaction in the same procedure as documented in **general procedure C**. For entry 6, the homocoupling of **3-1** was isolated in 17% yield (product **8-1**, with a little bit of 2-methylindole **1-1** in H-NMR spectra, as shown in following).

Table ST3. **Metal-chelating investigation, Related to Figure 3e**

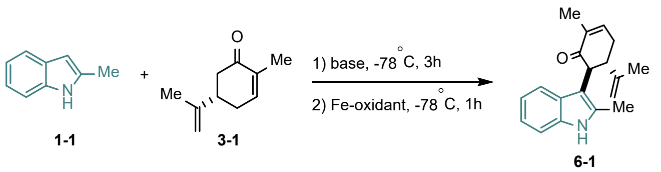

Entry[a]	base (4.0 equiv.)	oxidant (4.0 equiv.)	yield (%)
1	LiHMDS	$FeCl_3$	93% (260.1 mg)
2	NaHMDS	$FeCl_3$	89% (247.5 mg)
3	KHMDS	$FeCl_3$	95% (263.8 mg)
4	LDA	$FeCl_3$	41% (114.2 mg)
5	LiHMDS	Fe(acac)3	20% (56.1 mg)
6	LiHMDS	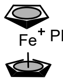 Fe^+ PF_6^-	trace

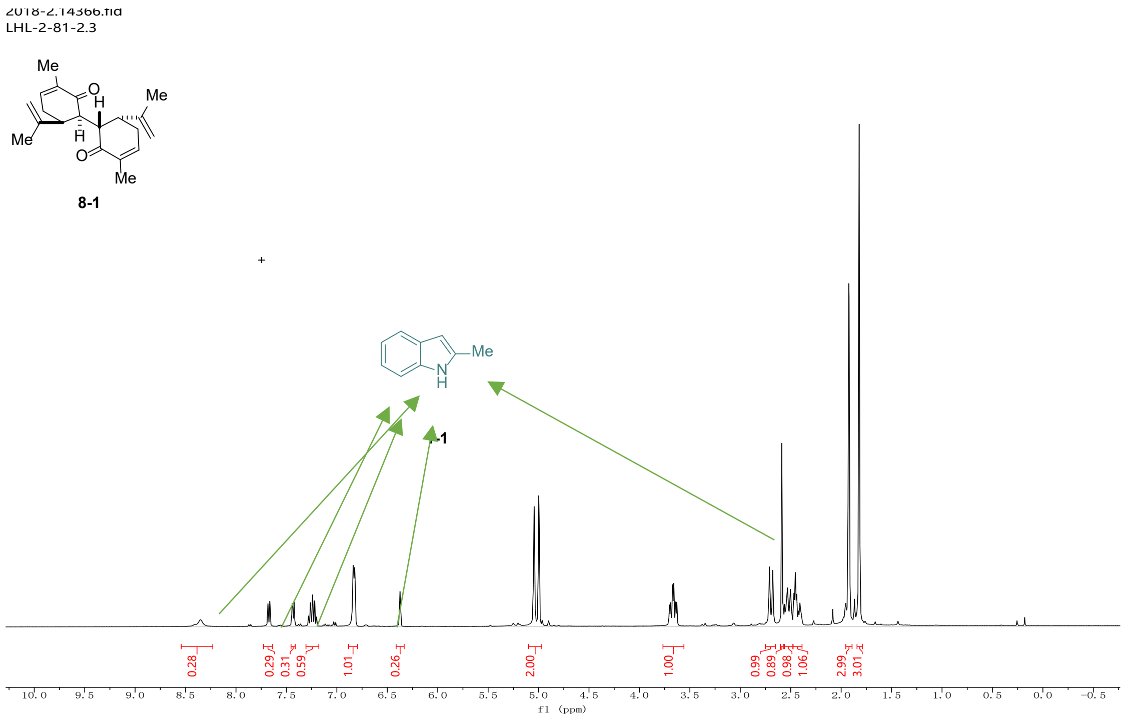

I-5. Cytotoxicity Study

Cell culture Human non-small cell lung cancer A549 and H1299 cells, Human colon cancer HCT116 and HT29, Human leukemia K562 and MV-4-11 cells, and Human liver cancer HepG2 cells were purchased from ATCC (Manassas, VA). Human cervix carcinoma KB-3-1 and KB/VCR cells were gifts from Dr. Linghua Meng (Shanghai Institute of Materia Medica, Shanghai, China). A549, H1299, K562, MV-4-11 and KB-3-1 cells were cultured in RPMI 1640 medium, containing 10% FBS, 100 U/mL penicillin and 100 μg/mL streptomycin at 37 °C in a humidified atmosphere of 5% CO_2. HCT116 and HT29 cells were cultured in McCoy' 5a, containing 10% FBS, 100 U/mL penicillin and 100 μg/mL streptomycin at 37 °C in a humidified atmosphere of 5% CO_2. HepG2 cells were cultured in DMEM, containing 10% FBS, 5 mM Hepes, 2 mM Glutamine, 100 U/mL penicillin and 100 μg/mL streptomycin and stored at 4 °C, and then cultured in 37°C in a humidified atmosphere of 5% CO_2 before being treated with tested compounds. The multidrug resistant subline KB/VCR were maintained in RPMI 1640 medium supplemented with 0.1 μg/mL vincristine.

Cytotoxicity Study of A549 cells The cells were seeded in 96-well plates at a density of 2000 cells/well. After cultured for overnight, the cells were treated with different concentrations of tested compounds for 72 h. Then the cells were fixed with 10% trichloroacetic acid followed by staining with 0.1% sulforhodamine B (SRB). After washed with 1% acetic acid to remove unbound dye, the plates were left to dry at room temperature and 100 μl TRIS base (10 mM) was added to solubilize the protein-bound SRB. The absorbance at 540 nm was measured with a microplate reader (SpectraMax M2, Molecular Devices). Inhibitory rate of growth was calculated by the following formula: inhibitory rate (%) = (A_{540} of vehicle control − A_{540} of treated cells) / (A_{540} of vehicle control − A_{540} of blank control)*100. The IC_{50} values were calculated by using GraphPad Prism 5 software. Experiments were performed in triplicate.

Cell cycle analysis Exponentially growing A549 cells were incubated with tested compounds or DMSO for 24 h. After fixed with 70% ice-cold ethanol, the cells were incubated in a DNA staining solution containing 50 μg/mL propidium iodide and 0.5 mg/mL RNase for 30 minutes. The DNA content was

measured with a FACSCalibur flow cytometer (Becton Dickinson, Mountain View, CA) and cell cycle phase distribution was analyzed by using FlowJo V10 software.

Cytotoxicity Study of H1299, HCT116, HT29, K562, MV-4-11 and HepG2 cells The cells were seeded in 96-well plates at certain density of cells/well (2000 cells/well for H1299, HCT116 and K562 cells; 3000 cells/well for HT29 cells; 5000 cells/well for HepG2 cells; 10000 cells/well for MV-4-11 cells). After cultured for overnight, the cells were treated with 20 μL of tested compounds (30 μM) for 72 h. And then 10 μL of CCK-8 solution to each well, followed by incubating the plate for 4h at 37 °C in a humidified atmosphere of 5% CO_2. The absorbance at 450 nm was measured with a microplate reader (Nivo). Inhibitory rate of growth was calculated by the following formula: inhibitory rate (%) = (A_{450} of vehicle control – A_{450} of treated cells) / (A_{450} of vehicle control – A_{450} of blank control)*100.

II. Spectra of starting material and products

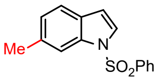

14-1

6-methyl-1-(phenylsulfonyl)-1*H*-indole (14-1) (Chen et al., 2011) was synthesized according to **general procedure A** and obtained as a white solid (15.2 mmol scale, 3.75g, 91% yield).

^{1}H NMR (400 MHz, CDCl3) δ 7.87 (d, *J* = 7.9 Hz, 2H), 7.81 (s, 1H), 7.53 (t, *J* = 7.4 Hz, 1H), 7.49 (d, *J* = 3.7 Hz, 1H), 7.47 – 7.37 (m, 3H), 7.06 (d, *J* = 8.0 Hz, 1H), 6.61 (d, *J* = 3.7 Hz, 1H), 2.48 (s, 3H).

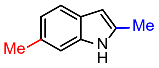

1-7

2,6-dimethyl-1*H*-indole (1-7) (Miyata et al., 2002) was synthesized according to **general procedure A** and obtained as a yellow solid (3.7 mmol scale, 406.2 mg, 76% yield).

^{1}H NMR (400 MHz, CDCl3) δ 7.70 (s, 1H), 7.39 (d, *J* = 8.0 Hz, 1H), 7.07 (s, 1H), 6.91 (d, 1H), 6.16 (s, 1H), 2.45 (s, 3H), 2.42 (s, 3H).

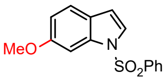

14-2

6-methoxy-1-(phenylsulfonyl)-1*H*-indole (14-2) (Trabbic et al., 2016) was synthesized according to **general procedure A** and obtained as a white solid (13.6 mmol scale, 3.60 g, 92% yield).

^{1}H NMR (400 MHz, CDCl3) δ 7.86 (dd, *J* = 8.4, 1.3 Hz, 2H), 7.62 – 7.50 (m, 2H), 7.49 – 7.35 (m, 4H), 6.87 (dt, *J* = 8.6, 1.5 Hz, 1H), 6.58 (dd, *J* = 3.7, 0.9 Hz, 1H), 3.88 (s, 3H).

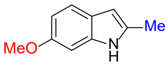

1-8

6-methoxy-2-methyl-1*H*-indole (**1-8**) (Trabbic et al., 2016) was synthesized according to **general procedure A** and obtained as a yellow solid (3 mmol scale, 459.4 mg, 95 % yield).

^{1}H NMR (400 MHz, $CDCl_3$) δ 7.73 (s, 1H), 7.37 (d, *J* = 8.6 Hz, 1H), 6.80 (d, *J* = 2.2 Hz, 1H), 6.74 (dd, *J* = 8.5, 2.2 Hz, 1H), 6.13 (s, 1H), 3.83 (s, 3H), 2.40 (s, 3H).

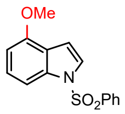

14-3

4-methoxy-1-(phenylsulfonyl)-1*H*-indole (**14-3**) (Stempel et al., 2018) was synthesized according to **general procedure A** and obtained as a white solid (13.6 mmol scale, 3.52 g, 90% yield).

^{1}H NMR (400 MHz, CDCl3) δ 7.87 (d, *J* = 7.7 Hz, 2H), 7.60 (d, *J* = 8.3 Hz, 1H), 7.52 (t, *J* = 7.4 Hz, 1H), 7.47 (d, *J* = 3.7 Hz, 1H), 7.43 (t, *J* = 7.6 Hz, 2H), 7.22 (d, *J* = 8.2 Hz, 1H), 6.78 (d, *J* = 3.7 Hz, 1H), 6.65 (d, *J* = 8.0 Hz, 1H), 3.89 (s, 3H).

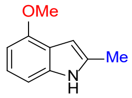

1-9

4-methoxy-2-methyl-1*H*-indole (**1-9**) (Trabbic et al., 2016) was synthesized according to **general procedure A** and obtained as a yellow solid (4 mmol scale, 481.1 mg, 75% yield).

^{1}H NMR (400 MHz, $CDCl_3$) δ 7.85 (s, 1H), 7.03 (t, *J* = 7.9 Hz, 1H), 6.93 (d, *J* = 8.1 Hz, 1H), 6.51 (d, *J* = 7.8 Hz, 1H), 6.32 (s, 1H), 3.94 (s, 3H), 2.43 (s, 3H).

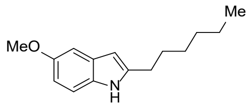

1-13

2-hexyl-5-methoxy-1*H*-indole (**1-13**) (Yamagishi et al., 2012) was synthesized according to **general procedure B** and obtained as a brown oil (20 mmol scale, 1090.9 mg, 24 % yield).

^{1}H NMR (400 MHz, $CDCl_3$) δ 7.77 (s, 1H), 7.17 (d, *J* = 8.7 Hz, 1H), 7.04 (d, *J* = 2.5 Hz, 1H), 6.79 (dd, *J* = 8.7, 2.4 Hz, 1H), 6.18 (s, 1H), 3.86 (s, 3H), 2.72 (t, *J* = 7.7 Hz, 2H), 1.71 (p, *J* = 7.5 Hz, 2H), 1.44 – 1.37 (m, 2H), 1.36 – 1.29 (m, 4H), 0.92 (t, *J* = 6.9, 5.8, 3.6 Hz, 3H).

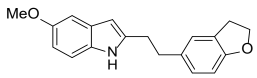

1-14

2-(2-(2,3-dihydrobenzofuran-5-yl)ethyl)-5-methoxy-1*H*-indole (**1-14**) was synthesized according to

general procedure B and obtained as a yellow solid (10 mmol scale, 732.3 mg, 28% yield).

^{1}H NMR (400 MHz, $CDCl_3$) δ 7.78 (s, 1H), 7.15 (d, *J* = 8.7 Hz, 1H), 7.06 (d, *J* = 2.5 Hz, 2H), 6.96 (dd, *J* = 8.2, 1.9 Hz, 1H), 6.82 (dt, *J* = 8.9, 2.5 Hz, 1H), 6.76 (dd, *J* = 8.1, 1.8 Hz, 1H), 6.23 (s, 1H), 4.58 (t, *J* = 8.7 Hz, 2H), 3.87 (s, 3H), 3.19 (t, *J* = 8.6 Hz, 2H), 3.05 – 2.91 (m, *J* = 4.3 Hz, 4H).

^{13}C NMR (101 MHz, $CDCl_3$) δ 158.5, 154.1, 140.2, 133.3, 131.0, 129.2, 127.8, 127.3, 125.0, 111.1, 110.9, 109.1, 102.1, 99.6, 71.3, 55.9, 35.1, 30.7, 29.8.

HRMS-API (m/z): calcd. for $C_{19}H_{18}NO_2$ [M - H^+] 292.1332, found 292.1339

FTIR (film, cm^{-1}): 3338, 1621, 1589, 1489, 1440, 1202, 1167 cm^{-1}

M.p.: 109 - 110 ℃

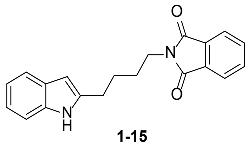

1-15

2-(4-(1*H*-indol-2-yl)butyl)isoindoline-1,3-dione (**1-15**) was synthesized according to **general procedure B** and obtained as a light yellow solid (10 mmol scale, 707.5 mg, 22% yield).

^{1}H NMR (400 MHz, $CDCl_3$) δ 8.11 (s, 1H), 7.85 (dd, *J* = 5.5, 3.1 Hz, 2H), 7.71 (dd, *J* = 5.4, 3.1 Hz, 2H), 7.50 (d, *J* = 7.7 Hz, 1H), 7.31 (d, *J* = 7.9 Hz, 1H), 7.14 – 7.01 (m, 2H), 6.23 (s, 1H), 3.75 (t, *J* = 6.6 Hz, 2H), 2.83 (t, *J* = 7.0 Hz, 2H), 1.77 (hept, *J* = 5.2, 4.8 Hz, 4H).

^{13}C NMR (101 MHz, $CDCl_3$) δ 168.7(x 2C), 139.2, 136.1, 134.1(x 2C), 132.2(x 2C), 128.9, 123.4(x 2C), 121.2, 119.9, 119.7, 110.5, 99.9, 37.4, 28.1, 27.6, 26.6.

HRMS-API (m/z): calcd. for $C_{20}H_{19}N_2O_2$ [M + H^+] 319.1441, found 319.1441

FTIR (film, cm^{-1}): 3381, 1763, 1706, 1401, 1040, 722 cm^{-1}

M.p.: 123 – 124 ℃

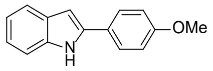

1-17

2-(4-methoxyphenyl)-1*H*-indole (**1-17**) (Yang et al., 2008) was synthesized according to **general procedure B** and obtained as a yellow solid (10 mmol scale, 1155.8 mg, 52% yield).

^{1}H NMR (400 MHz, DMSO-d_6) δ 11.41 (s, 1H), 7.83 – 7.76 (m, 2H), 7.50 (dd, 1H), 7.38 (dd, *J* = 8.0, 1.0

Hz, 1H), 7.05 (td, J = 7.5, 1.6 Hz, 3H), 6.98 (ddd, J = 8.0, 7.0, 1.1 Hz, 1H), 6.76 (dd, J = 2.2, 0.9 Hz, 1H), 3.81 (s, 3H).

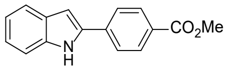

1-18

Methyl 4-(1*H*-indol-2-yl)benzoate (**1-18**) (Wetzel et al., 2016) was synthesized according to **general procedure B** and obtained as a yellow solid (10 mmol scale, 1331.4 mg, 53% yield).

^{1}H NMR (400 MHz, DMSO-d_6) δ 11.75 (s, 1H), 8.01 (q, J = 8.3 Hz, 4H), 7.57 (d, J = 7.9 Hz, 1H), 7.47 (d, J = 8.2 Hz, 1H), 7.17 (t, J = 7.6 Hz, 1H), 7.08 – 7.00 (m, 2H), 3.85 (s, 3H).

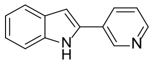

1-19

2-(pyridin-3-yl)-1*H*-indole (**1-19**) (Molander et al., 2009) was synthesized according to **general procedure B** and obtained as a yellow solid (10 mmol scale, 926.3 mg, 48% yield).

^{1}H NMR (400 MHz, $CDCl_3$ + DMSO-d_6) δ 8.98 (d, J = 2.3 Hz, 1H), 8.93 (s, 1H), 8.55 (dd, J = 4.8, 1.6 Hz, 1H), 7.96 (dt, J = 8.1, 2.0 Hz, 1H), 7.66 (d, J = 7.9 Hz, 1H), 7.42 (d, J = 8.1 Hz, 1H), 7.36 (dd, J = 8.0, 4.7 Hz, 1H), 7.22 (t, 1H), 7.15 (t, J = 7.4 Hz, 1H), 6.90 (d, J = 2.2 Hz, 1H).

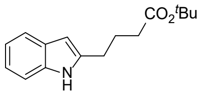

1-20

tert*-butyl** 4-(1H***-indol-2-yl)butanoate (**1-20**) was synthesized according to **general procedure B** and obtained as a white solid (5 mmol scale, 612.6 mg, 47% yield).

^{1}H NMR (400 MHz, $CDCl_3$) δ 8.16 (s, 1H), 7.54 (dd, J = 7.6, 1.4 Hz, 1H), 7.34 – 7.28 (m, 1H), 7.10 (dtd, J = 20.6, 7.1, 1.3 Hz, 2H), 6.26 (dd, J = 2.3, 1.0 Hz, 1H), 2.80 (t, J = 7.3 Hz, 2H), 2.31 (t, J = 7.3 Hz, 2H), 2.00 (p, J = 7.3 Hz, 2H), 1.47 (s, 9H).

^{13}C NMR (101 MHz, $CDCl_3$) δ 173.1, 138.8, 136.1, 128.9, 121.2, 119.9, 119.7, 110.5, 100.1, 80.6, 34.8, 28.3(x 3C), 27.4, 25.0.

HRMS-API (m/z): calcd for $C_{16}H_{22}NO_2^+$ [M + H$^+$] 260.1645, found 260.1644

FTIR (film, cm^{-1}): 3369, 1711, 1367, 1154, 779 cm^{-1}

M.p.: 92 - 93 ℃

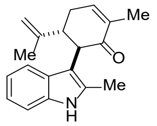

6-1

(5*R*,6*R*)-2-methyl-6-(2-methyl-1*H*-indol-3-yl)-5-(prop-1-en-2-yl)cyclohex-2-en-1-one (**6-1**) was synthesized according to **general procedure C** and obtained as a white solid (1 mmol scale, 259.1 mg, 93% yield).

^{1}H NMR (400 MHz, $CDCl_3$) δ 8.23 (s, 1H), 7.34 – 7.27 (m, 1H), 7.07 – 6.97 (m, 3H), 6.89 (d, *J* = 6.0 Hz, 1H), 4.59 (d, *J* = 1.9 Hz, 1H), 4.51 (d, *J* = 2.0 Hz, 1H), 3.80 (d, *J* = 13.0 Hz, 1H), 3.45 – 3.33 (m, 1H), 2.65 (ddt, *J* = 18.6, 11.3, 2.7 Hz, 1H), 2.45 (dt, *J* = 18.4, 5.4 Hz, 1H), 2.00 (s, 3H), 1.94 (s, 3H), 1.53 (s, 3H).

^{13}C NMR (101 MHz, $CDCl_3$) δ 200.1, 145.8, 144.4, 135.6, 135.5, 133.0, 127.9, 120.2, 118.6, 118.2, 112.3, 110.8, 108.0, 49.5, 48.5, 32.0, 19.3, 16.4, 11.9.

HRMS-ESI (m/z): calcd for $C_{19}H_{22}NO$ [M + H$^+$] 280.1695, found 280.1699

FTIR (film, cm^{-1}): 3329, 2916, 1670, 1459, 1431, 1366, 902, 742 cm^{-1}

M.p.: 126 – 127 ℃

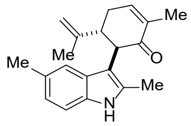

6-2

(5*R*,6*R*)-6-(2,5-dimethyl-1*H*-indol-3-yl)-2-methyl-5-(prop-1-en-2-yl)cyclohex-2-en-1-one (**6-2**) was synthesized according to **general procedure C** and obtained as a white solid (0.5 mmol scale, 140.4 mg, 96% yield).

^{1}H NMR (400 MHz, $CDCl_3$) δ 7.90 (s, 1H), 7.06 – 6.97 (m, 2H), 6.86 (td, *J* = 7.9, 1.8 Hz, 2H), 4.59 (d, *J* = 1.9 Hz, 1H), 4.52 (t, *J* = 1.7 Hz, 1H), 3.75 (d, *J* = 13.0 Hz, 1H), 3.37 (ddd, *J* = 12.9, 11.2, 4.4 Hz, 1H), 2.64 (ddt, *J* = 18.6, 11.3, 2.6 Hz, 1H), 2.46 (td, *J* = 5.1, 4.6, 2.8 Hz, 1H), 2.40 (s, 3H), 2.09 (s, 3H), 1.92 (s, 3H), 1.53 (s, 3H).

^{13}C NMR (101 MHz, $CDCl_3$) δ 199.9, 146.1, 144.1, 135.7, 133.9, 132.8, 128.3, 127.8, 122.0, 118.3, 112.3, 110.3, 108.0, 49.6, 48.5, 32.1, 21.8, 19.4, 16.5, 12.3.

HRMS-ESI (m/z): calcd for $C_{20}H_{24}NO$ [M + H+] 294.1852, found 294.1854

FTIR (film, cm^{-1}): 3355, 2917, 1670, 1446, 1435, 1366, 1307, 1227, 899, 790 cm^{-1}

M.p.: 160 – 161 ℃

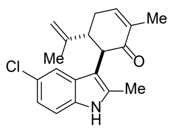

6-3

(5*R*,6*R*)-6-(5-chloro-2-methyl-1*H*-indol-3-yl)-2-methyl-5-(prop-1-en-2-yl)cyclohex-2-en-1-one (6-3) was synthesized according to **general procedure C** and obtained as a light yellow solid (1 mmol scale, 244.2 mg, 78% yield).

^{1}H NMR (400 MHz, $CDCl_3$) δ 8.05 (s, 1H), 7.19 (d, *J* = 1.9 Hz, 1H), 7.02 – 6.93 (m, 2H), 6.88 (d, *J* = 6.1 Hz, 1H), 4.56 (s, 1H), 4.50 (s, 1H), 3.70 (d, *J* = 13.2 Hz, 1H), 3.30 (ddd, *J* = 13.3, 11.3, 4.4 Hz, 1H), 2.62 (ddq, *J* = 19.1, 10.9, 2.6 Hz, 1H), 2.44 (dt, *J* = 18.6, 5.4 Hz, 1H), 2.10 (s, 3H), 1.89 (s, 3H), 1.49 (s, 3H).

^{13}C NMR (101 MHz, $CDCl_3$) δ 199.6, 145.7, 144.5, 135.7, 134.5, 134.0, 129.1, 124.6, 120.8, 118.0, 112.7, 111.6, 108.4, 49.5, 48.6, 32.2, 19.3, 16.5, 12.4.

HRMS-ESI (m/z): calcd for $C_{19}H_{21}ClNO$ [M + H+] 314.1306, found 314.1308

FTIR (film, cm^{-1}): 3336, 2920, 1665, 1458, 1370, 1310, 889, 882, 854, 791 cm^{-1}

M.p.: 149 – 150 ℃

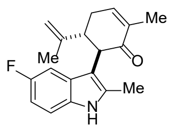

6-4

(5*R*,6*R*)-6-(5-fluoro-2-methyl-1*H*-indol-3-yl)-2-methyl-5-(prop-1-en-2-yl)cyclohex-2-en-1-one (6-4) was synthesized according to **general procedure C** and obtained as a light brown solid (0.5 mmol scale, 99.1 mg, 67% yield).

^{1}H NMR (400 MHz, $CDCl_3$) δ 8.07 (s, 1H), 6.96 (dd, *J* = 8.7, 4.5 Hz, 1H), 6.89 (dd, *J* = 10.1, 2.6 Hz, 2H), 6.74 (td, *J* = 9.1, 2.5 Hz, 1H), 4.57 (s, 1H), 4.51 (d, *J* = 2.2 Hz, 1H), 3.70 (d, *J* = 13.1 Hz, 1H), 3.36 – 3.24 (m, 1H), 2.63 (ddq, *J* = 19.1, 10.9, 2.6 Hz, 1H), 2.44 (dt, *J* = 18.5, 5.4 Hz, 1H), 2.07 (s, 3H), 1.90 (s, 3H), 1.50 (s, 3H).

^{13}C NMR (101 MHz, $CDCl_3$) δ 199.8, 157.6 (d, $J_{C\text{-}F}$ = 232.5 Hz, 1C), 145.8, 144.5, 135.7, 134.9, 132.1, 128.3 (d, $J_{C\text{-}F}$ = 9.8 Hz, 1C), 112.6, 111.1 (d, $J_{C\text{-}F}$ = 9.9 Hz, 1C), 108.8 (d, $J_{C\text{-}F}$ = 4.4 Hz, 1C), 108.5 (d, $J_{C\text{-}F}$ = 26.0 Hz, 1C), 103.5 (d, $J_{C\text{-}F}$ = 23.7 Hz, 1C), 49.5, 48.5, 32.1, 19.3, 16.4, 12.4.

F-NMR: -125.5

HRMS-ESI (m/z): calcd for $C_{19}H_{21}FNO$ [M + H$^+$] 298.1601, found 298.1603

FTIR (film, cm^{-1}): 3345, 2919, 1665, 1486, 1453, 1368, 892, 843, 793 cm^{-1}

M.p.: 63 - 64 ℃

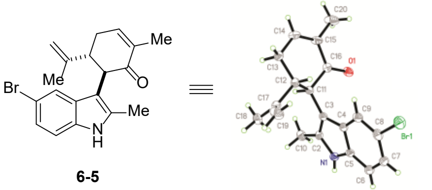

6-5

(5*R*,6*R*)-6-(5-bromo-2-methyl-1*H*-indol-3-yl)-2-methyl-5-(prop-1-en-2-yl)cyclohex-2-en-1-one (6-5) was synthesized according to **general procedure C** and obtained as a yellow solid (1 mmol scale, 275.8 mg, 77% yield).

^{1}H NMR (400 MHz, $CDCl_3$) δ 8.06 (s, 1H), 7.34 (d, *J* = 1.9 Hz, 1H), 7.09 (dd, *J* = 8.5, 1.9 Hz, 1H), 6.98 (d, *J* = 8.5 Hz, 1H), 6.89 – 6.85 (m, 1H), 4.57 (s, 1H), 4.50 (s, 1H), 3.70 (d, *J* = 13.2 Hz, 1H), 3.30 (ddd, *J* = 13.2, 11.3, 4.5 Hz, 1H), 2.63 (ddt, *J* = 18.6, 11.3, 2.6 Hz, 1H), 2.44 (dddd, *J* = 18.1, 5.9, 4.4, 1.4 Hz, 1H), 2.13 (s, 3H), 1.89 (s, 3H), 1.50 (s, 3H).

^{13}C NMR (101 MHz, $CDCl_3$) δ 199.7, 145.7, 144.5, 135.7, 134.4, 134.3, 129.7, 123.3, 120.9, 112.7, 112.3, 112.1, 108.2, 49.5, 48.6, 32.2, 19.3, 16.5, 12.3.

HRMS-ESI (m/z): calcd for $C_{19}H_{20}BrNONa$ [M + Na$^+$] 380.0620, found 380.0625

FTIR (film, cm^{-1}): 3336, 2888, 1670, 1473, 1364, 1310, 884, 801 cm^{-1}

M.p.: 155 – 156 ℃

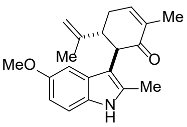

6-6

(5*R*, 6*R*)-6-(5-methoxy-2-methyl-1*H*-indol-3-yl)-2-methyl-5-(prop-1-en-2-yl)cyclohex-2-en-1-one (6-6)

was synthesized according to **general procedure C** and obtained as a yellow solid (0.5 mmol scale, 137.9 mg, 89% yield).

^{1}H NMR (400 MHz, $CDCl_3$) δ 7.83 (s, 1H), 7.03 (d, *J* = 8.6 Hz, 1H), 6.86 (d, *J* = 5.7 Hz, 1H), 6.74 – 6.67 (m, 2H), 4.59 (s, 1H), 4.52 (s, 1H), 3.80 (s, 3H), 3.73 (d, *J* = 12.9 Hz, 1H), 3.32 (td, *J* = 12.1, 4.4 Hz, 1H), 2.69 – 2.57 (m, 1H), 2.43 (dt, *J* = 18.1, 5.0 Hz, 1H), 2.14 (s, 3H), 1.89 (s, 3H), 1.52 (s, 3H).

^{13}C NMR (101 MHz, $CDCl_3$) δ 199.7, 153.6, 146.0, 144.1, 135.8, 133.7, 130.9, 128.6, 112.4, 111.1, 109.7, 108.5, 101.8, 56.1, 49.6, 48.5, 32.1, 19.4, 16.4, 12.5.

HRMS-ESI (m/z): calcd for $C_{20}H_{23}NO_2Na$ [M + Na$^+$] 332.1621, found 332.1623

FTIR (film, cm^{-1}): 3354, 2940, 1664, 1485, 1458, 1217, 1029, 902, 826 cm^{-1}

M.p.: 124 – 125 ℃

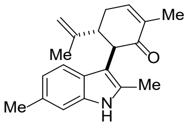

6-7

(5*R*,6*R*)-6-(2,6-dimethyl-1H-indol-3-yl)-2-methyl-5-(prop-1-en-2-yl)cyclohex-2-en-1-one (6-7) was synthesized according to **general procedure C** and obtained as a white solid (1 mmol scale, 267.2 mg, 91% yield).

^{1}H NMR (400 MHz, $CDCl_3$) δ 7.93 (s, 1H), 7.14 (d, *J* = 8.0 Hz, 1H), 6.87 (m, 2H), 6.82 (d, *J* = 8.0 Hz, 1H), 4.59 (s, 1H), 4.52 (s, 1H), 3.75 (d, *J* = 12.9 Hz, 1H), 3.35 (ddd, *J* = 13.0, 11.2, 4.4 Hz, 1H), 2.68 – 2.58 (m, 1H), 2.46 (t, *J* = 5.4 Hz, 1H), 2.40 (s, 3H), 2.11 (s, 3H), 1.90 (s, 3H), 1.53 (s, 3H).

^{13}C NMR (101 MHz, $CDCl_3$) δ 199.8, 146.1, 144.0, 136.0, 135.8, 131.9, 130.0, 125.8, 120.6, 118.2, 112.3, 110.8, 108.4, 49.6, 48.6, 32.1, 21.7, 19.5, 16.4, 12.2.

HRMS-API (m/z): calcd. for $C_{20}H_{24}NO$ [M + H+] 294.1852, found 294.1846

FTIR (film, cm^{-1}): 3367, 2920, 1674, 1466, 1367 cm^{-1}

M.p.: 161 – 162 ℃

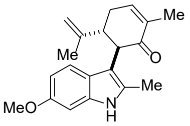

6-8

(5*R*,6*R*)-6-(6-methoxy-2-methyl-1*H*-indol-3-yl)-2-methyl-5-(prop-1-en-2-yl)cyclohex-2-en-1-one (6-8) was synthesized according to **general procedure C** and obtained as a yellow oil (1 mmol scale, 217.1 mg, 70% yield).

^{1}H NMR (400 MHz, $CDCl_3$) δ 7.90 (s, 1H), 7.11 (d, *J* = 8.5 Hz, 1H), 6.89 – 6.83 (m, 1H), 6.67 – 6.59 (m, 2H), 4.56 (s, 1H), 4.51 (s, 1H), 3.77 (s, 3H), 3.71 (d, *J* = 12.9 Hz, 1H), 3.32 (ddd, *J* = 13.0, 11.3, 4.4 Hz, 1H), 2.61 (ddt, *J* = 18.6, 11.3, 2.6 Hz, 1H), 2.47 – 2.38 (m, 1H), 2.11 (s, 3H), 1.89 (s, 3H), 1.51 (s, 3H).

^{13}C NMR (101 MHz, $CDCl_3$) δ 199.8, 155.4, 146.0, 144.0, 136.4, 135.8, 131.4, 122.4, 119.1, 112.3, 108.4, 108.4, 94.8, 55.7, 49.7, 48.7, 32.1, 19.5, 16.4, 12.3.

HRMS-ESI (m/z): calcd for $C_{20}H_{23}NO_2Na$ [M + Na$^+$] 332.1621, found 332.1623

FTIR (film, cm^{-1}): 3353, 2919, 1666, 1463, 1160 cm^{-1}

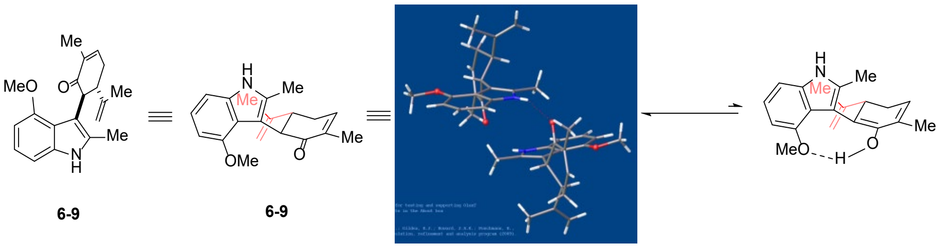

(5*R*,6*R*)-6-(4-methoxy-2-methyl-1*H*-indol-3-yl)-2-methyl-5-(prop-1-en-2-yl)cyclohex-2-en-1-one (6-9) was synthesized according to **general procedure C** and obtained as a gray solid (1 mmol scale, 163.0 mg, 53% yield). The H&C-NMR spectra of compound **6-9** is a little bit different compared to other products, probably due to its partial enolization to form an intramolecular H-bonding in solution.

^{1}H NMR (400 MHz, $CDCl_3$) δ 7.91 (s, 1H), 6.94 (t, *J* = 7.9 Hz, 1H), 6.79 (dd, *J* = 12.1, 7.2 Hz, 2H), 6.41 (d, *J* = 7.8 Hz, 1H), 4.53 (s, 2H), 3.75 (s, 3H), 3.47 (d, *J* = 60.0 Hz, 2H), 2.65 – 2.52 (m, 1H), 2.36 (dt, *J* = 17.8, 5.3 Hz, 1H), 2.14 (s, 3H), 1.89 (s, 3H), 1.47 (s, 3H).

^{13}C NMR (101 MHz, DMSO-*d*$_6$) δ 198.7, 152.4, 147.1, 142.6, 137.0, 134.4, 131.4, 120.4, 114.1, 108.4, 104.0, 99.2, 99.0, 54.5, 49.6, 48.7, 30.9, 20.2, 16.3, 11.5.

HRMS-API (m/z): calcd. for $C_{20}H_{24}NO_2$ [M + H$^+$] 310.1801, found 310.1803

FTIR (film, cm^{-1}): 3321, 2918, 1668, 1507, 1257, 1107, 732 cm^{-1}

M.p.: 136 – 137 ℃

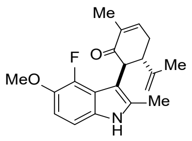

6-10

(5*R*,6*R*)-6-(4-fluoro-5-methoxy-2-methyl-1H-indol-3-yl)-2-methyl-5-(prop-1-en-2-yl)cyclohex-2-en-1-one (**6-10**) was synthesized according to **general procedure C** and obtained as a gray solid (1 mmol scale, 224.1 mg, 69% yield).

^{1}H NMR (400 MHz, $CDCl_3$) δ 7.79 (s, 1H), 6.88 – 6.75 (m, 3H), 4.57 (s, 1H), 4.53 (s, 1H), 3.86 (s, 3H), 3.72 (d, *J* = 7.7 Hz, 1H), 3.30 (td, *J* = 12.7, 2.7 Hz, 1H), 2.66 – 2.55 (m, 1H), 2.39 (dt, *J* = 18.0, 5.1 Hz, 1H), 2.21 (s, 3H), 1.86 (s, 3H), 1.58 (s, 3H).

^{13}C NMR (101 MHz, $CDCl_3$) δ C 198.8, 146.5, 144.1, 145.5 (d, J_{C-F} = 240 Hz, 1C), 139.0 (d, J_{C-F} = 10 Hz, 1C), 134.8 (d, J_{C-F} = 10 Hz, 1C), 134.1, 133.1 (d, J_{C-F} = 10 Hz, 1C), 11.8, 109.1, 106.2, 105.73, 105.70, 57.7, 49.1 (d, J_{C-F} = 5 Hz, 1C), 48.9, 31.2, 19.3, 16.2, 11.7.

F-NMR: -142.2 (br), -147.5.

HRMS-API (m/z): calcd. for $C_{20}H_{23}FNO_2$ [M + H$^+$] 328.1707, found 328.1708

FTIR (film, cm^{-1}): 3344, 2934, 1664, 1585, 1506, 1329, 1264, 1102, 1090 cm^{-1}

M.p.: 136 – 137 ℃

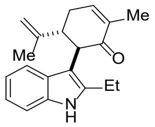

6-11

(5*R*,6*R*)-6-(2-ethyl-1*H*-indol-3-yl)-2-methyl-5-(prop-1-en-2-yl)cyclohex-2-en-1-one (**6-11**) was synthesized according to **general procedure C** and obtained as a yellow solid (1 mmol scale, 226.4 mg, 77% yield).

^{1}H NMR (400 MHz, $CDCl_3$) δ 7.96 (s, 1H), 7.27 (d, *J* = 7.6 Hz, 1H), 7.20 (d, *J* = 7.9 Hz, 1H), 7.05 (t, *J* = 7.5 Hz, 1H), 6.98 (t, *J* = 7.4 Hz, 1H), 6.86 (d, *J* = 6.0 Hz, 1H), 4.58 (s, 1H), 4.54 (s, 1H), 3.77 (d, *J* = 12.9 Hz, 1H), 3.38 (td, *J* = 12.1, 4.4 Hz, 1H), 2.68-2.56 (m, 3H), 2.44 (dt, *J* = 18.2, 5.1 Hz, 1H), 1.89 (s, 3H), 1.52 (s, 3H), 1.23 (t, *J* = 7.6 Hz, 3H).

^{13}C NMR (101 MHz, $CDCl_3$) δ 199.5, 146.0, 143.8, 138.1, 135.7, 135.6, 127.7, 120.6, 119.0, 118.8, 112.4, 110.6, 108.0, 49.7, 48.5, 31.9, 19.7, 19.6, 16.3, 13.7.

HRMS-ESI (m/z): calcd for $C_{20}H_{24}NO$ [M + H$^+$] 294.1852, found 294.1854

FTIR (film, cm^{-1}): 3361, 1669, 1462, 741 cm^{-1}

M.p.: 133 – 134 ℃

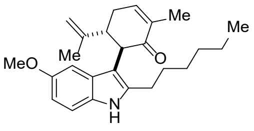

6-13

(5*R*,6*R*)-6-(2-hexyl-5-methoxy-1*H*-indol-3-yl)-2-methyl-5-(prop-1-en-2-yl)cyclohex-2-en-1-one (**6-13**) was synthesized according to **general procedure C** and obtained as a yellow solid (1 mmol scale, 239.3 mg, 63% yield).

^{1}H NMR (400 MHz, $CDCl_3$) δ 7.88 (s, 1H), 7.05 (d, *J* = 8.6 Hz, 1H), 6.86 (dt, *J* = 5.9, 2.0 Hz, 1H), 6.78 – 6.65 (m, 2H), 4.68 – 4.51 (m, 2H), 3.80 (s, 3H), 3.72 (d, *J* = 12.8 Hz, 1H), 3.35 (ddd, *J* = 12.7, 11.2, 4.4 Hz, 1H), 2.70 – 2.48 (m, 3H), 2.47 – 2.36 (m, 1H), 1.89 (dt, *J* = 2.6, 1.3 Hz, 3H), 1.63 – 1.55 (m, 2H), 1.53 (s, 3H), 1.40 – 1.19 (m, 6H), 0.89 (t, *J* = 6.6 Hz, 3H).

^{13}C NMR (101 MHz, $CDCl_3$) δ 199.5, 153.5, 146.1, 143.9, 138.3, 135.8, 130.9, 128.2, 112.4, 111.1, 109.7, 108.2, 102.1, 56.1, 49.7, 48.4, 31.9, 31.7, 29.4(x 2C), 26.8, 22.6, 19.8, 16.4, 14.1.

HRMS-ESI (m/z): calcd. for $C_{25}H_{34}NO_2$ [M + H$^+$] 380.2584, found 380.2587

FTIR (film, cm^{-1}): 3357, 2925, 1661, 1487, 1214, 900, 821, 789 cm^{-1}

M.p.: 133 – 134 ℃

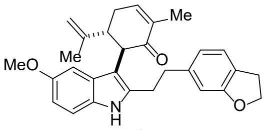

6-14

(5*R*,6*R*)-6-(2-(2-(2,3-dihydrobenzofuran-6-yl)ethyl)-5-methoxy-1*H*-indol-3-yl)-2-methyl-5-(prop-1-en-2-yl)cyclohex-2-en-1-one (**6-14**) was synthesized according to **general procedure C** and obtained as a white solid (0.5 mmol scale, 208.8 mg, 95% yield).

^{1}H NMR (400 MHz, $CDCl_3$) δ 7.77 (s, 1H), 7.02 (d, *J* = 8.7 Hz, 1H), 6.98 (d, *J* = 1.8 Hz, 1H), 6.88 (ddd, *J* = 9.2, 6.7, 2.0 Hz, 2H), 6.76 (d, *J* = 2.4 Hz, 1H), 6.74 – 6.68 (m, 2H), 4.63 (d, *J* = 1.8 Hz, 1H), 4.59 – 4.52 (m, 3H), 3.80 (s, 3H), 3.75 (d, *J* = 12.8 Hz, 1H), 3.36 (dd, *J* = 4.5, 1.6 Hz, 1H), 3.16 (t, *J* = 8.7 Hz, 2H),

2.86 – 2.72 (m, 4H), 2.69 – 2.57 (m, 1H), 2.54 – 2.38 (m, 1H), 1.90 (dt, *J* = 2.5, 1.3 Hz, 3H), 1.54 (s, 3H).

^{13}C NMR (101 MHz, $CDCl_3$) δ 199.4, 158.7, 153.7, 146.2, 143.9, 137.5, 135.8, 133.4, 130.9, 128.2, 127.9, 127.4, 125.2, 112.5, 111.2, 110.0, 109.2, 108.6, 102.2, 71.3, 56.1, 49.8, 48.5, 35.2, 32.0, 29.9, 29.2, 19.9, 16.5.

HRMS-ESI (m/z): calcd for $C_{29}H_{32}NO_3$ [M + H$^+$] 442.2376, found 442.2380

FTIR (film, cm^{-1}): 3342, 2919, 1664, 1490, 1240, 1215 cm^{-1}

M.p.: 152 – 153 ℃

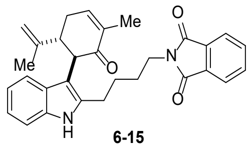

6-15

2-(4-(3-((1*R*,6*R*)-3-methyl-2-oxo-6-(prop-1-en-2-yl)cyclohex-3-en-1-yl)-1*H*-indol-2-yl)butyl)isoindoline-1,3-dione (**6-15**) was synthesized according to **general procedure C** and obtained as a white solid (0.5 mmol scale, 145.5 mg, 63% yield).

^{1}H NMR (400 MHz, $CDCl_3$) δ 8.42 (s, 1H), 7.86 (dd, *J* = 5.4, 3.0 Hz, 2H), 7.71 (dd, *J* = 5.4, 3.0 Hz, 2H), 7.30 (d, *J* = 7.8 Hz, 1H), 7.25 (d, *J* = 7.9 Hz, 1H), 7.11 – 7.04 (m, 1H), 6.99 (t, *J* = 7.4 Hz, 1H), 6.94 – 6.85 (m, 1H), 4.60 (s, 1H), 4.53 (t, *J* = 1.7 Hz, 1H), 3.89 – 3.65 (m, 3H), 3.42 (td, *J* = 12.0, 4.4 Hz, 1H), 2.80 – 2.56 (m, 3H), 2.52 – 2.41 (m, 1H), 1.94 – 1.85 (m, 3H), 1.75 (q, *J* = 6.7 Hz, 2H), 1.65 (qd, *J* = 7.8, 5.3 Hz, 2H), 1.52 (s, 3H).

^{13}C NMR (101 MHz, $CDCl_3$) δ 199.4, 168.6(x 2C), 146.1, 143.9, 136.5, 135.7, 135.6, 134.0(x 2C), 132.0(x 2C), 127.5, 123.2(x 2C), 120.6, 118.9, 118.8, 112.4, 110.7, 108.6, 49.7, 48.4, 37.2, 31.9, 28.1, 26.6, 25.7, 19.8, 16.4.

HRMS-ESI (m/z): calcd for $C_{30}H_{31}N_2O_3^+$ [M + H$^+$] 467.2329, found 467.2332

FTIR (film, cm^{-1}): 3383, 2943, 2922, 1768, 1706, 1671, 1463, 1438, 1397, 1371, 1036, 893, 722 cm^{-1}

M.p.: 118 – 119 ℃

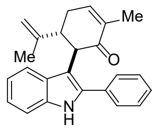

6-16

(5*R*,6*R*)-2-methyl-6-(2-phenyl-1H-indol-3-yl)-5-(prop-1-en-2-yl)cyclohex-2-en-1-one (**6-16**) was synthesized according to **general procedure C** and obtained as a white solid (1 mmol scale, 254.0 mg, 74% yield).

^{1}H NMR (400 MHz, $CDCl_3$) δ 8.0 (s, 1H), 7.5 – 7.4 (m, 6H), 7.3 (d, *J* = 8.0 Hz, 1H), 7.1 (t, *J* = 7.5 Hz, 1H), 7.0 (t, *J* = 7.5 Hz, 1H), 6.9 (d, *J* = 6.2 Hz, 1H), 4.3 (t, *J* = 1.8 Hz, 1H), 4.2 (s, 1H), 3.9 (d, *J* = 13.1 Hz, 1H), 3.5 – 3.4 (m, 1H), 2.5 (ddt, *J* = 18.7, 11.4, 2.7 Hz, 1H), 2.3 (dt, *J* = 18.4, 5.6 Hz, 1H), 2.0 – 1.9 (m, 3H), 1.2 (s, 3H).

^{13}C NMR (101 MHz, $CDCl_3$) δ 200.3, 145.2, 144.5, 137.3, 136.3, 135.6, 133.4, 128.9(x 2C), 128.8(x 2C), 128.1, 122.0, 120.1, 119.6, 112.8, 111.3, 110.3, 77.2, 49.7, 48.6, 31.9, 18.2, 16.5.

HRMS-ESI (m/z): calcd for $C_{24}H_{24}NO$ [M + H$^+$] 342.1852, found 342.1855

FTIR (film, cm^{-1}): 3338, 3056, 2916, 1651, 1448, 1370, 1242, 763, 741, 699 cm^{-1}

M.p.: 153 – 154 ℃

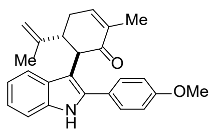

6-17

(5*R*,6*R*)-6-(2-(4-methoxyphenyl)-1H-indol-3-yl)-2-methyl-5-(prop-1-en-2-yl)cyclohex-2-en-1-one (**6-17**) was synthesized according to **general procedure C** and obtained as a white solid (1 mmol scale, 256.4 mg, 69% yield).

^{1}H NMR (400 MHz, $CDCl_3$) δ 8.00 (s, 1H), 7.40–7.32 (m, 3H), 7.27–7.23 (m, 1H), 7.09 (t, *J* = 7.5 Hz, 1H), 7.01 (t, *J* = 7.5 Hz, 1H), 6.95–6.90 (m, 2H), 6.84 (d, *J* = 6.1 Hz, 1H), 4.30 (d, *J* = 1.8 Hz, 1H), 4.24 (s, 1H), 3.88 (d, *J* = 13.2 Hz, 1H), 3.83 (s, 3H), 3.48–3.38 (m, 1H), 2.50–2.37 (m, 1H), 2.36–2.25 (m, 1H), 1.91 (s, 3H), 1.18 (s, 3H).

^{13}C NMR (101 MHz, $CDCl_3$) δ 200.4, 159.5, 145.3, 144.4, 137.2, 136.2, 135.7, 130.1 (x 2C), 127.8, 125.9, 121.7, 119.8, 119.5, 114.3 (x 2C), 112.7, 111.1, 109.7, 55.4, 49.8, 48.5, 31.9, 18.3, 16.5.

HRMS-ESI (m/z): calcd for $C_{25}H_{26}NO_2^+$ [M + H$^+$] 372.1958, found 372.1961

FTIR (film, cm^{-1}): 3347, 2920, 1660, 1506, 1458, 1248, 834, 743 cm^{-1}

M.p.: 140 – 141 ℃

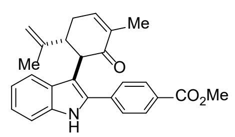

6-18

ethyl 4-(3-((1R,6R)-3-methyl-2-oxo-6-(prop-1-en-2-yl)cyclohex-3-en-1-yl)-1H-indol-2-yl)benzoate (**6-18**) was synthesized according to **general procedure C** and obtained as a white solid (0.5 mmol scale, 46.5 mg, 23% yield).

^{1}H NMR (400 MHz, $CDCl_3$) δ 8.43 (s, 1H), 8.14 – 7.88 (m, 2H), 7.52 – 7.42 (m, 2H), 7.38 (d, *J* = 7.9 Hz, 1H), 7.16 (d, *J* = 8.2 Hz, 1H), 7.08 (t, *J* = 7.6 Hz, 1H), 7.02 (t, *J* = 7.4 Hz, 1H), 6.87 (d, *J* = 6.0 Hz, 1H), 4.26 (s, 1H), 4.18 (s, 1H), 3.95 (s, 1H), 3.93 (s, 3H), 3.43 (td, *J* = 12.3, 4.6 Hz, 1H), 2.44 (dd, *J* = 18.2, 11.6 Hz, 1H), 2.32 (dt, *J* = 18.3, 5.4 Hz, 1H), 1.94 (s, 3H), 1.10 (s, 3H).

^{13}C NMR (101 MHz, $CDCl_3$) δ 200.2, 167.0, 145.0, 144.8, 138.0, 136.7, 136.0, 135.6, 130.1 (x 2C), 129.2, 128.4 (x 2C), 127.5, 122.5, 120.1, 119.8, 112.9, 111.5, 111.5, 52.3, 49.8, 48.5, 31.9, 18.2, 16.4.

HRMS-API (m/z): calcd. for $C_{26}H_{26}NO_3$ [M + H$^+$] 400.1907, found 400.1908

FTIR (film, cm^{-1}): 3352, 2950, 1725, 1656, 1610, 1435, 1276, 1102, 741 cm^{-1}

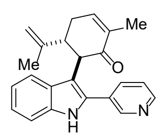

6-19

(5*R*,6*R*)-2-methyl-5-(prop-1-en-2-yl)-6-(2-(pyridin-3-yl)-1H-indol-3-yl)cyclohex-2-en-1-one (**6-19**) was synthesized according to **general procedure C** and obtained as a yellow solid (1 mmol scale, 131.9 mg, 39% yield).

^{1}H NMR (600 MHz, $CDCl_3$) δ 8.94 (s, 1H), 8.67 (s, 1H), 8.52 (d, *J* = 6.1 Hz, 1H), 7.70 (d, *J* = 7.9 Hz, 1H), 7.38 (d, *J* = 7.9 Hz, 1H), 7.29 – 7.25 (m, 1H), 7.17 (d, *J* = 8.1 Hz, 1H), 7.08 (t, *J* = 8.0 Hz, 1H), 7.02 (t, *J* = 7.5 Hz, 1H), 6.86 (d, *J* = 5.8 Hz, 1H), 4.30 (s, 1H), 4.20 (s, 1H), 3.83 (d, *J* = 13.1 Hz, 1H), 3.42 (td, *J* = 12.4, 4.5 Hz, 1H), 2.47 – 2.40 (m, 1H), 2.32 (dt, *J* = 18.5, 5.0 Hz, 1H), 1.90 (s, 3H), 1.13 (s, 3H).

^{13}C NMR (101 MHz, $CDCl_3$) δ 200.1, 149.2, 148.6, 144.9, 144.7, 136.8, 136.1, 135.5, 133.5, 129.6, 127.3, 123.6, 122.3, 119.9, 119.6, 112.8, 111.6, 111.3, 49.7, 48.3, 31.8, 18.5, 16.3.

HRMS-API (m/z): calcd. for $C_{23}H_{23}N_2O$ [M + H$^+$] 343.1804, found 343.1806

FTIR (film, cm^{-1}): 3333, 2922, 1666, 1455, 743 cm^{-1}

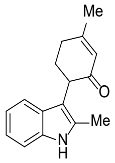

6-20

3-methyl-6-(2-methyl-1*H*-indol-3-yl)cyclohex-2-en-1-one (**6-20**) was synthesized according to **general procedure C** and obtained as a yellow solid (1 mmol scale, 102.9 mg, 46% yield).

^{1}H NMR (400 MHz, $CDCl_3$) δ 7.88 (s, 1H), 7.24 (d, *J* = 9.0 Hz, 2H), 7.07 (ddd, *J* = 8.1, 7.1, 1.3 Hz, 1H), 7.00 (ddd, *J* = 8.1, 7.1, 1.2 Hz, 1H), 6.11 (s, 1H), 3.70 (dd, *J* = 12.9, 5.1 Hz, 1H), 2.49 – 2.33 (m, 3H), 2.29 (s, 3H), 2.17 – 2.10 (m, 1H), 2.05 (t, *J* = 1.2 Hz, 3H).

^{13}C NMR (101 MHz, $CDCl_3$) δ 199.6, 162.5, 135.6, 132.2, 127.5, 127.3, 120.8, 119.1, 118.7, 110.7, 110.2, 44.2, 31.7, 30.5, 24.5, 12.1.

HRMS-ESI (m/z): calcd for $C_{16}H_{18}NO$ [M + H$^+$] 240.1382, found 240.1384

FTIR (film, cm^{-1}): 3311, 2910, 1647, 1462, 1214, 743 cm^{-1}

M.p.: 165 – 167 ℃

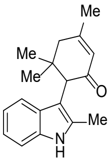

6-21

3,5,5-trimethyl-6-(2-methyl-1*H*-indol-3-yl)cyclohex-2-en-1-one (**6-21**) was synthesized according to **general procedure C** and obtained as oil (1 mmol scale, 168.5 mg, 63% yield).

^{1}H NMR (400 MHz, $CDCl_3$) δ 8.07 (s, 1H), 7.16 (t, *J* = 6.9 Hz, 2H), 7.00 (dt, *J* = 29.8, 7.6 Hz, 2H), 6.14 (d, *J* = 29.7 Hz, 1H), 3.54 (s, 1H), 2.55 (dd, *J* = 35.3, 17.9 Hz, 1H), 2.26 (d, *J* = 17.8 Hz, 1H), 2.19 (d, *J* = 5.7 Hz, 3H), 2.04 (d, *J* = 8.2 Hz, 3H), 1.00 (d, *J* = 2.5 Hz, 6H).

^{13}C NMR (101 MHz, $CDCl_3$) δ 199.4, 159.9, 135.7, 134.5, 127.8, 126.7, 121.5, 120.5, 118.9, 110.4, 107.0, 55.2, 47.1, 40.2, 29.8, 24.8, 24.4, 12.4.

HRMS-API (m/z): calcd. for $C_{18}H_{22}NO$ [M + H$^+$] 268.1695, found 268.1697

FTIR (film, cm^{-1}): 3311, 2946, 1650, 1461, 1310, 1292, 1222, 731, 727 cm^{-1}

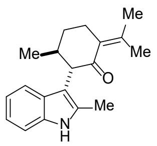

6-22

(2*S*,3*S*)-3-methyl-2-(2-methyl-1*H*-indol-3-yl)-6-(propan-2-ylidene)cyclohexan-1-one (**6-22**) was synthesized according to **general procedure C** and obtained as a yellow solid (1 mmol scale, 236.5 mg, 84% yield).

^{1}H NMR (400 MHz, $CDCl_3$) δ 7.92 (s, 1H), 7.28 – 7.24 (m, 1H), 7.21 – 7.16 (m, 1H), 7.06 (ddd, *J* = 8.1, 7.0, 1.3 Hz, 1H), 6.99 (ddd, *J* = 8.1, 7.0, 1.1 Hz, 1H), 3.28 (d, *J* = 11.5 Hz, 1H), 2.91 – 2.81 (m, 1H), 2.63 – 2.51 (m, 1H), 2.39 – 2.32 (m, 1H), 2.30 (s, 3H), 2.03 (ddt, *J* = 13.3, 5.1, 3.0 Hz, 1H), 1.96 – 1.91 (m, 3H), 1.87 (d, *J* = 1.3 Hz, 3H), 1.55 (tdd, *J* = 13.1, 11.7, 4.7 Hz, 1H), 0.85 (d, *J* = 6.4 Hz, 3H).

^{13}C NMR (101 MHz, $CDCl_3$) δ 204.6, 142.6, 135.6, 132.8, 132.7, 127.6, 120.7, 119.0, 118.9, 110.5, 110.2, 56.7, 37.1, 32.9, 29.1, 23.1, 22.3, 21.3, 12.3.

HRMS-API (m/z): calcd. for $C_{19}H_{24}NO$ [M + H$^+$] 282.1852, found 282.1854

FTIR (film, cm^{-1}): 3392, 2919, 1670, 1461, 1305, 1287, 744 cm^{-1}

M.p.: 117 – 119 ℃

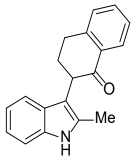

6-23

2-(2-methyl-1H-indol-3-yl)-3,4-dihydronaphthalen-1(2H)-one (6-23) (Maksymenko et al., 2017) was synthesized according to **general procedure C** and obtained as a yellow solid (1 mmol scale, 199.5 mg, 79% yield).

^{1}H NMR (400 MHz, DMSO-*d*$_6$) δ 10.78 (s, 1H), 7.95 (dd, *J* = 7.8, 1.5 Hz, 1H), 7.60 (td, *J* = 7.5, 1.5 Hz, 1H), 7.47 – 7.37 (m, 2H), 7.25 (dt, *J* = 8.2, 0.9 Hz, 1H), 7.07 (d, *J* = 7.9 Hz, 1H), 6.95 (ddd, *J* = 8.2, 7.0, 1.2 Hz, 1H), 6.81 (ddd, *J* = 8.0, 7.0, 1.1 Hz, 1H), 4.10 (dd, *J* = 13.0, 4.7 Hz, 1H), 3.24 (td, *J* = 12.4, 6.2 Hz, 1H), 3.01 (dt, *J* = 16.3, 3.7 Hz, 1H), 2.43 (td, *J* = 12.8, 4.0 Hz, 1H), 2.28 (s, 3H), 2.22 – 2.14 (m, 1H).

^{13}C NMR (101 MHz, DMSO-*d*$_6$) δ 197.9, 144.7, 135.3, 133.3, 132.7, 132.4, 129.0, 127.2, 126.8, 126.6, 119.7, 118.1, 118.0, 110.5, 109.9, 45.4, 30.9, 29.1, 11.7.

HRMS-ESI (m/z): calcd for $C_{19}H_{18}NO$ [M + H$^+$] 276.1382, found 276.1384

FTIR (film, cm^{-1}): 3225, 1670, 1596, 1455, 1308, 1224, 742 cm^{-1}

M.p.: 124 – 125 ℃

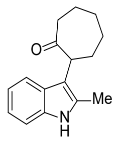

6-24

2-(2-methyl-1*H*-indol-3-yl)cycloheptan-1-one (6-24) was synthesized according to **general procedure C** and obtained as a light brown solid (1 mmol scale, 153.8 mg, 64% yield).

^{1}H NMR (400 MHz, $CDCl_3$) δ 8.01 (s, 1H), 7.53 – 7.47 (m, 1H), 7.21 – 7.14 (m, 1H), 7.07 (tt, *J* = 7.9, 5.9 Hz, 2H), 3.91 (dd, *J* = 11.4, 3.1 Hz, 1H), 2.89 (ddd, *J* = 14.5, 11.9, 2.6 Hz, 1H), 2.74 – 2.65 (m, 1H), 2.44 – 2.29 (m, 1H), 2.28 (d, 3H), 2.07 (ddt, *J* = 17.0, 13.0, 6.8 Hz, 4H), 1.72 (qt, *J* = 13.6, 2.5 Hz, 1H), 1.56 (pd, *J* = 12.7, 10.6, 4.4 Hz, 2H).

^{13}C NMR (101 MHz, $CDCl_3$) δ 214.7, 135.4, 131.7, 127.6, 120.9, 119.2, 118.9, 111.6, 110.6, 50.9, 44.1, 31.9, 30.6, 30.1, 25.3, 12.6.

HRMS-ESI (m/z): calcd for $C_{16}H_{19}NONa$ [M + Na$^+$] 264.1358, found 264.1360

FTIR (film, cm^{-1}): 3364, 2932, 1690, 1461, 743 cm^{-1}

M.p.: 76 – 77 ℃

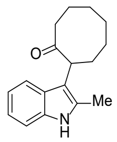

6-25

2-(2-methyl-1*H*-indol-3-yl)cycloheptan-1-one (6-25) was synthesized according to **general procedure C** and obtained as a light brown oil (1 mmol scale, 184.2 mg, 72% yield).

^{1}H NMR (400 MHz, $CDCl_3$) δ 8.21 (s, 1H), 7.98 (dd, *J* = 6.2, 3.0 Hz, 1H), 7.35 (dd, *J* = 6.1, 2.9 Hz, 1H), 7.24 (dt, *J* = 6.0, 2.8 Hz, 2H), 4.12 (d, *J* = 12.6 Hz, 1H), 3.10 – 2.89 (m, 2H), 2.59 (d, *J* = 2.2 Hz, 3H), 2.49 – 2.40 (m, 1H), 2.17 (s, 1H), 2.10 (dt, *J* = 10.3, 5.6 Hz, 2H), 2.03 – 1.90 (m, 2H), 1.84 – 1.55 (m, 4H).

^{13}C NMR (101 MHz, $CDCl_3$) δ 216.3, 135.2, 132.8, 127.6, 120.3, 119.3, 118.9, 110.5, 108.3, 50.6, 40.2,

39.7, 29.9, 27.6, 26.4, 24.7, 12.6.

HRMS-API (m/z): calcd. for $C_{17}H_{22}NO$ [M + H$^+$] 256.1695, found 256.1697

FTIR (film, cm^{-1}): 3357, 2926, 2854, 1689, 1459, 740 cm^{-1}

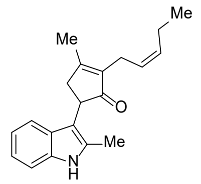

6-26

3-methyl-5-(2-methyl-1H-indol-3-yl)-2-pentylcyclopent-2-en-1-one (6-26) was synthesized according to **general procedure C** and obtained as a brown solid (1 mmol scale, 157.4 mg, 54% yield).

^{1}H NMR (400 MHz, $CDCl_3$) δ 7.97 (s, 1H), 7.21 (d, *J* = 8.0 Hz, 1H), 7.08 – 6.99 (m, 2H), 6.93 (t, *J* = 7.4 Hz, 1H), 5.48 – 5.31 (m, 2H), 3.71 (dd, *J* = 7.4, 3.0 Hz, 1H), 3.03 (dd, *J* = 45.9, 7.2 Hz, 3H), 2.72 (d, *J* = 18.5 Hz, 1H), 2.28 – 2.09 (m, 8H), 1.01 (t, *J* = 7.5 Hz, 3H).

^{13}C NMR (101 MHz, $CDCl_3$) δ 209.8, 169.5, 139.2, 135.7, 132.8, 132.7, 127.1, 125.2, 121.0, 119.2, 117.9, 110.7, 109.6, 42.8, 40.6, 21.7, 20.8, 17.4, 14.3, 11.7.

HRMS-ESI (m/z): calcd for $C_{20}H_{24}NO$ [M + H$^+$] 294.1852, found 294.1854

FTIR (film, cm^{-1}): 3314, 1687, 1639, 1620, 1461, 737 cm^{-1}

M.p.: 106 – 107 ℃

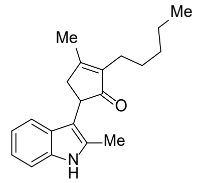

6-27

(*Z*)-3-methyl-5-(2-methyl-1*H*-indol-3-yl)-2-(pent-2-en-1-yl)cyclopent-2-en-1-one (6-27) was synthesized according to **general procedure C** and obtained as a brown solid (1 mmol scale, 142.1 mg, 48% yield).

^{1}H NMR (400 MHz, $CDCl_3$) δ 8.54 (s, 1H), 7.17 – 7.10 (m, 1H), 7.07 – 7.00 (m, 2H), 6.99 – 6.91 (m, 1H), 3.69 (dd, *J* = 7.3, 3.0 Hz, 1H), 2.98 (dd, *J* = 18.6, 7.3 Hz, 1H), 2.79 – 2.68 (m, 1H), 2.37 (dt, *J* = 8.1, 3.8 Hz, 2H), 2.17 (s, 3H), 1.86 (s, 3H), 1.61 – 1.49 (m, 2H), 1.38 (h, *J* = 3.9 Hz, 4H), 0.94 (t, *J* = 6.8 Hz, 3H).

^{13}C NMR (101 MHz, $CDCl_3$) δ 210.9, 169.6, 140.4, 135.7, 133.3, 127.0, 120.6, 118.8, 117.5, 110.8, 108.9, 42.8, 40.4, 31.9, 28.2, 23.4, 22.6, 17.2, 14.1, 11.1.

HRMS-API (m/z): calcd. for $C_{20}H_{26}NO$ [M + H$^+$] 296.2008, found 296.2011

FTIR (film, cm^{-1}): 3330, 2927, 1688, 1638, 1462, 735 cm^{-1}

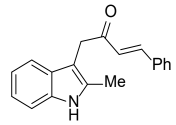

6-28

(*E*)-1-(2-methyl-1*H*-indol-3-yl)-4-phenylbut-3-en-2-one (6-28) was synthesized according to **general procedure C** and obtained as a light brown solid (1 mmol scale, 60.7 mg, 22% yield).

^{1}H NMR (400 MHz, $CDCl_3$) δ 8.01 (s, 1H), 7.63 (d, *J* = 16.0 Hz, 1H), 7.54 – 7.48 (m, 1H), 7.44 – 7.39 (m, 2H), 7.30 (d, *J* = 7.1 Hz, 3H), 7.22 (t, *J* = 4.4 Hz, 1H), 7.14 – 7.06 (m, 2H), 6.78 (d, *J* = 16.0 Hz, 1H), 3.92 (s, 2H), 2.34 (s, 3H).

^{13}C NMR (101 MHz, $CDCl_3$) δ 197.8, 142.8, 135.4, 134.5, 133.1, 130.4, 129.8, 128.9 (x 2C), 128.4 (x 2C), 124.6, 121.3, 119.7, 117.9, 110.5, 104.3, 38.2, 11.9.

HRMS-API (m/z): calcd. for $C_{19}H_{18}NO$ [M + H$^+$] 276.1382, found 276.1384

FTIR (film, cm^{-1}): 3346, 1651, 1175, 747 cm^{-1}

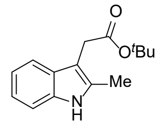

6-29

***Tert*-butyl 2-(2-methyl-1*H*-indol-3-yl)acetate (6-29)** was synthesized according to **general procedure C** and obtained as a yellow solid (1 mmol scale, 102.1 mg, 42% yield).

^{1}H NMR (600 MHz, $CDCl_3$) δ 8.00 (s, 1H), 7.58 (dd, *J* = 5.8, 3.2 Hz, 1H), 7.17 – 7.11 (m, 3H), 3.64 (s, 2H), 2.27 (s, 3H), 1.48 (s, 9H).

^{13}C NMR (101 MHz, $CDCl_3$) δ 171.7, 135.3, 132.8, 128.6, 121.0, 119.3, 118.2, 110.4, 105.0, 80.7, 31.8, 28.2(x 3C), 11.6.

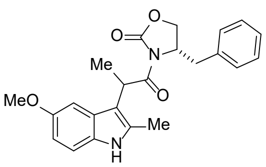

6-30 (1.9:1 dr)

(4*S*)-4-benzyl-3-(2-(5-methoxy-2-methyl-1*H*-indol-3-yl)propanoyl)oxazolidin-2-one (**6-30**) was synthesized according to **general procedure C** and obtained as a yellow solid (1 mmol scale, 258.6 mg, 66% yield).

^{1}H NMR (400 MHz, $CDCl_3$, data for major diastereomer given) δ 8.01 (s, 1H), 7.46 – 7.13 (m, 6H), 6.97 (dd, *J* = 7.3, 2.1 Hz, 1H), 6.83 (ddd, *J* = 8.8, 5.0, 2.4 Hz, 1H), 5.30 (dq, *J* = 9.6, 7.0 Hz, 1H), 4.86 (tt, *J* = 8.8, 3.7 Hz, 1H), 4.20 (t, *J* = 8.7 Hz, 1H), 4.10 – 4.01 (m, 1H), 3.95 (s, 1H), 3.90 (s, 2H), 3.19 (dd, *J* = 13.5, 3.4 Hz, 1H), 2.55 (s, 3H), 2.48 – 2.43 (m, 1H), 1.67 (dd, *J* = 7.1, 5.7 Hz, 3H).

^{13}C NMR (101 MHz, $CDCl_3$) major δ = 174.9, 153.8, 153.1, 135.1, 134.3, 130.3, 129.3 (x 2C), 128.7 (x 2C), 127.5, 127.1, 111.1, 110.8, 109.0, 101.2, 65.9, 55.8, 55.0, 37.8, 35.9, 17.2, 12.3; minor δ = 174.9, 153.8, 153.0, 135.5, 134.2, 130.4, 129.5 (x 2C), 128.9 (x 2C), 127.5, 127.3, 111.0, 110.4, 109.2, 101.5, 65.8, 56.1, 56.0, 37.8, 35.5, 17.4, 12.1.

HRMS-ESI (m/z): calcd for $C_{23}H_{25}N_2O_4$ [M + H$^+$] 393.1809, found 393.1811

FTIR (film, cm^{-1}): 3395, 2931, 1778, 1697, 1485, 1452, 1389, 1356, 1215 cm^{-1}

M.p.: 73 – 75 ℃

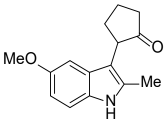

6-31

2-(5-methoxy-2-methyl-1*H*-indol-3-yl)cyclopentan-1-one (6-31) was synthesized according to **general procedure C** and obtained as a yellow solid by using LDA instead of LiHMDS (1 mmol scale, 95.1 mg, 39% yield).

^{1}H NMR (400 MHz, $CDCl_3$) δ 7.90 (s, 1H), 7.09 (d, *J* = 8.7 Hz, 1H), 6.74 (dd, *J* = 8.7, 2.4 Hz, 1H), 6.69 (d, *J* = 2.4 Hz, 1H), 3.81 (s, 3H), 3.54 – 3.42 (m, 1H), 2.58 (ddd, *J* = 18.8, 8.7, 2.1 Hz, 1H), 2.51 – 2.33 (m, 2H), 2.28 – 2.10 (m, 5H), 2.08 – 1.91 (m, 1H).

^{13}C NMR (101 MHz, $CDCl_3$) δ 220.0, 153.8, 133.7, 130.9, 127.8, 111.3, 110.1, 108.3, 101.4, 56.2, 47.5, 38.7, 31.1, 21.4, 12.1.

HRMS-API (m/z): calcd. for $C_{15}H_{18}NO_2$ [M + H$^+$] 244.1332, found 244.1333

FTIR (film, cm^{-1}): 3394, 2940, 1731, 1484, 1214 cm^{-1}

M.p.: 101 – 103 ℃

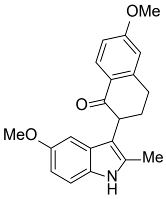

6-32

6-methoxy-2-(5-methoxy-2-methyl-1H-indol-3-yl)-3,4-dihydronaphthalen-1(2*H*)-one (6-32) was synthesized according to **general procedure C** and obtained as a light green solid (1 mmol scale, 164.3 mg, 49% yield).

^{1}H NMR (400 MHz, $CDCl_3$) δ 8.29 – 8.09 (m, 2H), 7.01 – 6.95 (m, 1H), 6.90 (dd, *J* = 8.7, 2.5 Hz, 1H), 6.79 (d, *J* = 2.5 Hz, 1H), 6.76 – 6.63 (m, 2H), 3.93 (d, *J* = 4.8 Hz, 1H), 3.89 (s, 3H), 3.73 (s, 3H), 3.16 (ddd, *J* = 16.4, 12.0, 4.4 Hz, 1H), 3.01 (dt, *J* = 16.5, 4.0 Hz, 1H), 2.54 (qd, *J* = 12.6, 4.1 Hz, 1H), 2.27 (dq, *J* = 13.2, 4.4 Hz, 1H), 2.07 (s, 3H).

^{13}C NMR (101 MHz, $CDCl_3$) δ 197.7, 163.6, 153.5, 147.1, 133.4, 130.8, 130.3, 127.9, 126.9, 113.3, 112.6, 111.3, 110.1, 109.9, 101.3, 55.9, 55.5, 46.0, 31.0, 30.2, 12.0.

HRMS-ESI (m/z): calcd for $C_{21}H_{22}NO_3$ [M + H$^+$] 336.1594, found 336.1597

FTIR (film, cm^{-1}): 3291, 1656, 1594, 1484, 1284, 1251, 1212 cm^{-1}

M.p.: 144 – 145 ℃

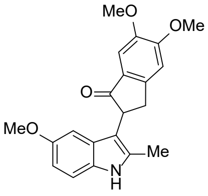

6-33

5,6-dimethoxy-2-(5-methoxy-2-methyl-1*H*-indol-3-yl)-2,3-dihydro-1*H*-inden-1-one (**6-33**) was synthesized according to **general procedure C** and obtained as a light brown solid (1 mmol scale, 122.4 mg, 33% yield).

^{1}H NMR (400 MHz, DMSO-*d$_6$*) 10.66 (s, 1H), 7.16 (dd, *J* = 16.7, 9.2 Hz, 3H), 6.61 (dd, *J* = 8.7, 2.4 Hz,

1H), 6.25 (d, *J* = 2.4 Hz, 1H), 4.06 (dd, *J* = 8.1, 3.9 Hz, 1H), 3.91 (s, 3H), 3.84 (s, 3H), 3.62 – 3.52 (m, 1H), 3.51 (s, 3H), 3.07 (dd, *J* = 17.4, 3.8 Hz, 1H), 2.27 (s, 3H).

^{13}C NMR (101 MHz, DMSO-*d₆*) 205.4, 155.4, 152.7, 149.3, 148.8, 133.7, 130.5, 128.9, 127.3, 111.1, 109.0, 108.8, 108.1, 104.0, 100.5, 56.0, 55.6, 55.1, 44.3, 34.2, 11.6.

HRMS-API (m/z): calcd. for $C_{21}H_{22}NO_4$ [M + H$^+$] 352.1543, found 352.1543

FTIR (film, cm^{-1}): 3375, 2922, 1699, 1588, 1501, 1487, 1310, 1264, 1220, 1112, 1033, 801 cm^{-1}

M.p.: 201 – 202 ℃

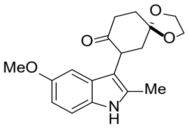

6-34

7-(5-methoxy-2-methyl-1*H*-indol-3-yl)-1,4-dioxaspiro[4.5]decan-8-one (**6-34**) was synthesized according to **general procedure C** and obtained as a yellow solid (1 mmol scale, 128.4 mg, 41% yield).

^{1}H NMR (400 MHz, $CDCl_3$) δ 7.99 (s, 1H), 7.02 (d, *J* = 8.6 Hz, 1H), 6.77 – 6.67 (m, 2H), 4.13 – 4.06 (m, 2H), 4.00 (dt, *J* = 5.7, 1.5 Hz, 2H), 3.81 (s, 3H), 2.94 – 2.80 (m, 1H), 2.62 – 2.46 (m, 2H), 2.22 – 2.14 (m, 5H), 2.08 – 1.93 (m, 2H).

^{13}C NMR (101 MHz, $CDCl_3$) δ 208.9, 153.7, 133.2, 130.7, 128.0, 111.2, 110.2, 107.8, 107.5, 101.5, 64.8, 64.7, 56.2, 44.6, 40.9, 38.4, 34.4, 12.4.

HRMS-ESI (m/z): calcd for $C_{18}H_{22}NO_4$ [M + H$^+$] 316.1543, found 316.1545

FTIR (film, cm^{-1}): 3349, 2888, 1712, 1487, 1454, 1216, 1122, 1029 cm^{-1}

M.p.: 150 – 151 ℃

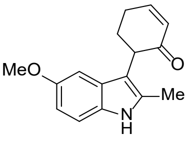

6-35

≡

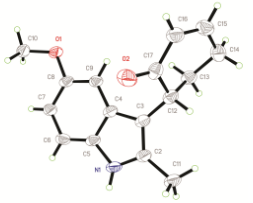

6-(5-methoxy-2-methyl-1*H*-indol-3-yl)cyclohex-2-en-1-one (**6-35**) was synthesized according to **general procedure C** and obtained as a yellow solid (1 mmol scale, 162.4 mg, 64% yield).

^{1}H NMR (400 MHz, DMSO-*d₆*) δ 10.62 (s, 1H), 7.16 (dd, *J* = 15.2, 8.1 Hz, 2H), 6.71 – 6.60 (m, 2H), 6.14

– 6.07 (m, 1H), 3.85 (dd, *J* = 13.5, 4.8 Hz, 1H), 3.69 (s, 3H), 2.59 (dddt, *J* = 18.4, 13.7, 4.5, 2.3 Hz, 1H), 2.48 – 2.38 (m, 1H), 2.35 – 2.27 (m, 1H), 2.23 (s, 3H), 2.03 (ddt, *J* = 13.9, 6.5, 2.6 Hz, 1H).

^{13}C NMR (101 MHz, DMSO-*d*$_6$) δ 198.9, 152.7, 151.1, 133.1, 130.4, 129.5, 127.8, 110.9, 109.4, 109.1, 100.9, 55.4, 44.4, 30.3, 26.0, 11.9.

HRMS-ESI (m/z): calcd for C_{16}H18NO_2 [M + H$^+$] 256.1332, found 256.1334

FTIR (film, cm^{-1}): 3281, 2931, 1656, 1481, 1215, 1032, 797 cm^{-1}

M.p.: 144 – 145 ℃

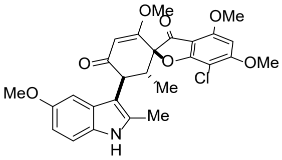

6-36

(5'*S*,6'*R*)-7-chloro-2',4,6-trimethoxy-5'-(5-methoxy-2-methyl-1H-indol-3-yl)-6'-methyl-3H-spiro[benzofuran-2,1'-cyclohexan]-2'-ene-3,4'-dione (6-36) was synthesized according to **general procedure C** and obtained as a light brown solid (1 mmol scale, 183.8 mg, 36% yield).

^{1}H NMR (400 MHz, DMSO-*d*$_6$, data for major diastereomer given) δ 10.70 (s, 1H), 7.16 (d, *J* = 8.6 Hz, 1H), 6.66 (d, *J* = 2.4 Hz, 1H), 6.57 (s, 1H), 6.52 (s, 1H), 5.84 (s, 1H), 4.17 (d, *J* = 12.5 Hz, 1H), 4.04 (s, 3H), 3.97 (s, 3H), 3.71 (d, *J* = 11.5 Hz, 6H), 3.18 (dd, *J* = 12.7, 6.7 Hz, 1H), 2.26 (s, 3H), 0.57 (d, *J* = 6.7 Hz, 3H).

^{13}C NMR (101 MHz, DMSO-*d*$_6$) δ 195.7, 191.6, 169.5, 168.6, 164.7, 157.7, 152.8, 135.4, 130.8, 126.5, 111.2, 109.2, 106.2, 104.9, 104.3, 101.3, 95.4, 91.3, 90.6, 57.6, 57.1, 56.6, 55.5, 45.0, 20.7, 12.5, 11.4.

HRMS-ESI (m/z): calcd for $C_{27}H_{27}ClNO_7$ [M + H$^+$] 512.1470, found 512.1474

FTIR (film, cm^{-1}): 3411, 2941, 1699, 1665, 1617, 1590, 1484, 1466, 1354, 1215 cm^{-1}

M.p.: 240 – 242 ℃

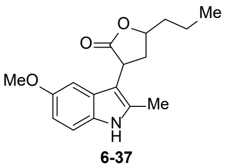

6-37

3-(5-methoxy-2-methyl-1*H*-indol-3-yl)-5-propyldihydrofuran-2(3*H*)-one (6-37) was synthesized

according to **general procedure C** and obtained as a brown solid (1 mmol scale, 255.3 mg, 89% yield, dr = 2.4:1).

^{1}H NMR (400 MHz, $CDCl_3$) **6-37-1 (anti)**: δ 8.06 (s, 1H), 7.09 (d, *J* = 8.6 Hz, 1H), 6.81 – 6.72 (m, 2H), 4.56 (ddd, *J* = 12.7, 10.9, 5.9 Hz, 1H), 4.01 (dd, *J* = 12.6, 9.0 Hz, 1H), 3.82 (s, 3H), 2.59 (ddd, *J* = 13.2, 9.0, 5.6 Hz, 1H), 2.20 – 2.13 (m, 1H), 2.12 (d, *J* = 1.2 Hz, 3H), 1.94 – 1.85 (m, 1H), 1.78 – 1.69 (m, 1H), 1.61 – 1.45 (m, 2H), 1.01 (t, *J* = 7.3 Hz, 3H); **6-37-2 (syn)**: δ 8.16 (s, 1H), 7.06 (d, *J* = 8.7 Hz, 1H), 6.80 (s, 1H), 6.75 (dd, *J* = 8.7, 2.2 Hz, 1H), 4.76 (tt, *J* = 8.2, 4.5 Hz, 1H), 4.02 (t, *J* = 9.5 Hz, 1H), 3.82 (s, 3H), 2.53 (dt, *J* = 13.2, 8.5 Hz, 1H), 2.33 – 2.24 (m, 1H), 2.08 (s, 3H), 1.93 – 1.76 (m, 1H), 1.72 – 1.42 (m, 3H), 1.01 (t, *J* = 7.2 Hz, 3H).

^{13}C NMR (101 MHz, $CDCl_3$) δ **6-37-1 (anti)**: 178.8, 154.0, 133.8, 130.7, 127.0, 111.5, 110.5, 106.7, 100.3, 79.1, 56.1, 37.8, 36.5, 35.2, 18.7, 13.9, 11.6.; **6-37-2 (syn)**: 178.2, 154.0, 134.0, 130.8, 127.1, 111.6, 110.7, 106.3, 100.5, 79.0, 56.1, 38.8, 37.8, 36.5, 18.6, 14.0, 11.7.

HRMS-API (m/z): calcd. for $C_{17}H_{22}NO_3$ [M + H$^+$] 288.1594, found 288.1595

FTIR (film, cm^{-1}): 3306, 2932, 1754, 1488, 1183 cm^{-1}

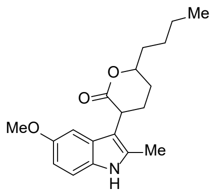

6-38 (two diastereomers)

6-butyl-3-(5-methoxy-2-methyl-1*H*-indol-3-yl)tetrahydro-2*H*-pyran-2-one (6-38) was synthesized according to **general procedure C** and obtained as a yellow oil (1 mmol scale, 175.1 mg, 56% yield, dr = 2.2:1).

^{1}H NMR (400 MHz, $CDCl_3$, data for major diastereomer given) δ 8.25 (s, 1H), 6.96 (dd, *J* = 8.7, 4.7 Hz, 1H), 6.77 (d, *J* = 2.4 Hz, 1H), 6.72 (d, *J* = 2.2 Hz, 1H), 4.61 – 4.46 (m, 1H), 3.82 (s, 3H), 3.76 (s, 1H), 2.16 – 2.00 (m, 3H), 1.94 (s, 3H), 1.87 – 1.62 (m, 3H), 1.56 (ddt, *J* = 12.1, 7.2, 3.8 Hz, 1H), 1.50 – 1.34 (m, 3H), 0.96 (t, *J* = 7.2, 1.9 Hz, 3H).

^{13}C NMR (101 MHz, $CDCl_3$) major δ = 173.2, 153.6, 133.4, 130.7, 126.8, 111.5, 109.8, 109.2, 100.6, 82.5, 56.0, 38.8, 36.0, 29.2, 28.0, 27.0, 22.5, 13.9, 11.3; minor δ = 174.2, 153.6, 133.2, 130.6, 127.3, 111.5, 110.2, 108.1, 100.6, 79.3, 55.9, 36.4, 35.2, 27.3, 27.0, 25.2, 22.5, 13.9, 11.6.

HRMS-API (m/z): calcd. for $C_{19}H_{26}NO_3$ [M + H$^+$] 316.1907, found 316.1905

FTIR (film, cm^{-1}): 3346, 2952, 2934, 2870, 1715, 1485, 1217, 1178, 1098, 1033, 954, 797 cm^{-1}

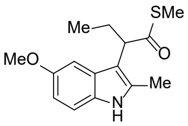

6-39

S-methyl 2-(5-methoxy-2-methyl-1*H*-indol-3-yl)butanethioate (**6-39**) was synthesized according to **general procedure C** and obtained as a light green oil (1 mmol scale, 183.3 mg, 66% yield).

^{1}H NMR (400 MHz, $CDCl_3$) δ 8.01 (s, 1H), 7.17 – 7.09 (m, 2H), 6.80 (dd, *J* = 8.7, 2.4 Hz, 1H), 3.87 (s, 3H), 3.84 (dd, *J* = 9.0, 6.4 Hz, 1H), 2.40 (s, 3H), 2.39 – 2.32 (m, 1H), 2.23 (s, 3H), 2.04 (ddt, *J* = 14.6, 8.9, 7.3 Hz, 1H), 0.91 (t, *J* = 7.4 Hz, 3H).

^{13}C NMR (101 MHz, $CDCl_3$) δ 201.6, 153.9, 134.1, 130.5, 127.9, 111.2, 110.7, 108.2, 101.7, 55.9, 53.3, 23.8, 12.3, 12.2, 11.8.

HRMS-ESI (m/z): calcd for $C_{15}H_{20}NO_2S$ [M + H$^+$] 278.1209, found 278.1211

FTIR (film, cm^{-1}): 3399, 2929, 1685, 1484, 1453, 1217, 1031, 996, 798 cm^{-1}

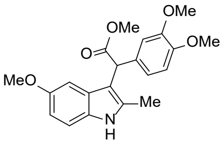

6-40

Methyl 2-(3,4-dimethoxyphenyl)-2-(5-methoxy-2-methyl-1*H*-indol-3-yl)acetate (**6-40**) was synthesized according to **general procedure C** and obtained as a brown solid (1 mmol scale, 213.3 mg, 58% yield).

^{1}H NMR (400 MHz, $CDCl_3$) δ 8.11 (s, 1H), 7.08 (d, *J* = 8.8 Hz, 1H), 6.97 (d, *J* = 2.5 Hz, 1H), 6.88 (d, *J* = 2.0 Hz, 1H), 6.83 (dd, *J* = 8.3, 2.0 Hz, 1H), 6.81 – 6.72 (m, 2H), 5.20 (s, 1H), 3.83 (s, 3H), 3.77 (d, *J* = 8.5 Hz, 6H), 3.72 (d, *J* = 2.5 Hz, 3H), 2.27 (s, 3H).

^{13}C NMR (101 MHz, $CDCl_3$) δ 173.9, 153.9, 148.7, 147.9, 133.8, 131.1, 130.3, 128.1, 120.5, 111.9, 111.1, 111.0, 110.8, 108.1, 101.5, 55.8, 54.4, 52.4, 52.1, 47.6, 12.1.

HRMS-API (m/z): calcd. for $C_{21}H_{22}NO_5$ [M - H$^+$] 368.1492, found 368.1502

FTIR (film, cm^{-1}): 3380, 2935, 1730, 1515, 1250, 1199, 1174, 1136, 1024 cm^{-1}

M.p.: 122 – 123 ℃

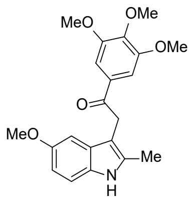

6-41

2-(5-methoxy-2-methyl-1*H*-indol-3-yl)-1-(3,4,5-trimethoxyphenyl)ethan-1-one (**6-41**) was synthesized according to **general procedure C** and obtained as a brown solid (1 mmol scale, 58.4 mg, 16% yield).

^{1}H NMR (400 MHz, $CDCl_3$) δ 7.95 (s, 1H), 7.30 (s, 2H), 7.10 (d, *J* = 8.7 Hz, 1H), 7.00 (d, *J* = 2.4 Hz, 1H), 6.75 (dd, *J* = 8.7, 2.4 Hz, 1H), 4.24 (s, 2H), 3.88 (s, 3H), 3.82 (s, 3H), 3.80 (s, 6H), 2.30 (s, 3H).

^{13}C NMR (101 MHz, $CDCl_3$) δ 196.8, 154.2, 153.0(x 2C), 142.3, 133.8, 131.9, 130.5, 128.9, 111.3, 110.7, 106.1(x 2C), 104.7, 100.5, 60.9, 56.3(x 2C), 56.0, 35.5, 12.1.

HRMS-ESI (m/z): calcd for $C_{21}H_{24}NO_5$ [M + H$^+$] 370.1648, found 370.1652

FTIR (film, cm^{-1}): 3424, 2940, 1673, 1587, 1483, 1456, 1413, 1332, 1212, 1153, 1127, 1030, 999 cm^{-1}

M.p.: 132 – 133 ℃

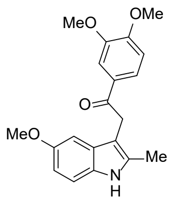

6-42

1-(3,4-dimethoxyphenyl)-2-(5-methoxy-2-methyl-1*H*-indol-3-yl)ethan-1-one (**6-42**) was synthesized according to **general procedure C** and obtained as a brown solid (1 mmol scale, 54.4 mg, 16% yield).

^{1}H NMR (400 MHz, $CDCl_3$) δ 7.92 (s, 1H), 7.73 (dt, *J* = 8.4, 1.6 Hz, 1H), 7.56 (t, *J* = 1.6 Hz, 1H), 7.08 (d, *J* = 8.7 Hz, 1H), 6.97 (d, *J* = 2.4 Hz, 1H), 6.85 (dd, *J* = 8.4, 1.2 Hz, 1H), 6.74 (dt, *J* = 8.7, 1.8 Hz, 1H), 4.24 (s, 2H), 3.91 (s, 3H), 3.83 (dd, *J* = 6.7, 1.2 Hz, 6H), 2.30 (s, 3H).

^{13}C NMR (101 MHz, $CDCl_3$) δ 196.7, 154.2, 153.2, 149.0, 133.6, 130.5, 130.1, 129.1, 123.1, 111.2, 110.8, 110.8, 110.1, 105.0, 100.6, 56.1, 56.0, 56.0, 35.0, 12.2.

HRMS-ESI (m/z): calcd for $C_{20}H_{22}NO_4$ [M + H$^+$] 340.1543, found 340.1546

FTIR (film, cm^{-1}): 3360, 1680, 1595, 1587, 1513, 1487, 1419, 1262, 1152, 1019 cm^{-1}

M.p.: 152- 153 ℃

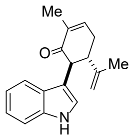

6-43

(5R,6R)-6-(1H-indol-3-yl)-2-methyl-5-(prop-1-en-2-yl)cyclohex-2-en-1-one (**6-43**) (Baran et al., 2004) was synthesized according to **general procedure C** and obtained as a white solid (1 mmol scale, 78.7 mg, 30% yield).

^{1}H NMR (400 MHz, $CDCl_3$) δ 8.30 (s, 1H), 7.46 (d, *J* = 7.8 Hz, 1H), 7.19 (d, *J* = 7.9 Hz, 1H), 7.14 (t, *J* = 7.4 Hz, 1H), 7.08 (t, *J* = 7.3 Hz, 1H), 6.84 (s, 1H), 6.52 (d, *J* = 2.0 Hz, 1H), 4.65 (d, *J* = 15.7 Hz, 2H), 3.92 (d, *J* = 10.8 Hz, 1H), 3.27 (td, *J* = 9.7, 5.1 Hz, 1H), 2.64 – 2.41 (m, 2H), 1.93 (s, 3H), 1.62 (s, 3H).

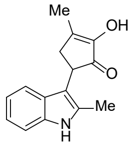

6-48

2-hydroxy-5-methyl-3-(2-methyl-1H-indol-3-yl)cyclopent-2-en-1-one (6-48) was synthesized according to **general procedure C** and obtained as a light brown solid (1 mmol scale, 21.0 mg, 9% yield).

^{1}H NMR (400 MHz, DMSO-*d*$_6$) δ 10.80 (s, 1H), 8.98 (s, 1H), 7.23 (dd, *J* = 7.7, 1.4 Hz, 1H), 7.02 – 6.92 (m, 2H), 6.86 – 6.80 (m, 1H), 3.74 (dd, *J* = 7.0, 2.4 Hz, 1H), 2.94 – 2.79 (m, 1H), 2.46 (d, *J* = 17.7 Hz, 1H), 2.29 (s, 3H), 1.99 (s, 3H).

^{13}C NMR (101 MHz, DMSO-*d*$_6$) δ 203.2, 149.2, 142.5, 135.2, 132.8, 126.8, 120.0, 118.2, 117.2, 110.6, 108.7, 40.0, 35.8, 14.2, 11.4.

HRMS-API (m/z): calcd. for $C_{15}H_{16}NO_2$ [M + H$^+$] 242.1175, found 242.1177

FTIR (film, cm^{-1}): 3423, 3360, 1693, 1642, 1462, 1402, 1203, 1096, 745 cm^{-1}

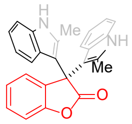

9-1

3,3-bis(2-methyl-1*H*-indol-3-yl)benzofuran-2(3*H*)-one (**9-1**) was synthesized according to **general procedure C** and obtained as a yellow solid (1 mmol scale, 87.8 mg, 23% yield).

^{1}H NMR (400 MHz, DMSO-*d*$_6$) δ 11.08 (d, *J* = 12.0 Hz, 2H), 7.46 – 7.35 (m, 2H), 7.28 (t, *J* = 7.5 Hz, 3H), 7.15 (td, *J* = 7.2, 1.5 Hz, 1H), 6.97 – 6.90 (m, 2H), 6.75 – 6.64 (m, 3H), 6.53 (d, *J* = 8.1 Hz, 1H), 2.01 (s, 3H), 1.94 (s, 3H).

^{13}C NMR (101 MHz, DMSO-*d*$_6$) δ 176.8, 151.8, 135.0, 135.0, 134.0, 132.8, 132.8, 129.2, 126.8, 126.6, 126.1, 124.4, 120.3, 120.3, 118.8, 118.7, 118.6, 118.5, 110.9, 110.8 (x 2C), 108.6, 107.7, 50.5, 13.0, 12.9.

HRMS-ESI (m/z): calcd for $C_{26}H_{20}N_2O_2Na$ [M + Na$^+$] 415.1416, found 415.1420

FTIR (film, cm^{-1}): 3245, 3222, 2921, 1801, 1460, 1050, 1008, 755, 739 cm^{-1}

M.p.: 128 – 130 ℃

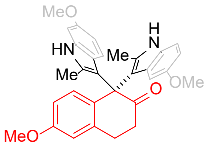

9-2

6-methoxy-1-(5-methoxy-2-methyl-1*H*-indol-3-yl)-1-(6-methoxy-2-methyl-1*H*-indol-3-yl)-3,4-dihydronaphthalen-2(1*H*)-one (**9-2**) was synthesized according to **general procedure C** and obtained as a light brown solid (1 mmol scale, 141.4 mg, 29% yield).

^{1}H NMR (400 MHz, DMSO-*d*$_6$) δ 10.92 (s, 1H), 10.89 (s, 1H), 7.24 (d, *J* = 8.3 Hz, 1H), 7.16 (dd, *J* = 10.5, 8.7 Hz, 2H), 6.88 (dd, *J* = 8.3, 2.7 Hz, 1H), 6.55 (ddd, *J* = 18.7, 8.7, 2.4 Hz, 2H), 6.35 (d, *J* = 2.7 Hz, 1H), 5.44 (d, 1H), 5.31 (d, 1H), 3.53 (s, 3H), 3.33 (s, 3H), 3.22 (s, 3H), 2.79 (t, *J* = 6.7 Hz, 2H), 2.73 – 2.53 (m, 2H), 1.80 (s, 3H), 1.71 (s, 3H).

^{13}C NMR (101 MHz, DMSO-*d*$_6$) δ 202.7, 158.0, 152.4, 152.4, 142.1, 136.1, 135.3, 130.6, 130.2, 130.2, 129.2, 128.9, 128.6, 116.5, 111.3, 110.8, 110.7, 109.3, 108.9, 107.9, 106.5, 102.0, 101.5, 58.6, 54.9, 54.7, 54.4, 36.2, 26.8, 13.9, 13.2.

HRMS-API (m/z): calcd. for $C_{21}H_{22}NO_5$ [M - H$^+$] 493.2121, found 493.2130

FTIR (film, cm^{-1}): 3402, 2926, 1707, 1579, 1485, 1445, 1212, 1026, 796 cm^{-1}

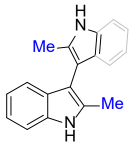

7-1

2,2'-dimethyl-1*H*,1'*H*-3,3'-biindole (7-1) (Greci et al., 2003) was synthesized according to **general procedure C** without adding carbonyl substrates and obtained as a light brown solid (2 mmol scale, 32.8 mg, 13% yield).

^{1}H NMR (400 MHz, DMSO-d_6) δ 11.01 (s, 2H), 7.33 (d, J = 8.0 Hz, 2H), 7.12 (d, J = 7.8 Hz, 2H), 7.02 (t, J = 7.5 Hz, 2H), 6.90 (t, J = 7.4 Hz, 2H), 2.27 (s, 6H).

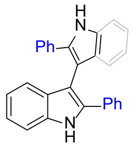

7-2

2,2'-diphenyl-1*H*,1'*H*-3,3'-biindole (7-2) (Niu et al., 2010) was synthesized according to **general procedure C** was obtained as a light brown solid (2 mmol scale, 217.6 mg, 57% yield).

^{1}H NMR (400 MHz, DMSO-d_6) δ 11.63 (s, 2H), 7.61 (d, J = 7.6 Hz, 4H), 7.54 (d, J = 8.1 Hz, 2H), 7.21 (t, J = 7.6 Hz, 4H), 7.15-7.10 (m, 4H), 7.02 (d, J = 7.9 Hz, 2H), 6.87 (t, J = 7.4 Hz, 2H).

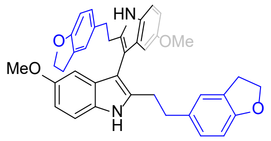

7-3

2,2'-bis(2-(2,3-dihydrobenzofuran-5-yl)ethyl)-5,5'-dimethoxy-1*H*,1'*H*-3,3'-biindole (**7-3**) was synthesized according to **general procedure C** and obtained as a brown solid (0.18 mmol scale, 22.2 mg, 41% yield).

^{1}H NMR (400 MHz, $CDCl_3$) δ 7.82 (s, 2H), 7.21 (d, J = 8.7 Hz, 2H), 6.89 (s, 2H), 6.82 (d, J = 8.3 Hz, 4H), 6.77 (s, 2H), 6.65 (d, J = 8.1 Hz, 2H), 4.52 (t, J = 8.7 Hz, 4H), 3.72 (s, 6H), 3.08 (t, J = 8.4 Hz, 4H), 2.93 (d, J = 7.3 Hz, 4H), 2.83 (d, J = 7.3 Hz, 4H).

^{13}C NMR (101 MHz, $CDCl_3$) δ 158.6(x 2C), 154.2(x 2C), 137.8(x 2C), 133.2(x 2C), 130.6(x 2C), 130.0(x 2C), 127.9(x 2C), 127.3(x 2C), 125.0(x 2C), 111.3(x 2C), 111.1(x 2C), 109.1(x 2C), 106.5(x 2C), 101.6(x

2C), 71.3(x 2C), 56.0(x 2C), 35.2(x 2C), 29.8(x 2C), 29.1(x 2C).

HRMS-API (m/z): calcd. for $C_{38}H_{35}N_2O_4$ [M - H^+] 583.2591, found 583.2595

FTIR (film, cm^{-1}): 3400, 2927, 1488, 1449, 1216, 981, 800 cm^{-1}

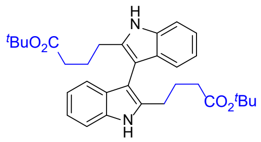

7-4

di-*tert*-butyl 4,4'-(1*H*,1'*H*-[3,3'-biindole]-2,2'-diyl)dibutyrate (7-4) was synthesized according to **general procedure C** and obtained as yellow oil (0.5 mmol scale, 24.0 mg, 19% yield) by utilizing LiHMDS (1.25 mmol, 2.5 equivalent) and $FeCl_3$ (201 mg, 1.25 mmol, 2.5 equivalent).

^{1}H NMR (400 MHz, $CDCl_3$) δ 8.51 (s, 2H), 7.39 (dt, *J* = 8.0, 0.9 Hz, 2H), 7.31 – 7.27 (m, 2H), 7.17 (ddd, *J* = 8.1, 7.0, 1.2 Hz, 2H), 7.04 (ddd, *J* = 8.0, 7.1, 1.0 Hz, 2H), 2.72 (t, *J* = 7.4 Hz, 4H), 2.19 (t, *J* = 7.2 Hz, 4H), 1.88 (p, *J* = 7.3 Hz, 4H), 1.40 (s, 18H).

^{13}C NMR (101 MHz, $CDCl_3$) δ 173.3(x 2C), 136.6(x 2C), 135.7(x 2C), 129.6(x 2C), 121.2(x 2C), 119.6(x 2C), 119.4(x 2C), 110.5(x 2C), 106.5(x 2C), 80.6(x 2C), 34.8(x 2C), 28.1(x 6C), 25.8(x 2C), 25.1(x 2C).

HRMS-API (m/z): calcd. for $C_{32}H_{41}N_2O_4^+$ [M + H^+] 517.3061, found 517.3059

FTIR (film, cm^{-1}): 3394, 2975, 2931, 1724, 1704, 1153, 741 cm^{-1}

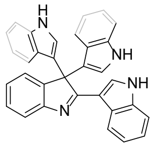

10-1

3'-(1*H*-indol-3-yl)-1*H*, 1''*H*, 3'*H*-3, 2': 3', 3''-terindole (**10-1**) (Richter et al., 2007) was isolated as a light brown solid (2 mmol scale, 81.2 mg, 35% yield).

^{1}H NMR (400 MHz, DMSO-*d*$_6$) δ 11.46 (d, *J* = 3.0 Hz, 1H), 11.06 (d, *J* = 2.6 Hz, 2H), 8.98 – 8.76 (m, 1H), 7.80 (d, *J* = 7.6 Hz, 1H), 7.67 (d, *J* = 3.0 Hz, 1H), 7.58 (d, *J* = 7.4 Hz, 1H), 7.43 (dd, *J* = 10.9, 7.9 Hz, 3H), 7.38 – 7.21 (m, 5H), 7.13 – 6.98 (m, 5H), 6.84 (t, *J* = 7.5 Hz, 2H).

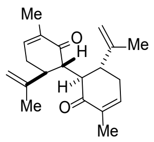

8-1

(1R,1'*S*,6*S*,6'*R*)-3,3'-dimethyl-6,6'-di(prop-1-en-2-yl)-[1,1'-bi(cyclohexane)]-3,3'-diene-2,2'-dione (**8-1**) (Bailey et al., 2018) was isolated as byproduct during the condition optimization, yield not determined.

^{1}H NMR (400 MHz, $CDCl_3$) δ 6.64 – 6.59 (m, 2H), 4.79 (dt, *J* = 19.7, 2.2 Hz, 4H), 3.43 (td, *J* = 12.2, 4.2 Hz, 2H), 2.46 (dd, *J* = 12.6, 3.0 Hz, 2H), 2.38 – 2.14 (m, 4H), 1.69 (d, *J* = 2.8 Hz, 6H), 1.59 (d, *J* = 3.1 Hz, 6H).

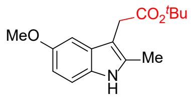

6-46

***tert*-butyl 2-(5-methoxy-2-methyl-1*H*-indol-3-yl)acetate (6-46)** was obtained as a light brown solid (0.5 mmol scale, 84.3 mg, 61% yield) according to **general procedure C**.

^{1}H NMR (400 MHz, $CDCl_3$) δ 7.85 (s, 1H), 7.08 (d, *J* = 8.7 Hz, 1H), 7.01 (d, *J* = 2.5 Hz, 1H), 6.76 (dd, *J* = 8.7, 2.4 Hz, 1H), 3.86 (s, 3H), 3.56 (s, 2H), 2.32 (s, 3H), 1.44 (s, 9H).

^{13}C NMR (101 MHz, $CDCl_3$) δ 171.7, 154.0, 133.6, 130.4, 129.1, 111.0, 110.9, 105.1, 100.6, 80.6, 56.0, 32.0, 28.2(x 3C), 11.8.

HRMS-API (m/z): calcd. for $C_{16}H_{22}NO_3$ [M + H^{+}] 276.1594, found 276.1595

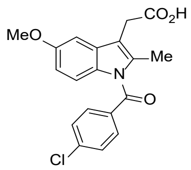

12-2, indomethacin

2-(1-(4-chlorobenzoyl)-5-methoxy-2-methyl-1*H*-indol-3-yl)acetic acid (**12-2, Indomethacin**) (Kasaya et al., 2009) was synthesized according to **general procedure D** and obtained as a white solid (0.052 mmol scale, 18.4 mg, 99% yield).

^{1}H NMR (400 MHz, $CDCl_3$) δ 7.70 – 7.61 (m, 2H), 7.47 (d, *J* = 8.4 Hz, 2H), 6.95 (d, *J* = 2.5 Hz, 1H), 6.85 (d, *J* = 9.0 Hz, 1H), 6.67 (dd, *J* = 9.0, 2.5 Hz, 1H), 3.83 (s, 3H), 3.69 (s, 2H), 2.39 (s, 3H).

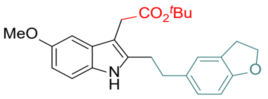

6-47

tert-butyl 2-(2-(2-(2,3-dihydrobenzofuran-5-yl)ethyl)-5-methoxy-1*H*-indol-3-yl)acetate (6-47) was synthesized according to **general procedure D** and obtained as a yellow solid (0.5 mmol scale, 102.2 mg, 50% yield)

^{1}H NMR (400 MHz, $CDCl_3$) δ 7.76 (s, 1H), 7.12 – 7.03 (m, 2H), 6.97 (d, *J* = 1.8 Hz, 1H), 6.88 (dd, *J* = 8.1, 1.9 Hz, 1H), 6.77 (dd, *J* = 8.7, 2.5 Hz, 1H), 6.71 (d, *J* = 8.1 Hz, 1H), 4.56 (t, *J* = 8.7 Hz, 2H), 3.87 (s, 3H), 3.54 (s, 2H), 3.15 (t, *J* = 8.6 Hz, 2H), 2.97 (t, *J* = 7.1 Hz, 2H), 2.87 (t, *J* = 7.4 Hz, 2H), 1.45 (s, 9H).

^{13}C NMR (101 MHz, $CDCl_3$) δ 171.6, 158.6, 154.0, 137.3, 133.3, 130.3, 128.9, 127.9, 127.2, 125.1, 111.1, 109.1, 105.0, 100.7, 80.6, 71.3, 55.9, 35.6, 32.0, 29.8, 28.9, 28.2, 28.2 (x 3C).

HRMS-API (m/z): calcd. for $C_{25}H_{30}NO_4$ [M + H$^+$] 408.2169, found 408.2169

FTIR (film, cm^{-1}): 3379, 2965, 1718, 1485, 1215, 1150, 829 cm^{-1}

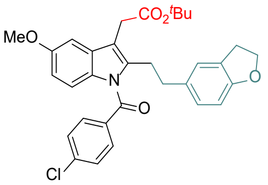

12-3

tert-butyl 2-(1-(4-chlorobenzoyl)-2-(2-(2,3-dihydrobenzofuran-5-yl)ethyl)-5-methoxy-1*H*-indol-3-yl)acetate (12-3) was synthesized according to **general procedure D** and obtained as a yellow oil (0.158 mmol scale, 69.9 mg, 81% yield)

^{1}H NMR (400 MHz, $CDCl_3$) δ 7.63 – 7.54 (m, 2H), 7.51 – 7.41 (m, 2H), 7.00 (dd, *J* = 4.5, 2.1 Hz, 2H), 6.86 (dd, *J* = 8.0, 1.9 Hz, 1H), 6.67 – 6.58 (m, 2H), 6.55 (d, *J* = 9.0 Hz, 1H), 4.48 (t, *J* = 8.7 Hz, 2H), 3.83 (s, 3H), 3.54 (s, 2H), 3.23 (dd, *J* = 9.4, 6.3 Hz, 2H), 3.06 (t, *J* = 8.7 Hz, 2H), 2.84 (dd, *J* = 9.1, 6.5 Hz, 2H), 1.47 (s, 9H).

^{13}C NMR (101 MHz, $CDCl_3$) δ 170.3, 168.4, 158.5, 155.9, 140.0, 139.5, 133.7, 133.1, 131.4 (x 2C), 131.0, 130.7, 129.1 (x 2C), 128.1, 127.2, 125.1, 114.9, 113.6, 111.8, 109.0, 101.6, 81.2, 71.2, 55.7, 35.9, 31.8, 29.8, 28.6, 28.2 (x 3C).

HRMS-ESI (m/z): calcd. for $C_{32}H_{33}ClNO_5$ [M + H$^+$] 546.2041, found 546.2033

FTIR (film, cm^{-1}): 2975, 2930, 1730, 1683, 1590, 1491, 1478 cm^{-1}

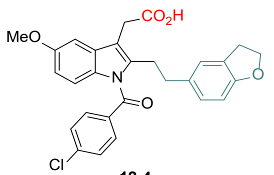

12-4

2-(1-(4-chlorobenzoyl)-2-(2-(2,3-dihydrobenzofuran-5-yl)ethyl)-5-methoxy-1*H*-indol-3-yl)acetic acid (12-4) was synthesized according to **general procedure D** and obtained as a white solid (0.037 mmol scale, 17.5 mg, 96% yield)

^{1}H NMR (400 MHz, $CDCl_3$) δ 7.60 (d, *J* = 8.2 Hz, 2H), 7.45 (d, *J* = 8.2 Hz, 2H), 6.95 (d, *J* = 3.0 Hz, 2H), 6.80 (d, *J* = 8.0 Hz, 1H), 6.61 (td, *J* = 6.9, 3.1 Hz, 2H), 6.53 (d, *J* = 9.0 Hz, 1H), 4.47 (t, *J* = 8.6 Hz, 2H), 3.80 (s, 3H), 3.61 (s, 2H), 3.23 (t, *J* = 7.7 Hz, 2H), 3.04 (t, *J* = 8.6 Hz, 2H), 2.81 (t, *J* = 7.7 Hz, 2H).

^{13}C NMR (101 MHz, $CDCl_3$) δ 176.7, 168.5, 158.6, 156.0, 140.4, 139.7, 133.6, 132.9, 131.5(x 2C), 131.0, 130.4, 129.2(x 2C), 128.1, 127.3, 125.1, 115.0, 112.3, 111.9, 109.1, 101.6, 71.2, 55.8, 35.7, 30.1, 29.8, 28.7.

HRMS-API (m/z): calcd. for $C_{32}H_{33}ClNO_5$ [M + H^+] 546.2041, found 546.2033

FTIR (film, cm^{-1}): 3079, 2946, 1698, 1679, 1491, 1476, 1218 cm^{-1}

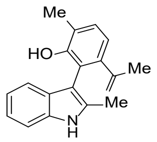

13-1

6-methyl-2-(2-methyl-1H-indol-3-yl)-3-(prop-1-en-2-yl)phenol (**13-1**) was obtained as sticky oil (0.1 mmol scale, 14.7 mg, 54% yield, 63% brsm, 3% ee) according to Table **ST1**. The enatioselectivitity was determined by Chiral HPLC analysis on CHIRALPAK® AD-H column, temperature 35℃, flow n-Hexaen/^{i}PrOH=90:10(v/v), 1mL/1min, detected by UV254 nm, t_{R1} = 5.45 min, t_{R2} = 15.7 min.

^{1}H NMR (400 MHz, $CDCl_3$) δ 8.16 (s, 1H), 7.34 (d, *J* = 8.0 Hz, 1H), 7.28 – 7.22 (m, 1H), 7.20 – 7.11 (m, 2H), 7.07 (t, *J* = 7.4 Hz, 1H), 6.84 (d, *J* = 7.7 Hz, 1H), 5.28 (s, 1H), 4.82 (d, *J* = 1.4 Hz, 2H), 2.30 (s, 3H), 2.23 (s, 3H), 1.53 (s, 3H).

^{13}C NMR (101 MHz, $CDCl_3$) δ 152.7, 146.6, 144.2, 135.7, 134.6, 130.2, 128.4, 122.6, 122.0, 120.3, 119.9,

119.4, 117.2, 114.3, 110.5, 106.6, 23.1, 16.3, 12.6.

HRMS-API (m/z): calcd. for $C_{19}H_{18}NO$ [M - H^+] 276.1382, found 276.1393

FTIR (film, cm^{-1}): 3468, 3399, 2918, 1458, 1307, 1249, 1222, 1196, 1070, 1013, 894, 818, 744 cm^{-1}

Reference

Bailey, S. J., Sapkota, R. R., Golliher, A. E., Dungan, B., Talipov, M., Holguin, F. O. and Maio, W. A. (2018). Lewis-Acid-Mediated Union of Epoxy-Carvone Diastereomers with Anisole Derivatives: Mechanistic Insight and Application to the Synthesis of Non-natural CBD Analogues. Org. Lett. *20*, 4618-4621.

Baran, P. S. and Richter, J. M. (2004). Direct Coupling of Indoles with Carbonyl Compounds: Short, Enantioselective, Gram-Scale Synthetic Entry into the Hapalindole and Fischerindole Alkaloid Families. J. Am. Chem. Soc. *126*, 7450- (2004).

Chen, J., Li, C.-M., Wang, J., Ahn, S., Wang, Z., Lu, Y., Dalton, J. T., Miller, D. D. and Li, W. (2011). Synthesis and antiproliferative activity of novel 2-aryl-4-benzoyl-imidazole derivatives targeting tubulin polymerization. Biorg. Med. Chem. *19*, 4782-4795.

Greci, L., Castagna, R., Carloni, P., Stipa, P., Rizzoli, C., Righi, L. and Sgarabotto, P. (2003). Nitrenium ions. Reactions of N,N-dimethyl-p-benzoyloxyaniline-iminium chloride with indoles and indolizines. X-ray structure of unexpected [2-chloro-4-(4-dimethylaminophenyl-ONN-azoxy)phenyl]dimethylamine (azoxy derivative). Org. Biomol. Chem. *1*, 3768-3771

Kasaya, Y., Hoshi, K., Terada, Y., Nishida, A., Shuto, S. and Arisawa, M. (2009). Aromatic Enamide/Ene Metathesis toward Substituted Indoles and Its Application to the Synthesis of Indomethacins. Eur. J. Org. Chem. 4606-4613.

Maksymenko, S., Parida, K. N., Pathe, G. K., More, A. A., Lipisa, Y. B. and Szpilman, A. M. (2017). Transition-Metal-Free Intermolecular α-Arylation of Ketones via Enolonium Species. Org. Lett. *19*, 6312-6315.

Miyata, O.; Takeda, N. and Naito, T. (2002) Synthesis of 2, 4-disubstituted indoles via thermal cyclization of N-trifluoroacetyl enehydrazines. Heterocycles. *57*, 1101-1107.

Molander, G. A. Canturk, B. and Kennedy, L. E. (2009). Scope of the Suzuki−Miyaura Cross-Coupling Reactions of Potassium Heteroaryltrifluoroborates. J. Org. Chem. *74*, 973-980.

Niu, T. and Zhang, Y. (2010). Iron-catalyzed oxidative homo-coupling of indoles via C–H cleavage. Tetrahedron Lett. *51*, 6847-6851.

Richter, J. M. W., B. W., Maimone, T. J., Lin, D. W., Castroviejo, M. P. and Baran, P. S (2007). Scope and mechanism of direct indole and pyrrole couplings adjacent to carbonyl compounds: total synthesis of acremoauxin A and oxazinin 3. J. Am. Chem. Soc. *129*, 12857-12869.

Stempel, E.; Kaml, R. F.-X.; Budisa, N. and Kalesse, M. (2018) Painting argyrins blue: Negishi cross-coupling for synthesis of deep-blue tryptophan analogue β-(1-azulenyl)-l alanine and its incorporation into argyrin C. Biorg. Med. Chem. *26*, 5259-5269.

Trabbic, C. J., George, S. M., Alexander, E. M., Du, S.; Offenbacher, J. M., Crissman, E. J., Overmeyer, J. H & Maltese, W. A and Erhardt, P. W. (2016). Synthesis and biological evaluation of isomeric methoxy substitutions on anti-cancer indolyl-pyridinyl-propenones: Effects on potency and mode of activity. Eur. J. Med. Chem.*122*, 79-91.

Yamagishi, M., Nishigai, K., Ishii, A., Hata, T. and Urabe, H. (2012). Facile Preparation of Indoles and 1,2-Benzothiazine 1,1-Dioxides: Nucleophilic Addition of Sulfonamides to Bromoacetylenes and Subsequent Palladium-Catalyzed Cyclization. Angew. Chem. Int. Ed. *51*, 6471-6474.

Yang, S.-D., Sun, C.-L., Fang, Z., Li, B.-J., Li, Y.-Z. and Shi, Z.-J. (2008). Palladium-Catalyzed Direct Arylation of (Hetero)Arenes with Aryl Boronic Acids. Angew. Chem. Int. Ed. *47*, 1473-1476.

Wetzel, A., Pratsch, G., Kolb, R. and Heinrich, M. R. (2016). Radical Arylation of Phenols, Phenyl Ethers, and Furans. Chem. Eur. J. *16*, 2547-2556.

Zuo, Z., Xie, W. and Ma, D. (2010) Total Synthesis and Absolute Stereochemical Assignment of (−)-Communesin F. J. Am. Chem. Soc. *132*, 13226-13228.

III. Spectrum

2018-1.3250.fid
LHL-1-53-1

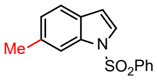

14-1

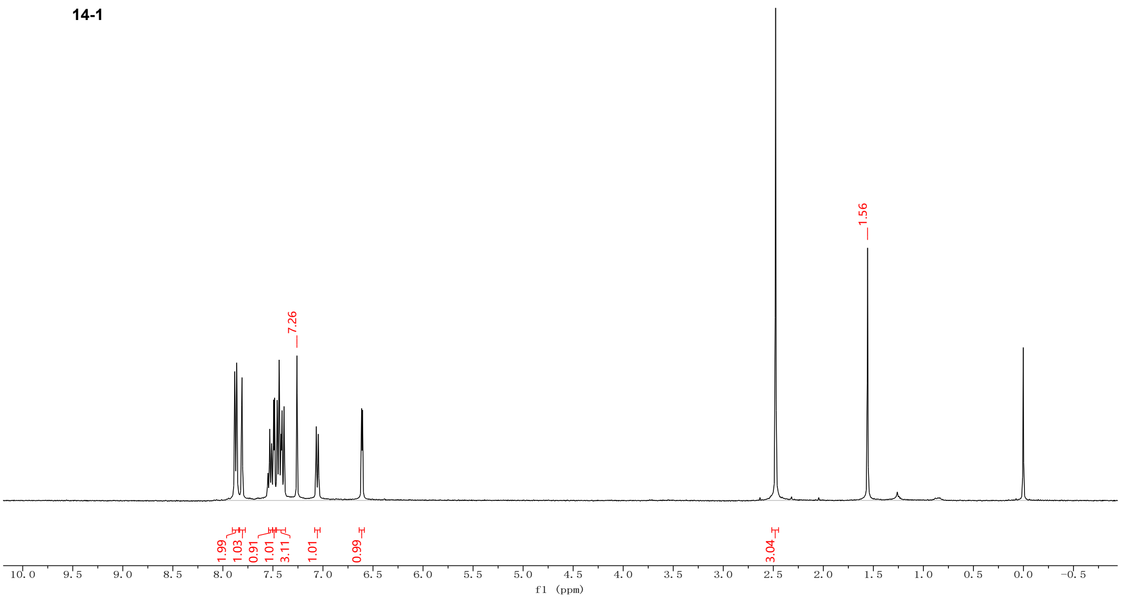

2018-1.3417.fid
LHL1-54-1

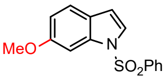

14-2

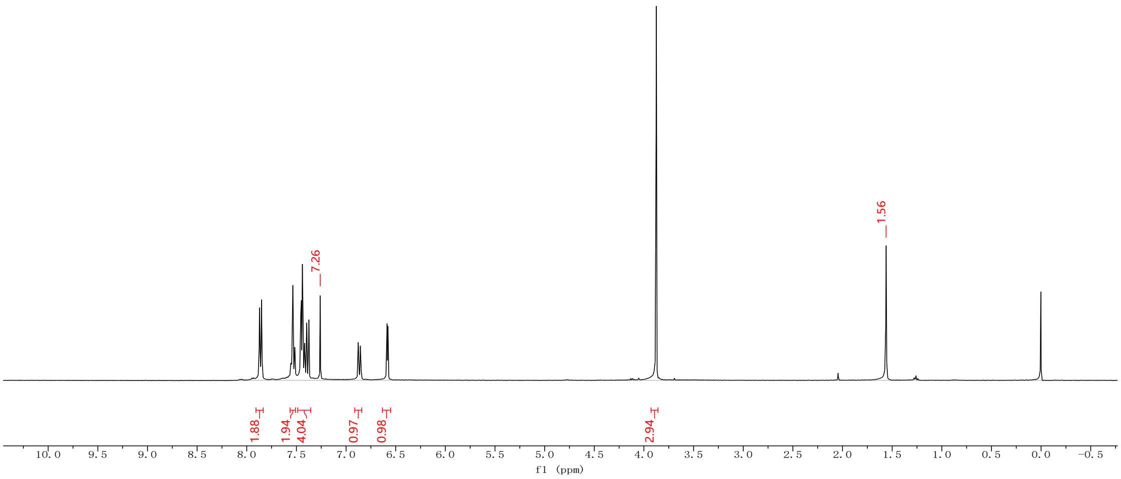

2018-1.3249.fid
LHL-1-52-2

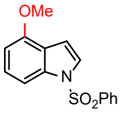

14-3

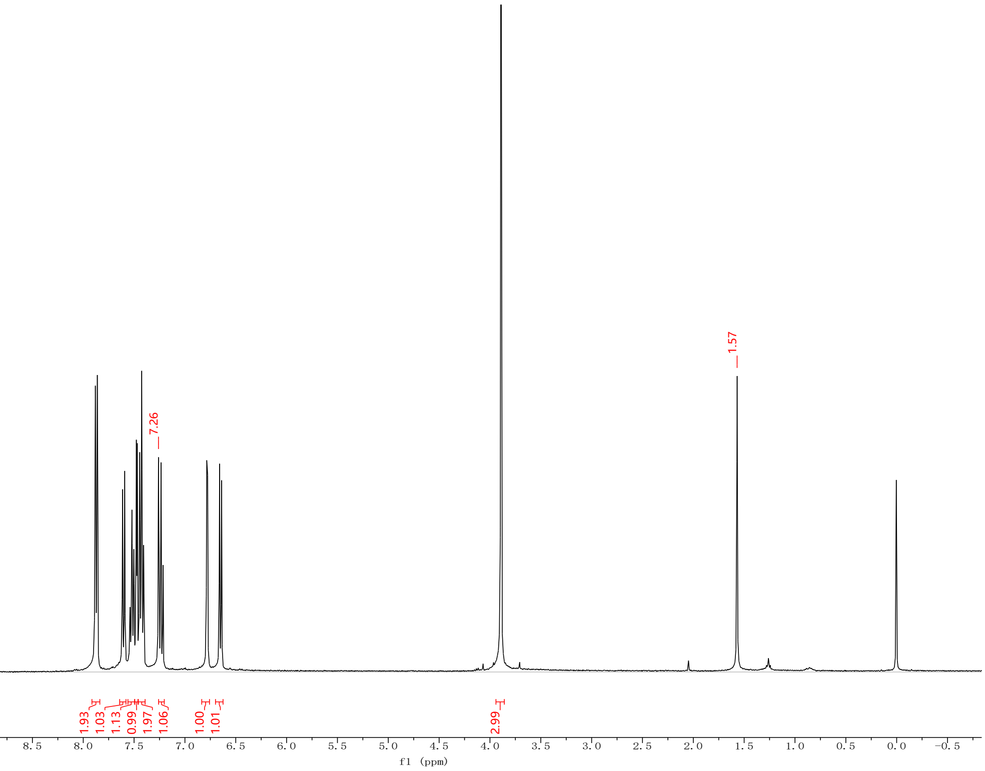

2018-2.7772.fid
LHL2-31-1

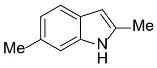

1-7

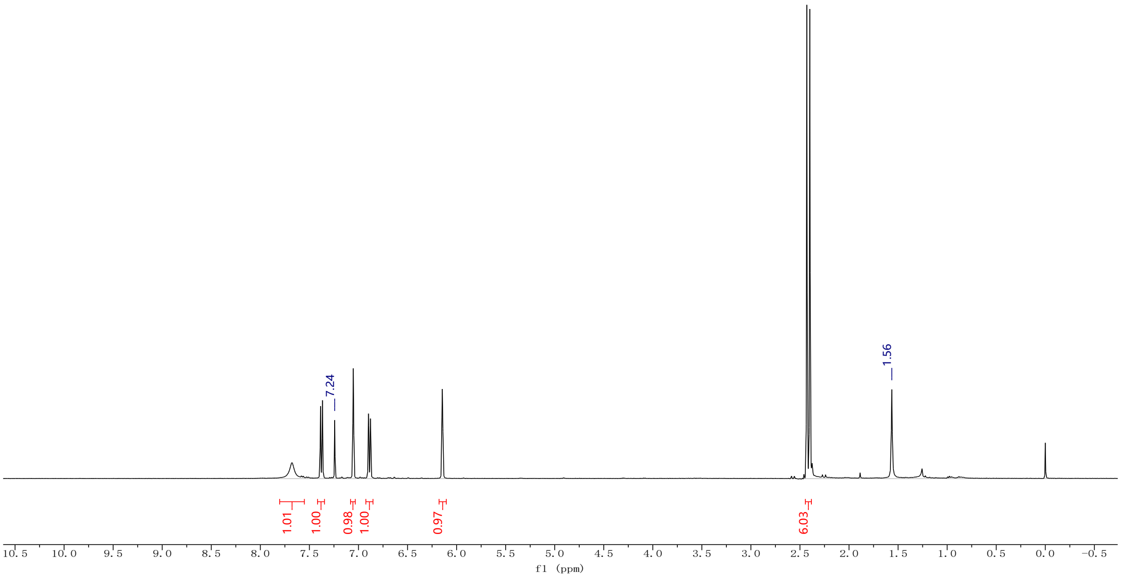

2018-2.7770.fid
LHL2-31-2

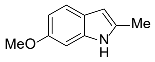

1-8

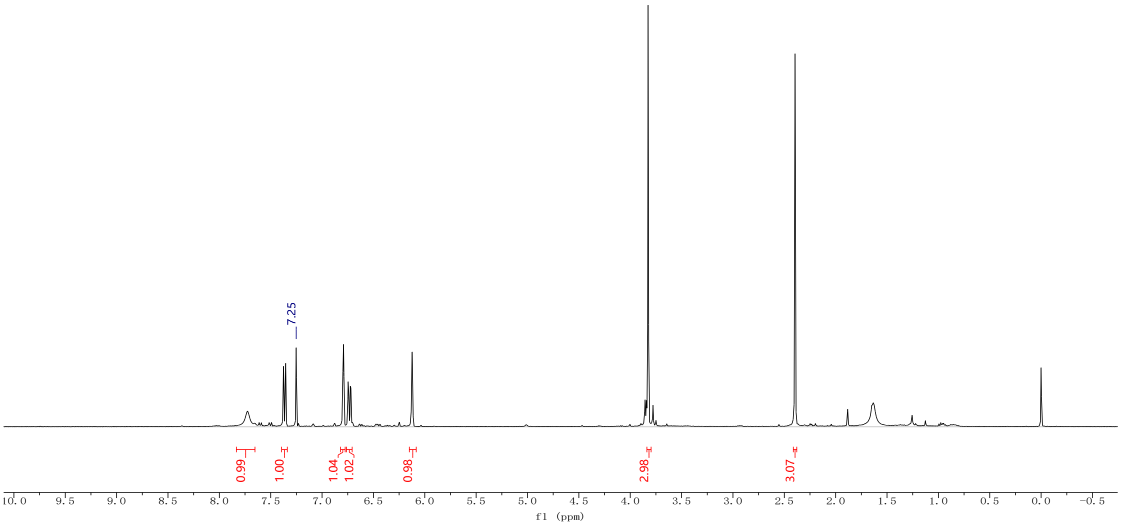

2018-2.9375.fid
LHL-2-38-1

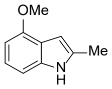

1-9

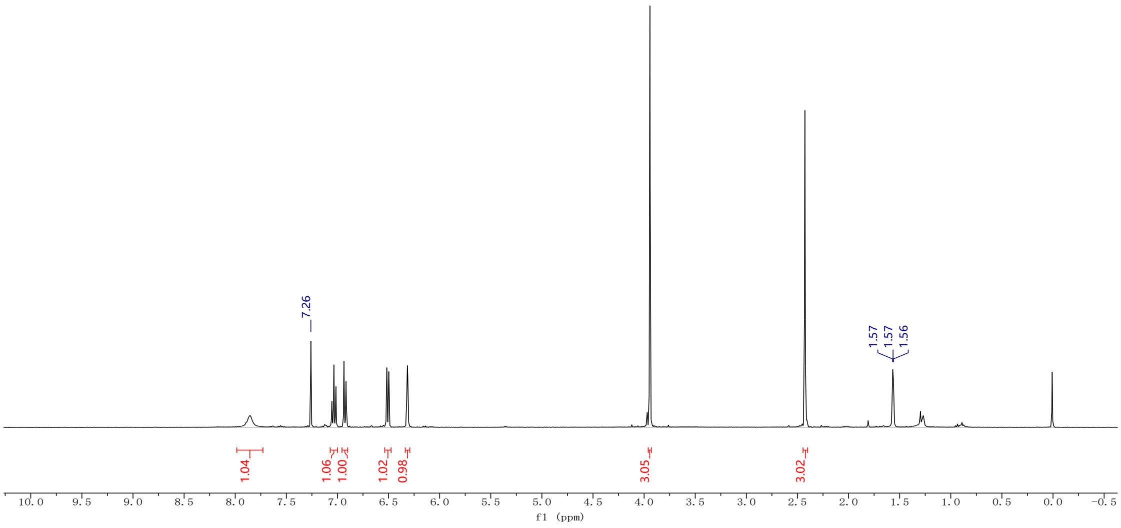

2018-2.11985.fid [illegible]
LHL-2-43-1

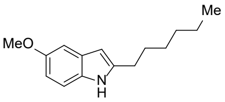

1-13

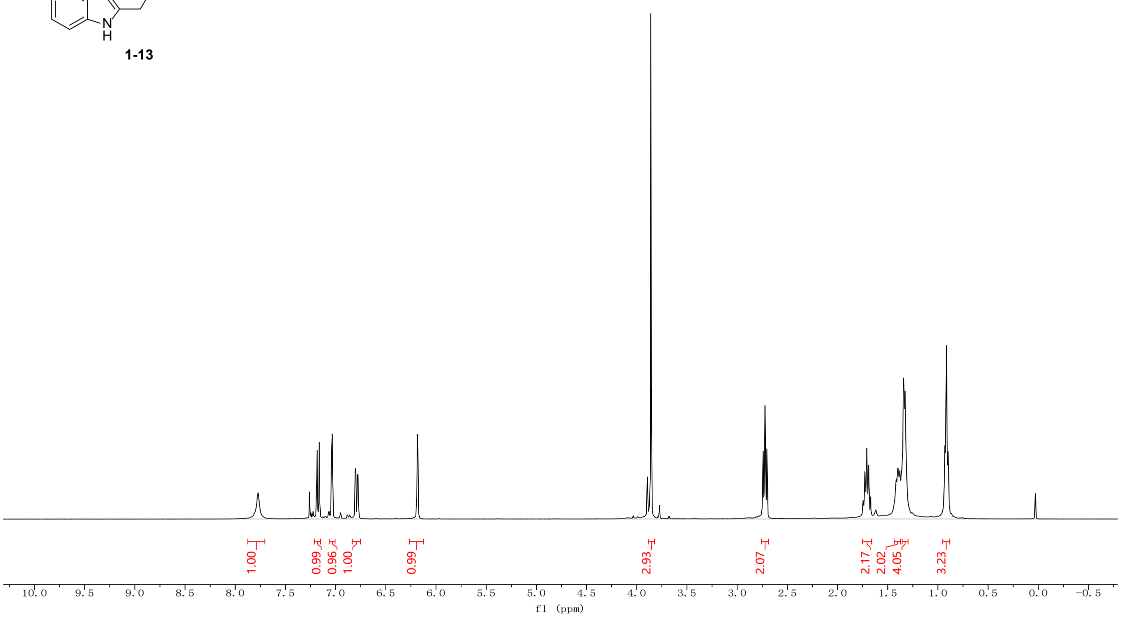

2018-2.11634.fid [illegible]
LHL-2-60-2

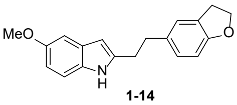

1-14

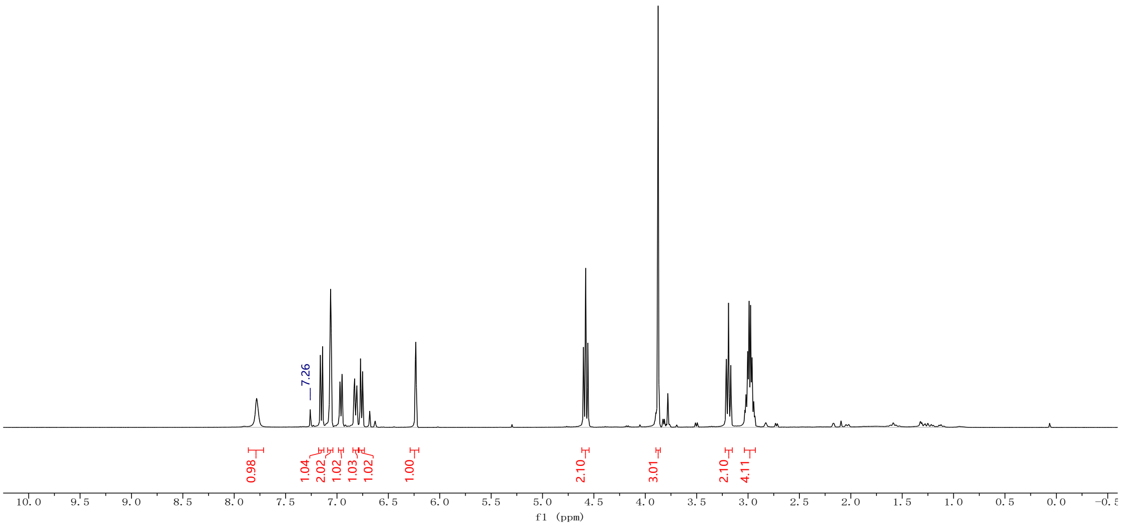

2018-2.12022.fid
LHL-2-60-2

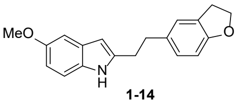

1-14

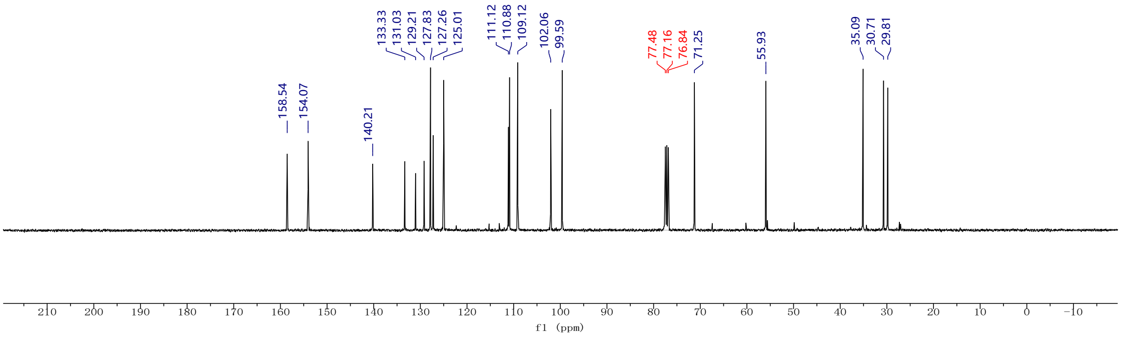

2018-2.7768.fid
LHL1-51-2

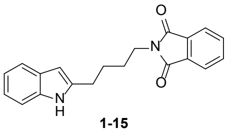

1-15

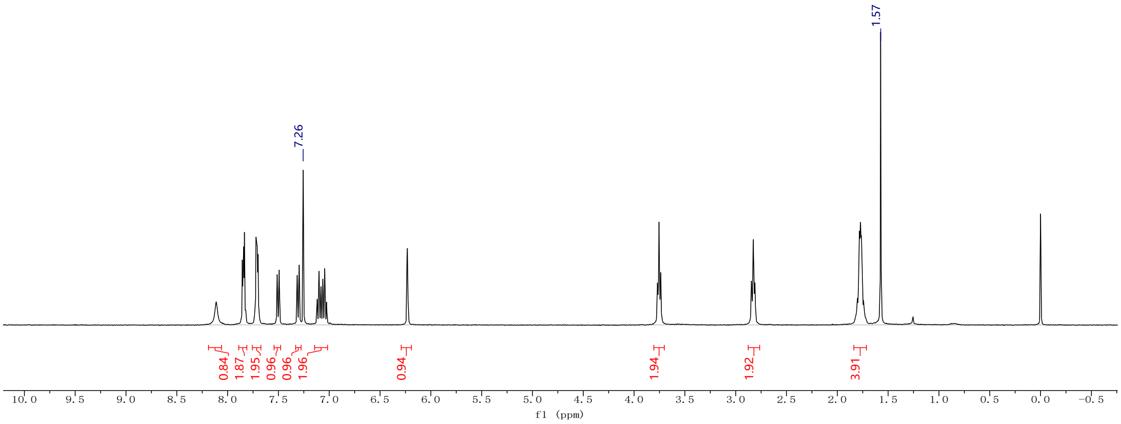

2018-2.8623.fid
LHL-1-51-2

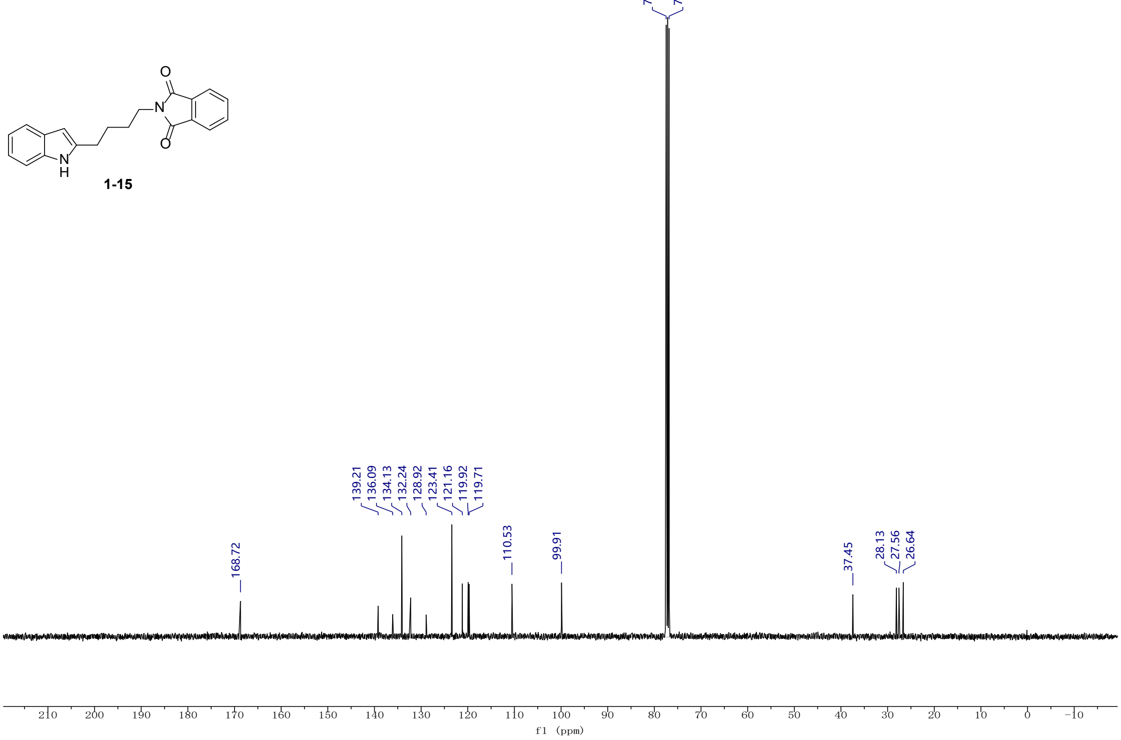

2018-1.3036.fid
LHL1-49-1.2

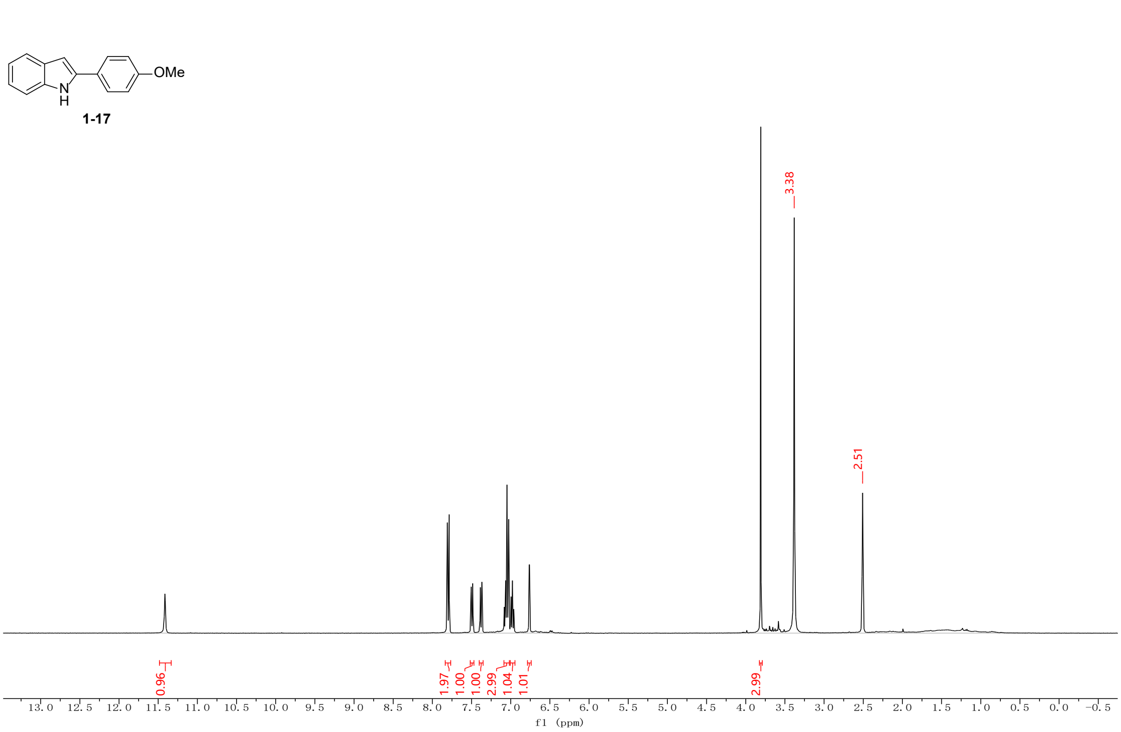

2019-1.1324.fid
LHL-3-12-1.2

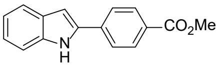

1-18

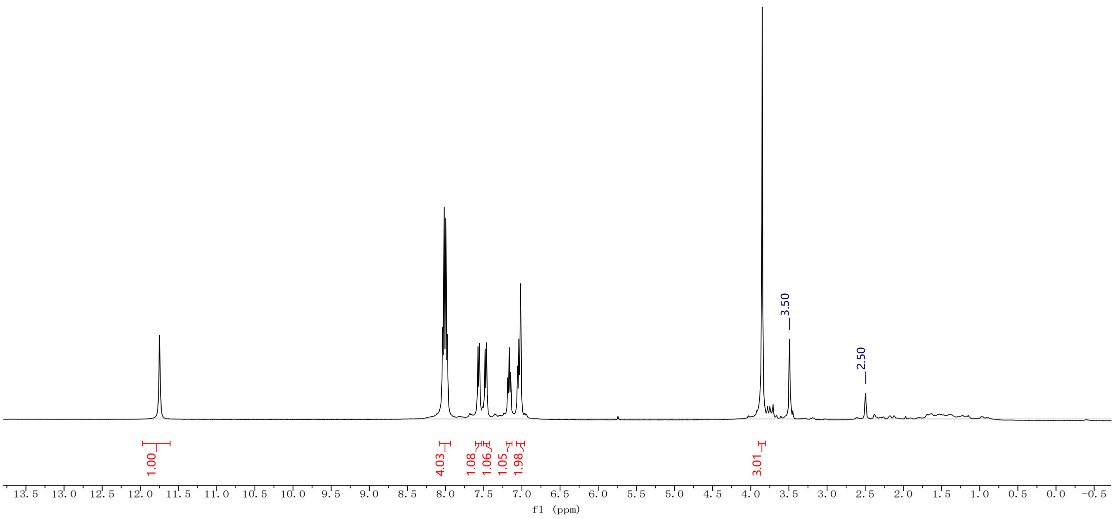

2018-1.2630.fid
LHL1-48-2

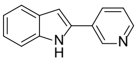

1-19

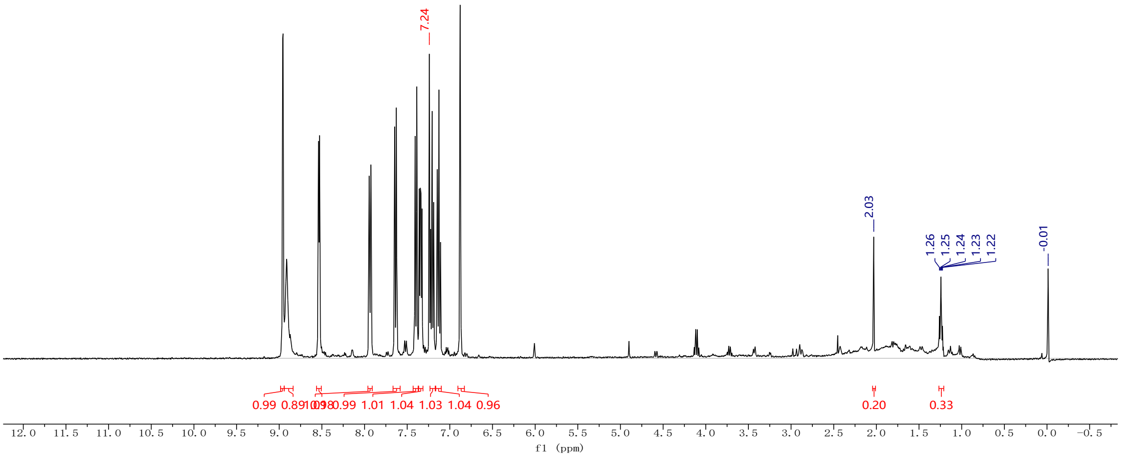

2019-1.15013.fid
LHL 4-29-1

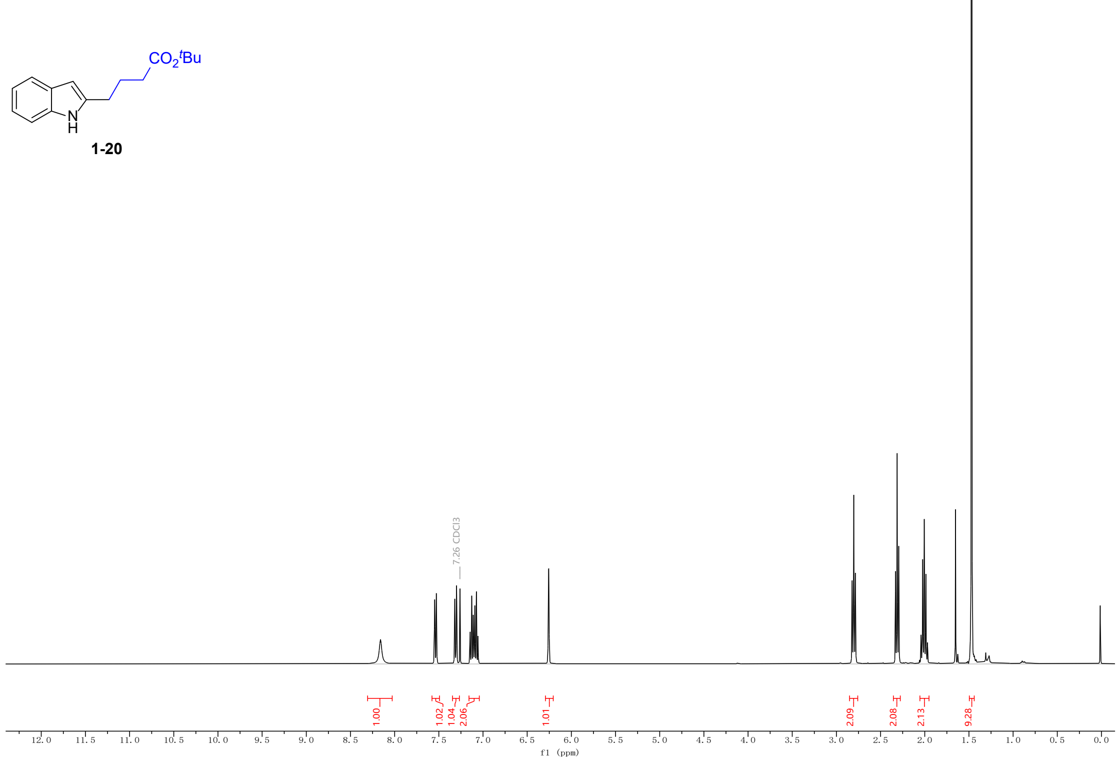

2019-1.15522.fid
LHL 4-29-1

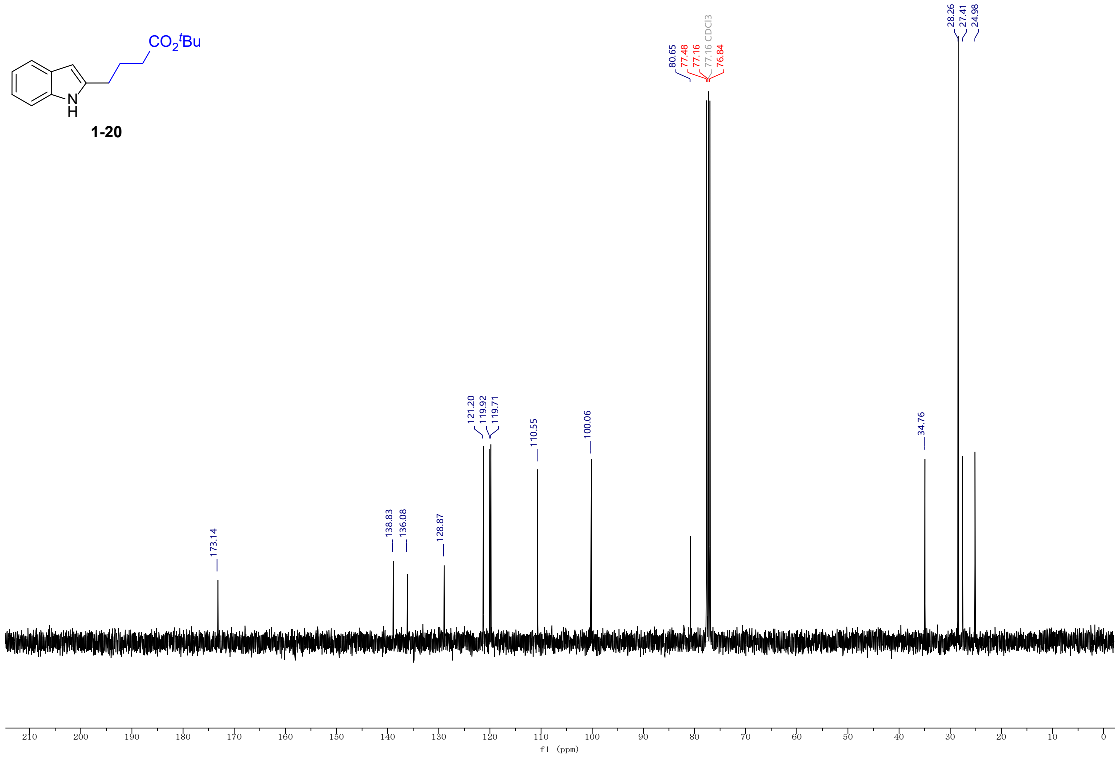

2018-2.10404.fid
LHL -2-44-1

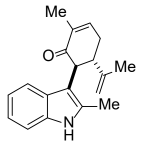

6-1

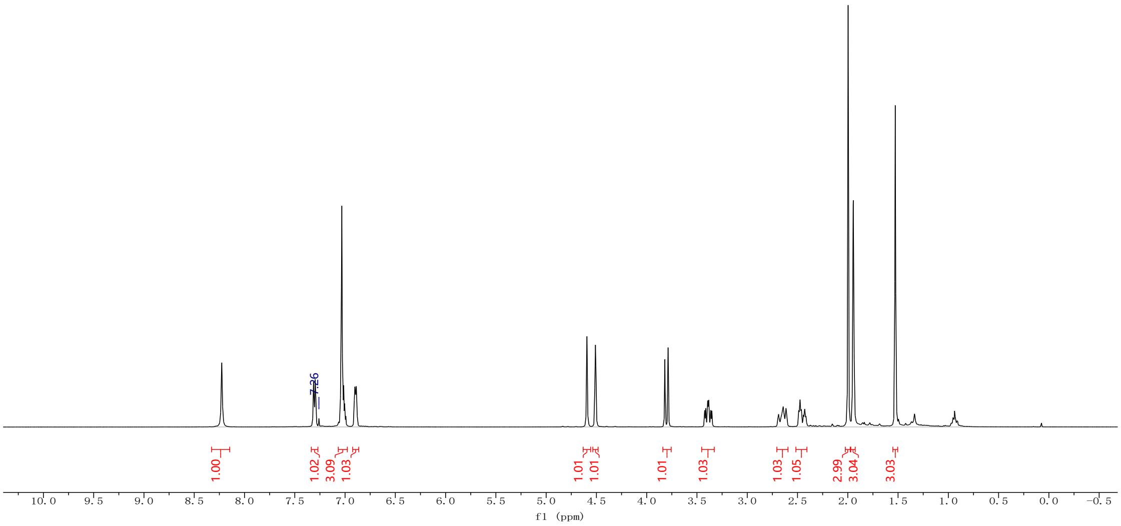

2018-2.10849.fid
LHL-2-44-1

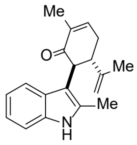

6-1

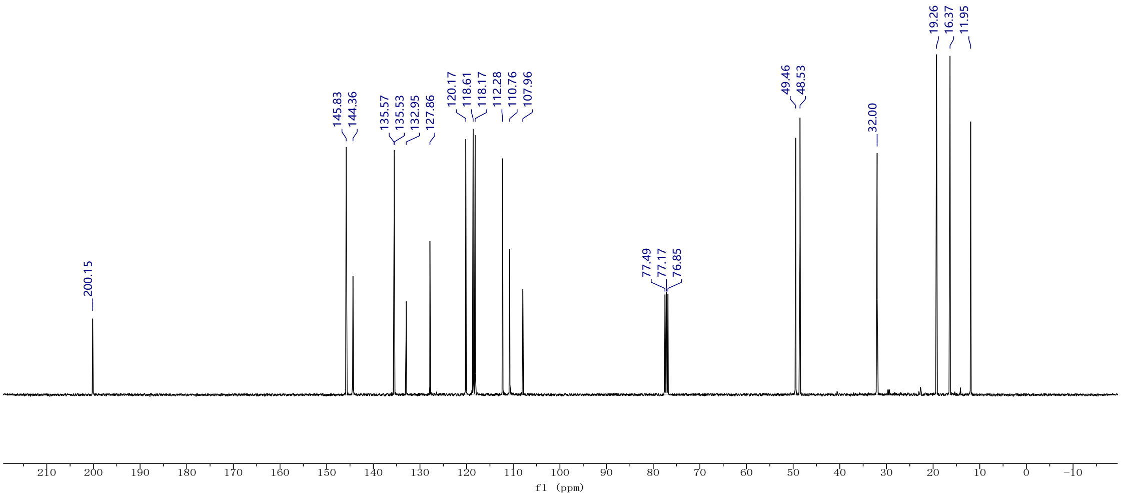

2017-3.17979.fid
LHL1-30-2

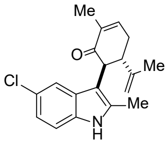

6-2

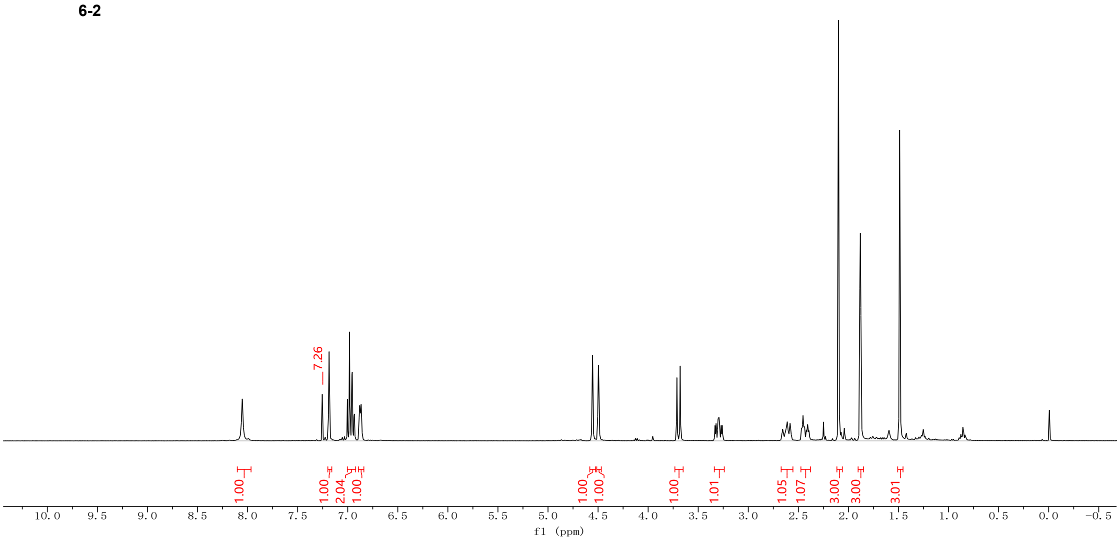

2017-3.18392.fid
LHL1-30-2

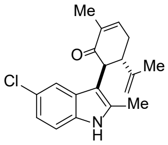

6-2

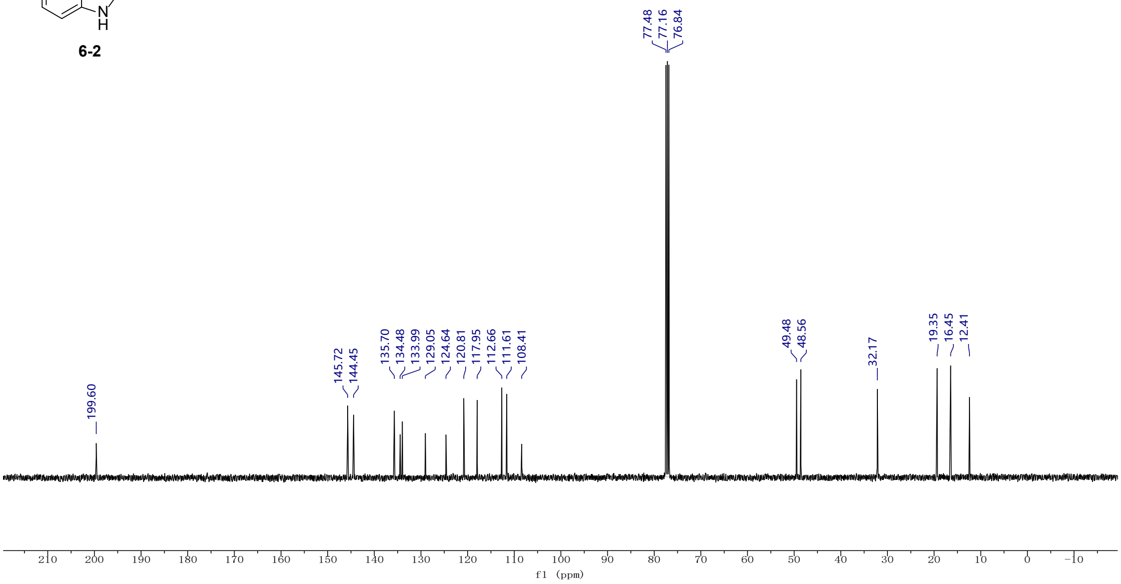

2018-1.3031.fid
LHL1-39-2

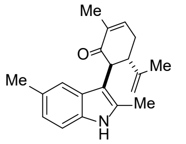

6-3

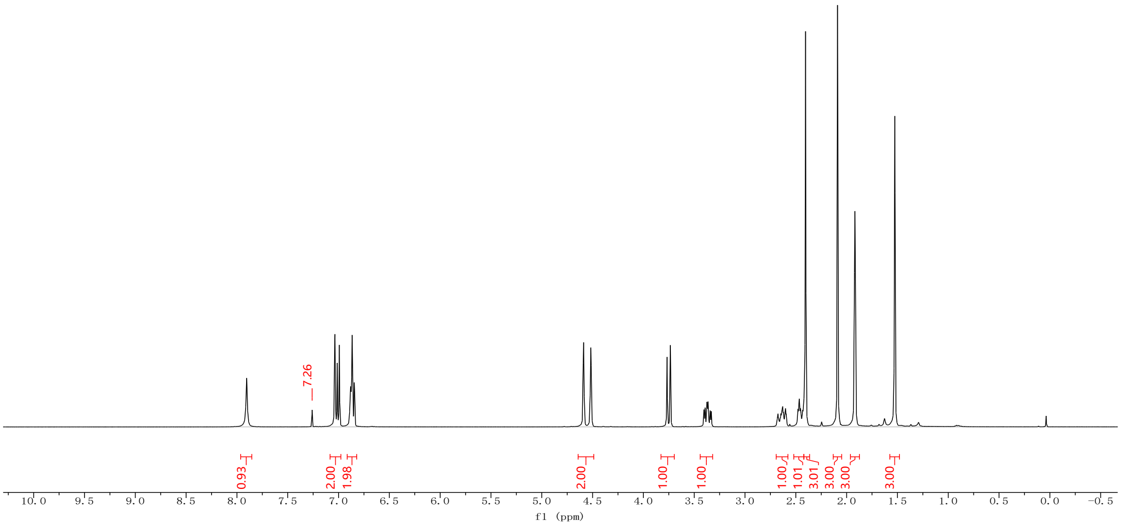

2018-1.3175.fid
LHL1-39-2

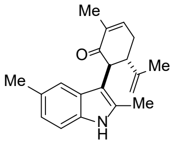

6-3

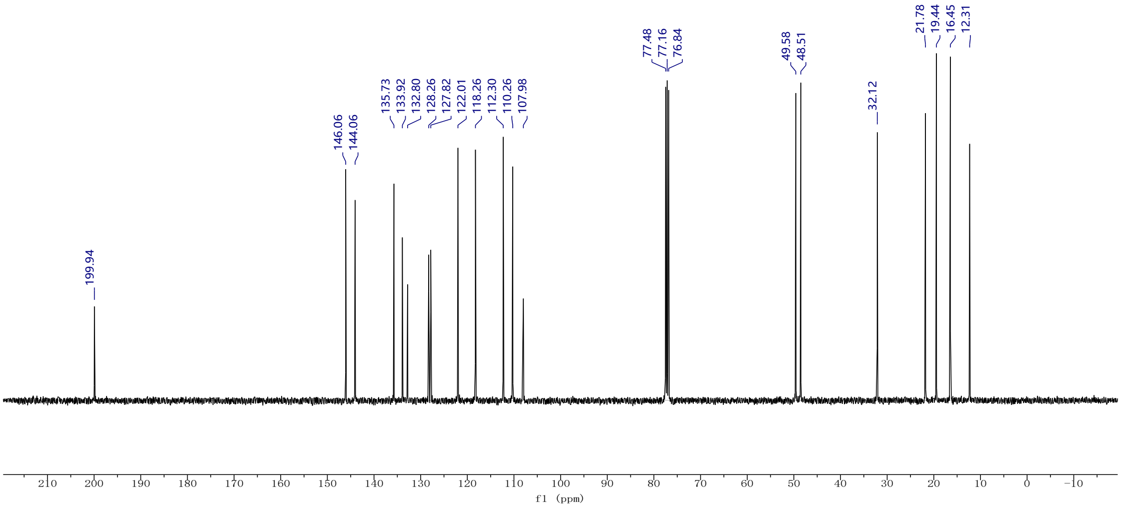

2017-3.17980.fid
LHL1-31-1

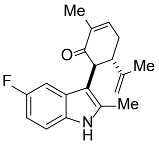

6-4

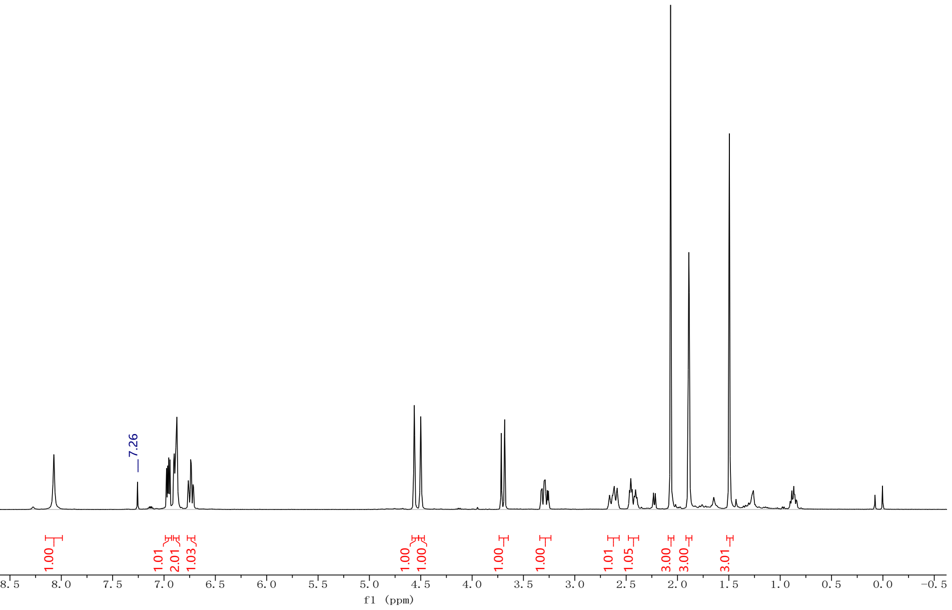

2017-3.18393.fid
LHL1-31-1

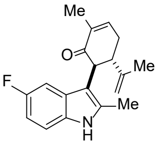

6-4

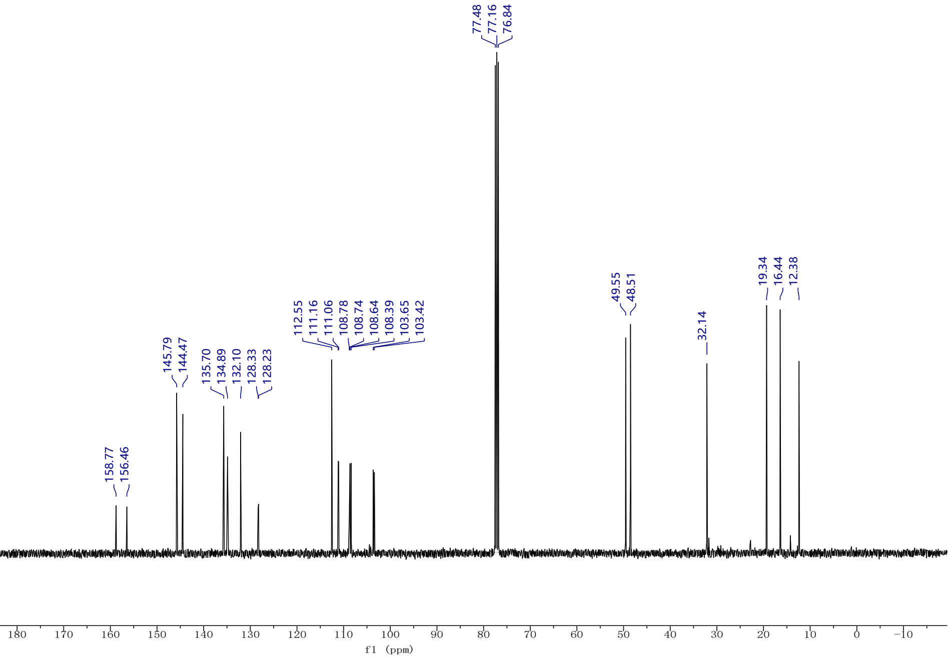

chenzhilong-20190505-2#.30.fid
LHL-1-31-1

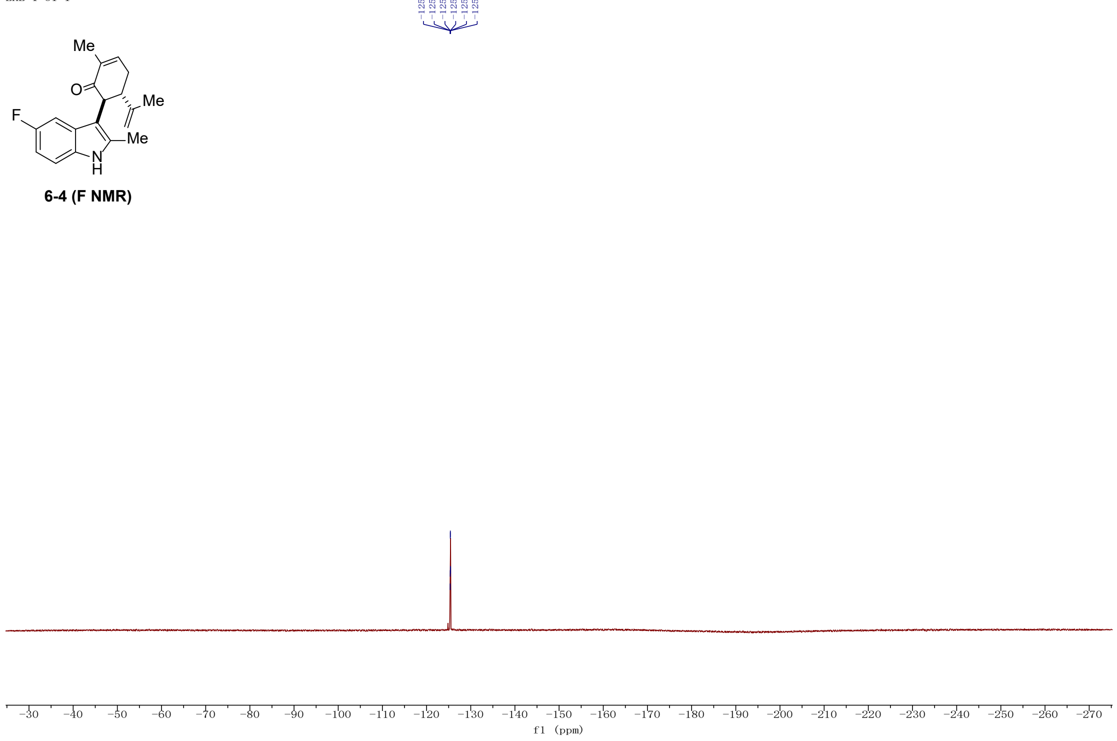

xiangguangya-20180427-37#.1.fid
lhl-1-64-2

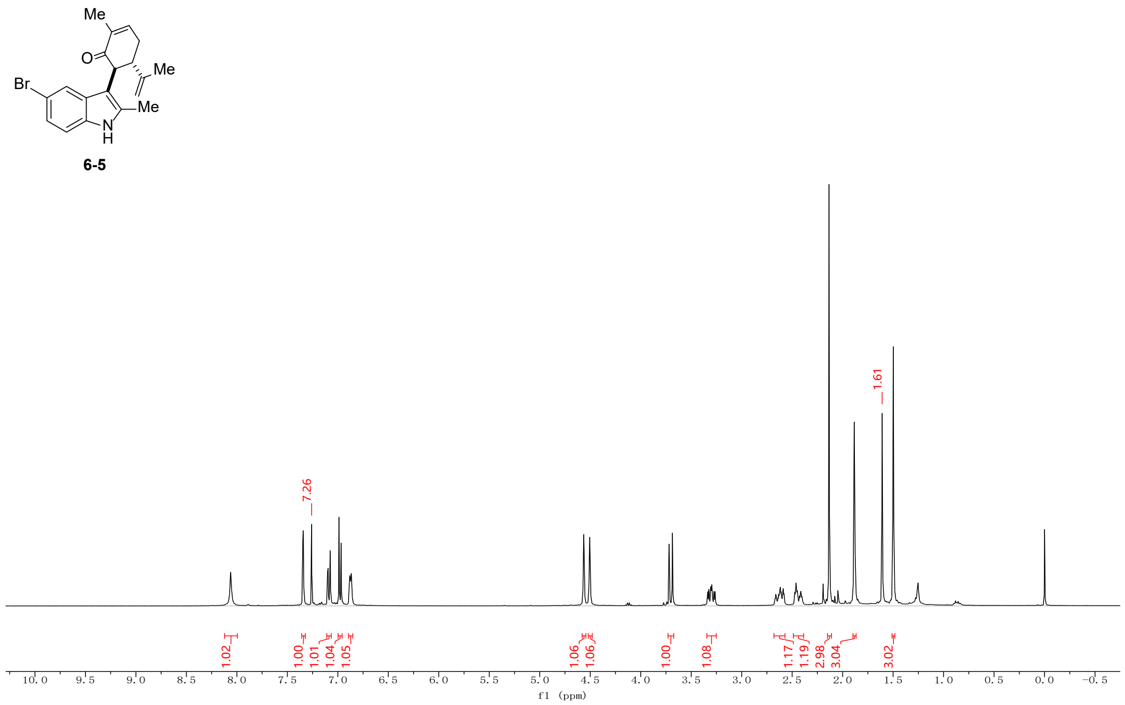

2019-1.2997.fid
LHL-1-64-2

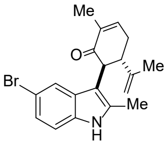

6-5

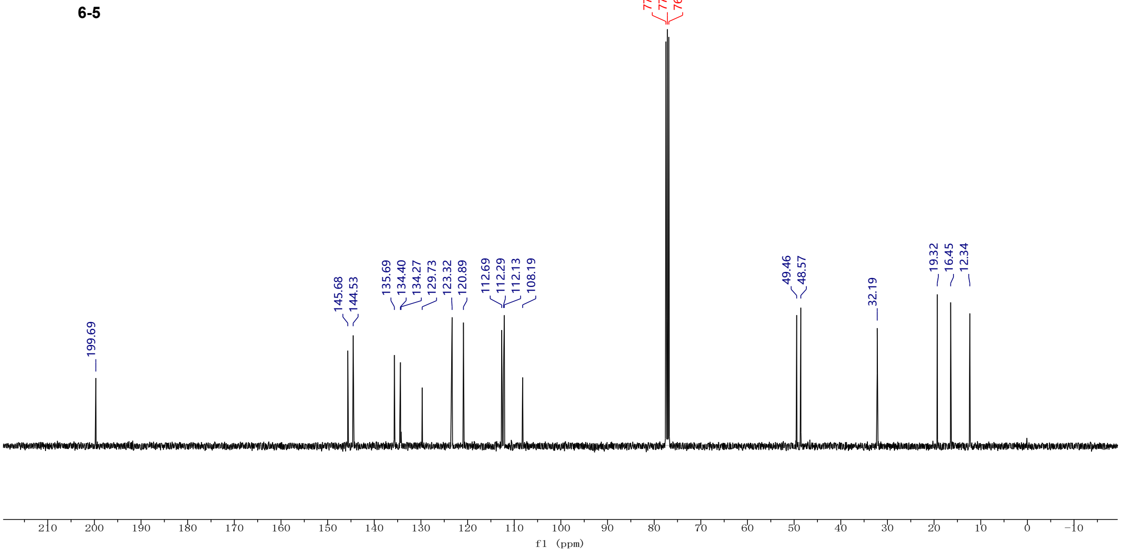

2017-3.17981.fid
LHL1-31-2

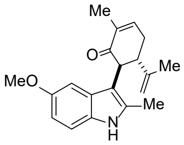

6-6

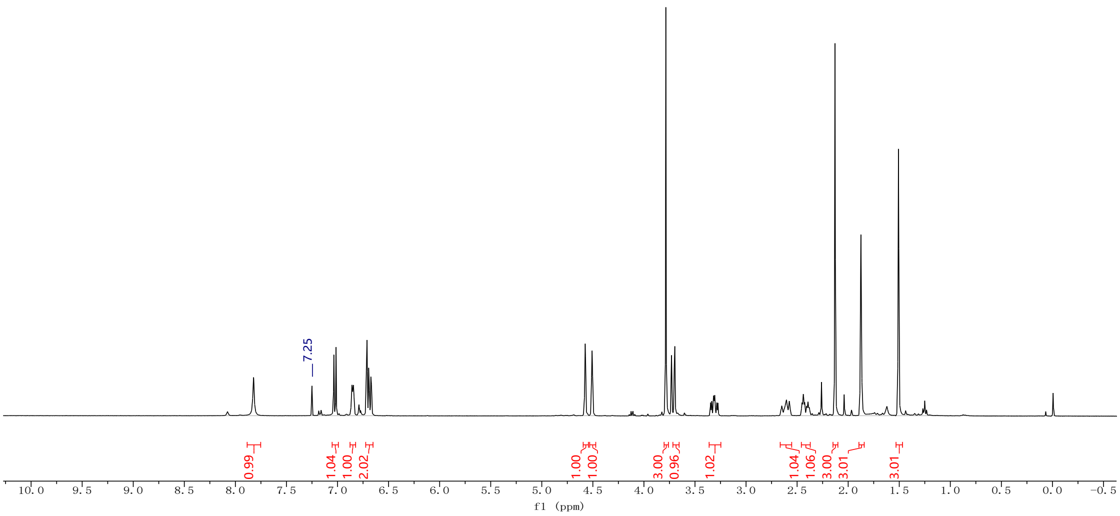

2017-3.18391.fid
LHL1-31-2

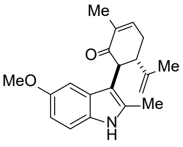

6-6

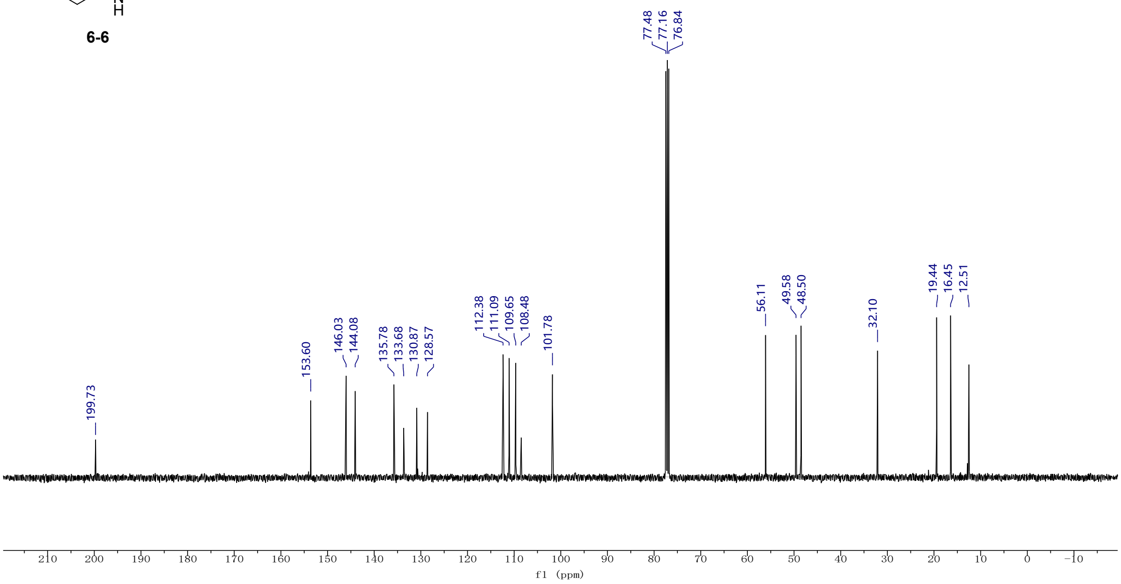

2018-2.8141.fid
LHL-2-32-2

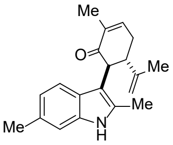

6-7

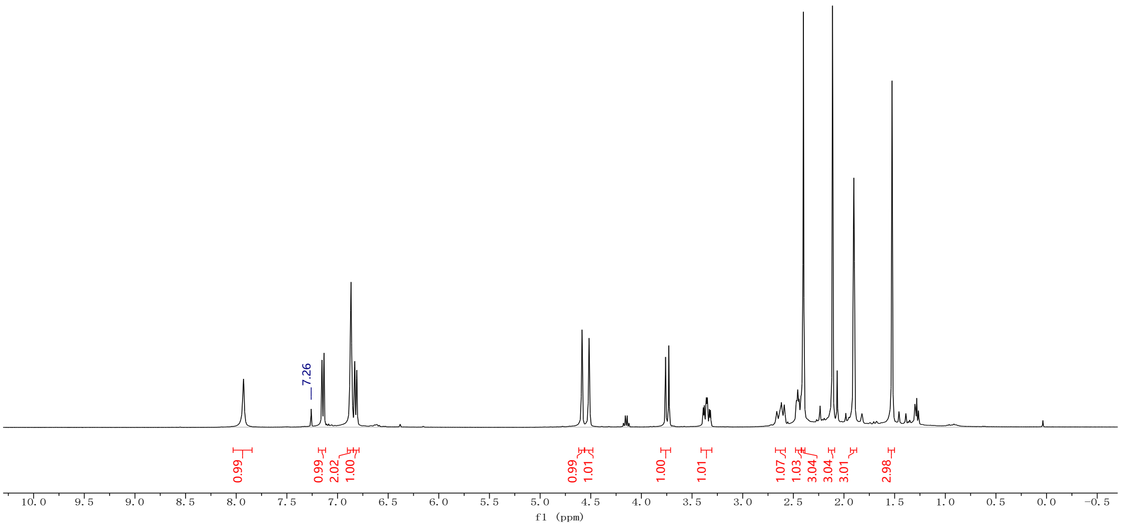

2018-2.8621.fid
LHL-2-32-2

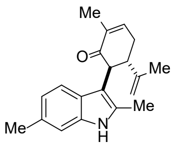

6-7

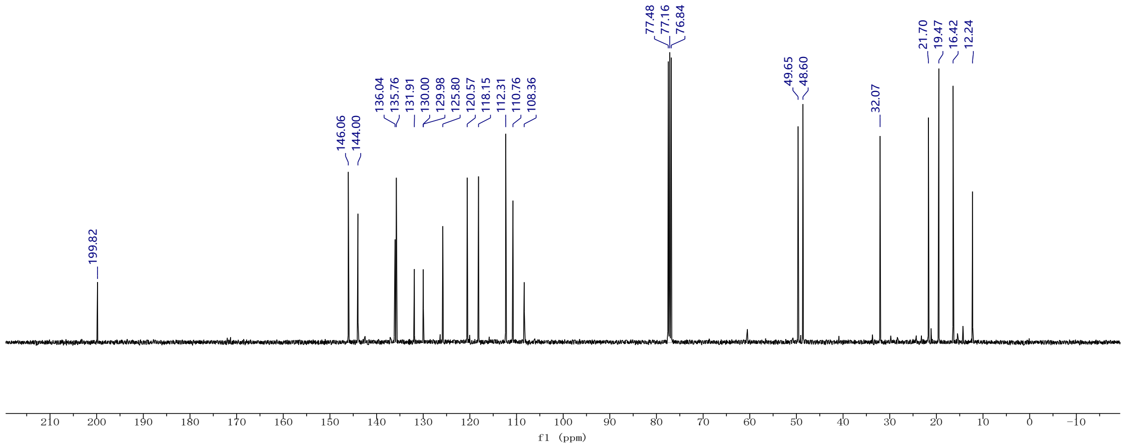

2018-2.8139.fid
LHL-2-33-1.1

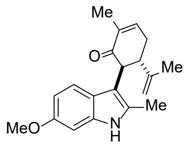

6-8

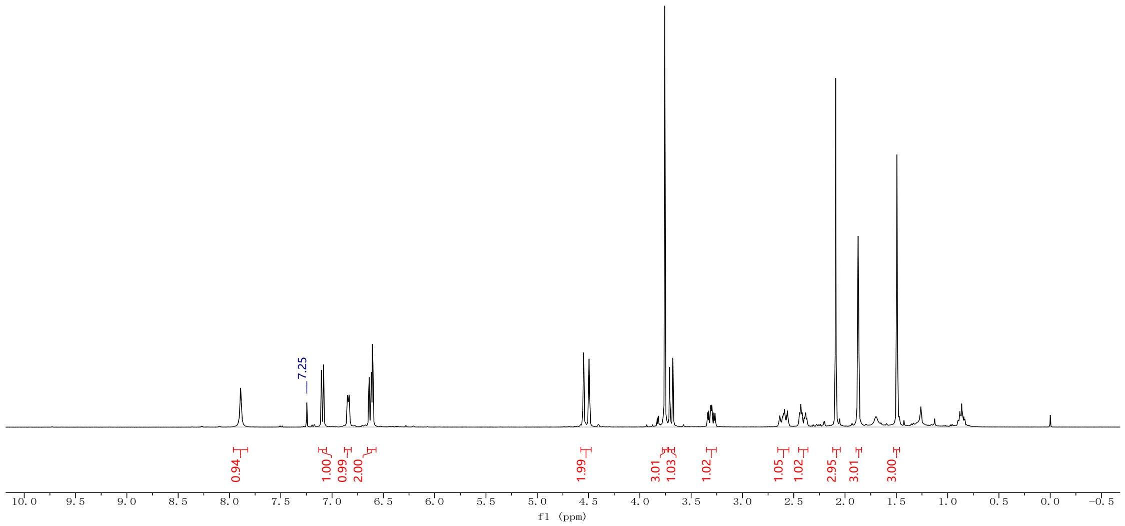

LHL-2-33-1.1

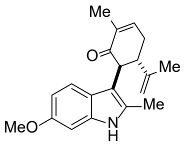

6-8

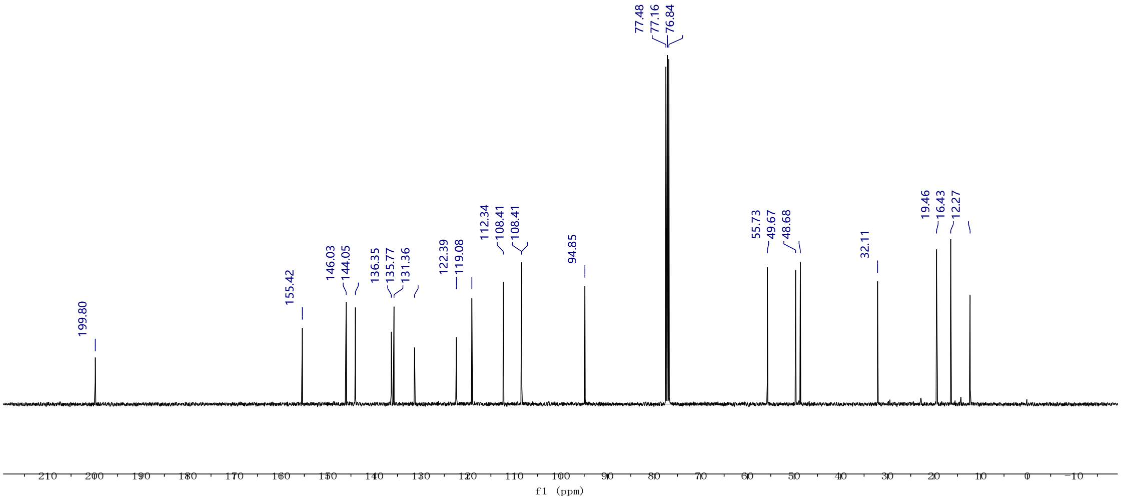

LHL-2-41-2

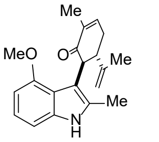

6-9

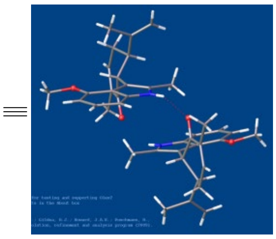

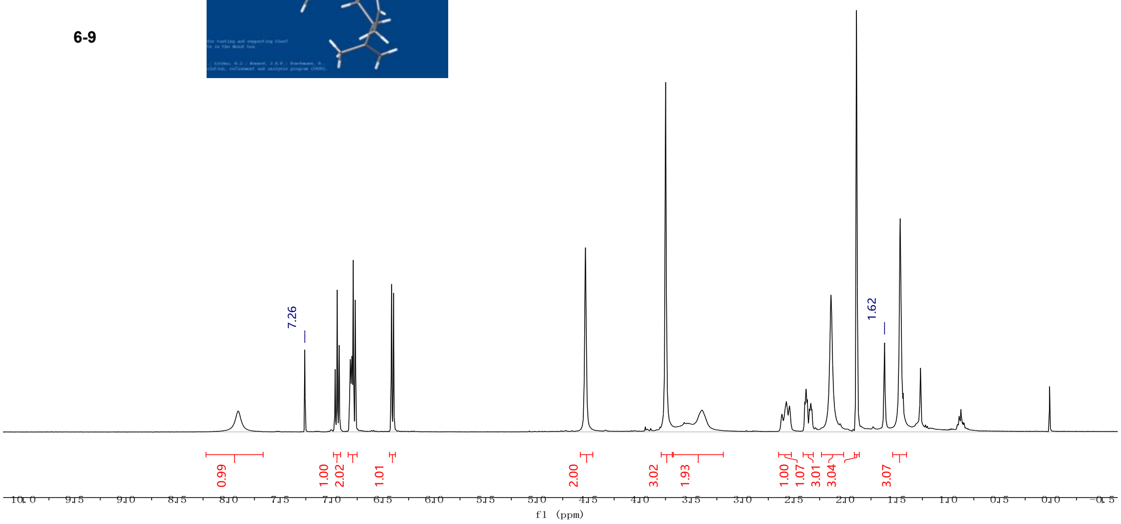

LHL 2-41-2

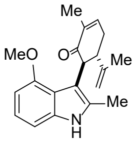

6-9

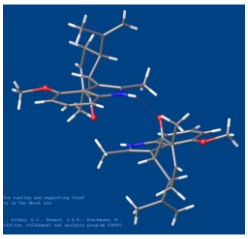

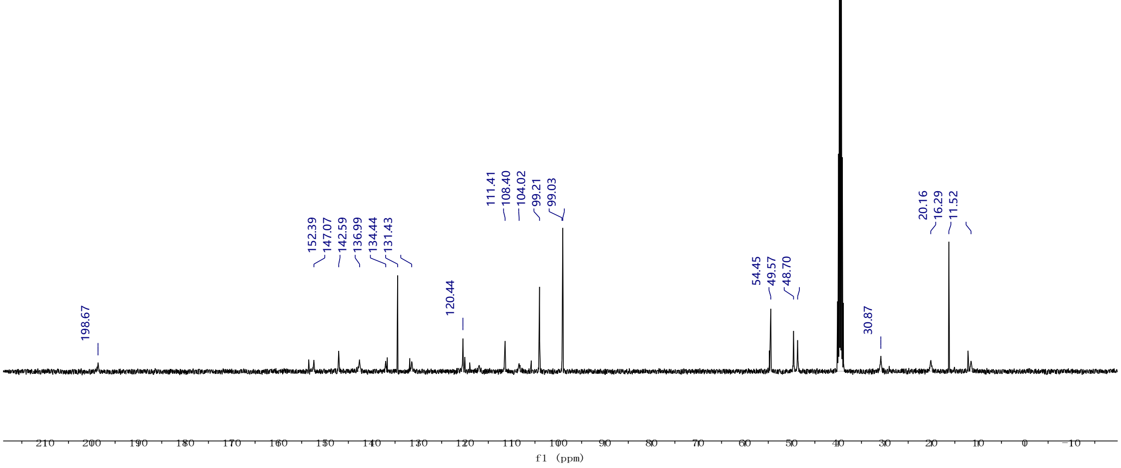

LHL2-25-1

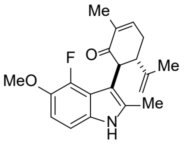

6-10

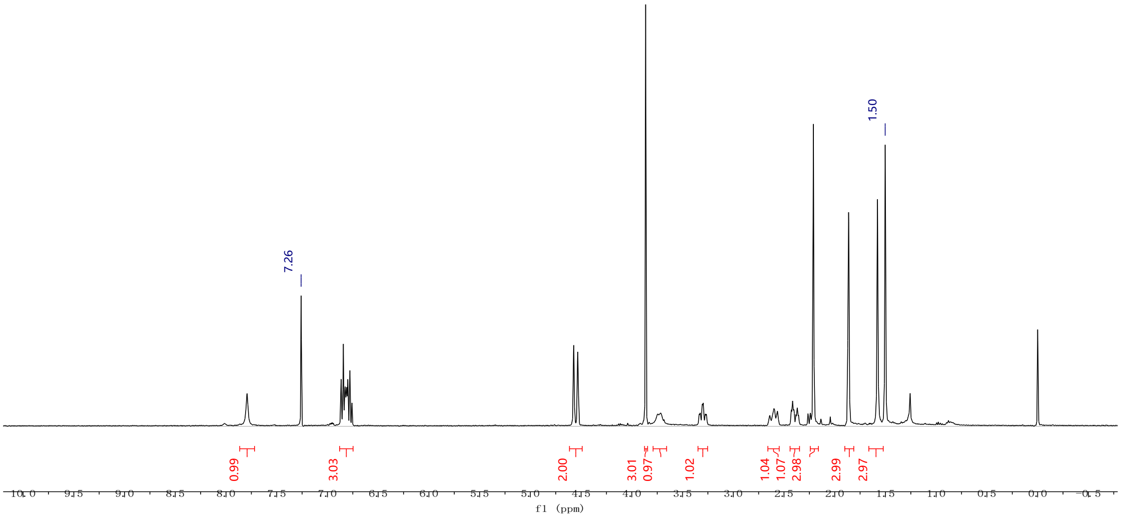

2019-1.4789.fid
LHL 2-25-1

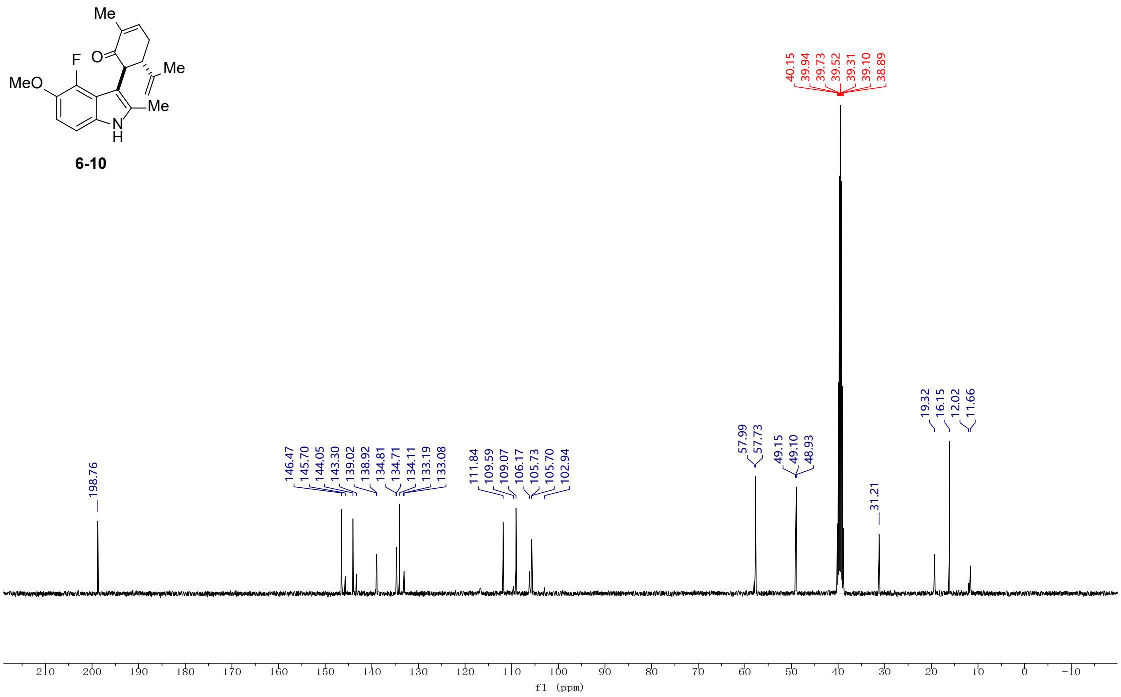

chenzhilong-20190505-3#.30.fid
LHL-2-25-1

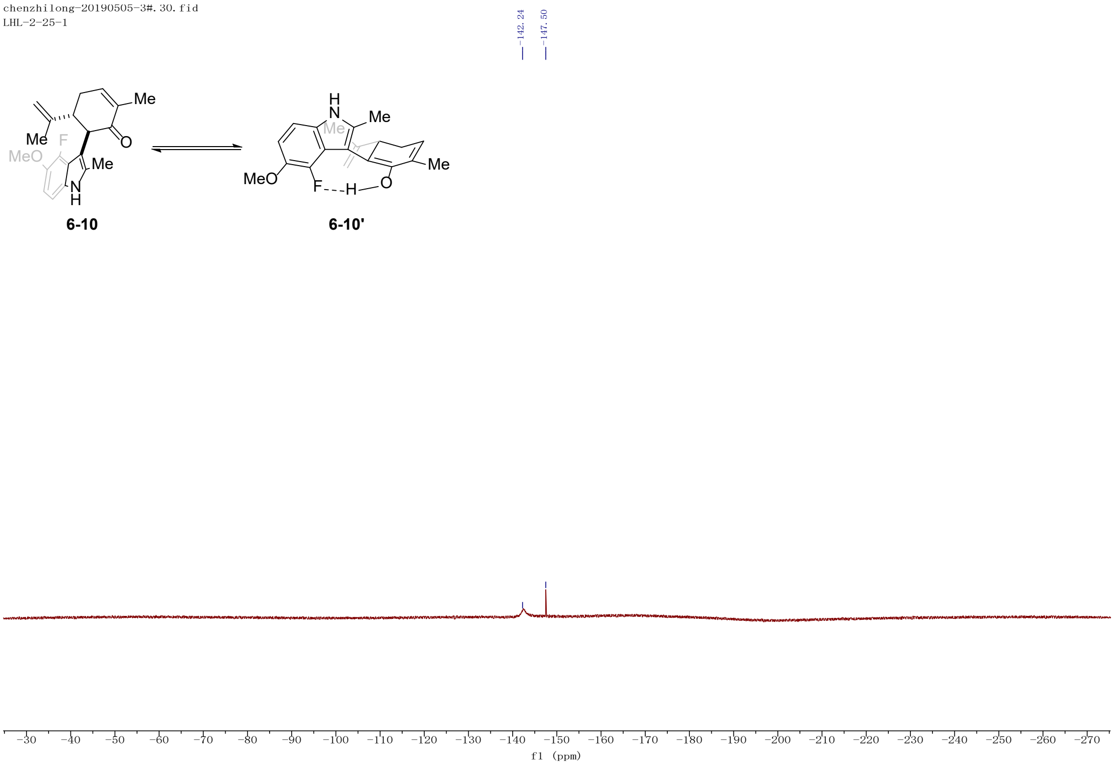

2018-2.7054.fid
LHL2-24-1

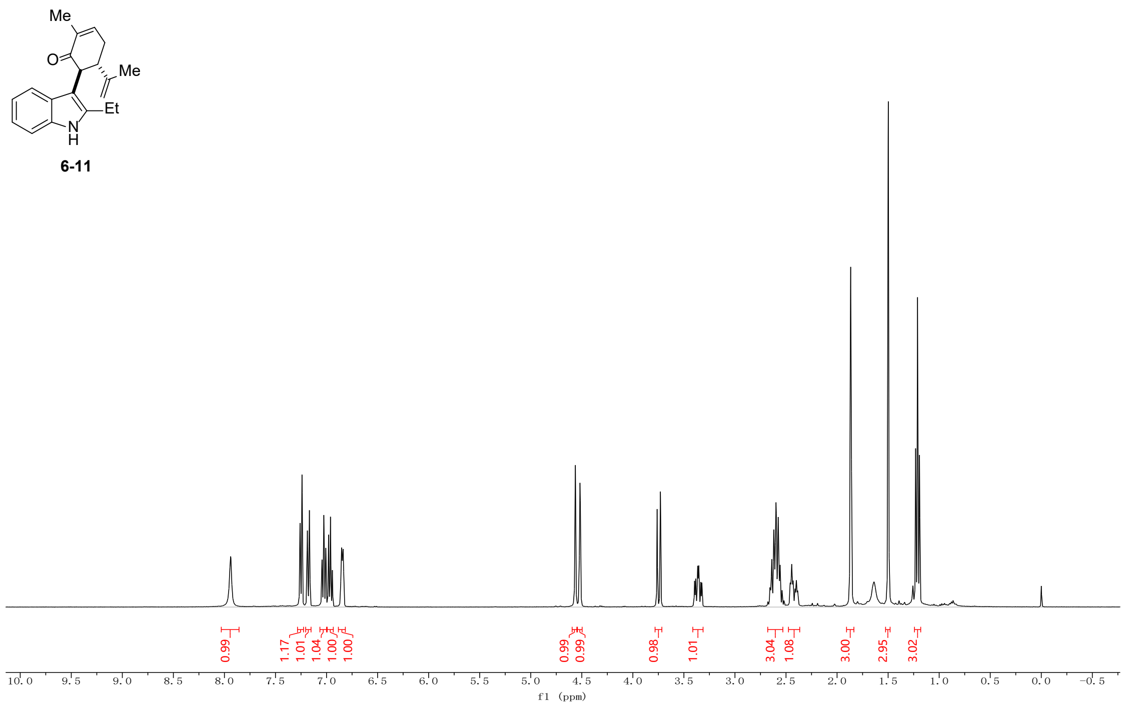

6-11

2018-2.7488.fid
LHL-2-24-1

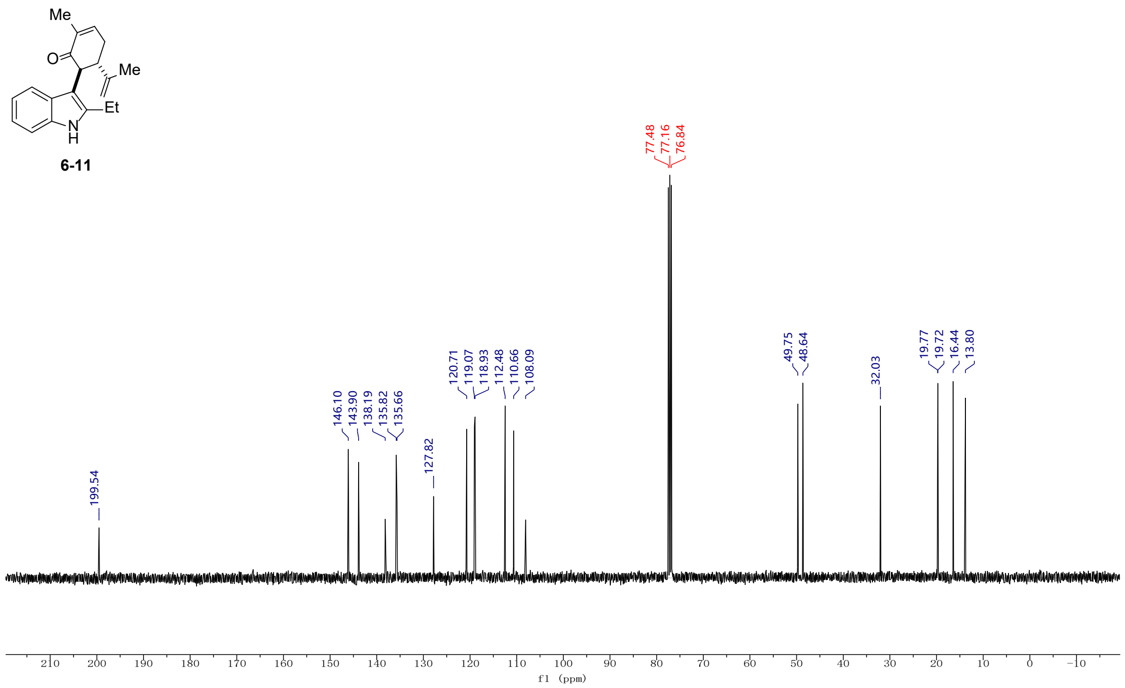

6-11

2018-2.13508.fid
LHL-2-78-2

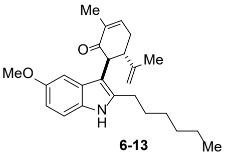

6-13

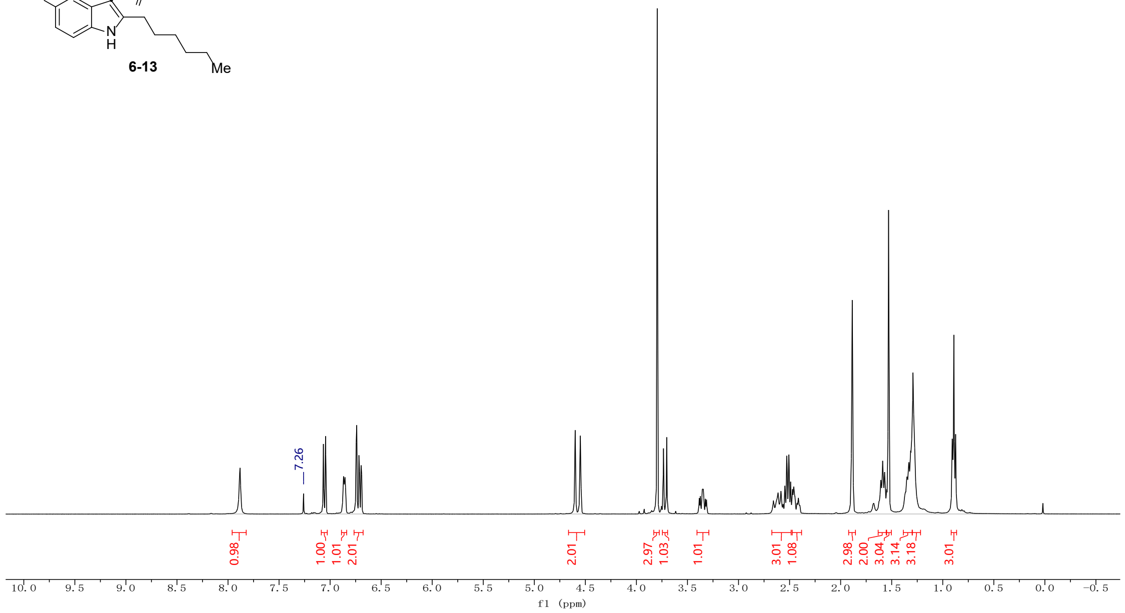

2018-2.14039.fid
LHL-2-78-2

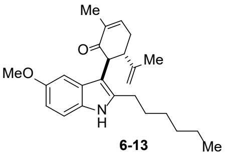

6-13

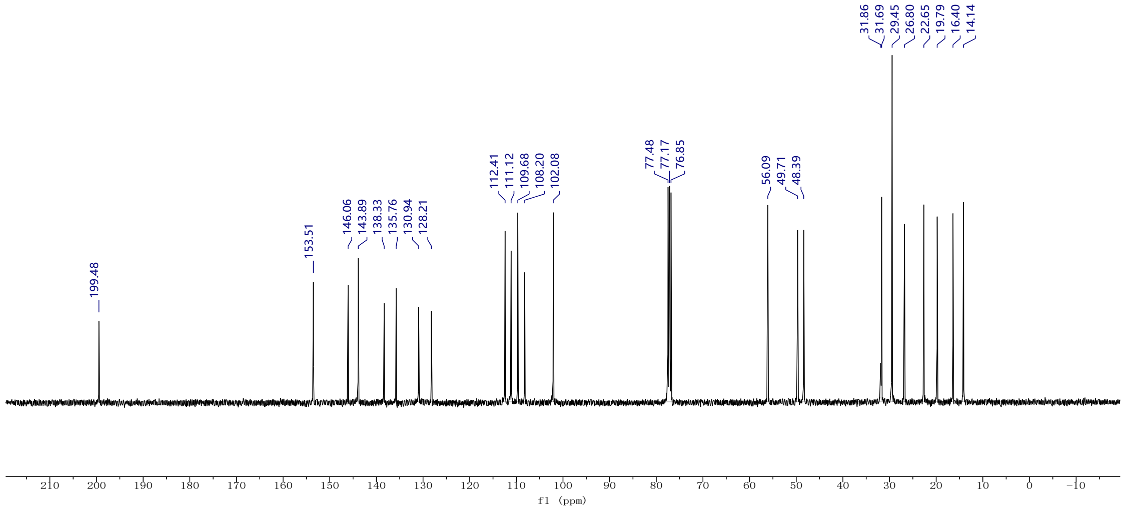

2018-2.13082.fid
LHL-2-74-2.3

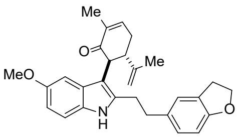

6-14

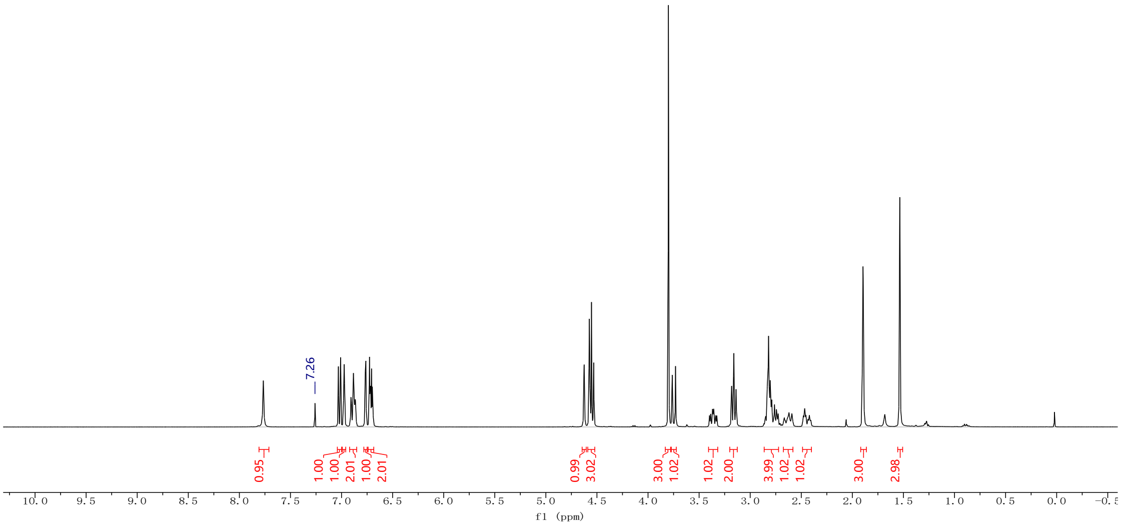

2019-1.2541.fid
LHL-2-74-2.3

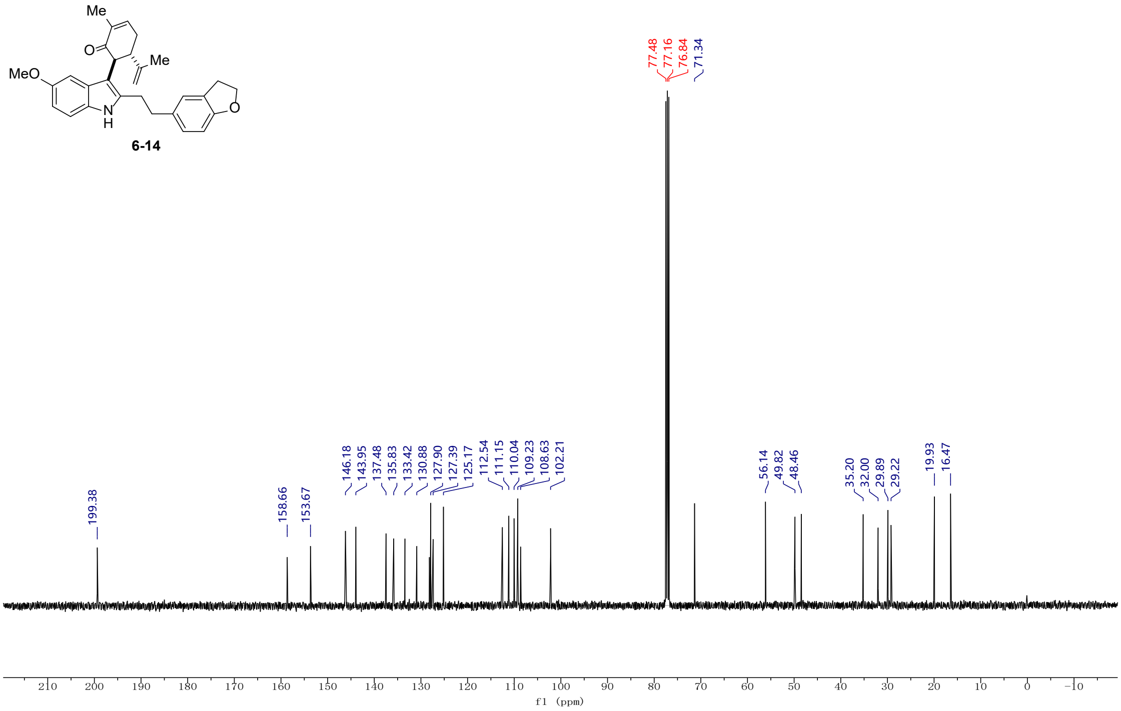

2019-1.674.fid
LHL-3-7-1

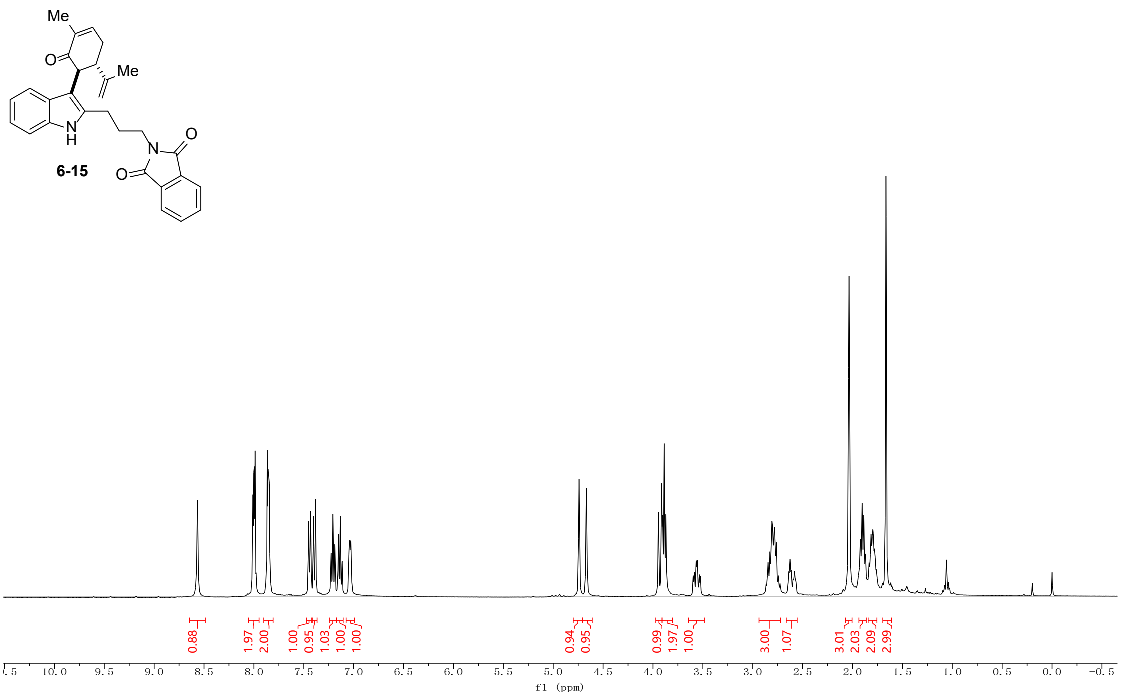

2019-1.861.fid
LHL-3-7-1

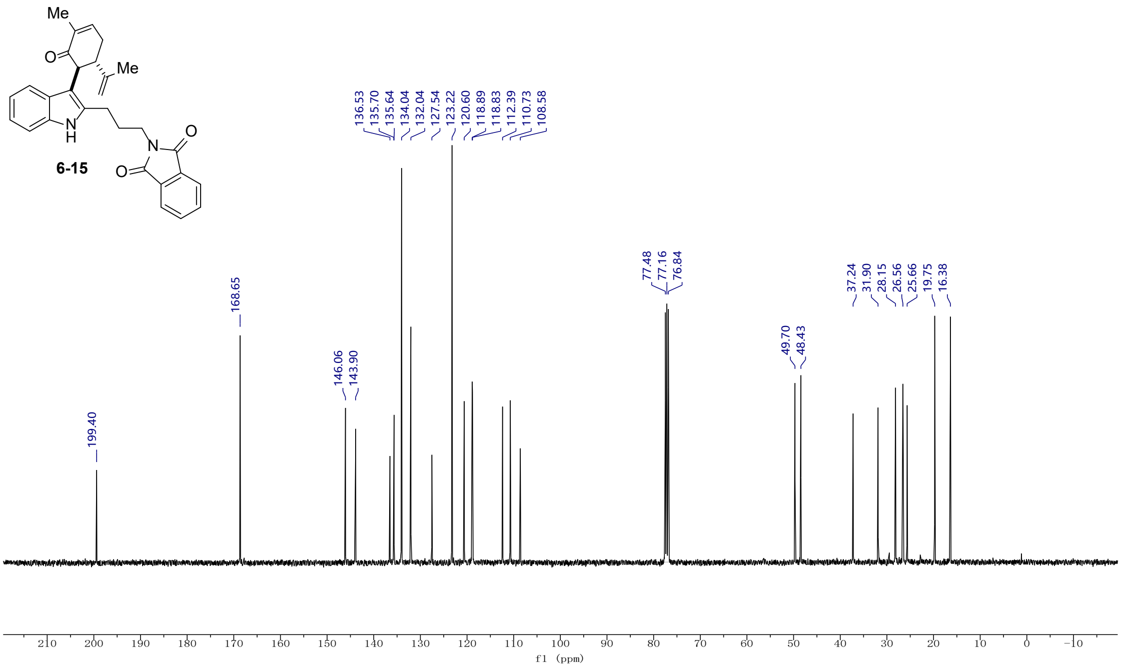

2017-3.18346.fid
LHL1-32-1

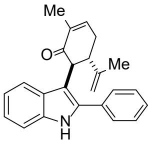

6-16

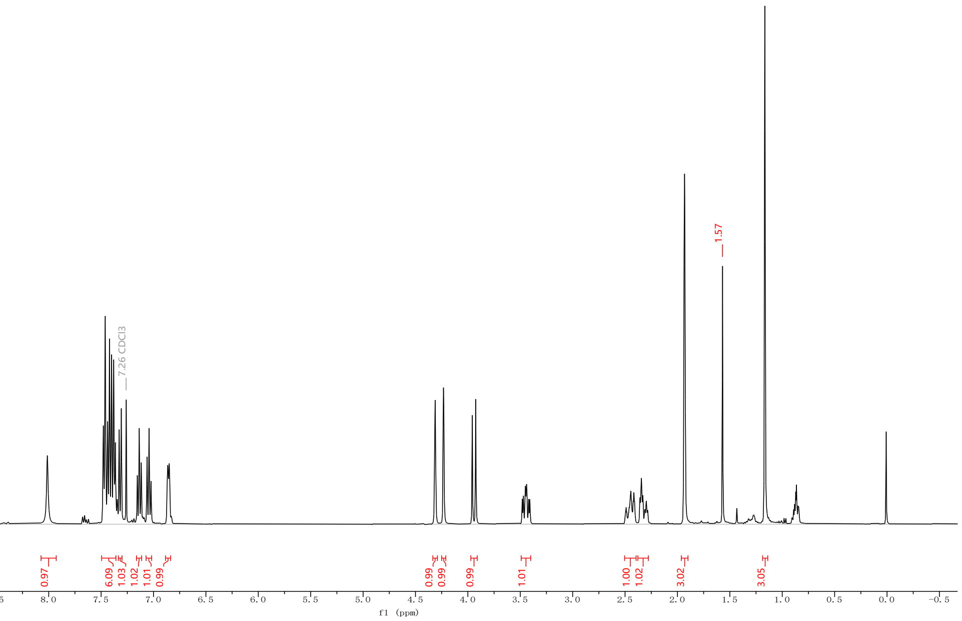

2018-1.399.fid
LHL-1-32-1

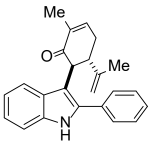

6-16

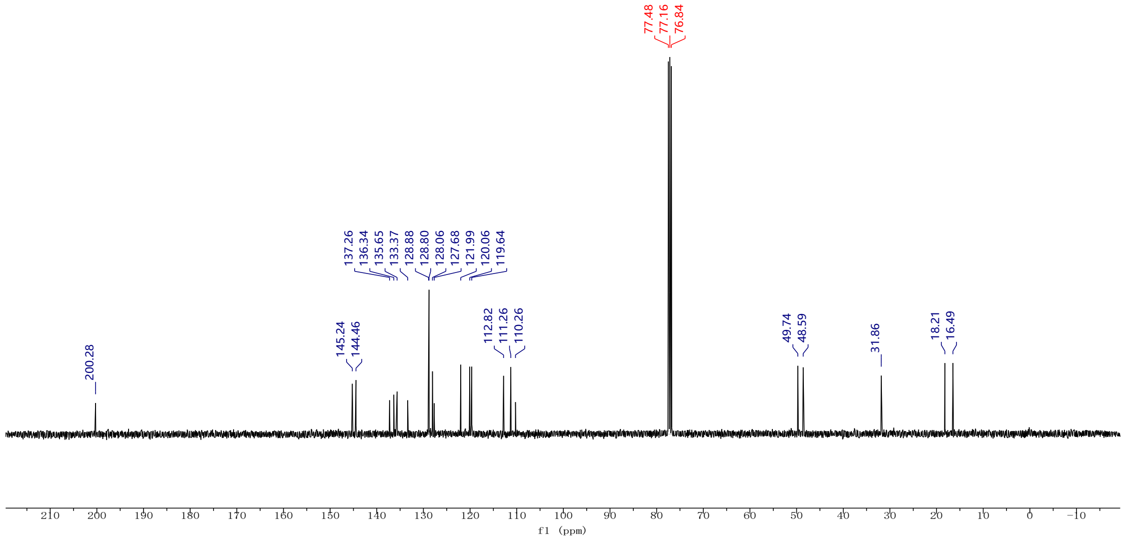

2018-2.5822.fid
LHL1-81-1

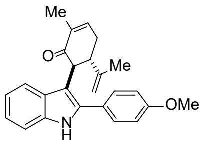

6-17

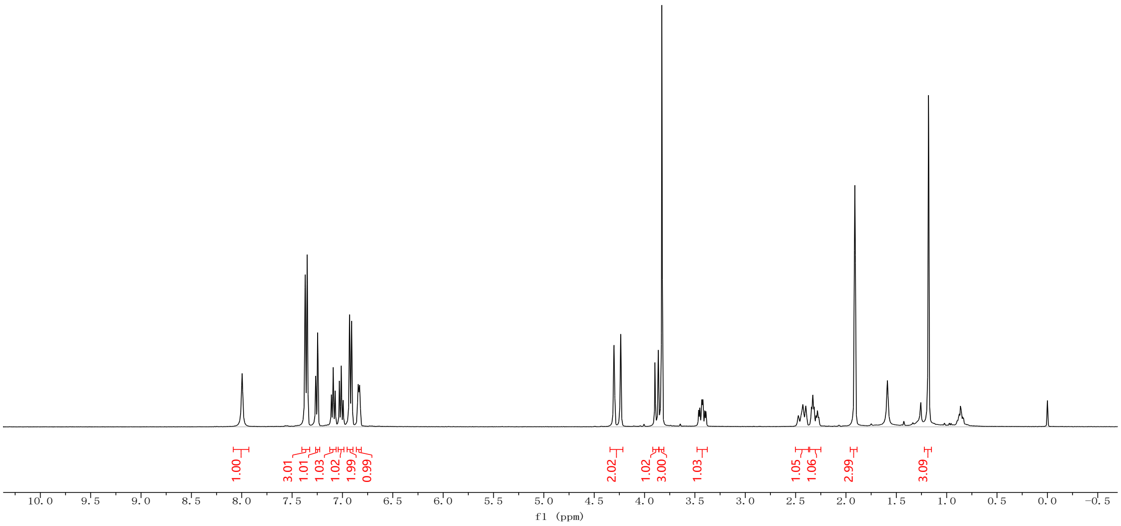

2019-1.2773.fid
LHL -1-81-1

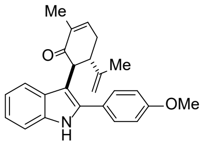

6-17

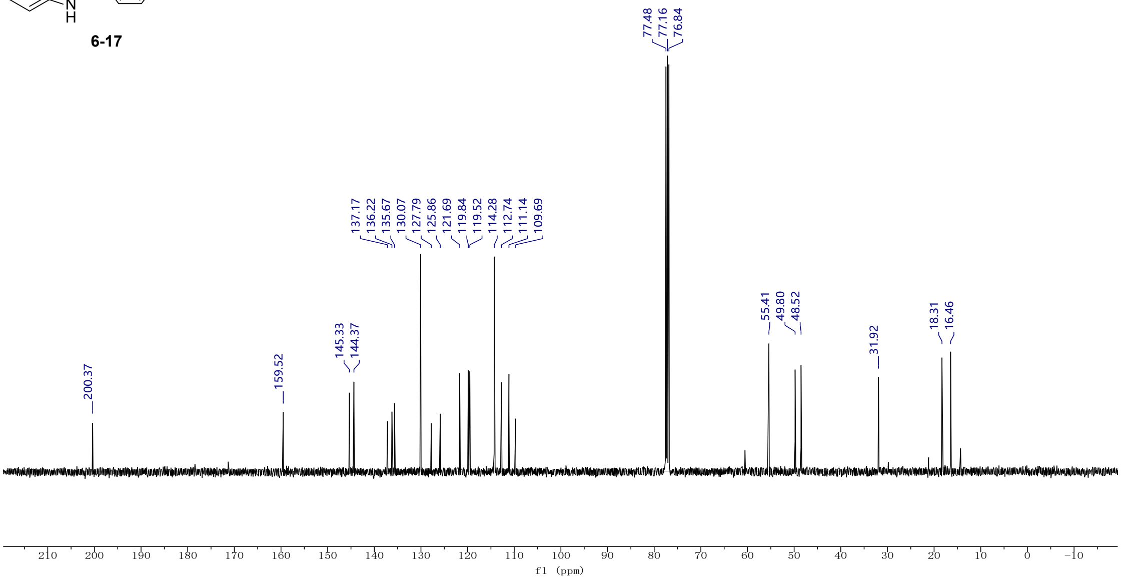

2019-1.3200.fid
LHL-3-15-1

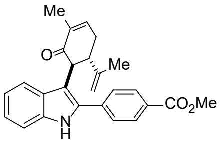

6-18

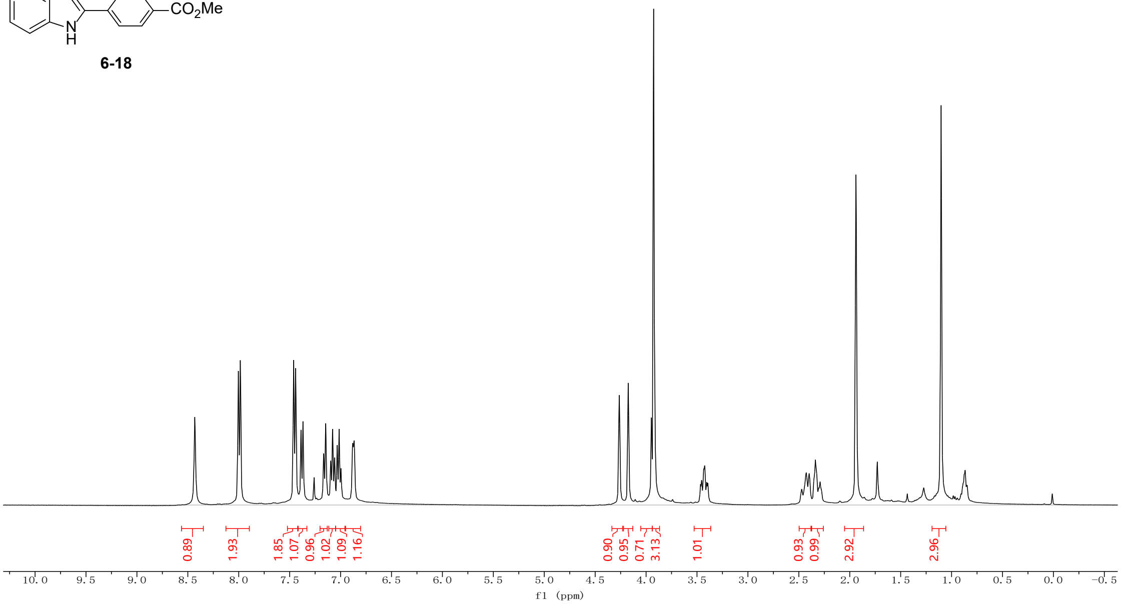

2019-1.3743.fid
LHL 3-15-1

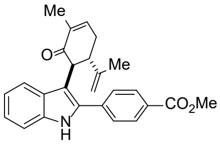

6-18

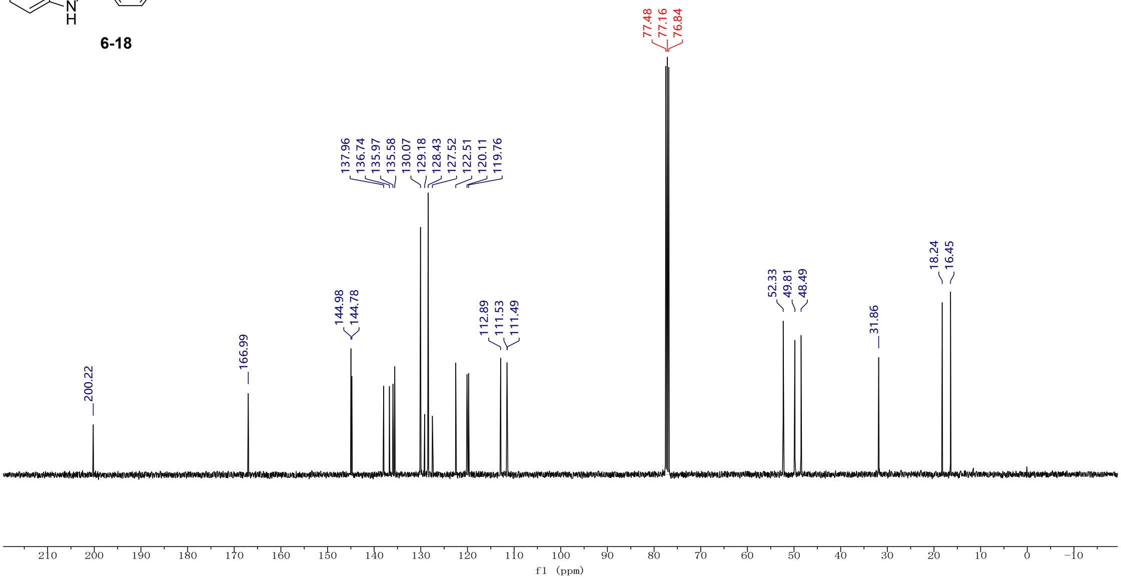

xiangguangya-20180629-003#.10.fid
LHL-1-82-2

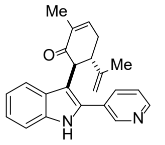

6-19

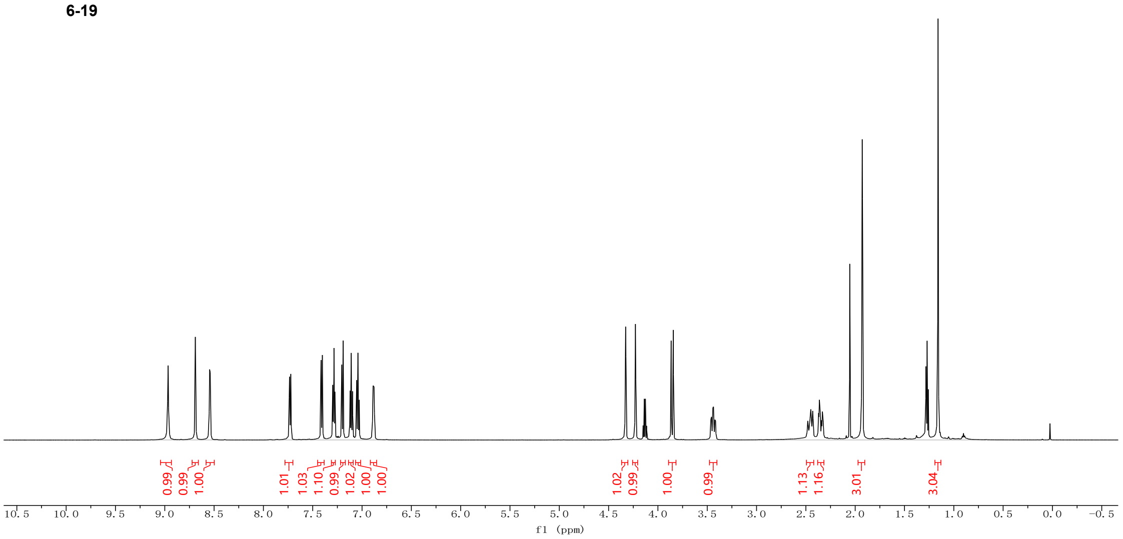

2019-1.3000.fid
LHL-1-82-2

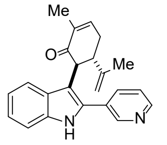

6-19

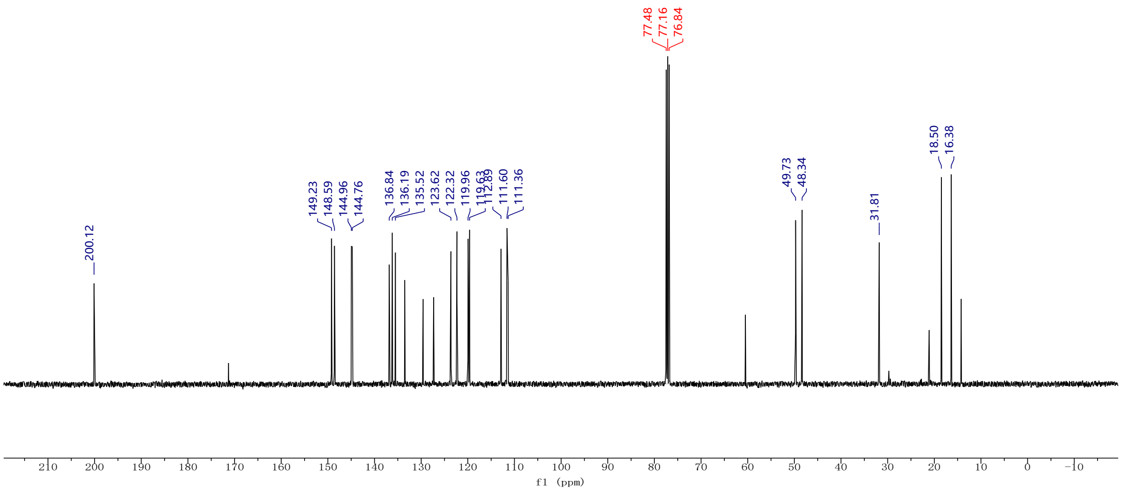

xiangguangya-20180601-18#.1.fid
LHL-1-77-1.2

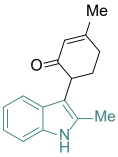

6-20

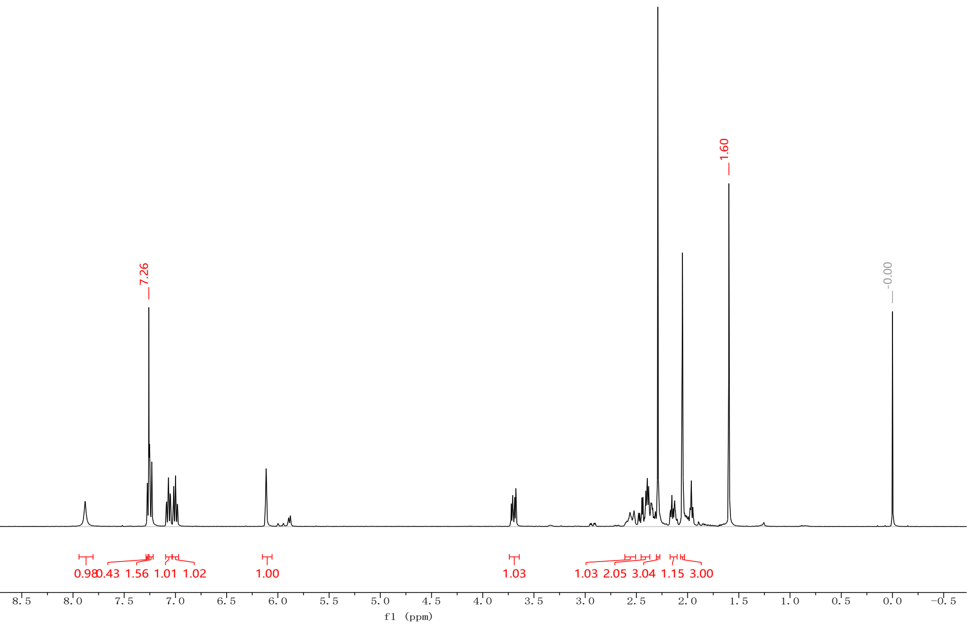

2019-1.2771.fid
LHL -1-71-1.2

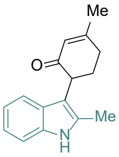

6-20

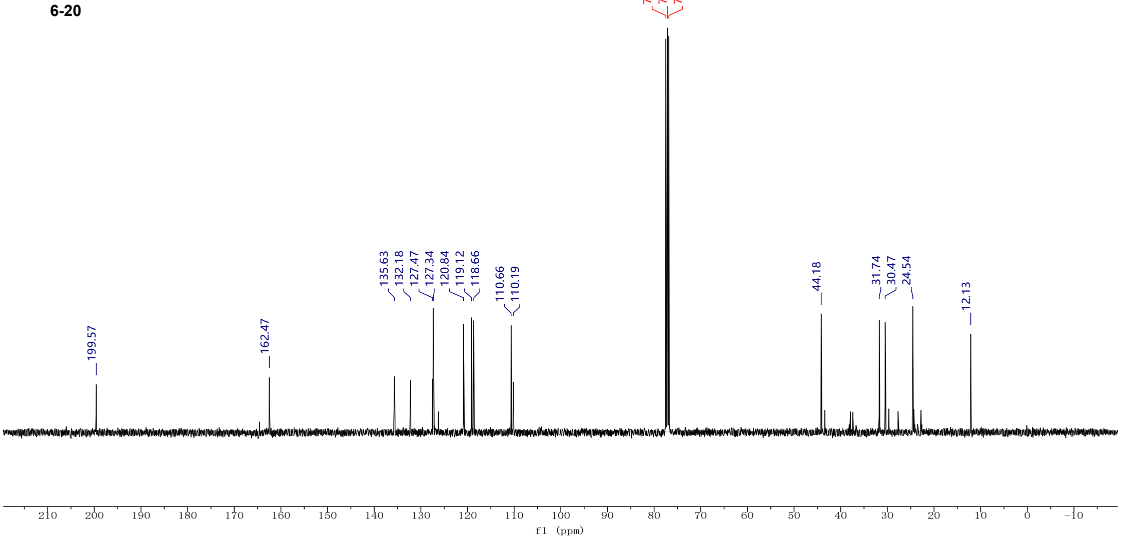

2018-1.55.fid
LHL1-33-1.3

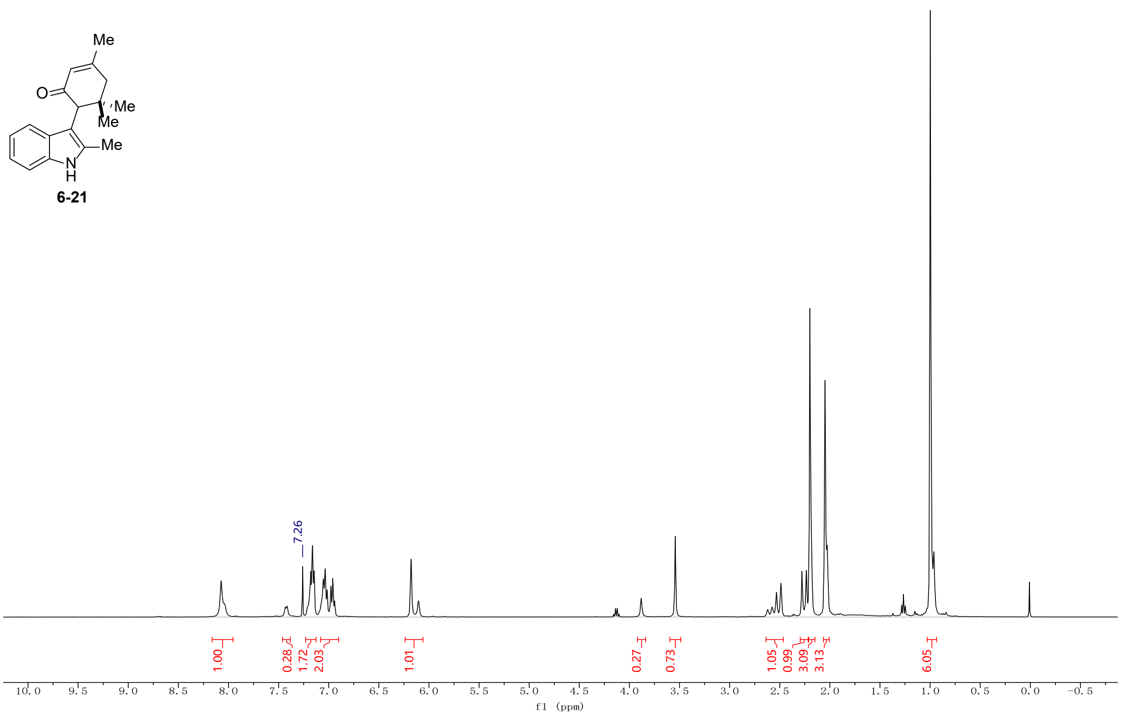

2019-1.2998.fid
LHL-1-33-1.3

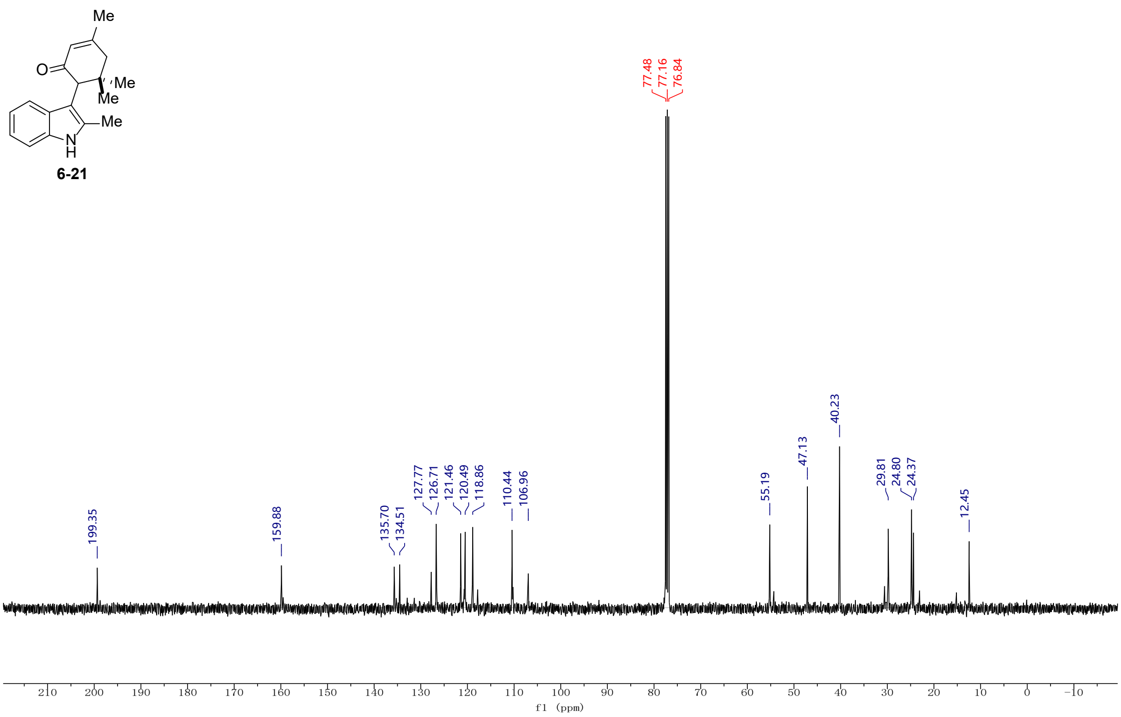

xiangguangya-20180511-36#.1.fid
LHL-1-66-1

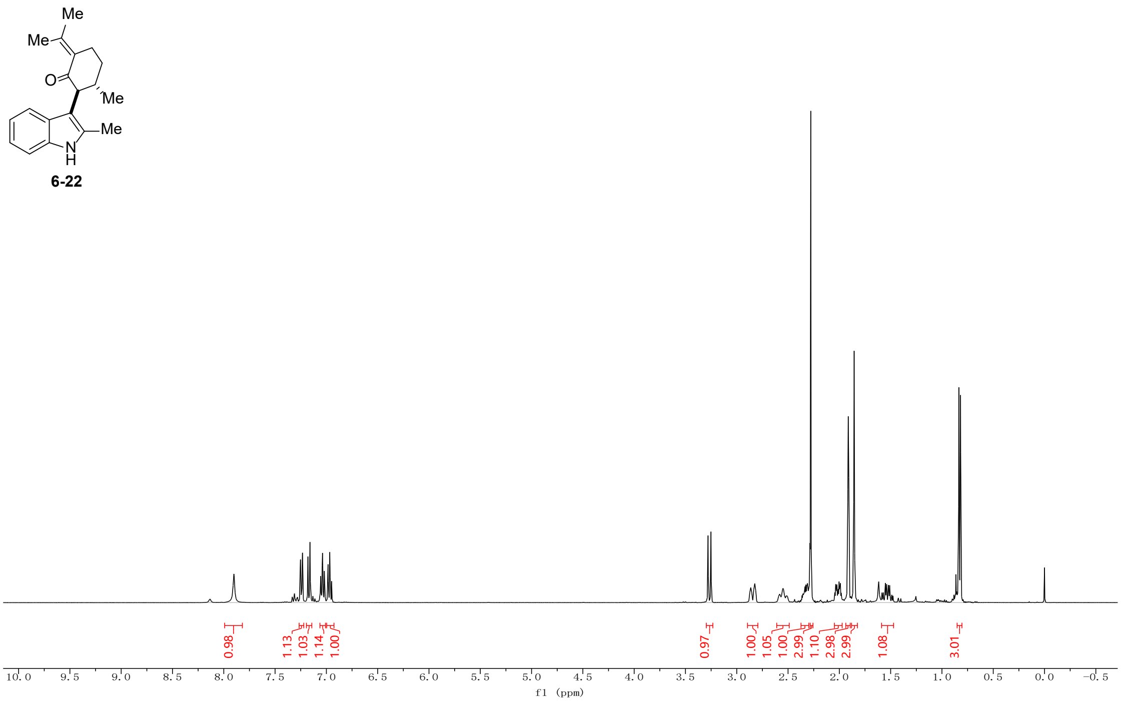

2019-1.2996.fid
LHL-1-66-1

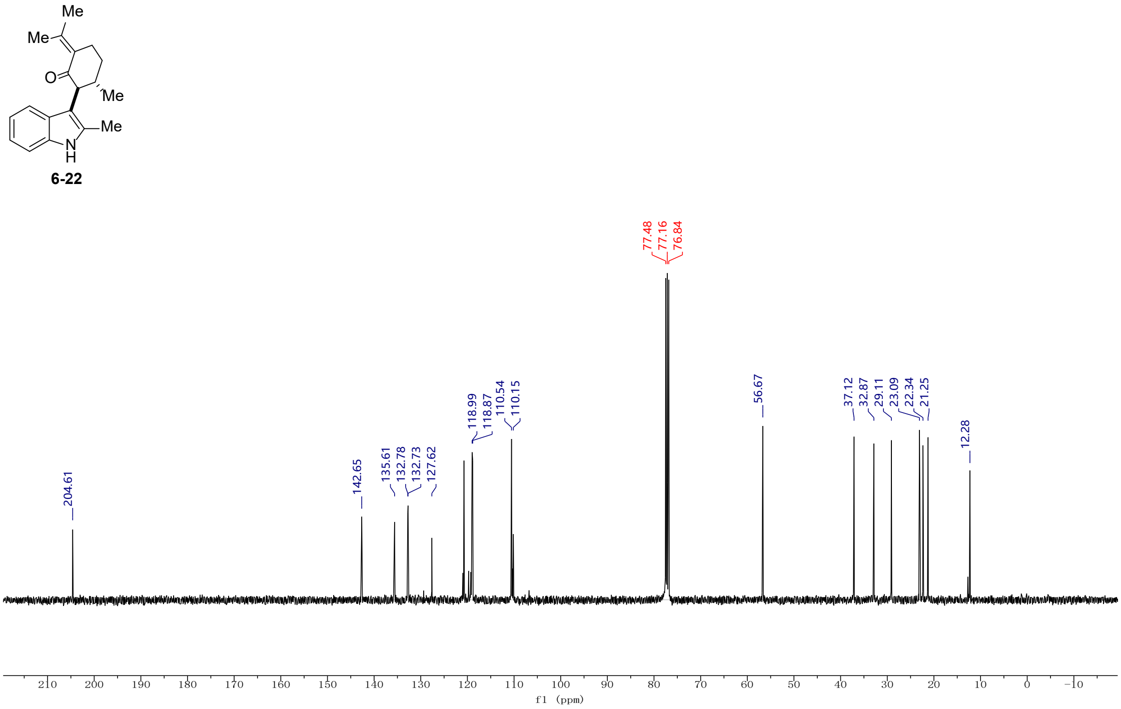

2018-2.3992.fid
LHL-1-74-1.2

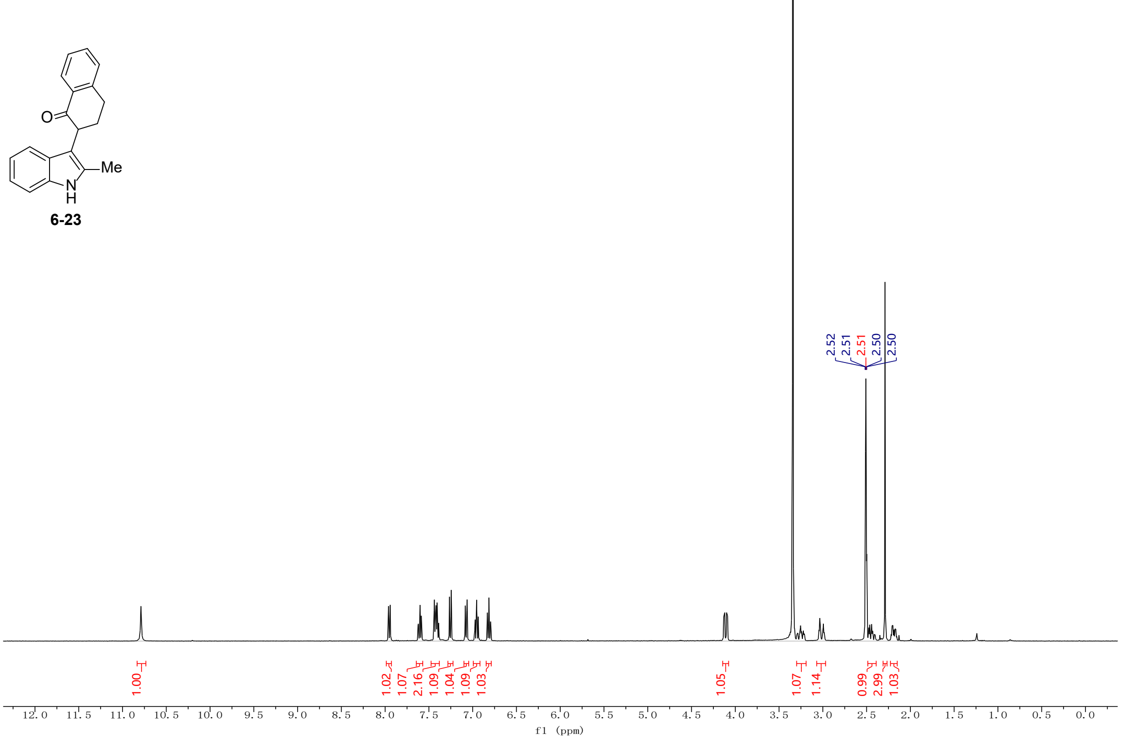

2018-2.4864.fid
LHL1-74-1.2

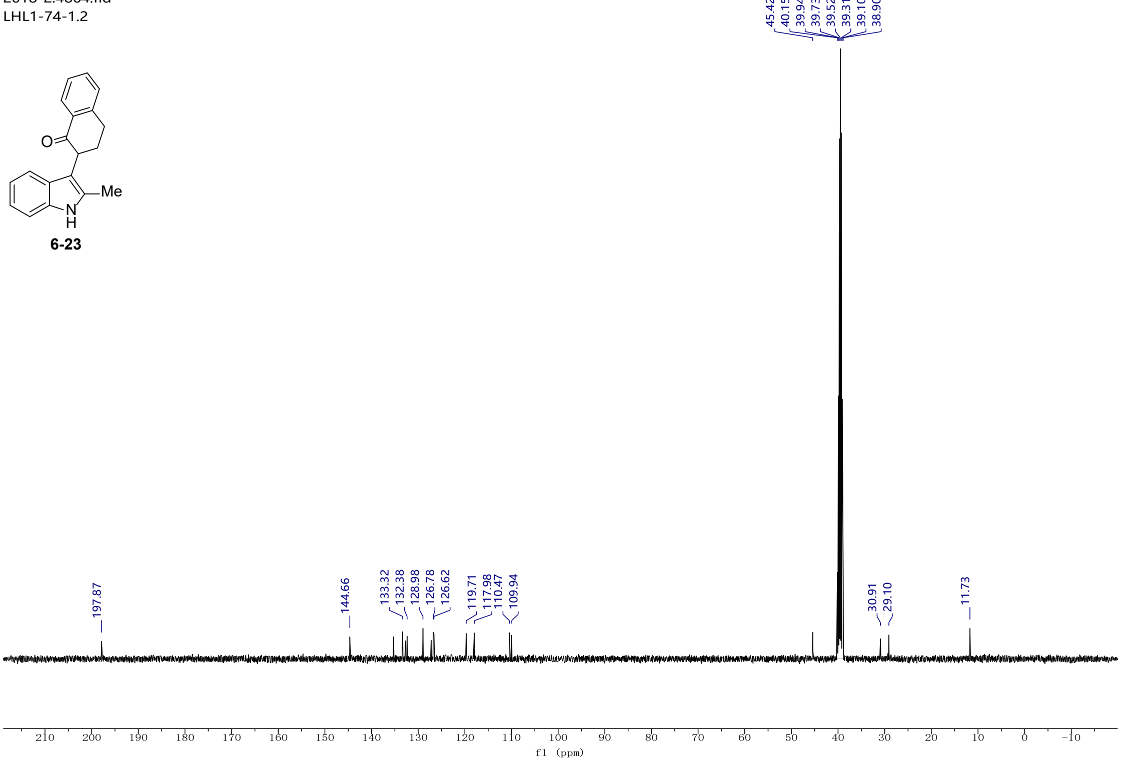

2018-2.7299.fid
lhl-2-26-2

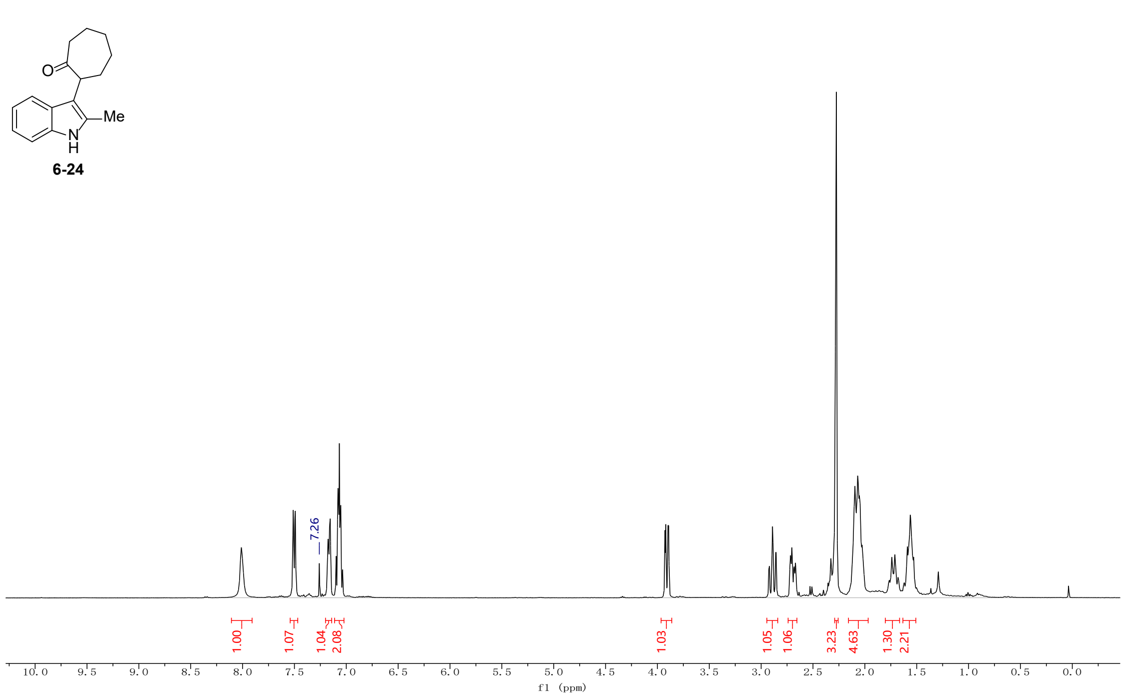

2018-2.8230.fid
LHL-2-26-2

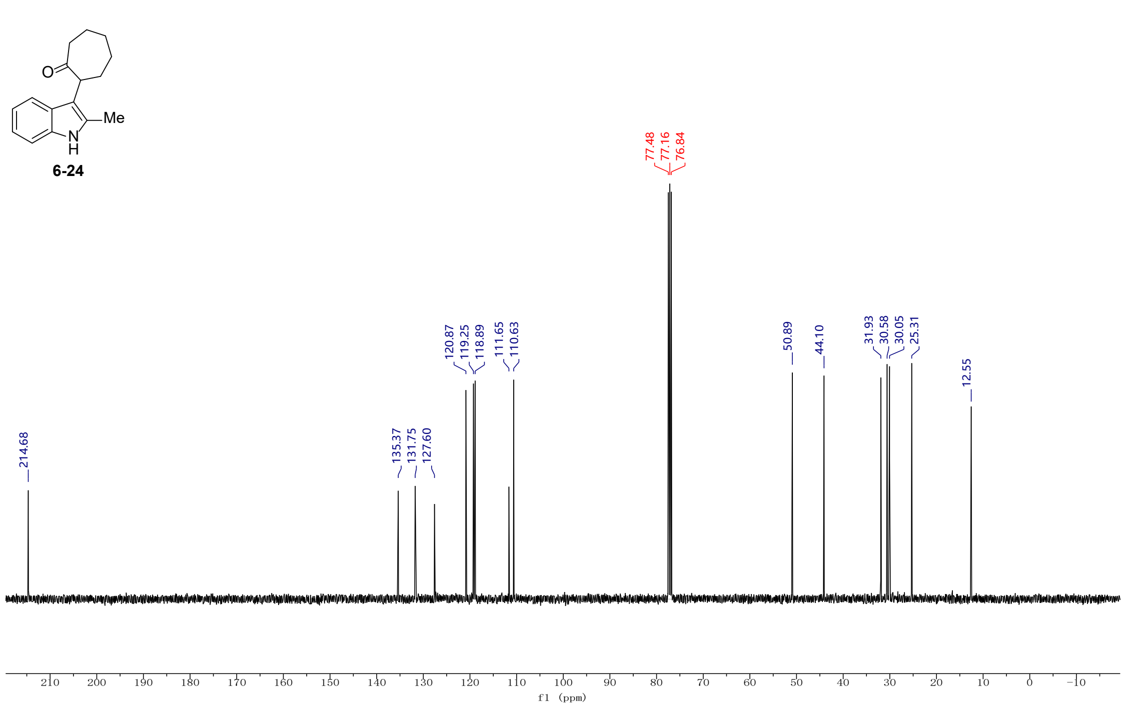

2018-2.13903.fid
LHL-2-80-2

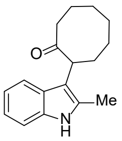

6-25

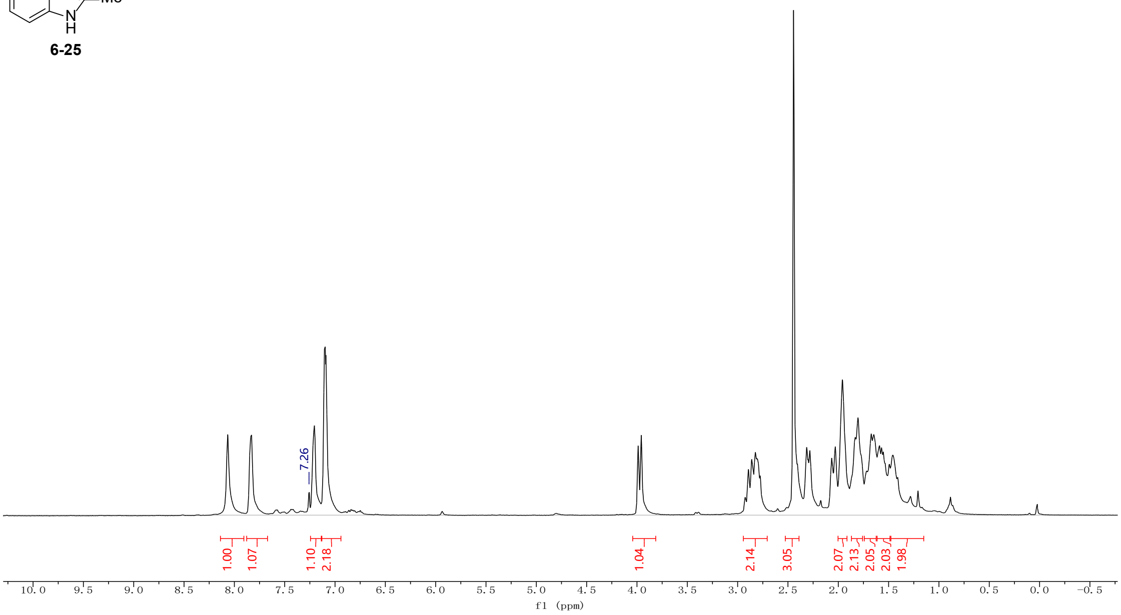

2018-2.14525.fid
LHL-2-80-2

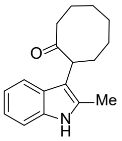

6-25

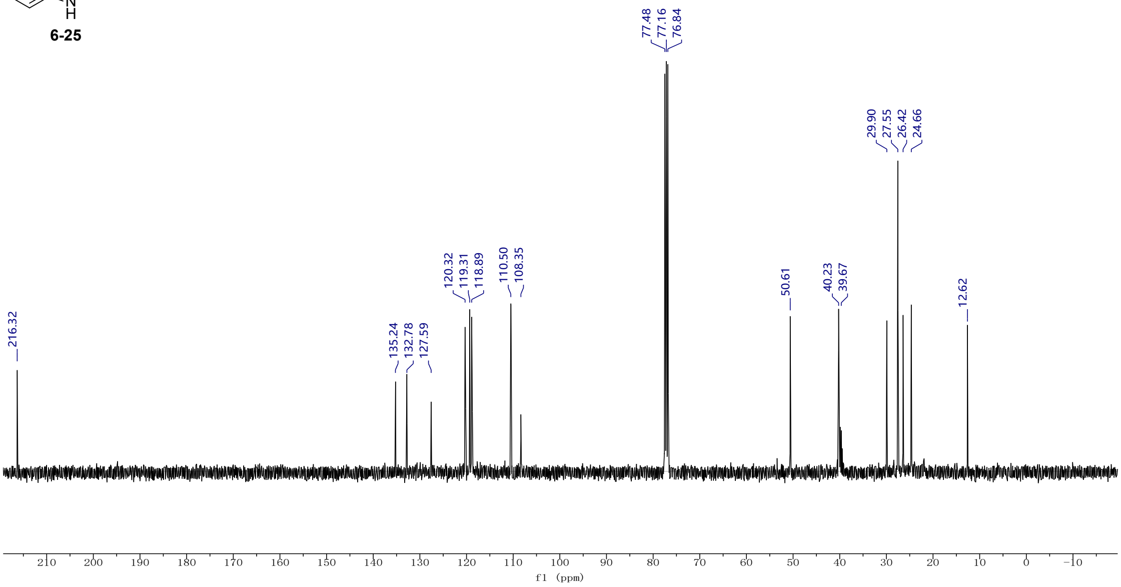

2018-2.7771.fid
LHL2-30-2.2

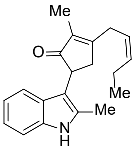

6-26

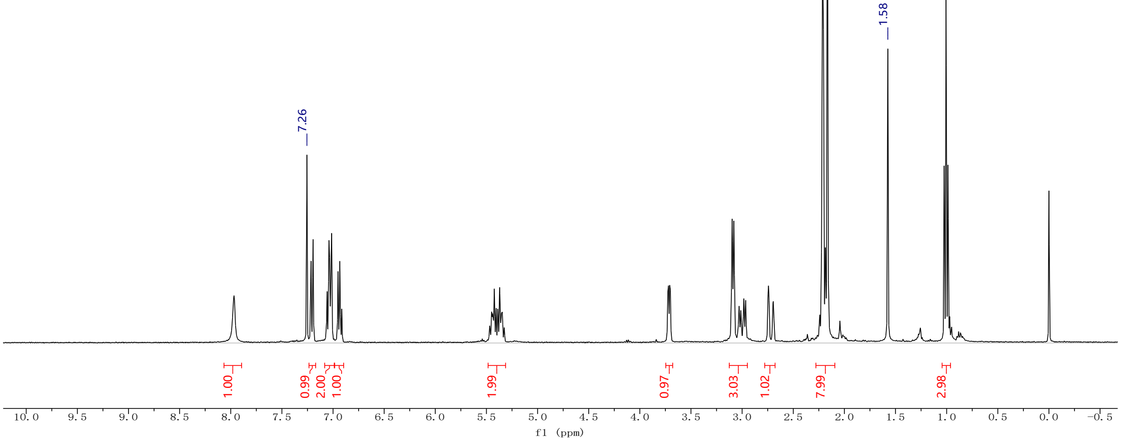

2019-1.2550.fid
LHL -2-30-2.2

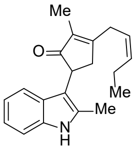

6-26

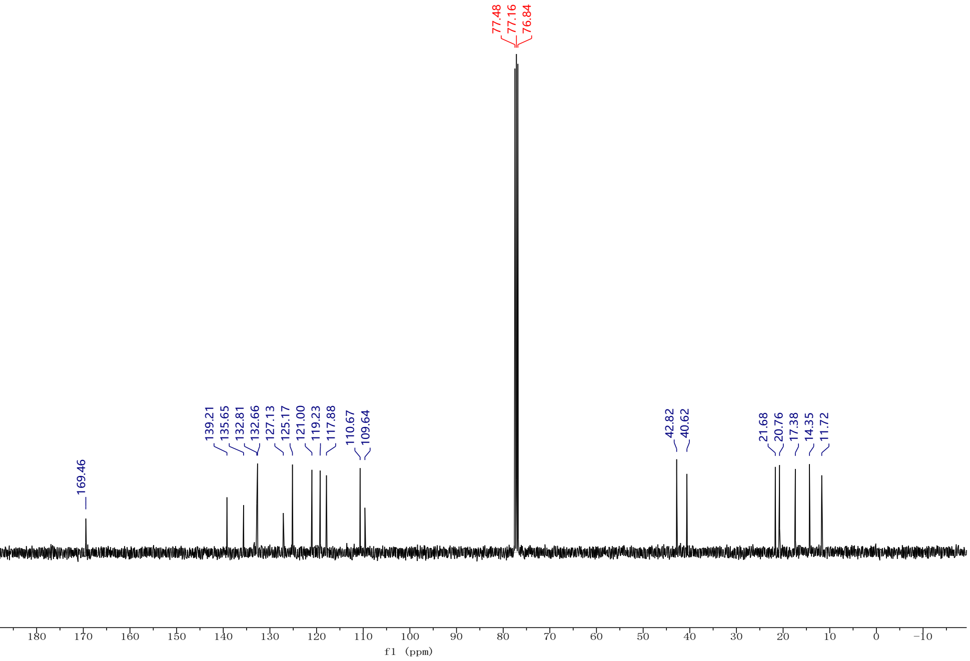

2018-2.13904.fid
LHL-2-81-1.2

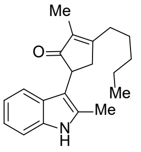

6-27

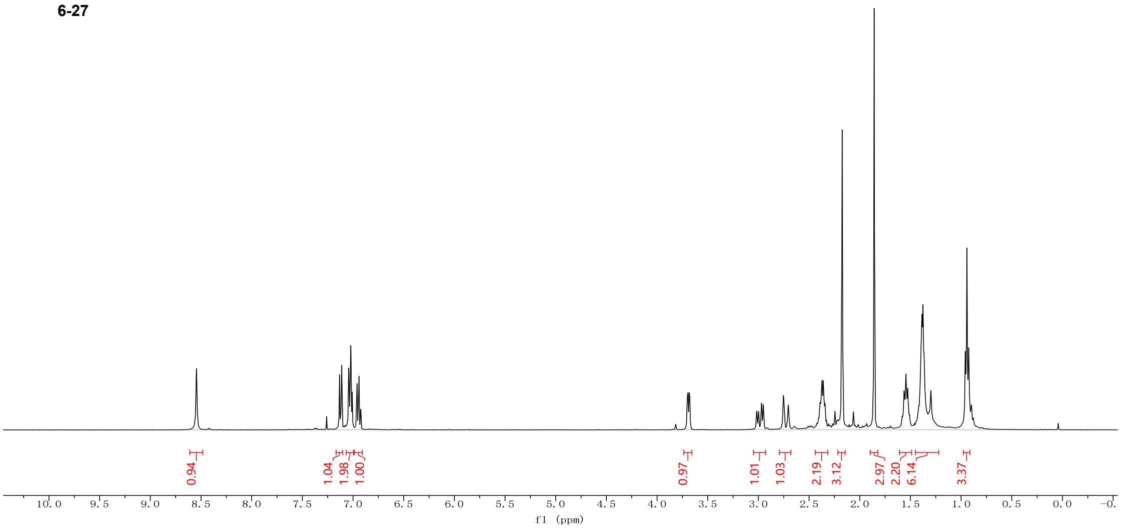

2018-2.14524.fid
LHL-2-81-1.2

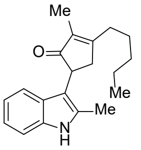

6-27

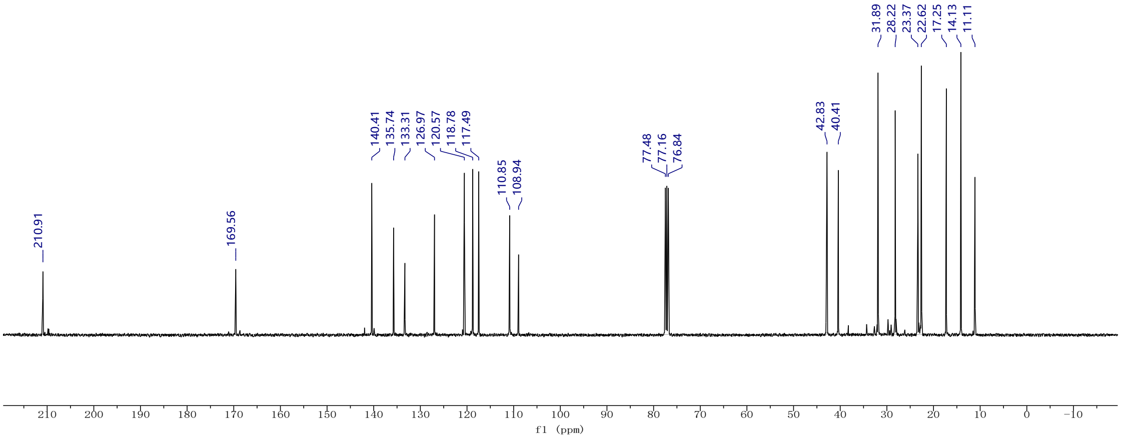

2019-1.3206.fid
LHL-2-35-1.2

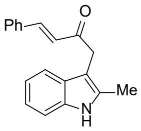

6-28

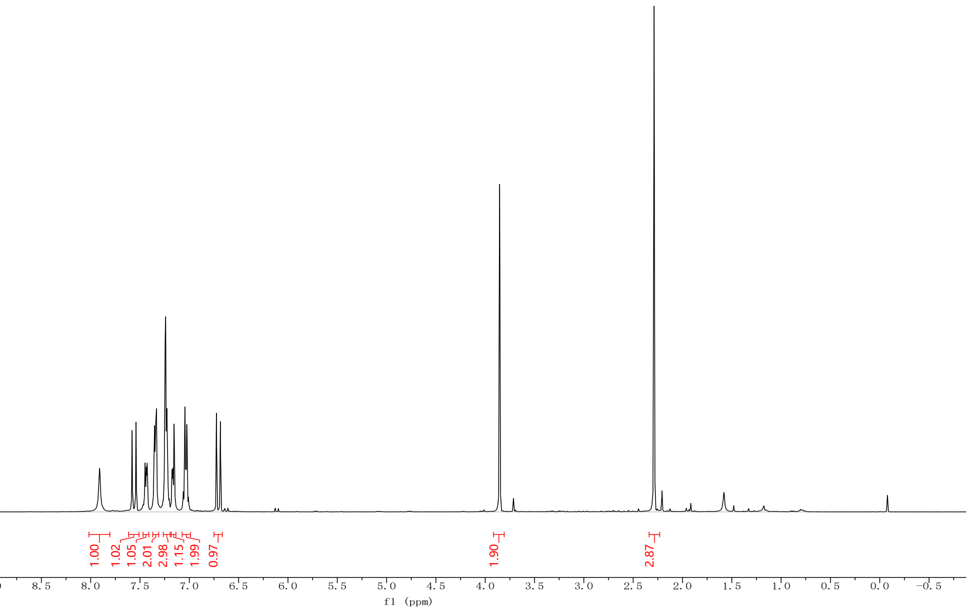

2019-1.3517.fid
LHL 2-35-1.2

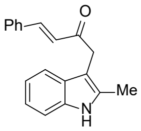

6-28

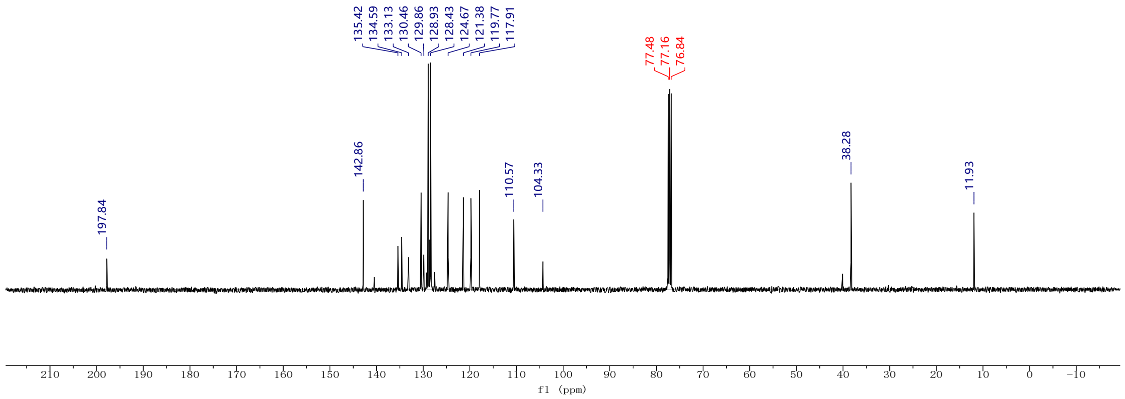

xiangguangya-20180629-002#.10.fid
LHL-1-83-1

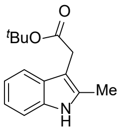

6-29

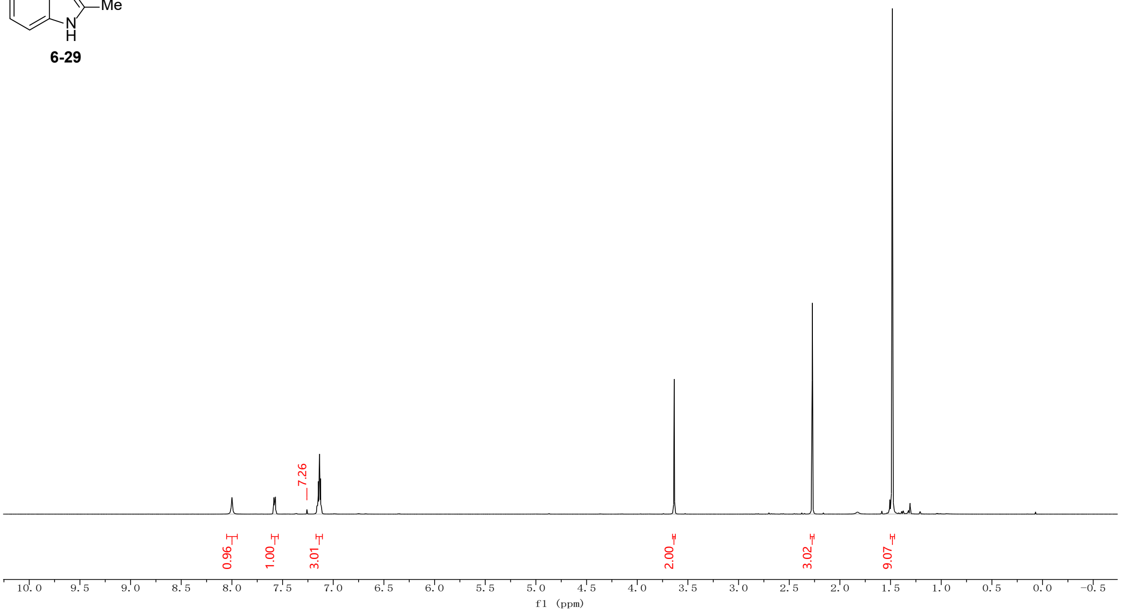

2019-1.7404.fid
LHL-1-83-1

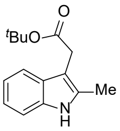

6-29

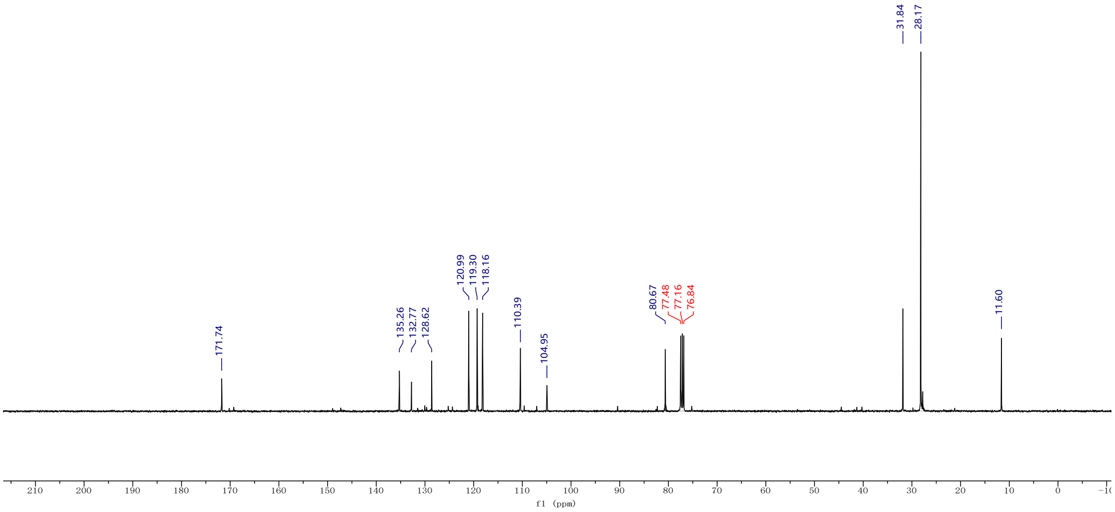

2018-2.11987.fid
LHL -2-64-1.1

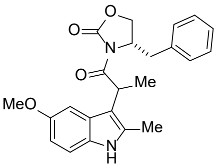

6-30 (1.9:1 dr)

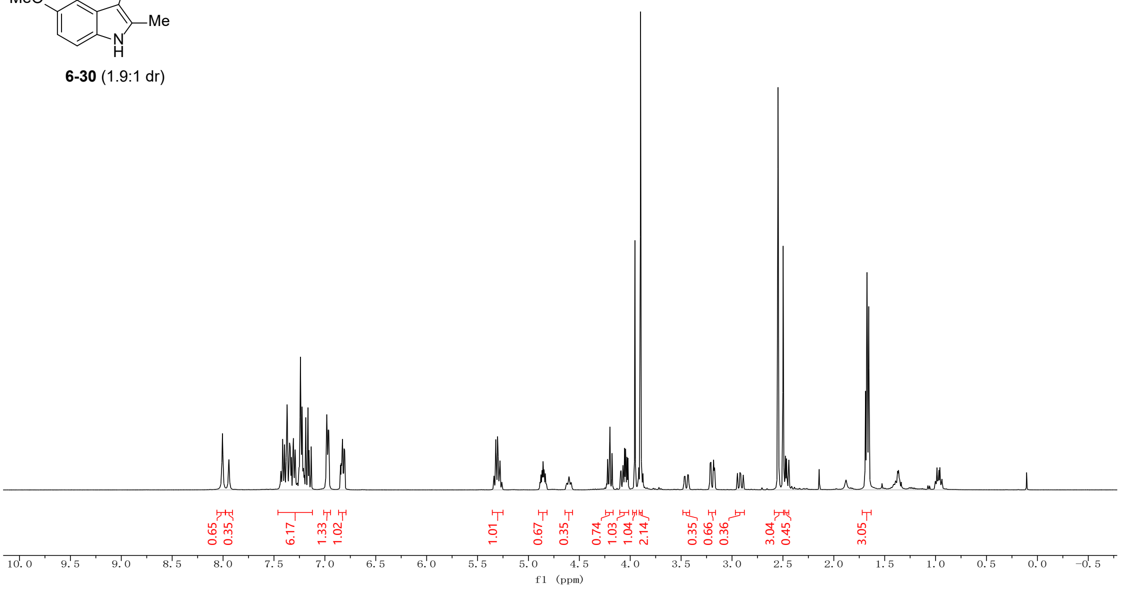

2018-2.12634.fid
LHL-2-64-1.1

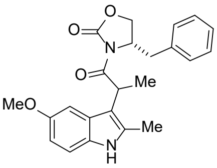

6-30 (1.9:1 dr)

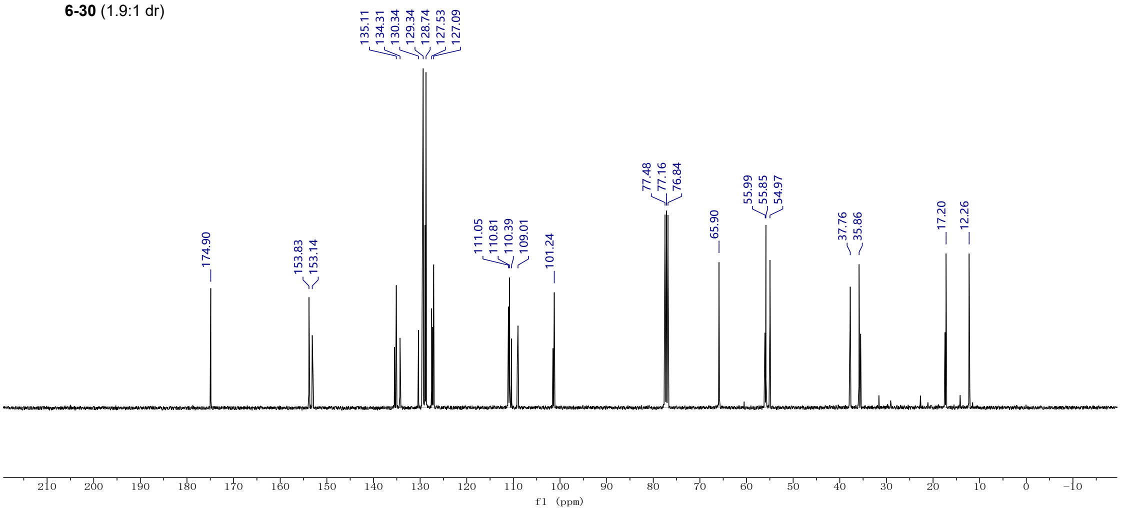

2018-2.14363.fid
LHL -2-83-2

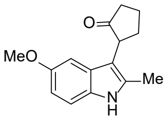

6-31

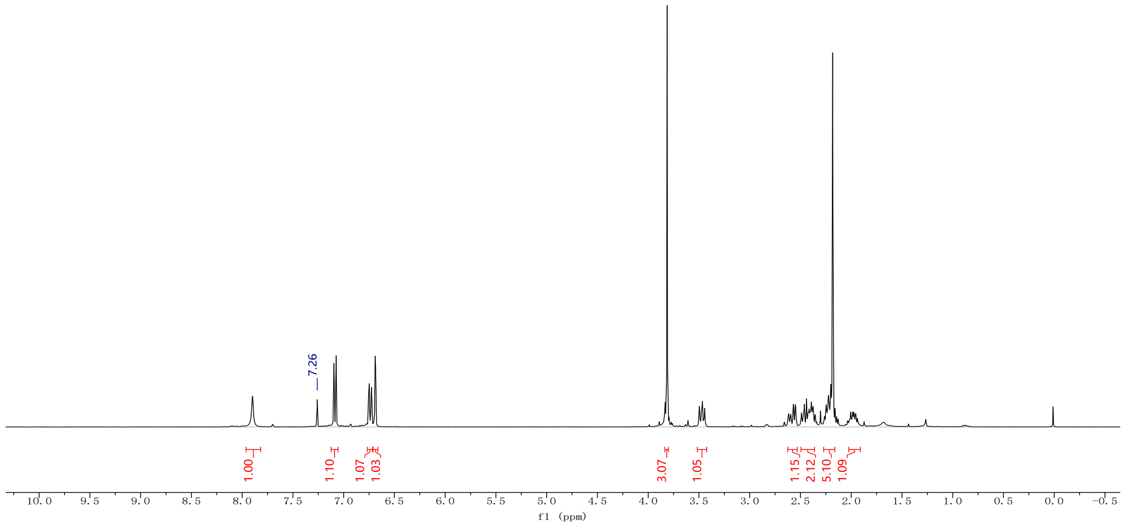

2019-1.6915.fid
LHL -2-83-2.2

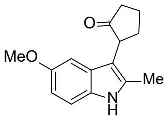

6-31

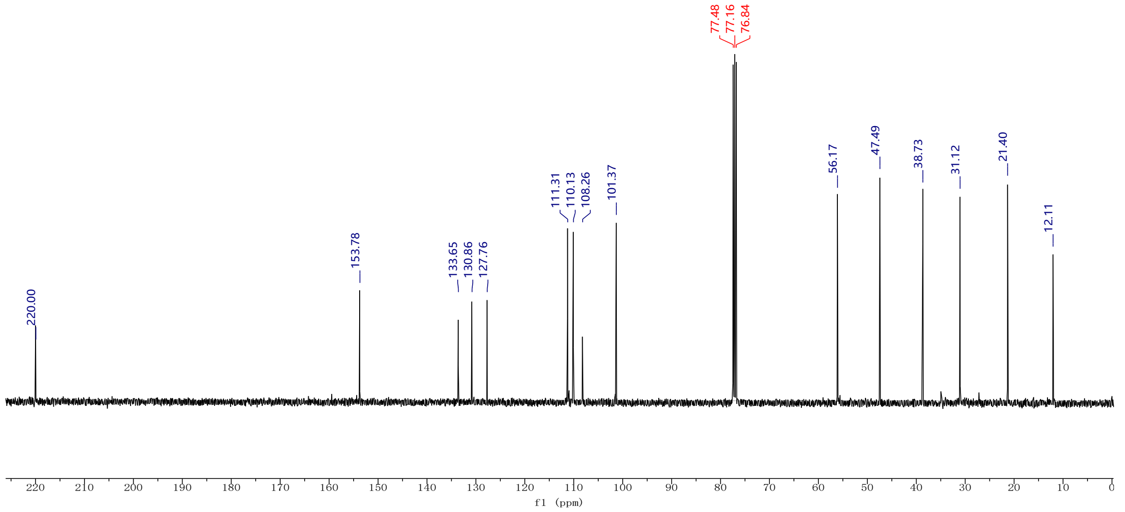

2018-2.14872.fid
LHL-2-84-2.2

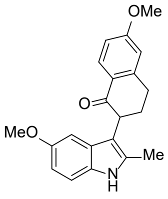

6-32

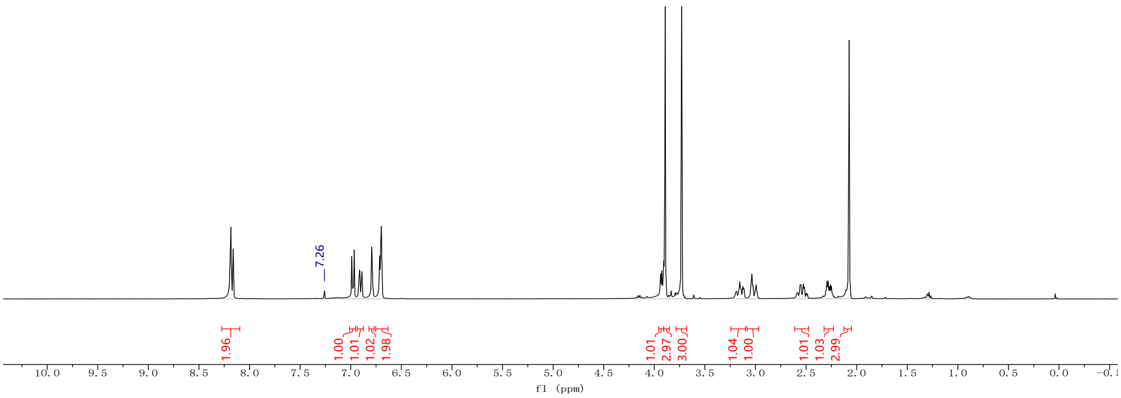

2018-2.15370.fid
LHL -2-84-2.2

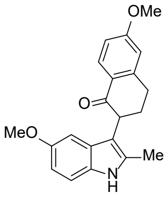

6-32

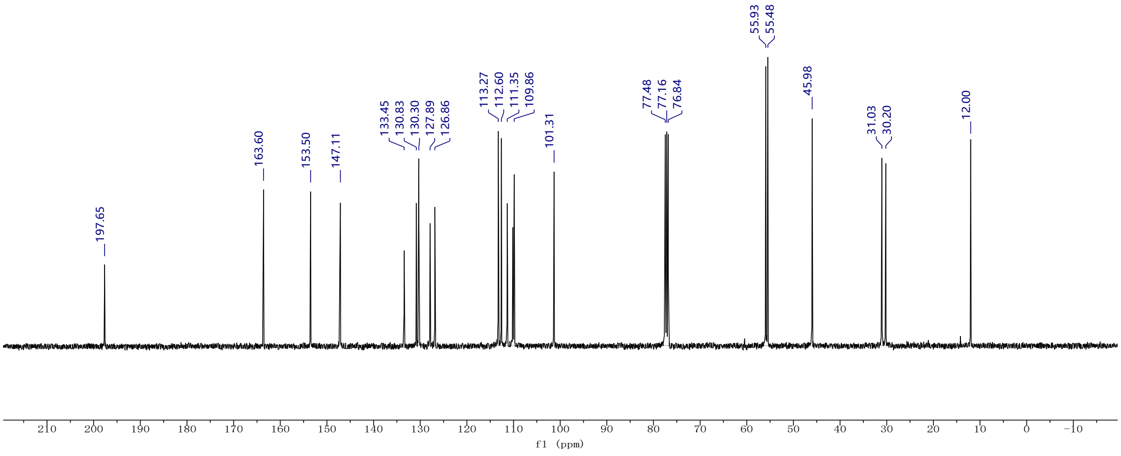

2019-1.2074.fid
LHL-3-14-2.1

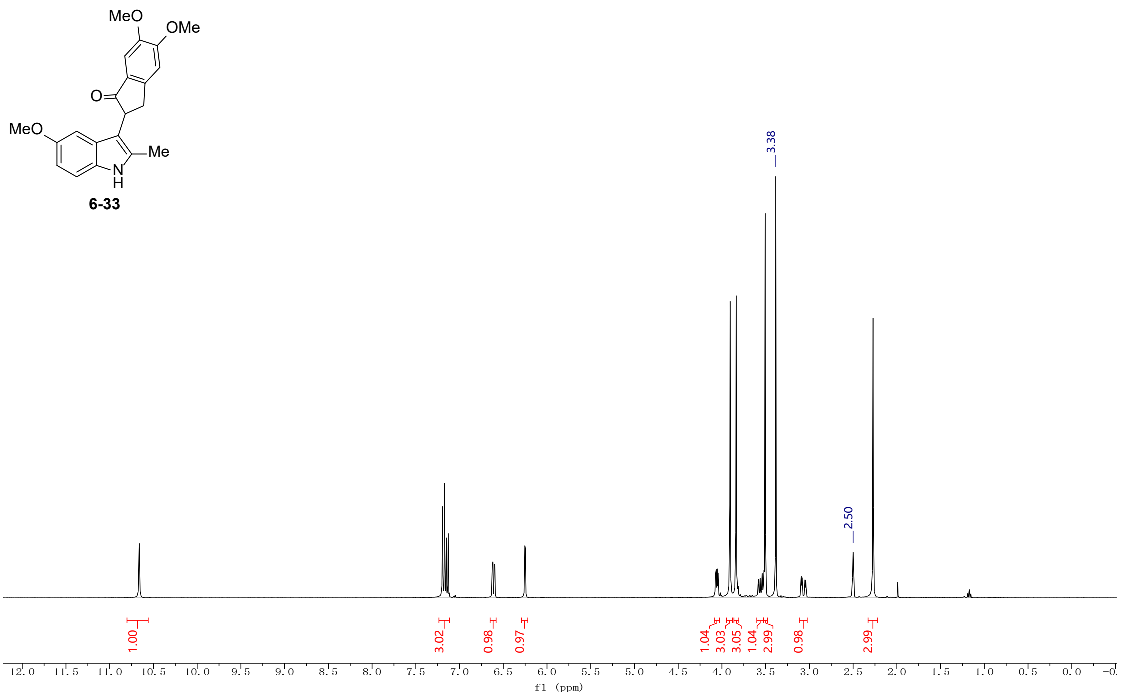

2019-1.2547.fid
LHL -3-14-2.1

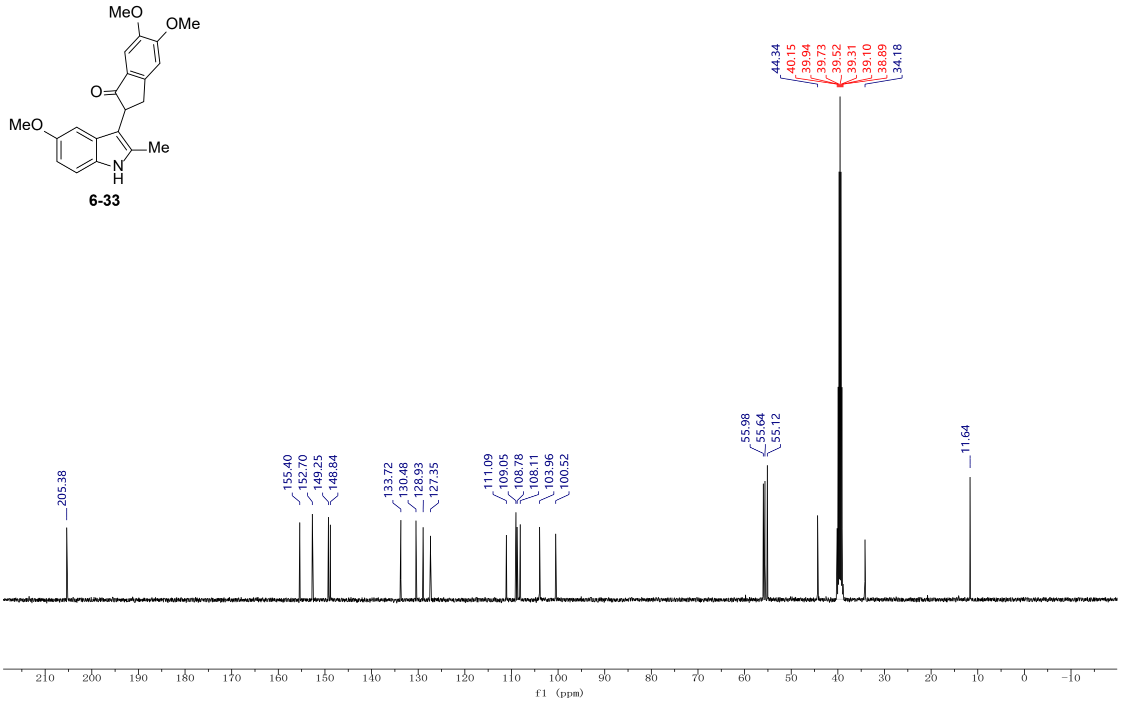

2019-1.3458.fid
LHL-2-89-2

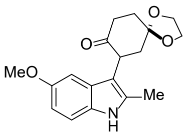

6-34

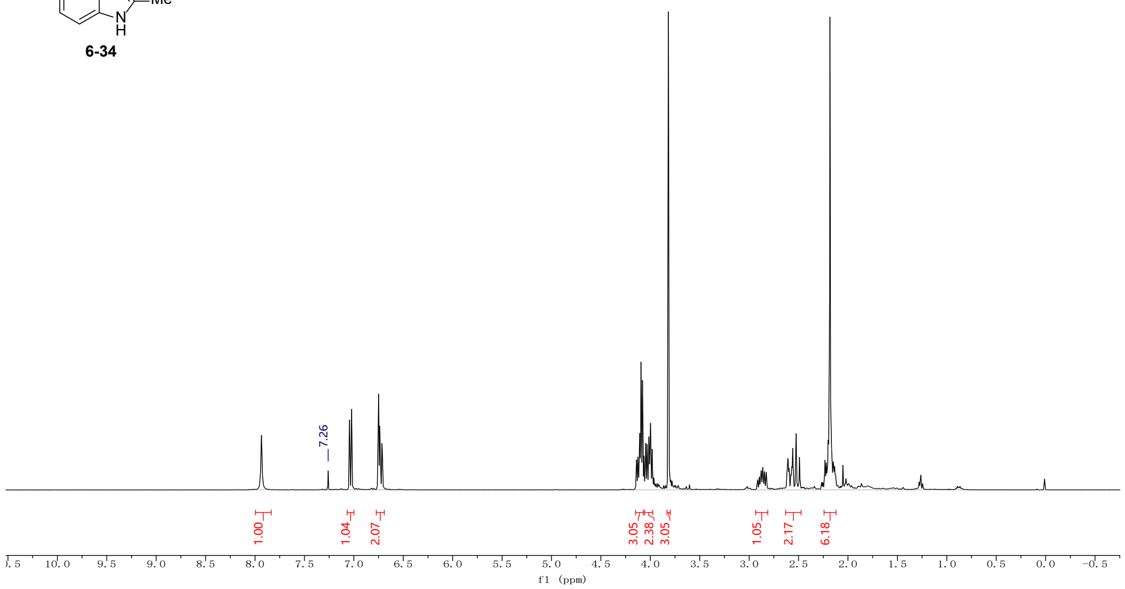

2019-1.3744.fid
LHL 2-89-2

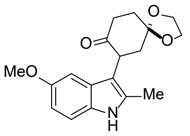

6-34

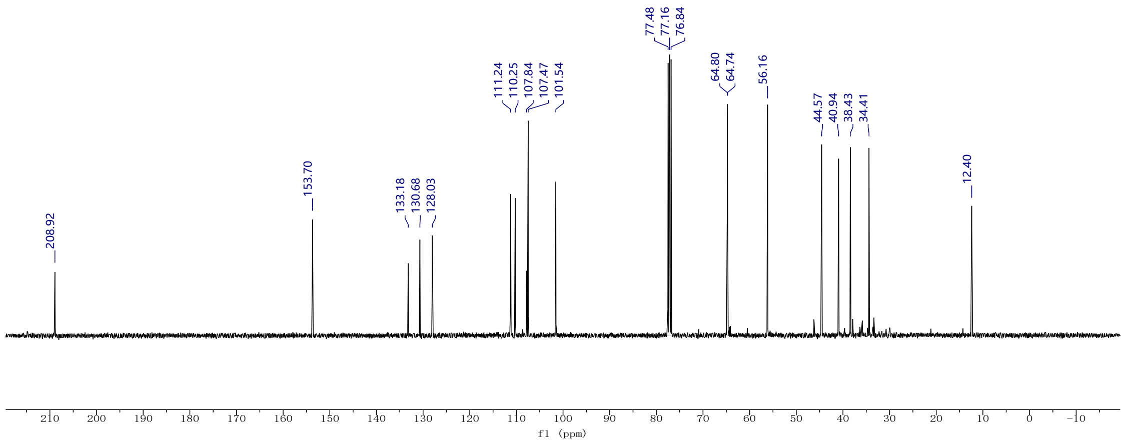

2018-2.13910.fid
LHL-2-82-1.2

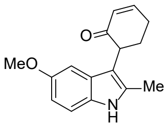

6-35

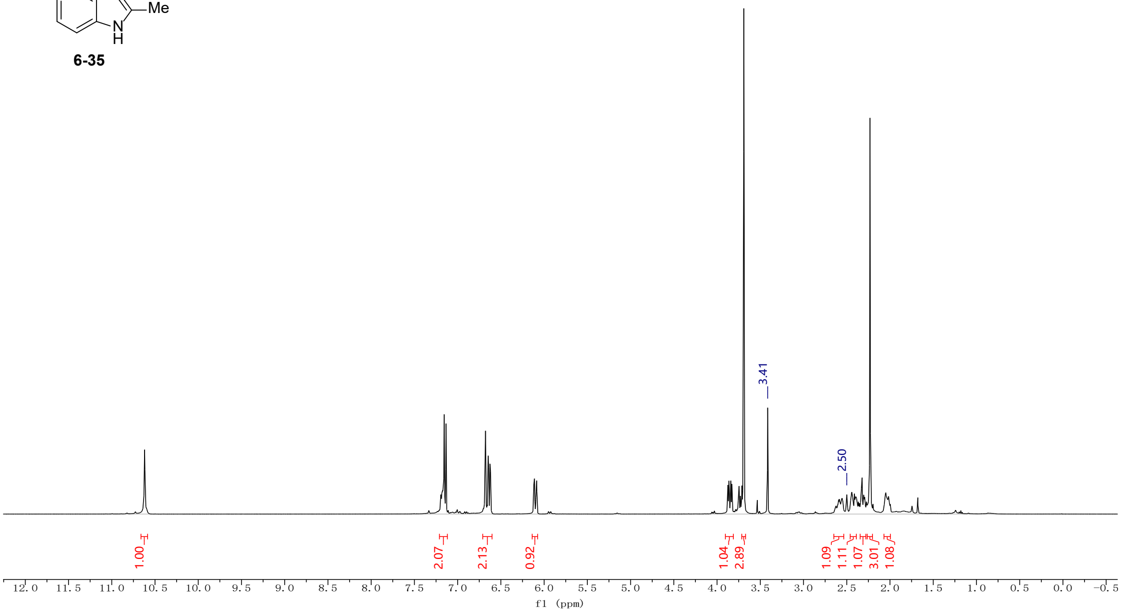

2018-2.14540.fid
LHL-2-82-1.2

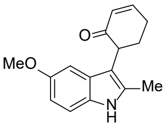

6-35

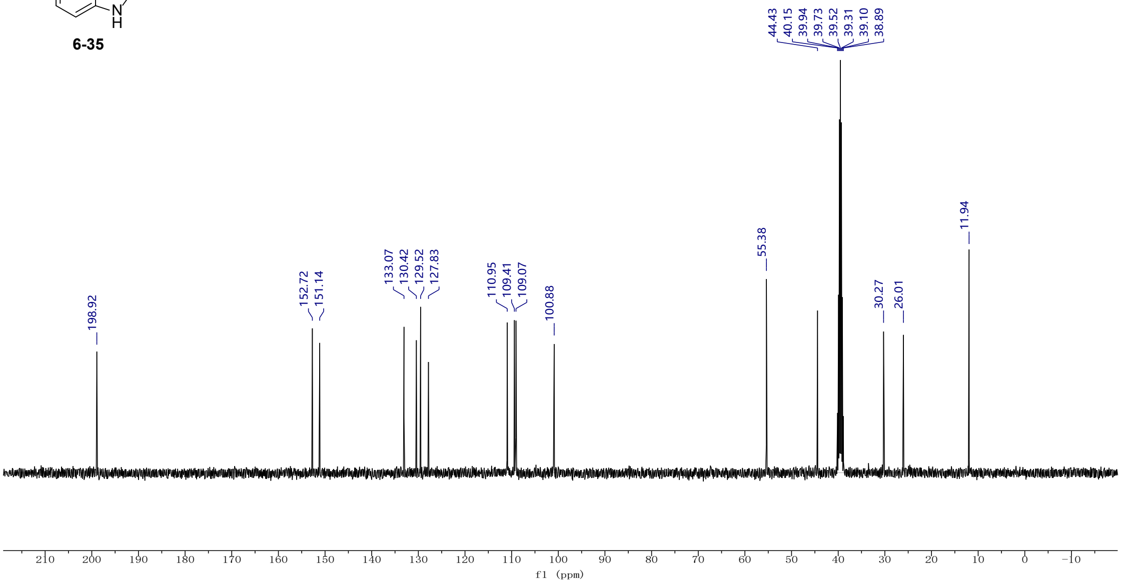

LHL-2-90-1.2

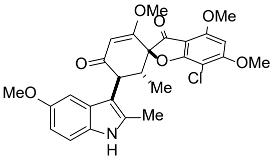

6-36

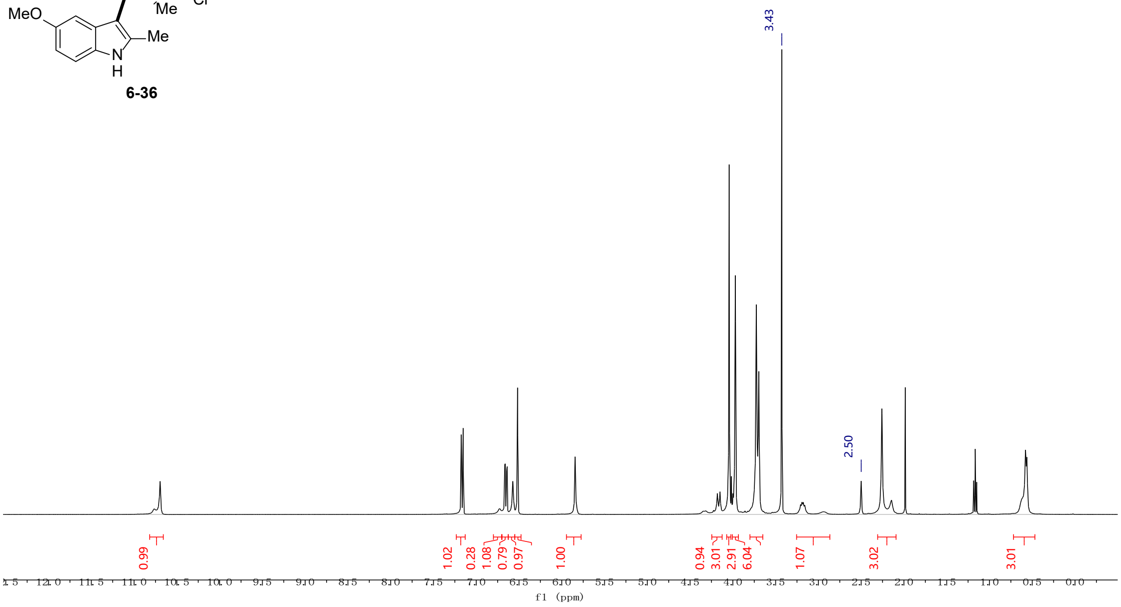

2019-1.717.fid
LHL -2-90-1.2

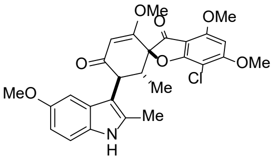

6-36

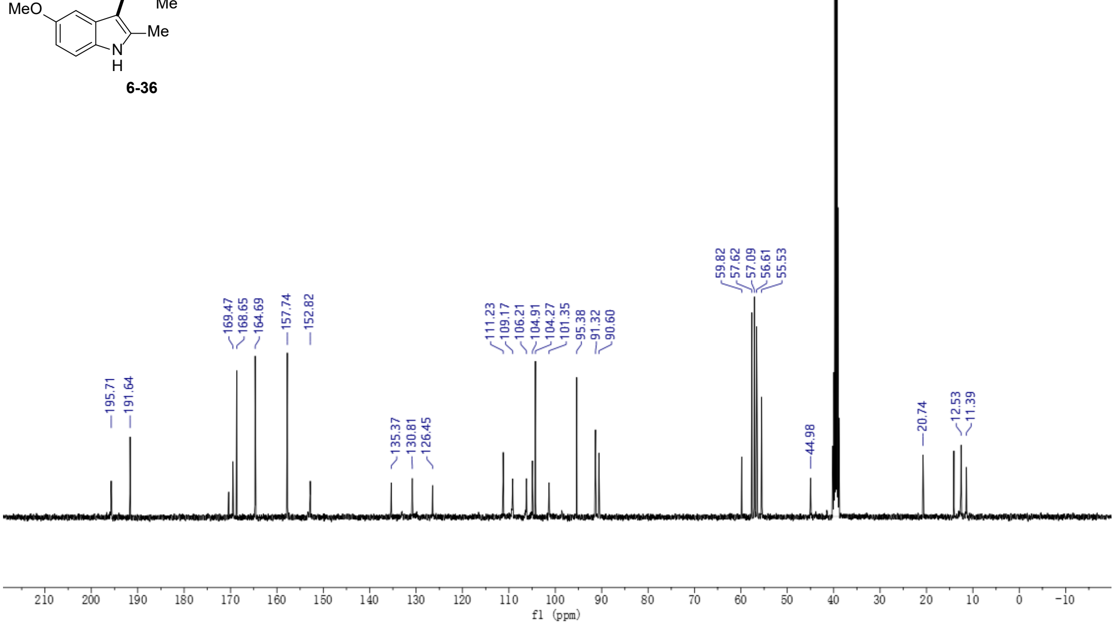

2018-2.14858.fid
LHL-2-88-1.1

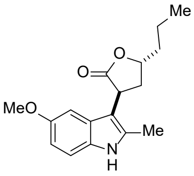

6-37-1 (isomer 1)

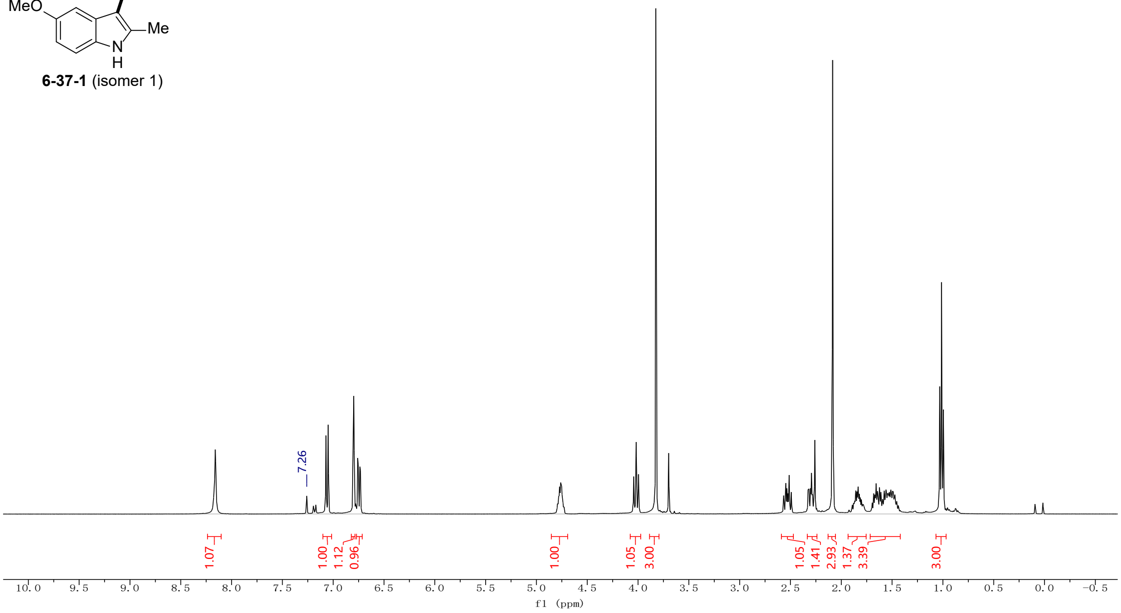

2018-2.15367.fid
LHL 2-88-1.1

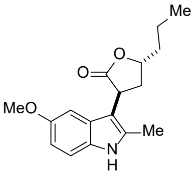

6-37-1 (isomer 1)

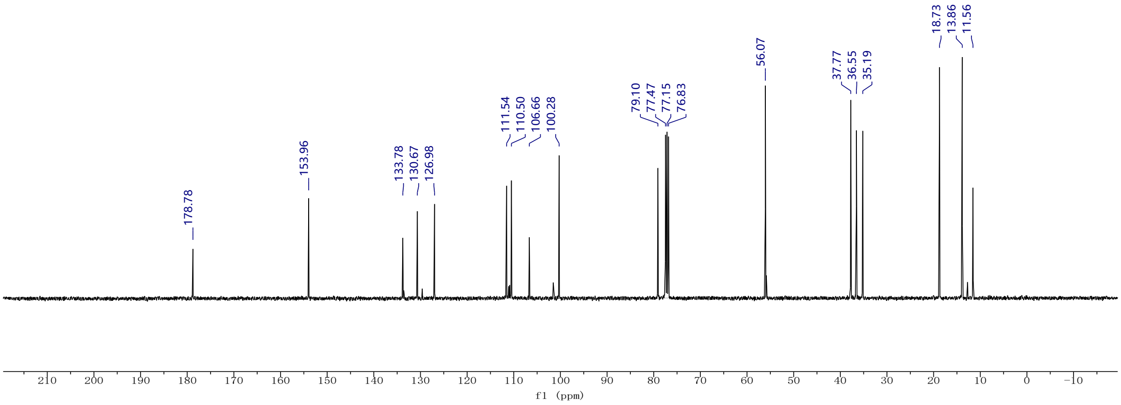

2018-2.14860.fid
LHL-2-88-1.2

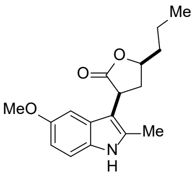

6-37-2 (isomer 2)

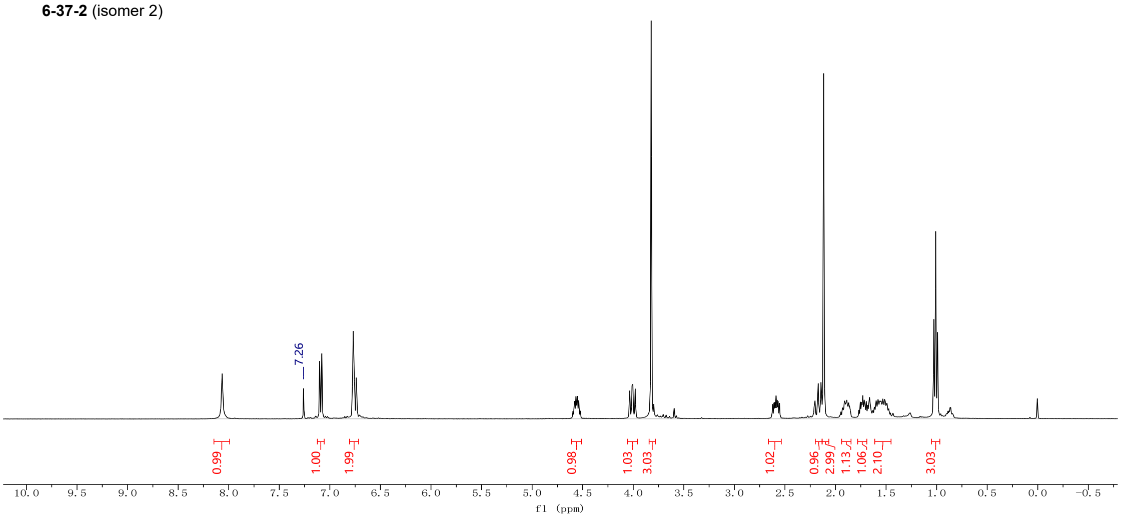

2018-2.15375.fid
LHL-2-88-1.2

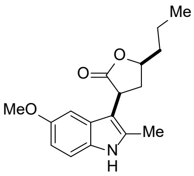

6-37-2 (isomer 2)

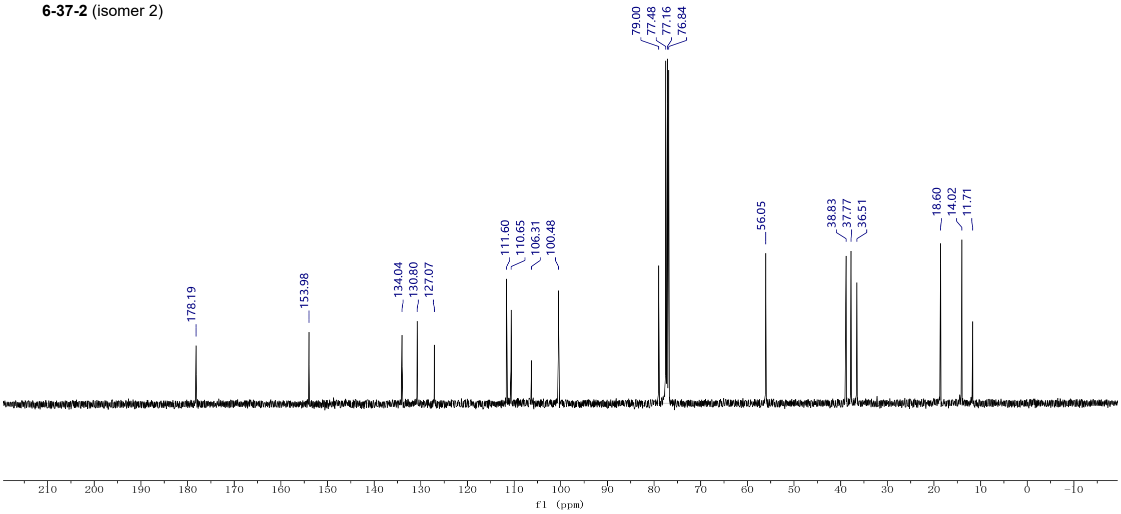

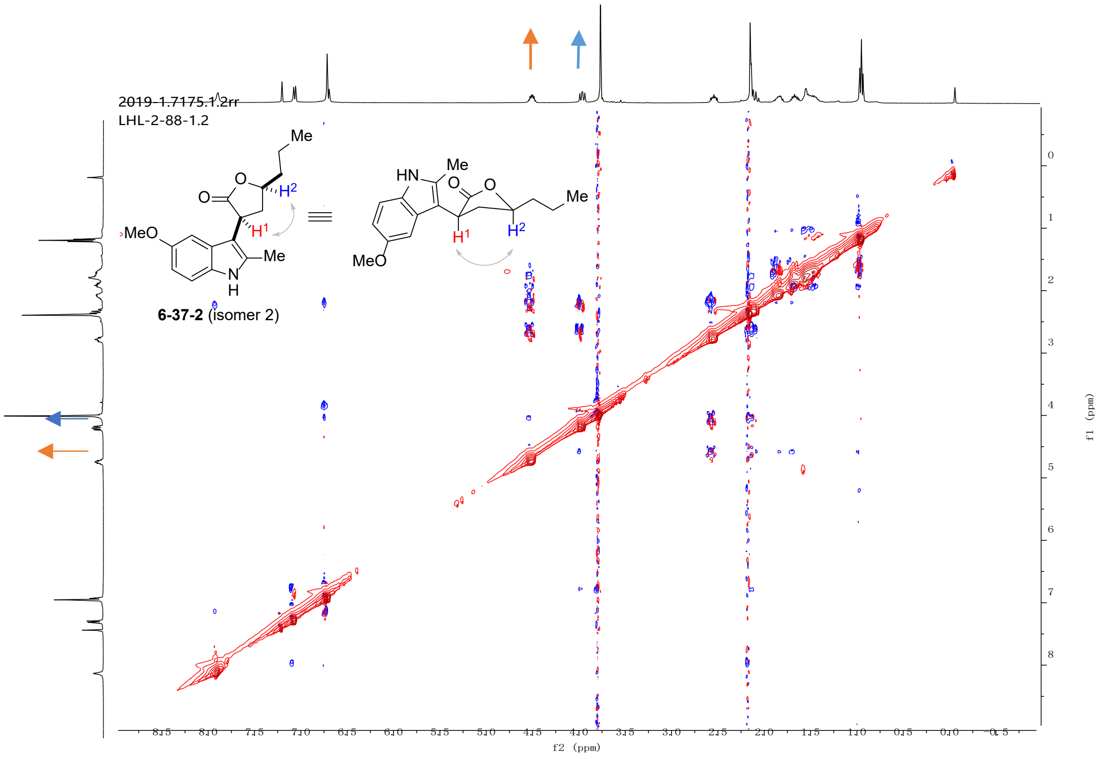

LHL-3-6-2

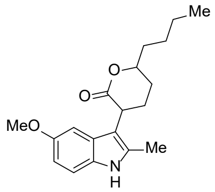

6-38 (two diastereomers)

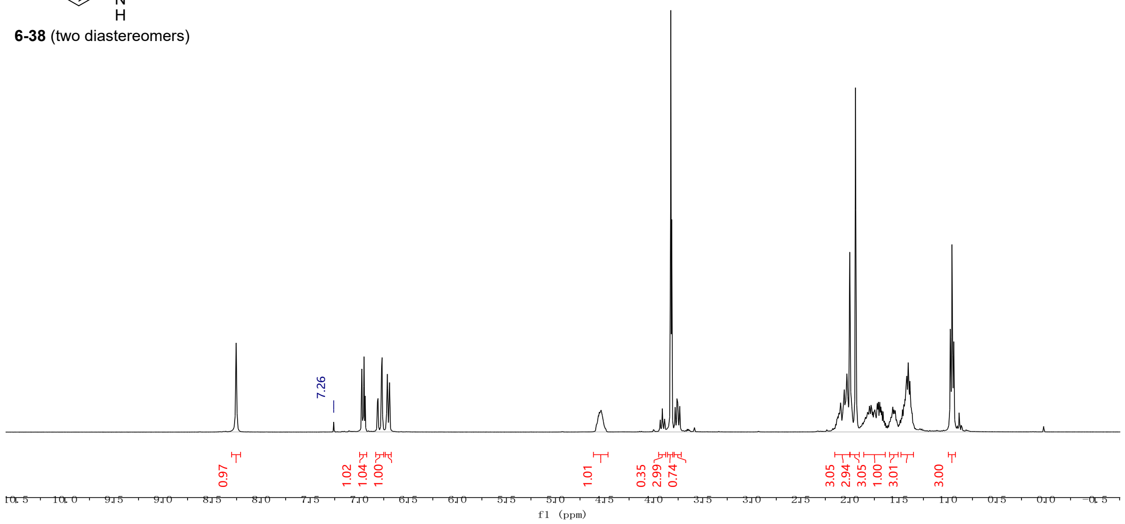

2019-1.859.fid
LHL-3-6-2

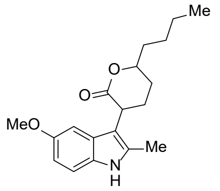

6-38 (two diastereomers)

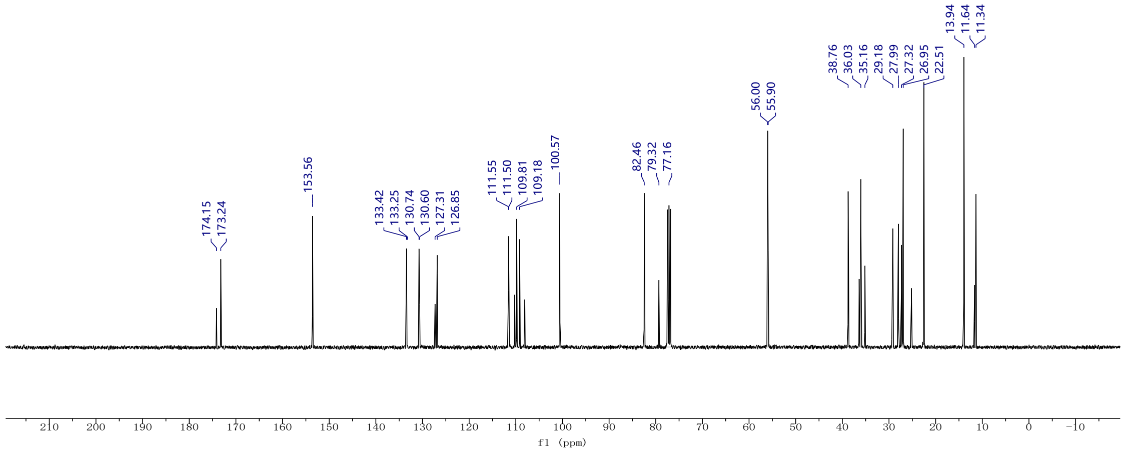

2019-1.675.fid
LHL-3-6-1

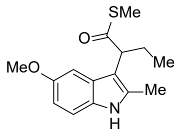

6-39

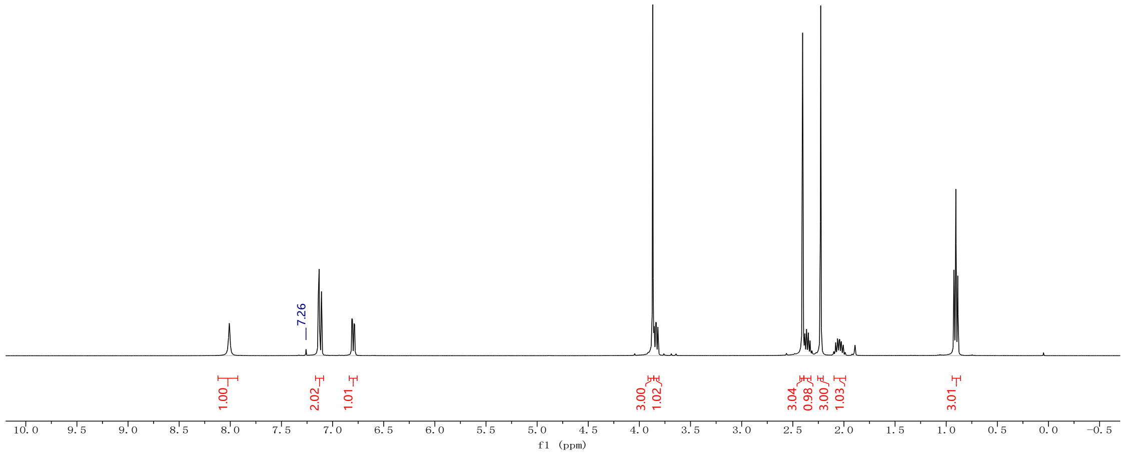

2019-1.860.fid
LHL-3-6-1

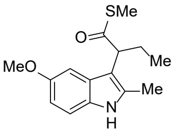

6-39

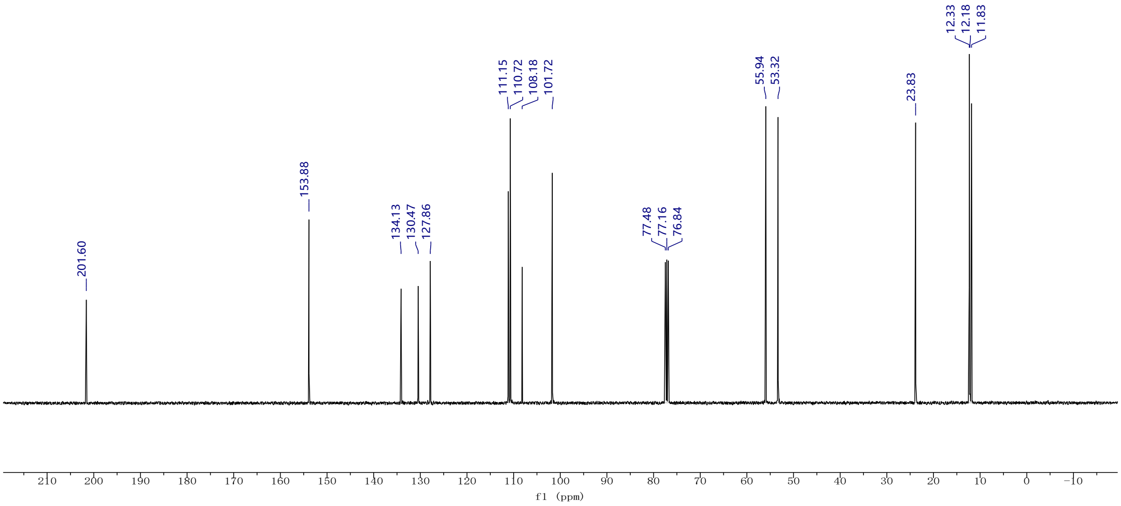

2018-2.14861.fid
LHL-2-89-1

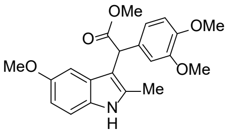

6-40

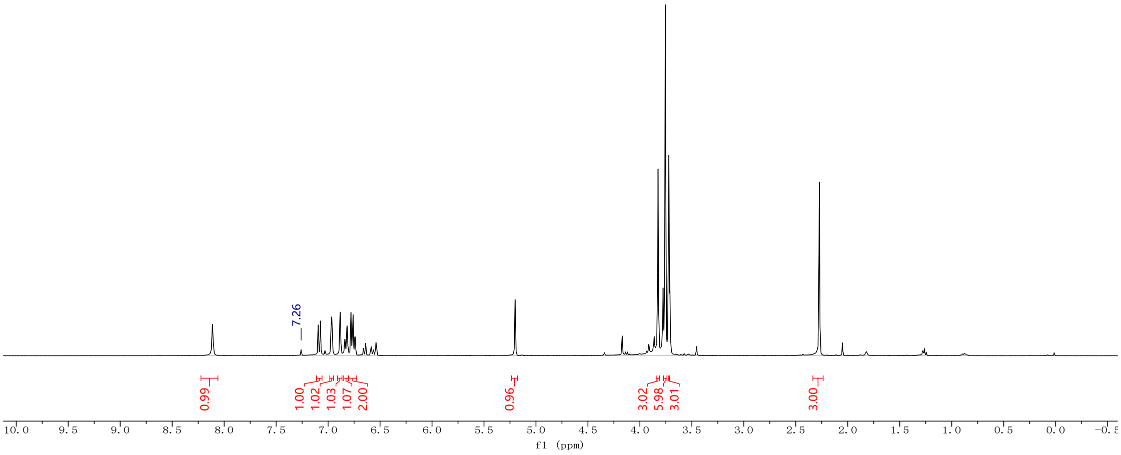

2018-2.15374.fid
LHL -2-89-1

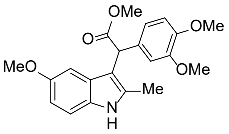

6-40

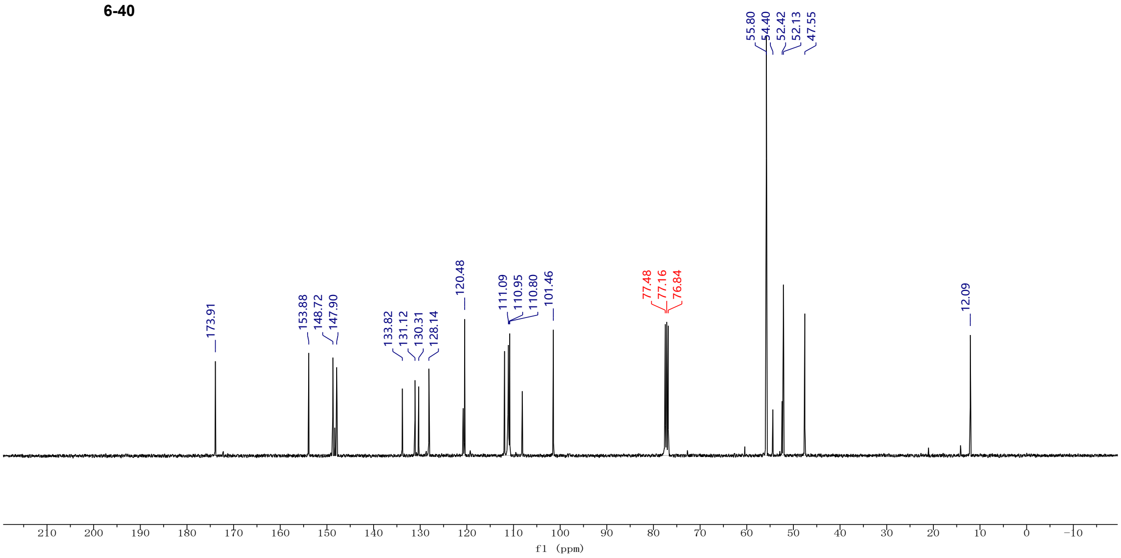

2018-2.14873.fid
LHL-2-86-1.1

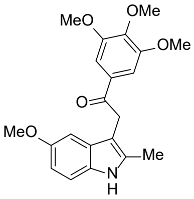

6-41

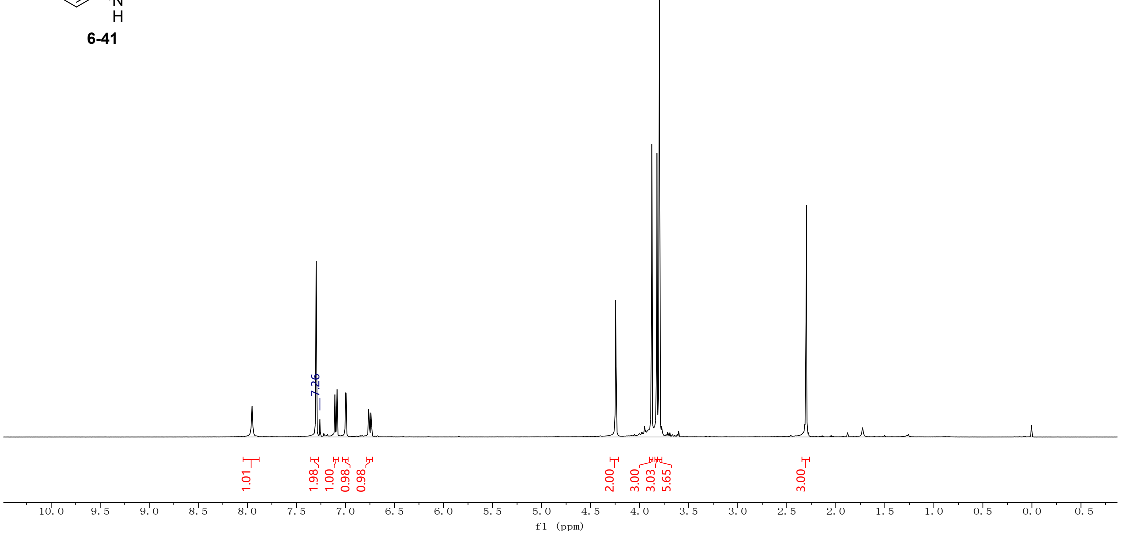

2018-2.15368.fid
LHL -2-86-1.1

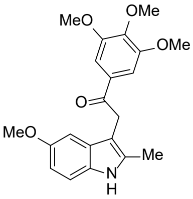

6-41

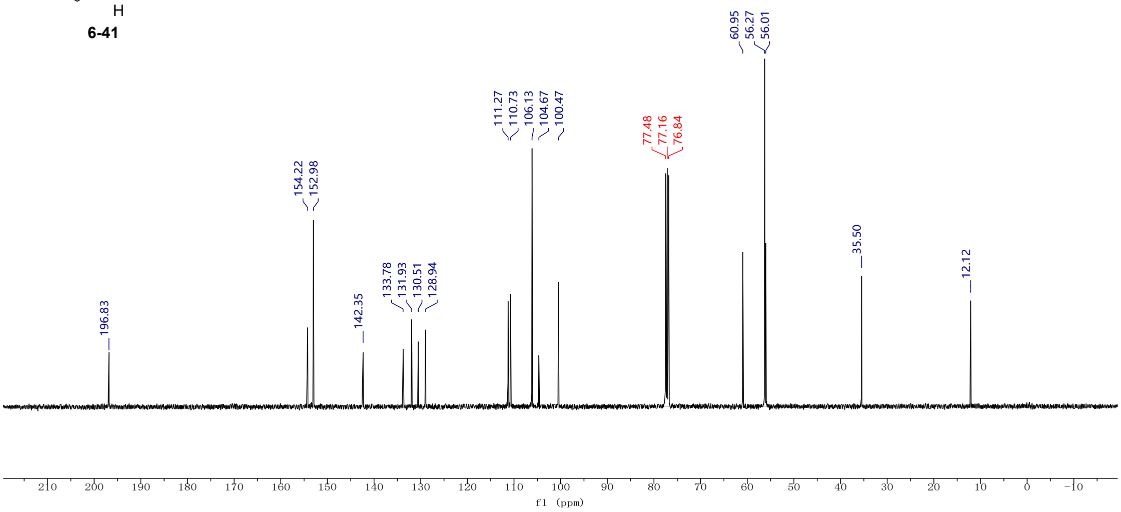

2018-2.14856.fid
LHL-2-85-2.1

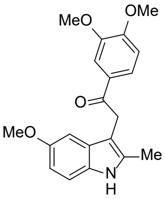

6-42

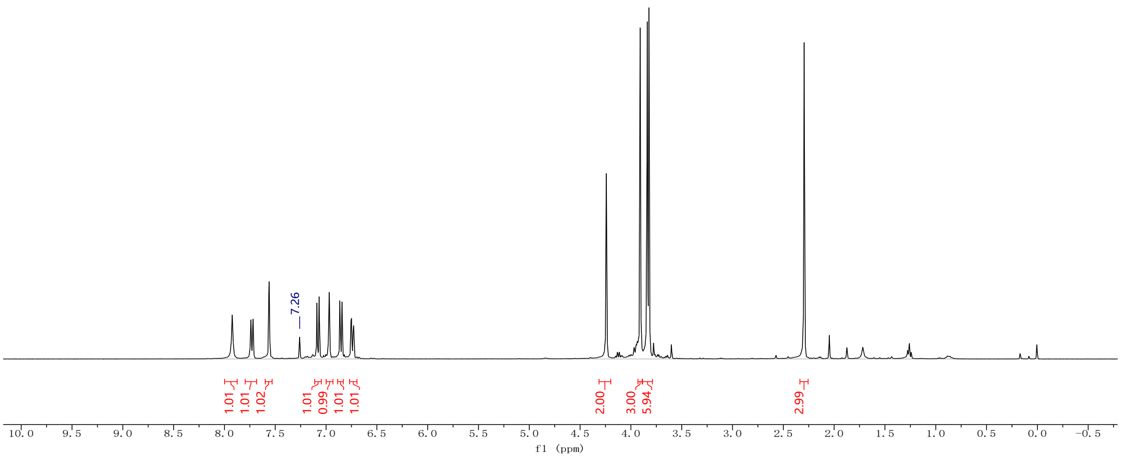

2018-2.15371.fid
LHL -2-85-2.1

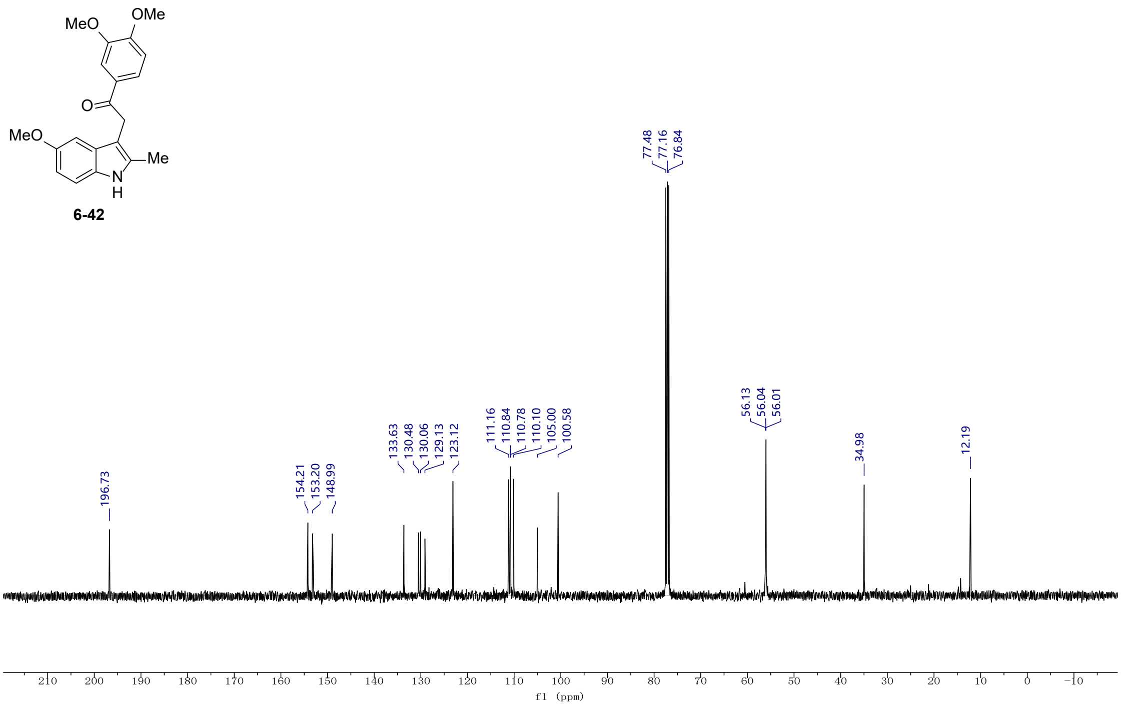

2018-2.3997.fid
LHL-1-73-2.2

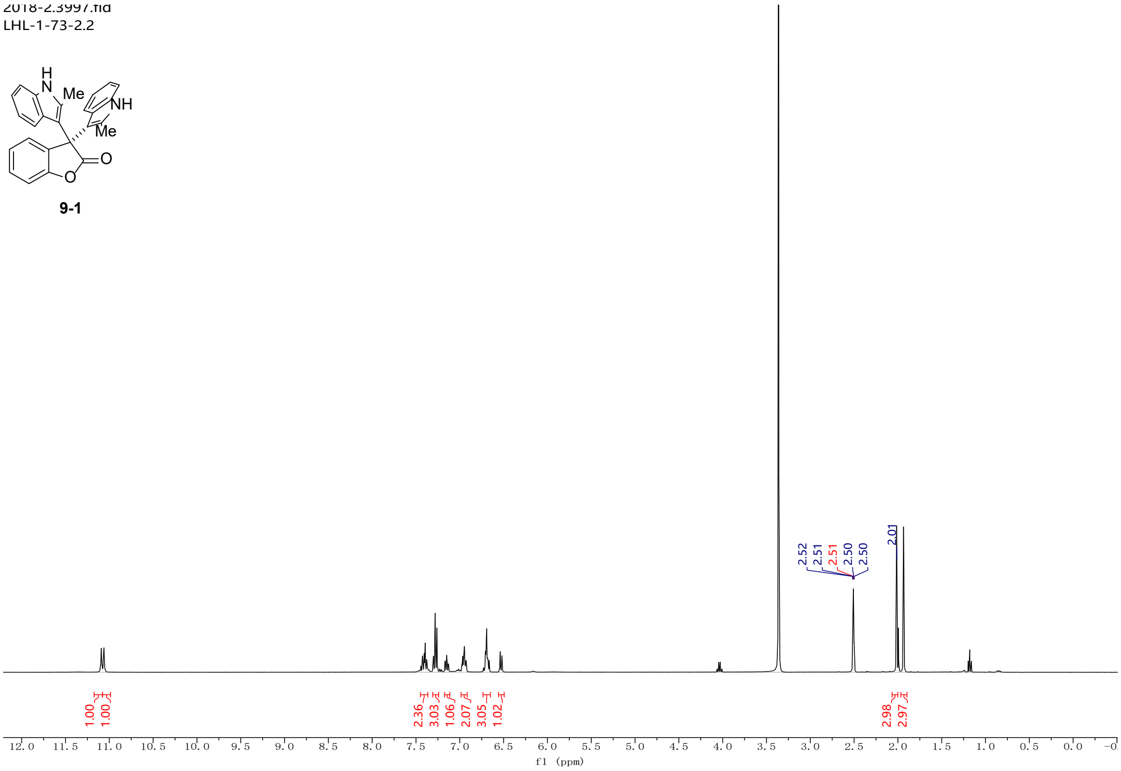

2019-1.3268.fid
LHL-1-73-2.2

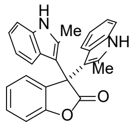

9-1

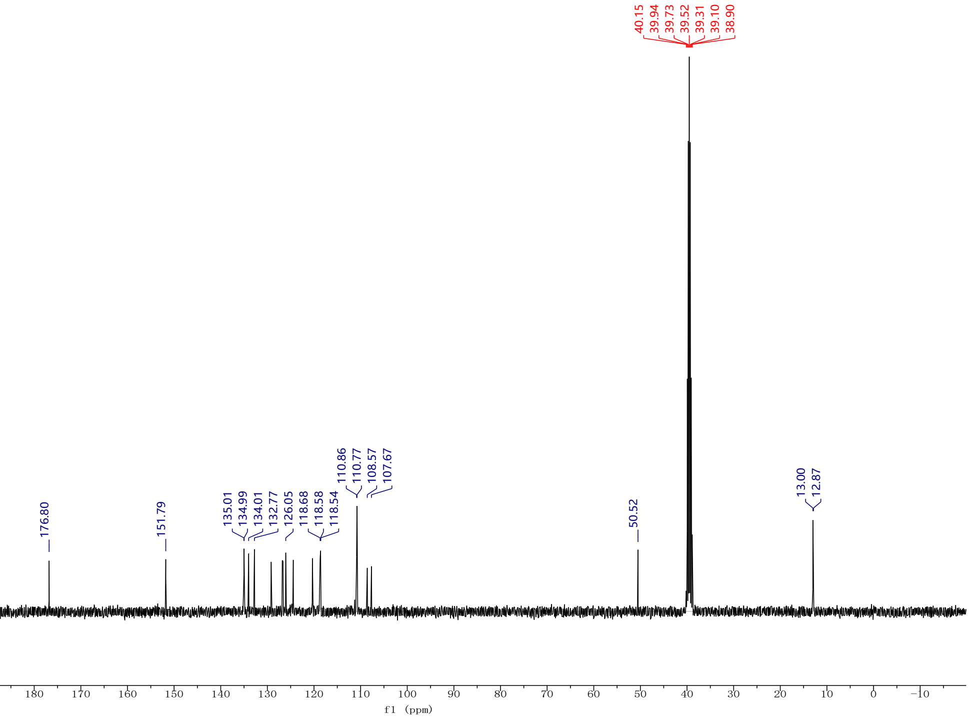

2018-2.14855.fid
LHL-2-87-1

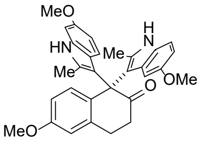

9-2

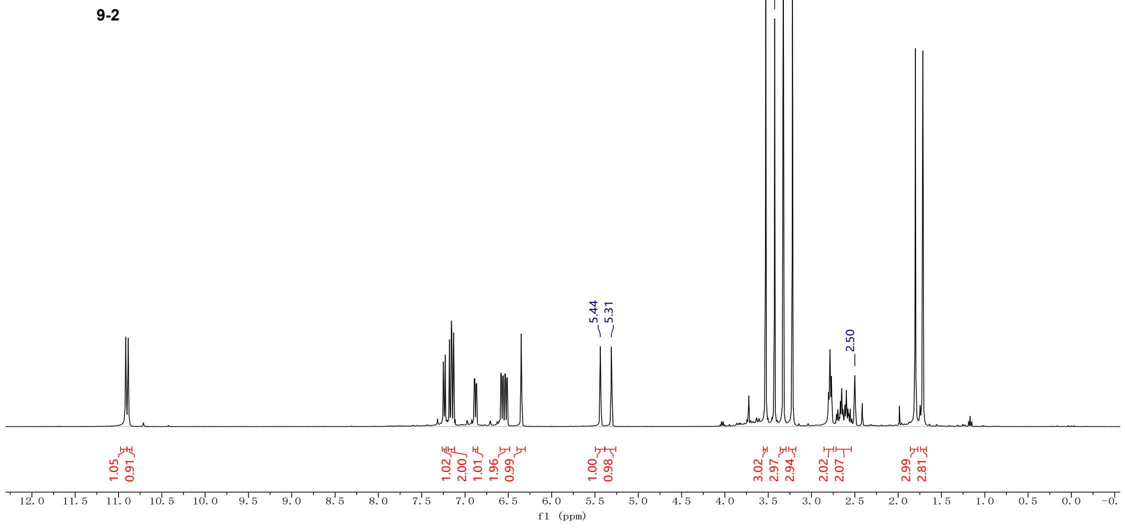

2018-2.15369.fid
LHL -2-87-1

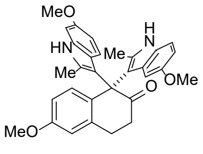

9-2

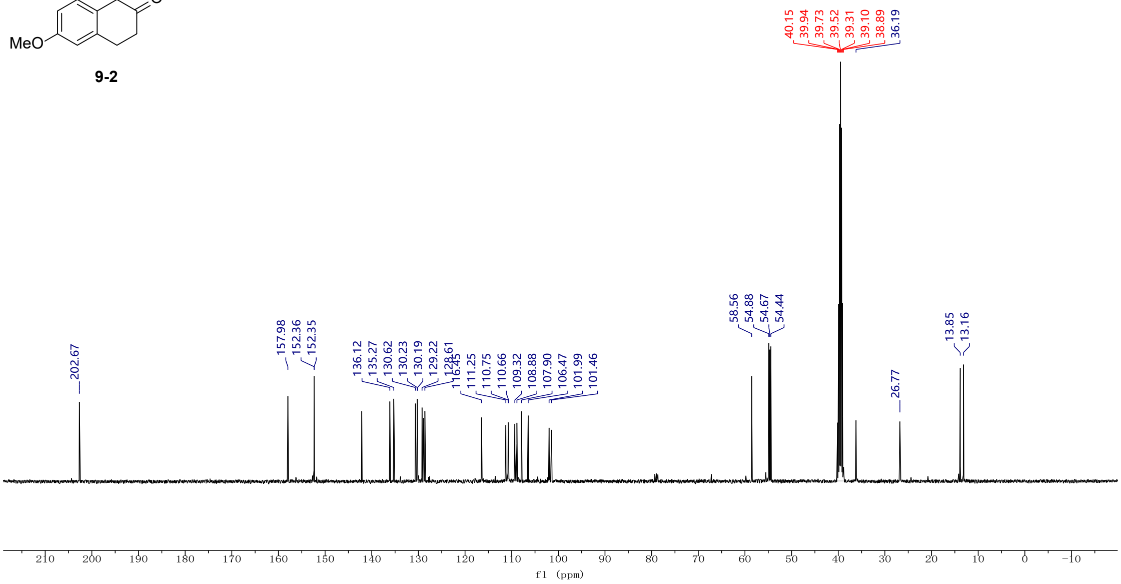

2018-2.9898.fid
LHL-2-46-1

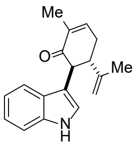

6-43

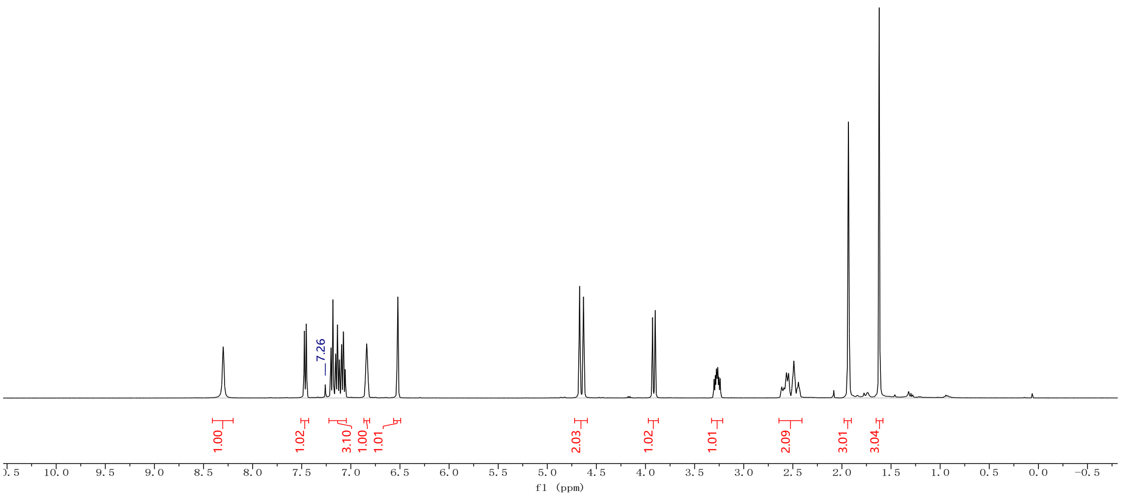

LHL-2-46-2-2

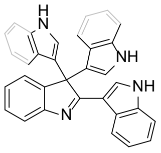

10 (tetramer)

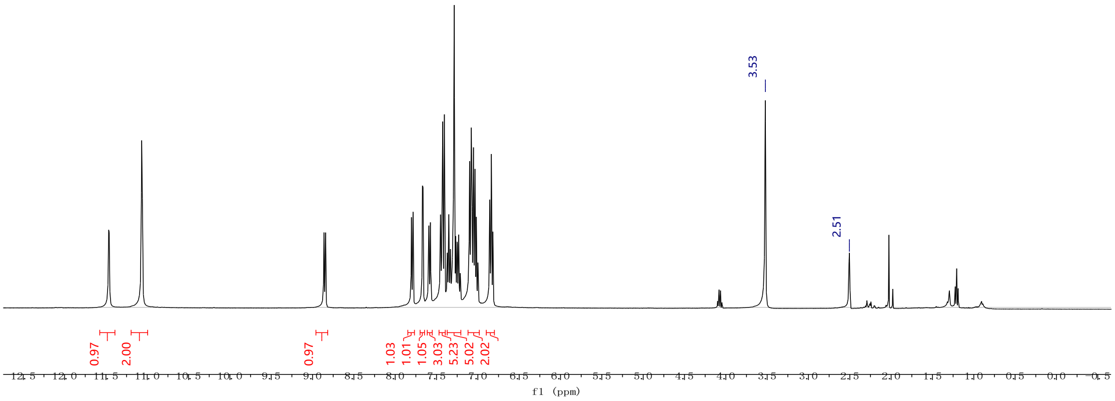

LHL1-75-2-1

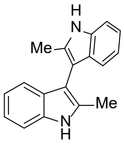

7-1

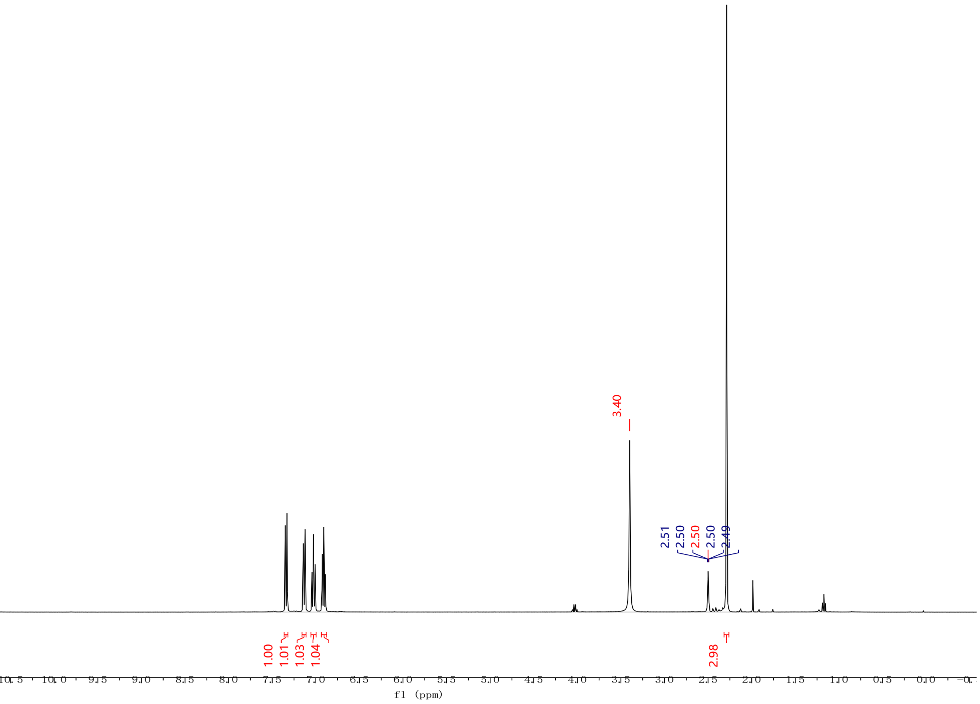

2018-2.10147.fid
LHL-2-47-2

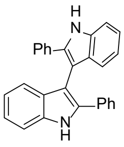

7-2

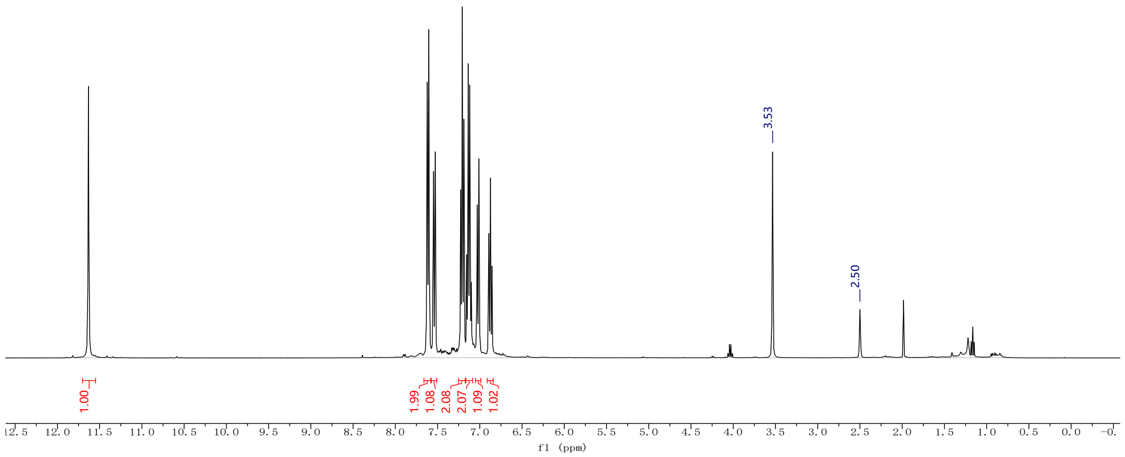

2019-1.46.fid
LHL-3-1-2

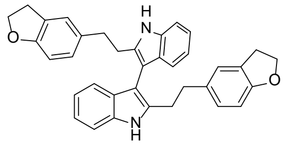

7-3

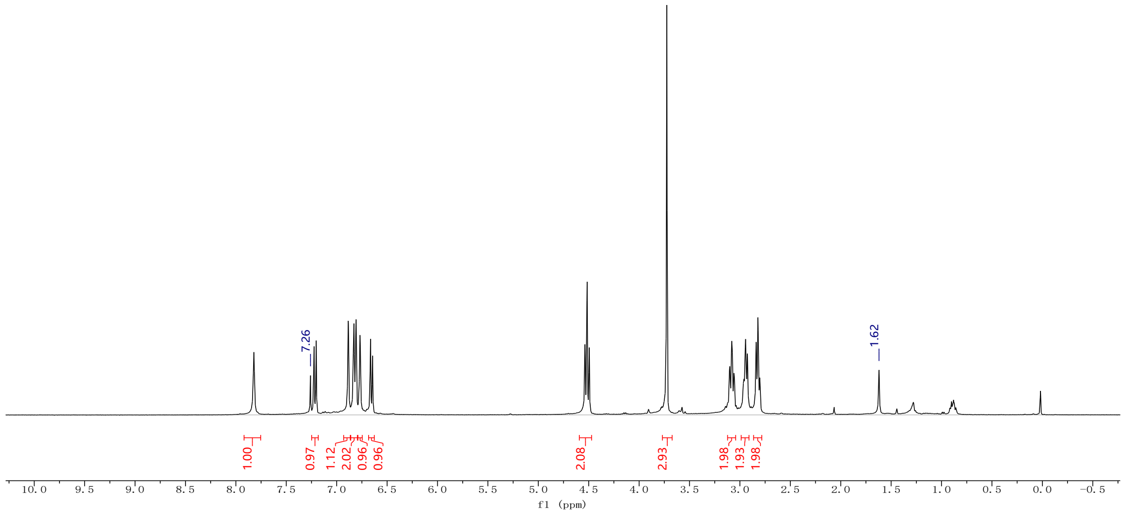

2019-1.718.fid
LHL -3-1-2

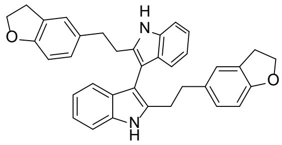

7-3

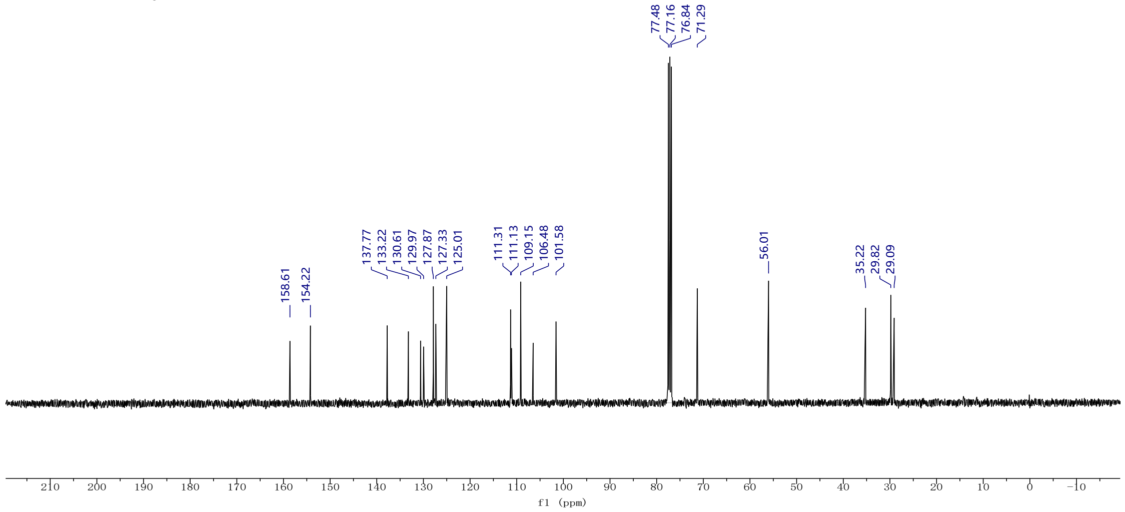

2019-1.15244.fid
lhl 4-30-2.1

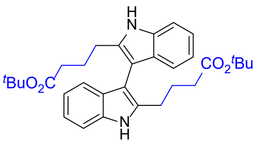

7-4

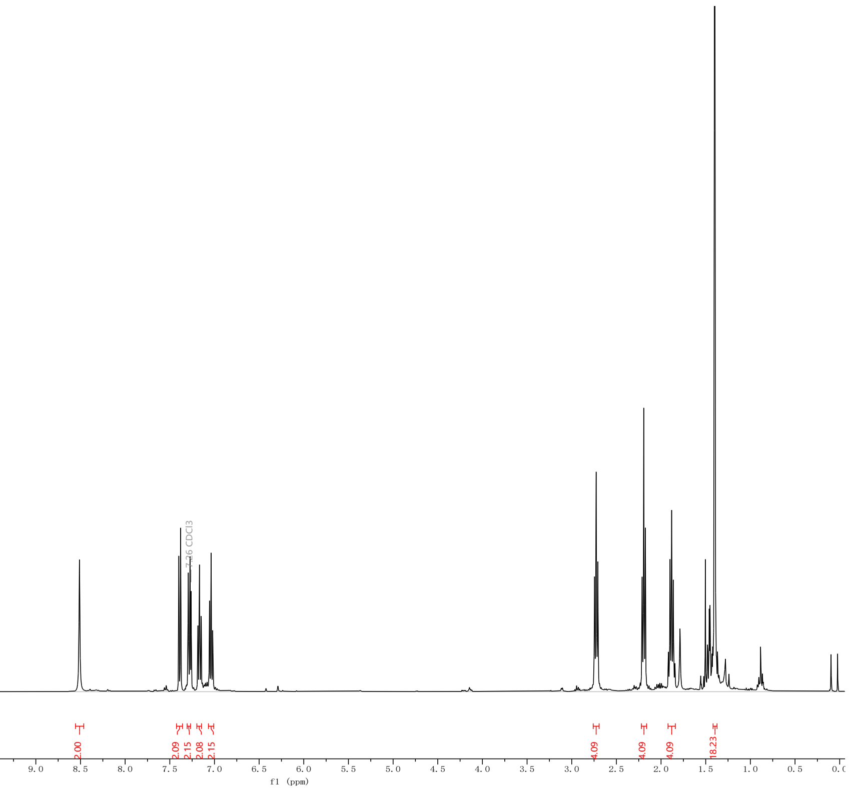

2019-1.15859.fid
LHL -4-30-2.1

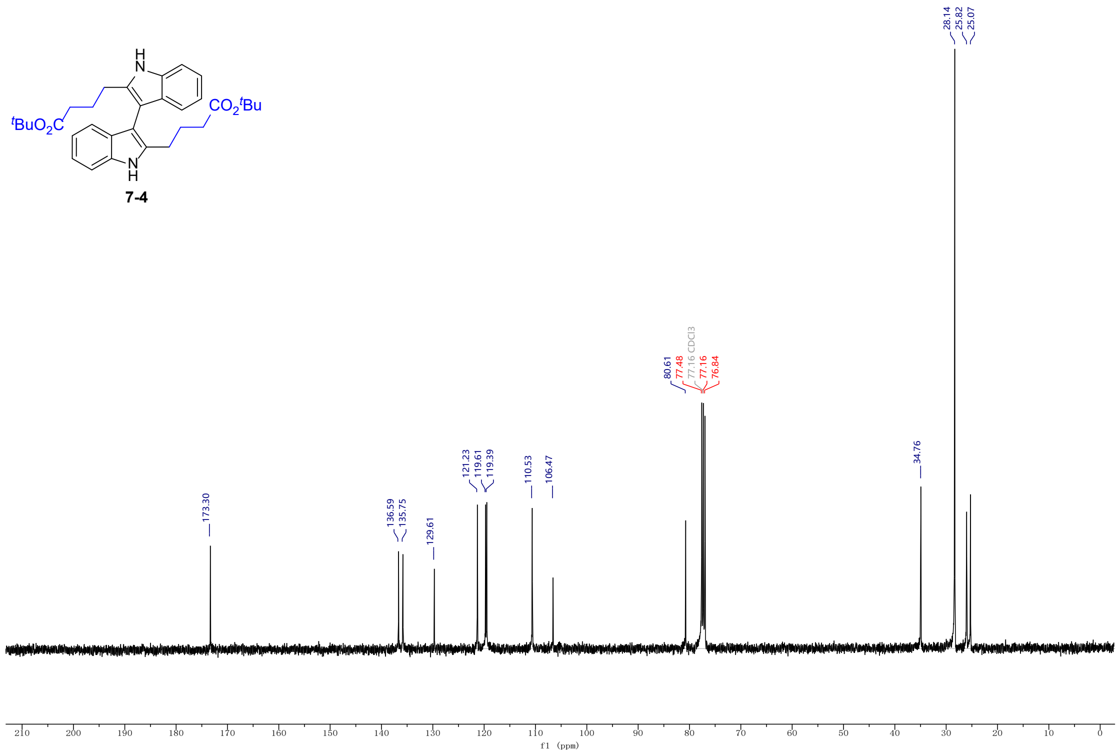

2018-2.14865.fid
LHL-2-88-2.1

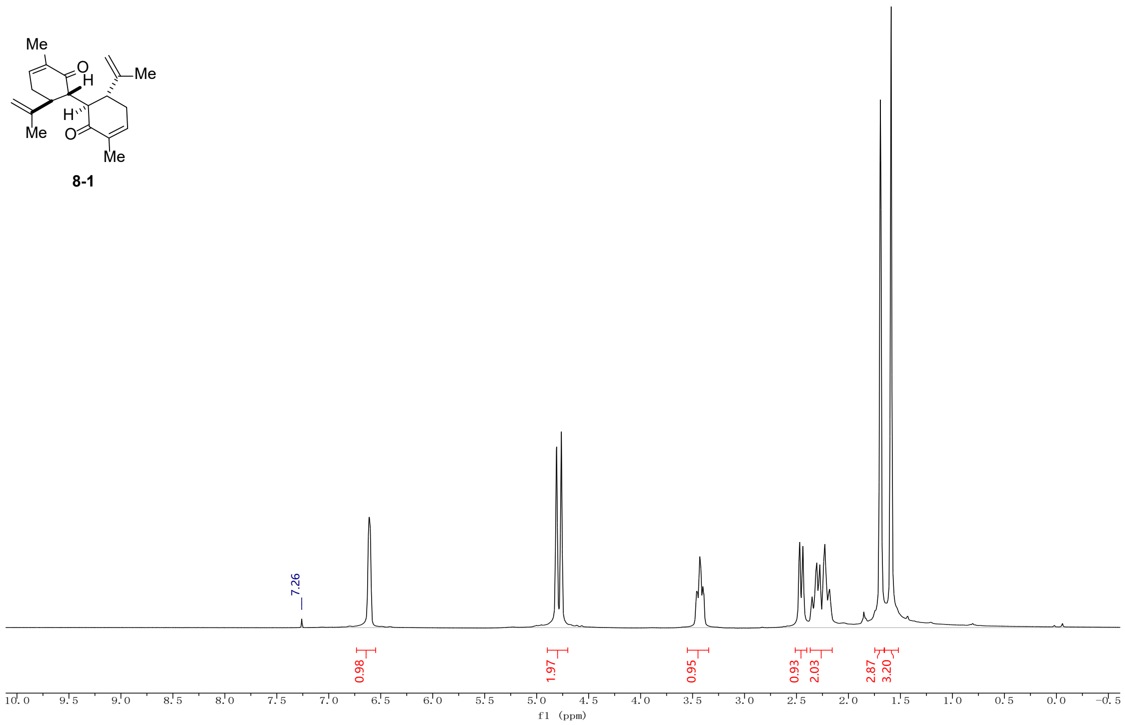

2018-2.10801.fid
LHL-2-55-2

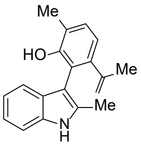

13-1

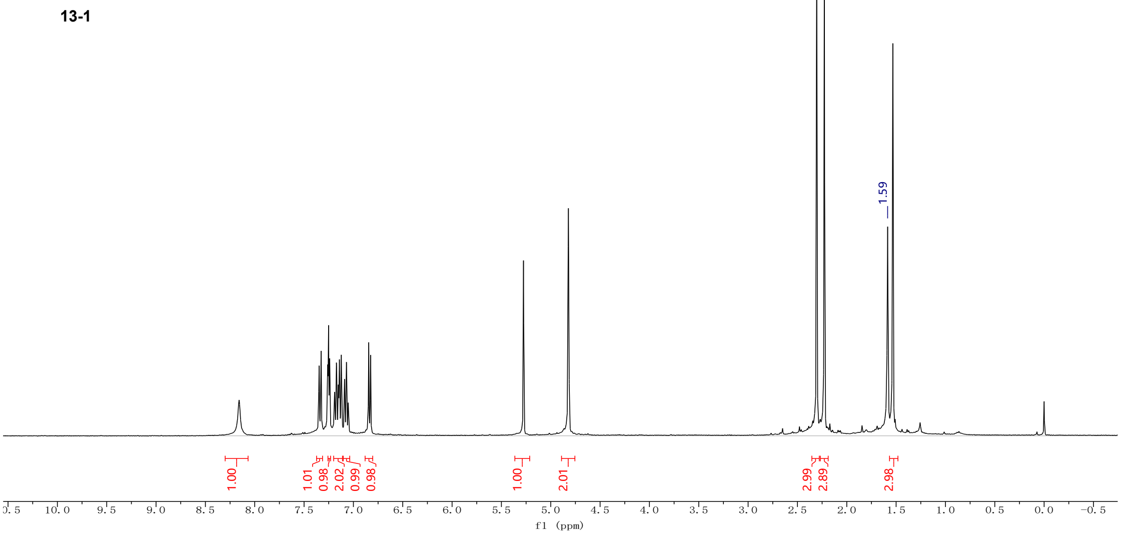

2019-1.3516.fid
LHL-3-20-1

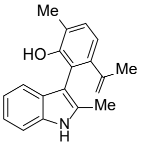

13-1

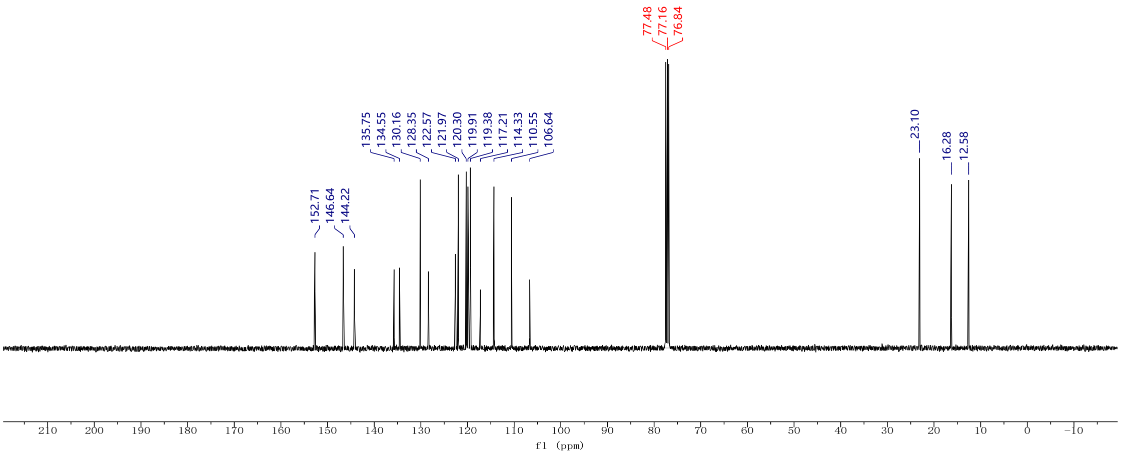

2018-2.13509.fid
LHL -2-79-1

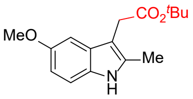

6-46

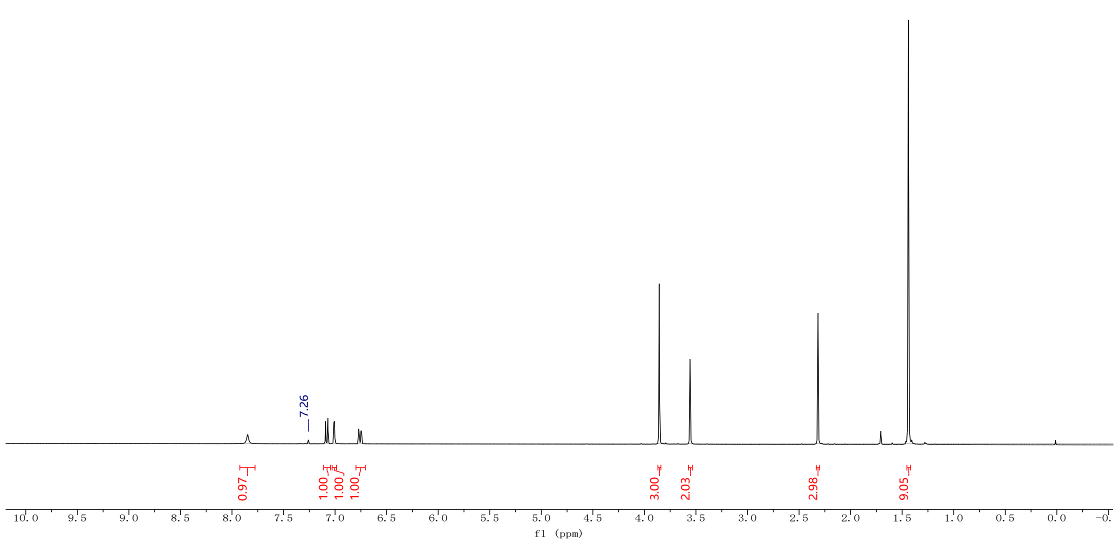

2018-2.14040.fid
LHL-2-79-1

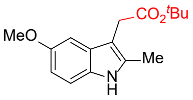

6-46

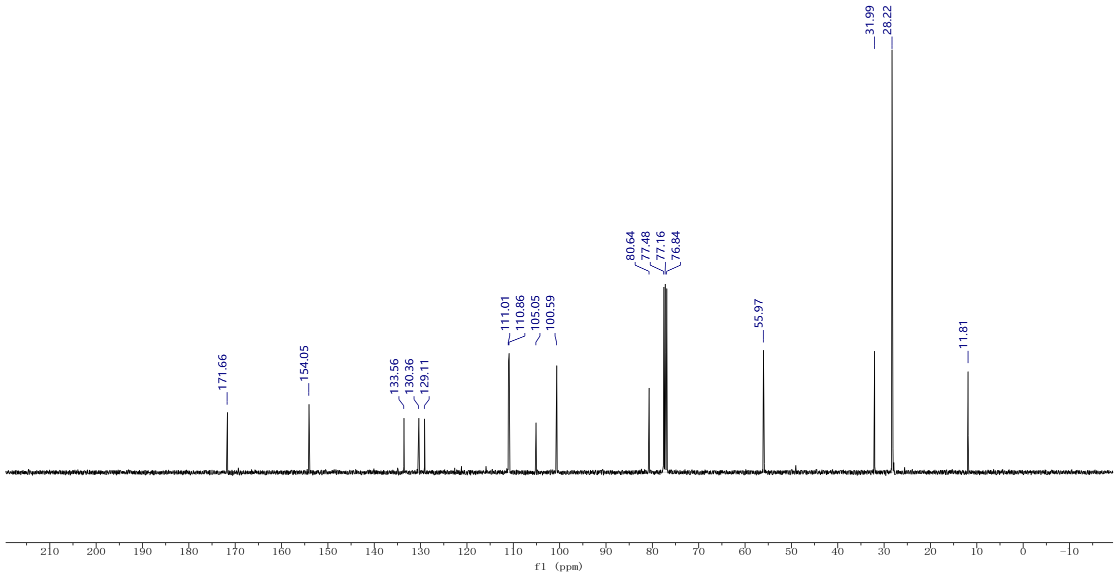

2019-1.2929.fid
LHL-3-19-1

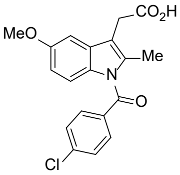

12-2, indomethacin

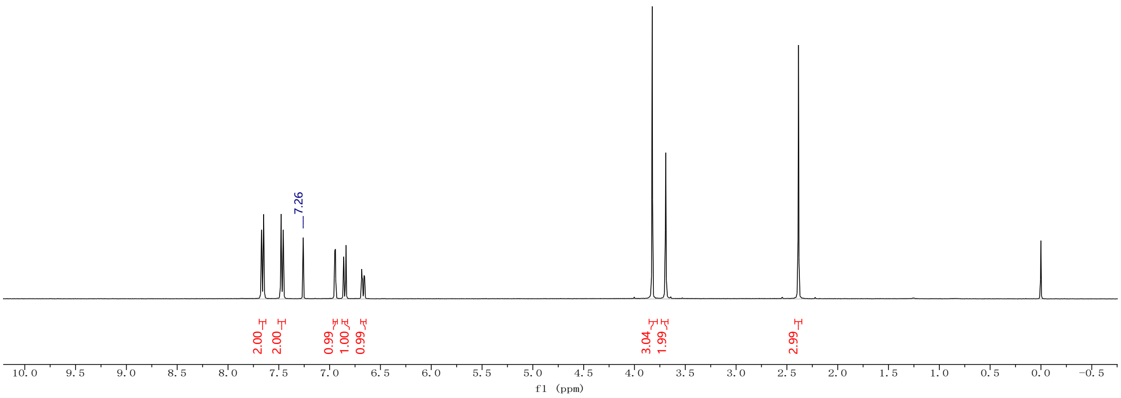

2018-2.12512.fid
LHT-2-68-1.1

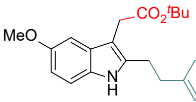

6-47

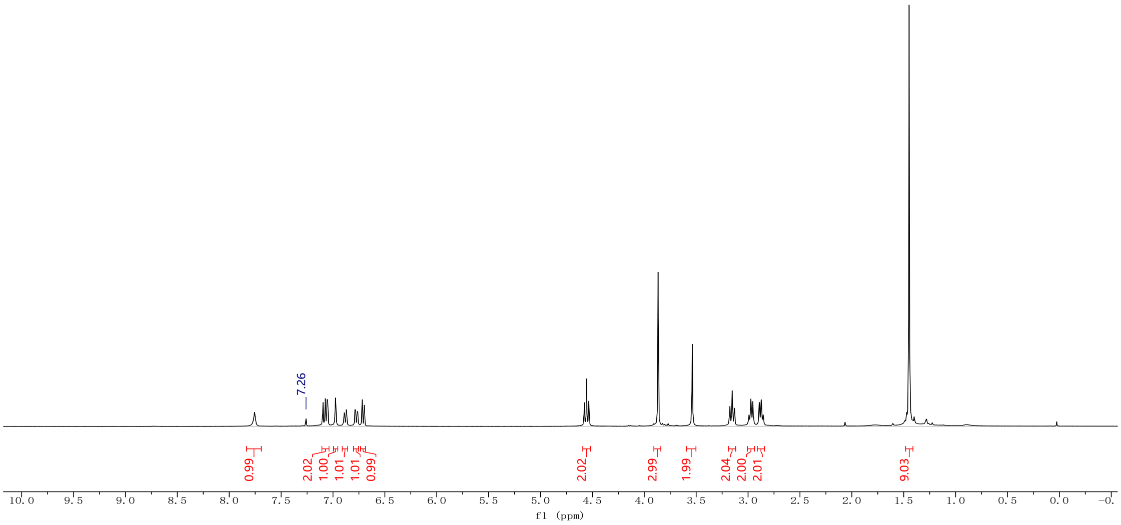

2018-2.13270.fid
LHL -2-68-1.1

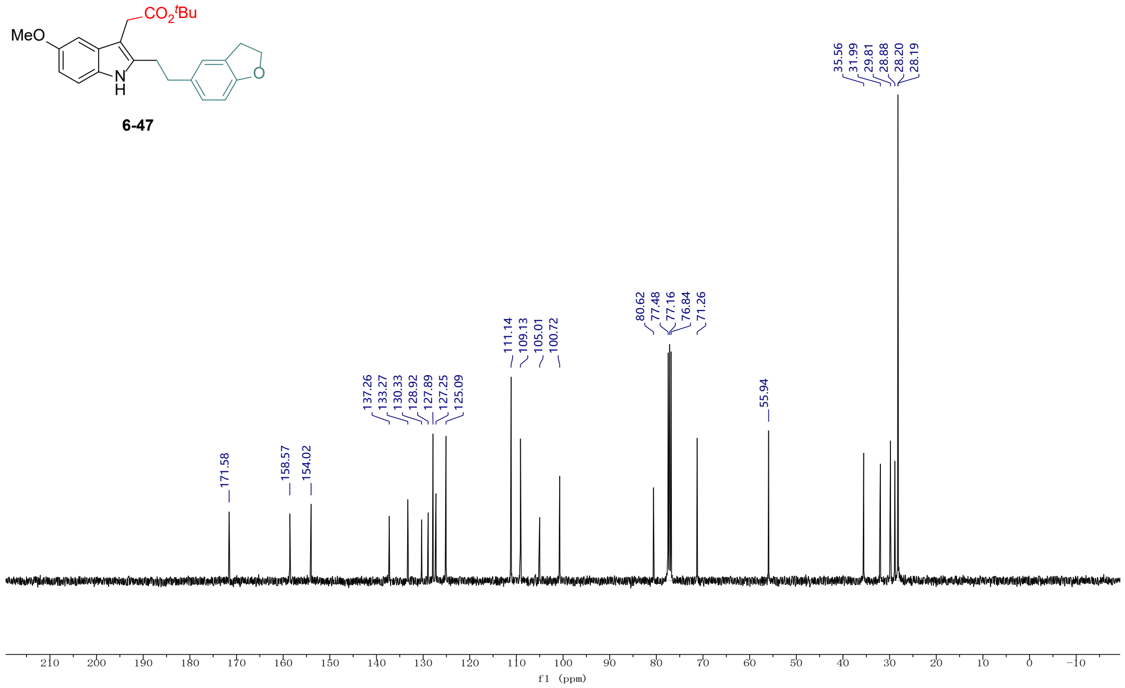

6-47

2019-1.3937.fid
LHL -3-25-1

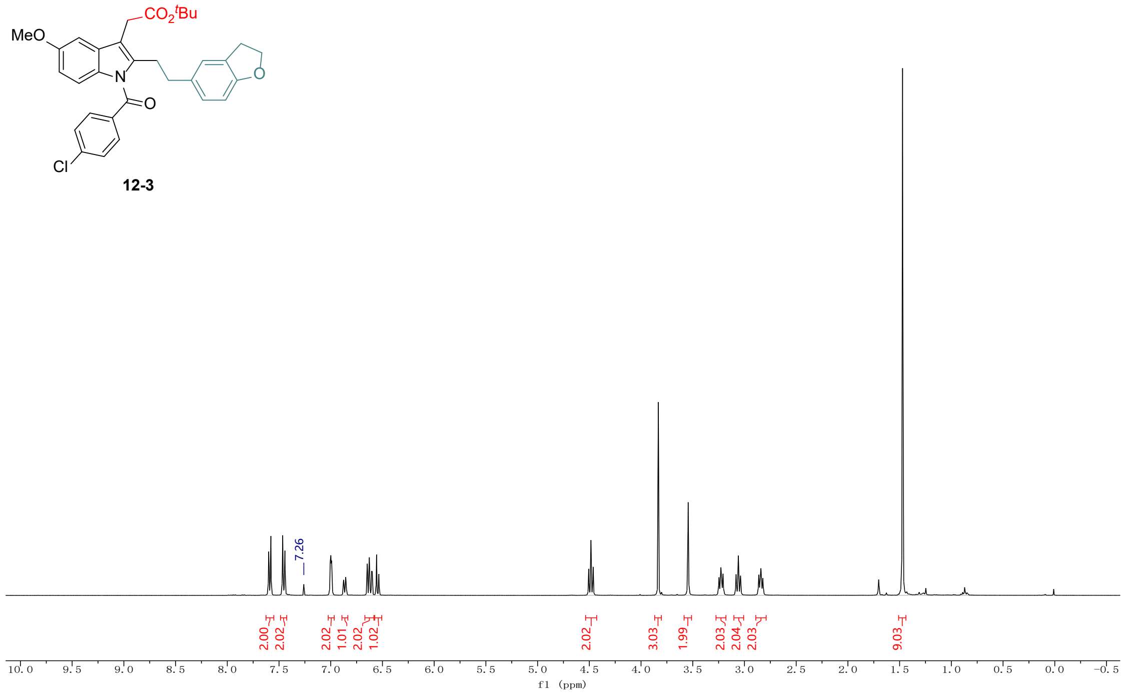

12-3

2019-1.4173.fid
LHL -3-25-1

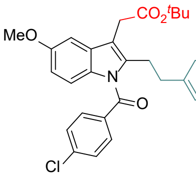

12-3

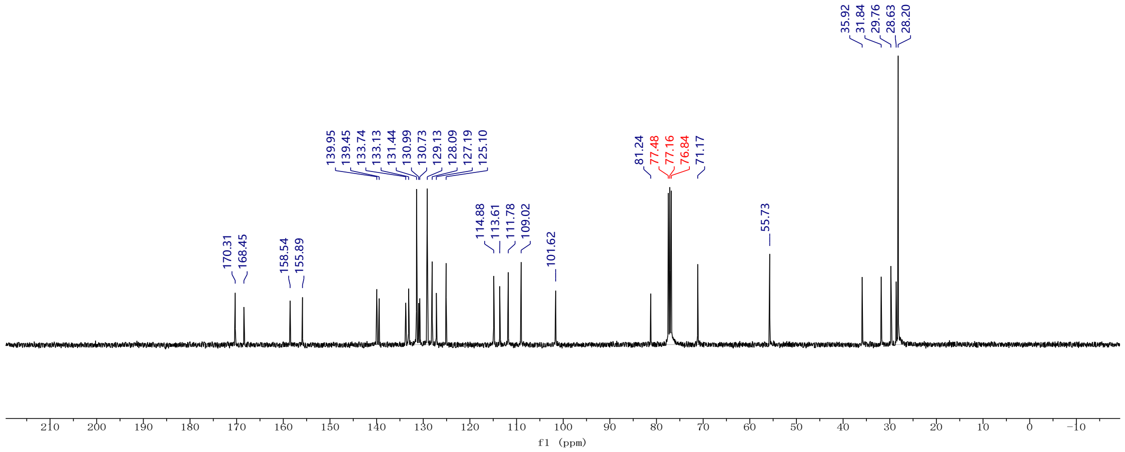

2019-1.5332.fid
LHL-3-39-1

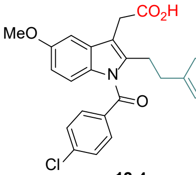

12-4

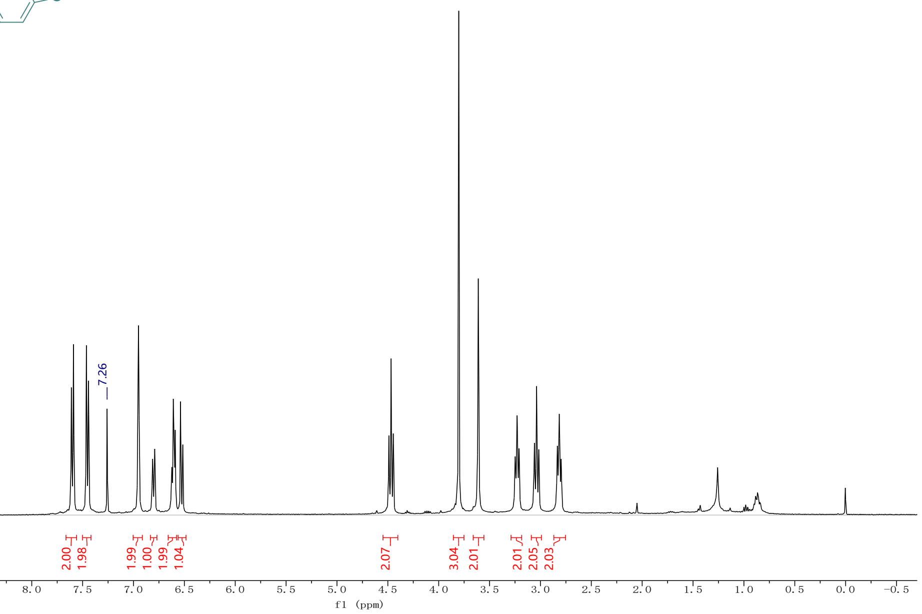

2019-1.5631.fid
LHL-3-39-1

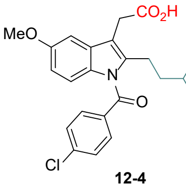

12-4

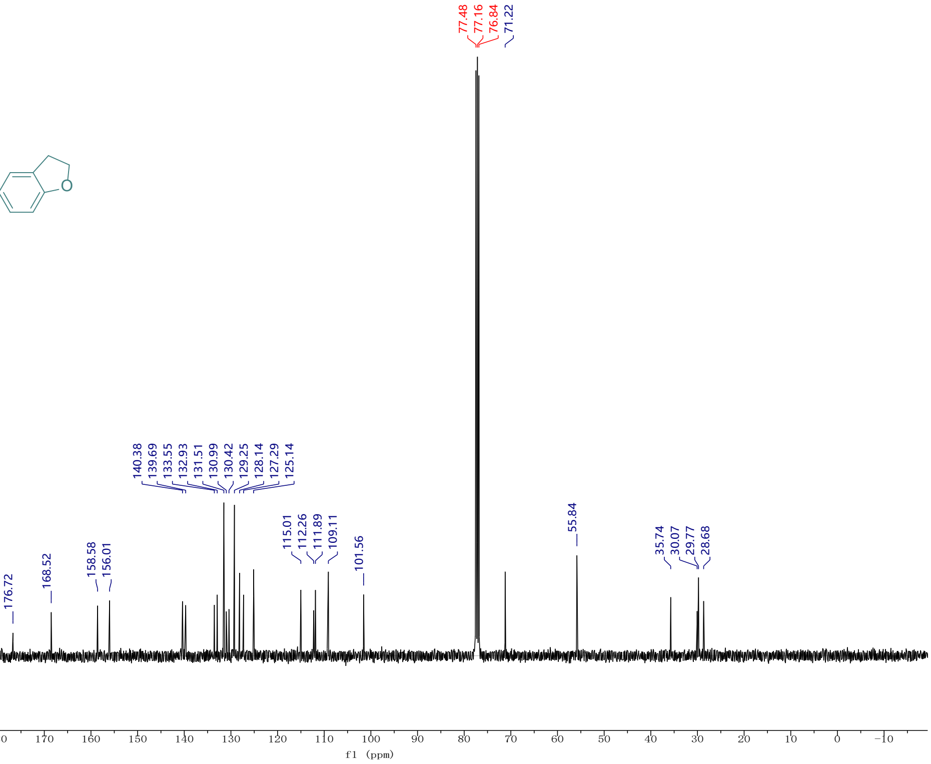

2018-2.13908.fid
LHL-2-80-1.2

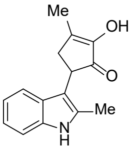

6-48

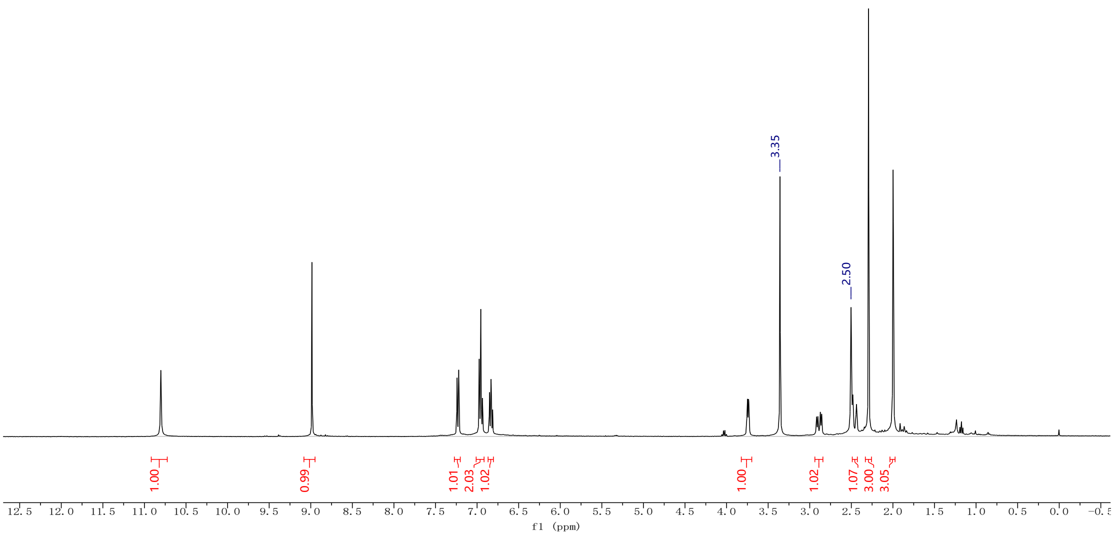

2019-1.2545.fid
LHL -2-80-1.2

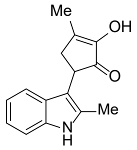

6-48

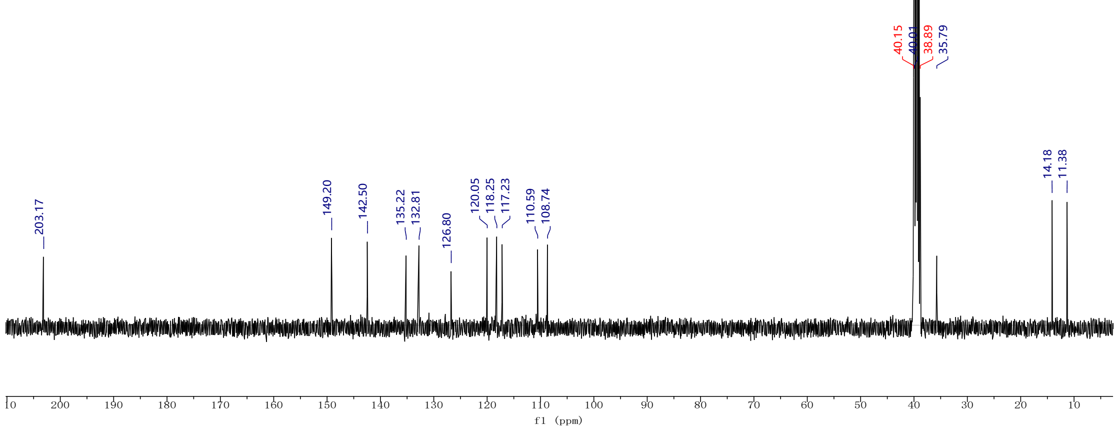

Supplement: Document S1. Transparent Methods, Figures S1–S9, and Tables S1–S3 [file mmc1.pdf]
